# Exploring T-cell metabolism in tuberculosis: development of a diagnostic model using metabolic genes

**DOI:** 10.1186/s40001-025-02768-0

**Published:** 2025-06-16

**Authors:** Shoupeng Ding, Chunxiao Huang, Jinghua Gao, Chun Bi, Yuyang Zhou, Zihan Cai

**Affiliations:** 1 Department of Laboratory Medicine, Gutian County Hospital, Gutian, 352200 China; 2 Department of Medical Laboratory, Siyang Hospital, Siyang, 237000 China; 3 Oncology and Laboratory Immunology Research Innovation Center, Siyang Hospital, Siyang, 237000 China; 4 Center for Precision Medicine, The People’s Hospital of Chuxiong Yi Autonomous Prefecture, Chuxiong, 675000 China

**Keywords:** Tuberculosis, T-cell metabolism, Machine learning biomarkers, Metabolic gene signatures, Molecular subtypes

## Abstract

**Objectives:**

The early diagnosis and immunoregulatory mechanisms of active tuberculosis (ATB) and latent tuberculosis infection (LTBI) remain unclear, and the role of metabolic genes in host–pathogen interactions requires further investigation.

**Methods:**

Single-cell RNA sequencing (scRNA-seq) was applied to analyze peripheral blood mononuclear cells (PBMCs) from 7 individuals, including 2 healthy controls (HC), 2 LTBI patients, and 3 ATB patients. We identified T-cell-associated metabolic differentially expressed genes (TCM–DEGs) through integrated differential expression analysis and machine learning algorithms (XGBoost, SVM–RFE, and Boruta). These TCM–DEGs were then used to construct a diagnostic model and evaluate its clinical applicability.

**Results:**

The analysis revealed significant immunological alterations in TB patients, characterized by markedly elevated monocyte/macrophage populations (*p* < 0.001) accompanied by reduced T and NK cell counts. Notably, LTBI cases demonstrated an intermediate CD4+/CD8+ T-cell ratio, indicative of dynamic immune homeostasis. The TB cohort exhibited increased inflammatory T-cell populations, while CD8+ T-cell-mediated MHC-I and BTLA signaling pathways were identified as key regulators of immune clearance and modulation.

Transcriptomic profiling identified five metabolically significant differentially expressed genes (FHIT, MAN1C1, SLC4C7, NT5E, AKR1C3; *p* < 0.05) that effectively distinguish between latent tuberculosis infection (LTBI) and active tuberculosis (TB). The machine learning-driven diagnostic framework demonstrated remarkable consistency across independent validation cohorts (GSE39940, GSE39939), exhibiting AUC values spanning 0.867–0.873. Molecular subtyping analysis delineated two distinct TB phenotypes: an immune-activated M1 macrophage-dominant subtype and a CD8 + T-cell infiltrated immunophenotype. Clinical validation substantiated the differential expression patterns of T-cell-related metabolic differentially expressed genes (TCM–DEGs; *p* < 0.05), while the nomogram predictive model achieved exceptional discriminative capacity (C-index = 0.944), demonstrating superior clinical applicability through decision curve analysis.

**Conclusions:**

Our findings reveal that TCM–DEGs critically regulate TB progression through immune–metabolic reprogramming and cell–cell communication networks. The developed diagnostic model and molecular subtyping strategy enable precise TB–LTBI differentiation and inform immunotherapy optimization.

**Supplementary Information:**

The online version contains supplementary material available at 10.1186/s40001-025-02768-0.

## Introduction

Tuberculosis (TB) is a granulomatous disease caused by Mycobacterium tuberculosis (Mtb), which remains a major global public health threat. According to the 2023 Global Tuberculosis Report by the World Health Organization, TB caused 1.25 million deaths, ranking as the second most deadly infectious disease globally, following COVID-19 [1]. Clinically, TB manifests in two primary forms: latent tuberculosis infection (LTBI), characterized by immune control, and active tuberculosis (ATB), marked by persistent tissue damage [2]. Although the majority of exposed individuals (90–95%) successfully eliminate Mtb, some develop persistent LTBI. Approximately 5–10% of these individuals will progress to active tuberculosis, depending on their immune status.[1]. Despite existing epidemiological data, the molecular mechanisms underlying the transition from LTBI to ATB remain poorly understood, thus rendering the effective differentiation between these two pathophysiological states a significant challenge in clinical diagnosis.

Currently, routine TB diagnostic methods predominantly rely on the tuberculin skin test (TST) and interferon-gamma release assays (IGRAs) [3, 4]. However, these methods exhibit clear limitations: they cannot effectively differentiate LTBI from ATB, nor can they predict which high-risk populations are likely to develop active tuberculosis. Therefore, there is an urgent need to identify novel biomarkers capable of accurately stratifying different stages of infection to overcome the current diagnostic bottleneck.

Recent studies have highlighted the pivotal role of immune–metabolism interactions in antimicrobial immunity. T-cell metabolism plays a central regulatory role in activation thresholds, clonal expansion, functional differentiation, and effector responses. T lymphocytes can dynamically transition between metabolic pathways, including glycolysis [5], oxidative phosphorylation (OXPHOS) [6, 7], fatty acid β-oxidation [8], and glutaminolysis [9, 10]. This transition is governed by immune demands and adapts to shifting bioenergetic requirements.

Notably, Mtb disrupts these metabolic pathways through a dual mechanism. It suppresses glycolysis and promotes lipogenesis, which creates an immunosuppressive microenvironment [11, 12]. In addition, Mtb-induced metabolic reprogramming enhances the immunosuppressive functions of regulatory T cells (Tregs), facilitating immune evasion and the persistence of chronic infection [13]. Despite these findings, systematic studies elucidating the specific role of T-cell metabolic networks in the LTBI-to-ATB transition are still lacking.

Contemporary research further underscores the vast potential of metabolic genomics in TB diagnosis and personalized treatment [14, 15]. Metabolic abnormalities not only serve as indicators of disease progression but also hold significant promise as biomarkers, particularly in chronic infections such as tuberculosis. In these instances, the dynamic interplay between immunity and metabolism profoundly influences diagnostic accuracy and therapeutic outcomes.

**Fig. 1:**
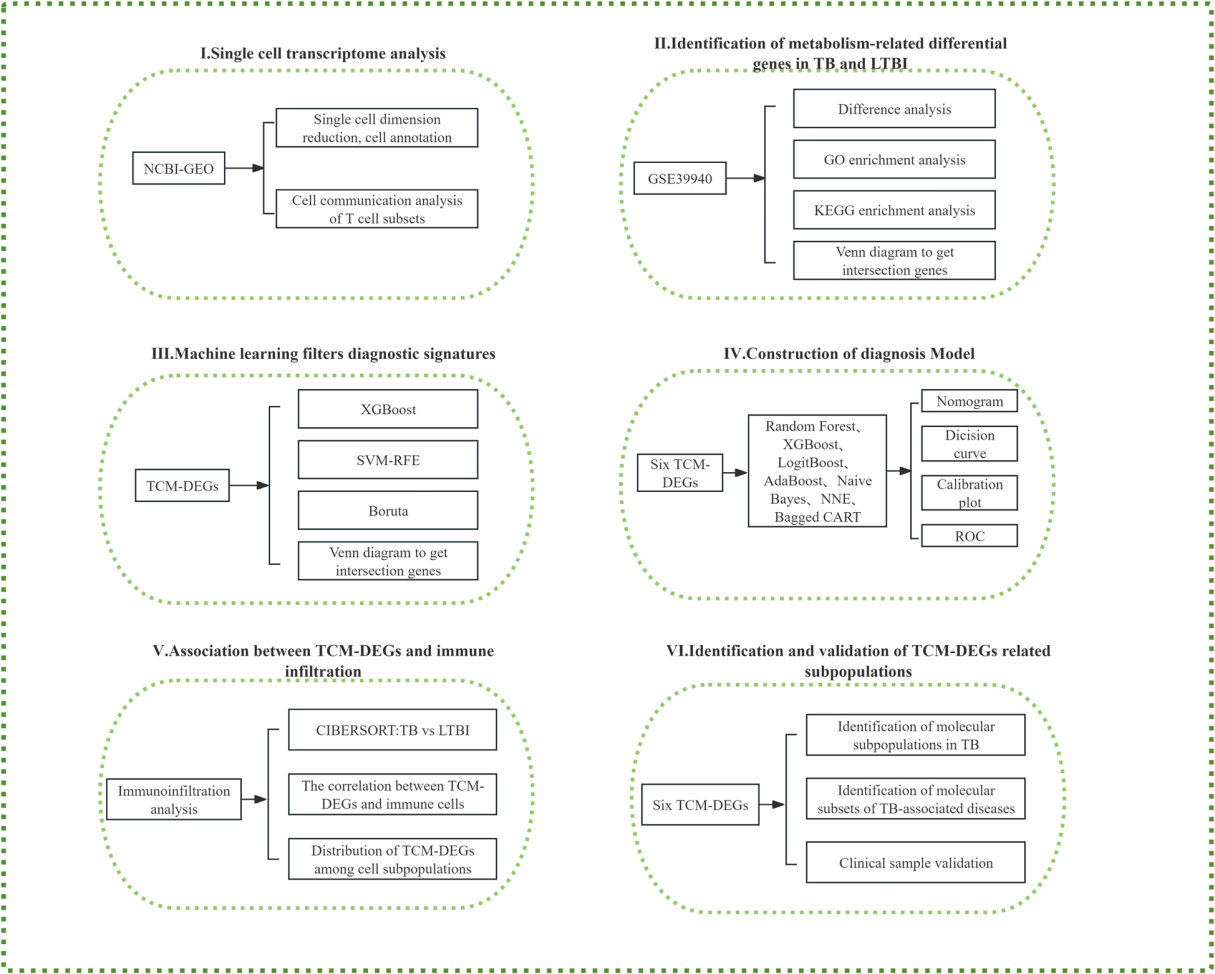
Experimental flow

To elucidate these mechanisms, we employed a variety of methodologies, including single-cell transcriptomics, cross-cohort bioinformatics analysis, and machine learning, to systematically explore the relationship between T-cell-specific metabolic pathways and tuberculosis disease status. Specifically, we will: (1) perform single-cell transcriptomic analysis of peripheral blood mononuclear cells (PBMCs) from tuberculosis patients; (2) in conjunction with multi-cohort bioinformatics techniques, identify differentially expressed genes specific to T-cell metabolism (TCM–DEGs); (3) develop machine learning-based diagnostic algorithms, grounded in the stratification of LTBI and ATB; and (4) conduct molecular classification based on the expression profiles of key genes, integrated with comprehensive biological characterization. Furthermore, the clinical relevance of the identified biomarkers is confirmed through rigorous experimental validation. This research framework (Fig. 1) not only advances the development of tuberculosis diagnostics but also provides a conceptual foundation for metabolism-targeted precision medicine, potentially offering crucial insights for the design of personalized treatment strategies.

## Materials and methods

### Data sources

Transcriptomic data sets associated with tuberculosis pathogenesis were systematically retrieved from the NCBI Gene Expression Omnibus (GEO) repository, comprising three primary TB-related cohorts (GSE39939, GSE39940, GSE28623) and three supplementary validation data sets (GSE93272, GSE162635, and GSE50772).The detailed information is provided in Table 1. Immunometabolic gene signatures were curated through integrative analysis of the Molecular Signatures Database (MSigDB), KEGG pathway annotations, and consensus findings from systematic reviews, as comprehensively documented in Supplementary Table 1.

**Table 1:** Microarray information of gene expression data set

Type	Data set	Source	Sample			Platform
			HC	LTBI	Abnormal	
GEO	GSE39939	PBMC	–	64	93 (TB)	GPL10558
	GSE39940	PBMC	–	54	111 (TB)	GPL10558
	GSE28623	PBMC	37	25	46 (TB)	GPL4133
	GSE93272	PBMC	42	–	233 (RA)	GPL570
	GSE162635	PBMC	16	–	185 (COPD)	GPL570
	GSE50772	PBMC	20	–	61 (SLE)	GPL570

### Single-cell transcriptomic analysis

The original single-cell RNA sequencing (scRNA-seq) data were retrieved from the NCBI Sequence Read Archive (SRA), including seven peripheral blood mononuclear cell (PBMC) samples. This data set consists of two healthy control samples (SRR11038994, SRR11038995) and two samples from individuals with latent tuberculosis infection (LTBI) (SRR11038992, SRR11038993). In addition, three patients with active tuberculosis (ATB) (SRR11038989–SRR11038991) were included.

Data preprocessing was performed in R using the Seurat software package (v5.1.0). Cell filtration was conducted according to standard quality control thresholds: cells expressing fewer than 200 genes or more than 6,000 genes, or those with mitochondrial gene expression exceeding 20%, were excluded, while genes expressed in at least three cells were retained. The SCTransform method was applied to standardize the gene expression matrix. This method performs variance-stabilized transformation and eliminates technical noise. To mitigate the impact of batch effects and technical variations on the analysis outcomes, we utilized the FindIntegrationAnchors() and IntegrateData() functions from Seurat during the data integration process. These methods effectively integrate data from distinct groups (ATB, LTBI, and HC), mitigate batch effects, ensure data consistency, and minimize the impact of technical noise on subsequent analyses (such as clustering and differential expression analysis). Furthermore, given the relatively small sample sizes of the LTBI and HC groups, we employed a weighted linear model and an excessive discrete normalization method in the differential expression analysis to balance sample size disparities, reduce potential bias from sample imbalance, and enhance the robustness of the findings. Dimensionality reduction and cluster analysis were performed using Principal Component Analysis (PCA) and Uniform Manifold Approximation and Projection (UMAP). Clustering analysis was performed with a resolution of 0.6, and the FindClusters function was employed to identify the major clusters. Cell-type annotation was integrated based on predictions from CellMarker 2.0, PanglaoDB, and ScType algorithms, and further validated using marker gene signatures corroborated in the literature to ensure biological accuracy.

### Cell–cell communication analysis

Ligand–receptor interaction dynamics were deconvoluted using CellChat (v1.6.0), leveraging its comprehensive repository of 2298 experimentally validated molecular pairs [16].Communication probability matrices were computed through weighted summation of co-expression patterns, while the patchwork framework facilitated cross-sample integration of single-cell expression profiles, effectively mitigating technical variability while preserving biological heterogeneity. This analytical paradigm enabled systematic mapping of autocrine/paracrine signaling axes, dynamic visualization of pathway-specific communication fluxes, and mechanistic interpretation of immunometabolic crosstalk within the tuberculosis microenvironment.

### Identification of metabolism-related differential genes

T-cell-specific metabolic differentially expressed genes (TCM–DEGs) were identified through limma-based differential expression analysis (|log2 fold change|> 1; Benjamini–Hochberg adjusted *p* < 0.05). Multidimensional functional characterization was performed using clusterProfiler (v4.0) for Gene Ontology (GO) biological process enrichment, hypergeometric testing for KEGG pathway mapping (false discovery rate < 0.05), and pathview-mediated visualization of pathway perturbation states. This integrative strategy elucidated three critical mechanistic axes: metabolic checkpoints regulating T-cell differentiation, nutrient-sensing pathways modulating immune effector functions, and pathogen-driven metabolic subversion mechanisms facilitating immune evasion.

### Machine learning-based selection of metabolism-related genes

A comprehensive machine learning-based method was employed to identify key metabolic genes, integrating three algorithms: XGBoost [17], support vector machine–recursive feature elimination (SVM–RFE) [18], and the Boruta algorithm [19]. XGBoost (extreme gradient boosting, XGBoost v1.7.8.1): a gradient boosting framework with built-in feature importance ranking. SVM–RFE (recursive feature elimination of support vector machine, e1071 v1.7–16): a regression feature elimination method based on a linear kernel SVM. Boruta (Boruta v8.0.0): an encapsulation algorithm based on the random forest classifier, used to identify all relevant features.

Each algorithm operates with default hyperparameters, and genes consistently selected by all three algorithms are considered stable features. The intersected genes were visualized using Venn plots. This multi-algorithm approach ensures the stability of the feature selection process and avoids the bias of any single model.

### Construction of a machine learning-based diagnostic model

Based on the selected TCM–DEGs, seven machine learning models were constructed: random forest, XGBoost, LogitBoost, AdaBoost, Naive Bayes, neural networks (NNET), and bagged CART. All models were implemented using the caret package (v6.0–94) in R, with the following additional dependencies: random forest, XGBoost, fastAdaboost, e1071, and nnet, which were used to implement the classifiers. The machine learning parameters are provided in Supplementary Table 1. The data set was randomly split into a training set (70%) and a test set (30%), ensuring stratification by phenotype (HC, LTBI, ATB) to maintain class balance.

A tenfold cross-validation strategy was applied to the training set to tune the hyperparameters and prevent overfitting. Model performance was evaluated using accuracy, sensitivity, specificity, and the area under the receiver operating characteristic (ROC) curve (AUC). Finally, an independent external validation data set was used for validating the models to assess their stability and generalizability.

### GSVA

To investigate the regulatory mechanisms of T-cell-specific metabolic differentially expressed genes (TCM–DEGs), this study performed functional enrichment analysis using the Gene Set Variation Analysis (GSVA) method. KEGG/GO gene sets from MSigDB (v7.4) were used to calculate pathway activity scores with the GSVA package (v1.46.0) [20]. Differential score analysis was performed using the limma package (v3.56.0) (|*t* value|> 2, *p* < 0.05). Key immune-related pathways, carbohydrate metabolism, and extracellular matrix metabolism pathways were identified. Gene Set Enrichment Analysis (GSEA) was further employed for pre-ranked pathway analysis to validate enrichment significance (FDR < 0.05).

### Nomogram construction and validation

Based on the identified core genes, a nomogram model was constructed using the"rms"package in R. The"Total points"reflected the contribution scores of predictive variables, with each variable being assigned a corresponding score based on its importance. The model’s accuracy and clinical applicability were systematically validated through calibration curves, decision curve analysis (DCA), and clinical impact curves.

### Consensus clustering analysis

Consensus molecular clustering was implemented via the ConsensusClusterPlus package (v1.68.0) in R environment, leveraging expression profiles of hub TCM–DEGs [21]. The analytical framework employed the following parameter configuration: maximum cluster count (maxK) = 9, 50 bootstrap iterations (reps) to ensure algorithmic stability, 80% sample sampling rate (pItem) = 0.8, full feature retention (pFeature) = 1, partitioning around medoids (PAM) algorithm for cluster delineation, and Euclidean distance metric for similarity quantification. Cluster robustness was validated through multi-criteria validation incorporating the Calinski–Harabasz index and intercluster correlation analysis, thereby delineating TB molecular subtypes with distinct immunometabolic characteristics.

### Evaluation of immune cell infiltration

To assess the impact of TB patient subtypes on immune phenotypes, the CIBERSORT algorithm was used to analyze immune cell infiltration levels in different subtypes [22]. This approach facilitated an in-depth exploration of immune microenvironment differences among subtypes and provided insights into the relationship between T-cell metabolic subtypes and immune responses.

### Quantitative real-time PCR (RT-qPCR)

To further validate the expression levels of the selected T-cell-related differentially expressed genes (DEGs), this study included 20 peripheral blood samples, comprising 10 patients diagnosed with active tuberculosis (TB group) and 10 patients with latent tuberculosis infection (LTBI group). All participants provided written informed consent.

The inclusion criteria were as follows: age between 18 and 65 years, with patients in the TB group diagnosed through sputum smear, culture, or nucleic acid testing for Mycobacterium tuberculosis. Subjects in the LTBI group tested positive for IGRA (Interferon-γ release assay) or TST (tuberculin skin test) and exhibited no clinical or imaging manifestations of active pulmonary tuberculosis. The exclusion criteria included severe immune system diseases, diabetes, malignant tumors, or other major chronic underlying conditions. Significant differences were observed between the two groups in demographic characteristics, including age (see Supplementary Table 2 for details).

Following the collection of whole blood samples, total RNA was extracted using the TRIzol method, and its purity was assessed before reverse transcription to synthesize cDNA. Primers were designed using Primer 3 Plus and verified for specificity via NCBI BLAST, and their sequences are listed in Table 2. RT-qPCR was performed according to the SYBR Green kit instructions, and amplification was conducted using the StepOnePlus system, with β-actin as the internal reference gene.

**Table 2:** Primers sequence

Gene	Primer (5′–3′)	
β-Actin	F:TGGCAAAACGTCTTCAGGAGG	R:AGCTTGACTTAGTGGCTTTGG
FHIT	F:ATCTCATCAAGCCCTCTGTAGT	R:GGACGCAGGTCATGGAAGC
MAN1C1	F:CCGCTTTGACTTCAACGCATT	R:CATAACGCTTATAGCTCTGCCAA
MAN1A1	F:AATATACGCTTTGTTGGTGGACT	R:GCAATGCCCAAGGTATTCCAG
SLC4A7	F:ATCTTGGCAAAACTAGCTCAACT	R:CGACTCTCTTTACTAAACGGGAC
NT5E	F:GCCTGGGAGCTTACGATTTTG	R:TAGTGCCCTGGTACTGGTCG
AKR1C3	F:GTCATCCGTATTTCAACCGGAG	R:CCACCCATCGTTTGTCTCGTT

All experiments were repeated in triplicate for each sample. Gene expression levels were calculated using the 2^−ΔΔCt^ method. Normality of the data was tested using the Shapiro–Wilk test. For normally distributed data, independent *t* tests were used for inter-group comparisons, while Mann–Whitney *U* tests were employed for non-normally distributed data. Statistical analysis was conducted using GraphPad Prism 10.1, and a *p* value of < 0.05 was considered statistically significant.

#### Statistical analysis

Nonparametric Wilcoxon rank-sum tests were used to analyze continuous variables between groups, with *p* < 0.05 considered statistically significant. All statistical analyses were conducted using R (version 4.3.2) and Prism 10 (GraphPad Software, USA).

## Results

### Diversity and functional insights of multi-subtype immune cell lineages

**Fig. 2:**
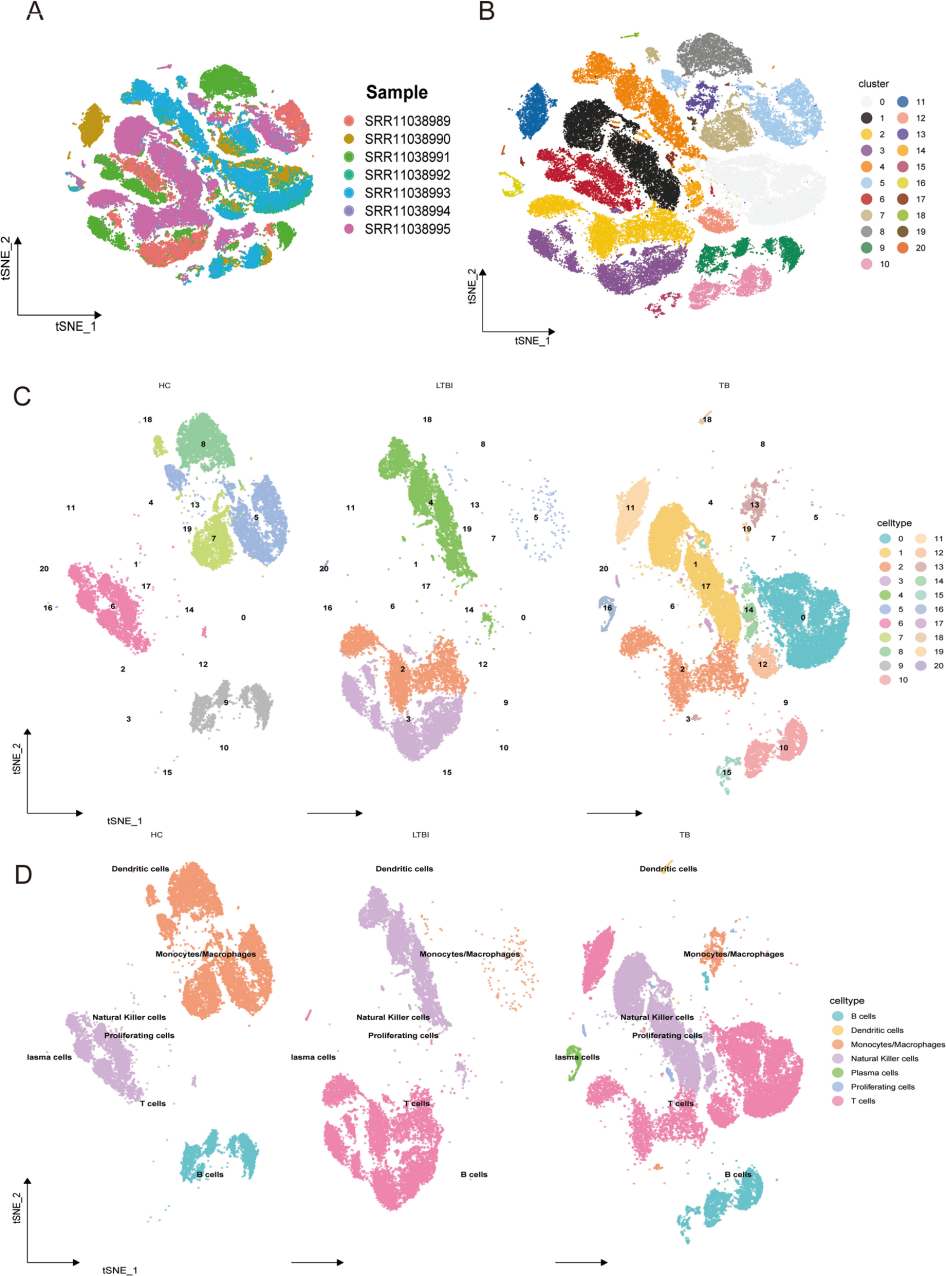
Cell subpopulation annotation diagram.** A** t-SNE plot of samples, with each point representing a single cell. Cells are colored by their sample of origin (SRR11038989 to SRR11038995). **B** t-SNE plot showing the clustering of cells into 20 distinct clusters. Each cluster is represented by a different color. **C** t-SNE plots displaying cell distribution across different conditions: healthy controls (HC), latent tuberculosis infection (LTBI), and tuberculosis (TB), with colors representing different cell types. **D** t-SNE plots with annotated cell types, including dendritic cells, monocytes/macrophages, NK cells, plasma cells, proliferating cells, T cells, B cells, and isema cells, for each condition (HC, LTBI, and TB)

Through the integration of single-cell sequencing with t-SNE/UMAP dimensionality reduction and unsupervised clustering across multiple samples, we successfully delineated several functionally distinct subpopulations, including dendritic cells, proliferative cells, plasma cells, B cells, T cells, NK cells, and monocytes/macrophages (Fig. 2A–D). These cellular subsets demonstrated both overlapping features and distinctive characteristics across samples, revealing heterogeneous transcriptional profiles and functional states among healthy controls (HC), latent tuberculosis infection (LTBI), and tuberculosis (TB) patients.

Comprehensive cell-type annotation coupled with gene expression profiling unveiled substantial immune cell infiltration beyond conventional reproductive cell populations, suggesting an intricately orchestrated microenvironment. The systematic classification of immune cells (e.g., classical DCs, B cells, T cells, NK cells, monocytes/macrophages) provided unprecedented insights into immune regulatory mechanisms under both physiological homeostasis and pathological conditions. Collectively, these findings furnish robust molecular evidence for deciphering multicellular crosstalk, signal transduction networks, and functional specialization in LTBI and TB patients, thereby establishing a critical foundation for elucidating disease pathogenesis and developing targeted therapeutic interventions.

**Fig. 3:**
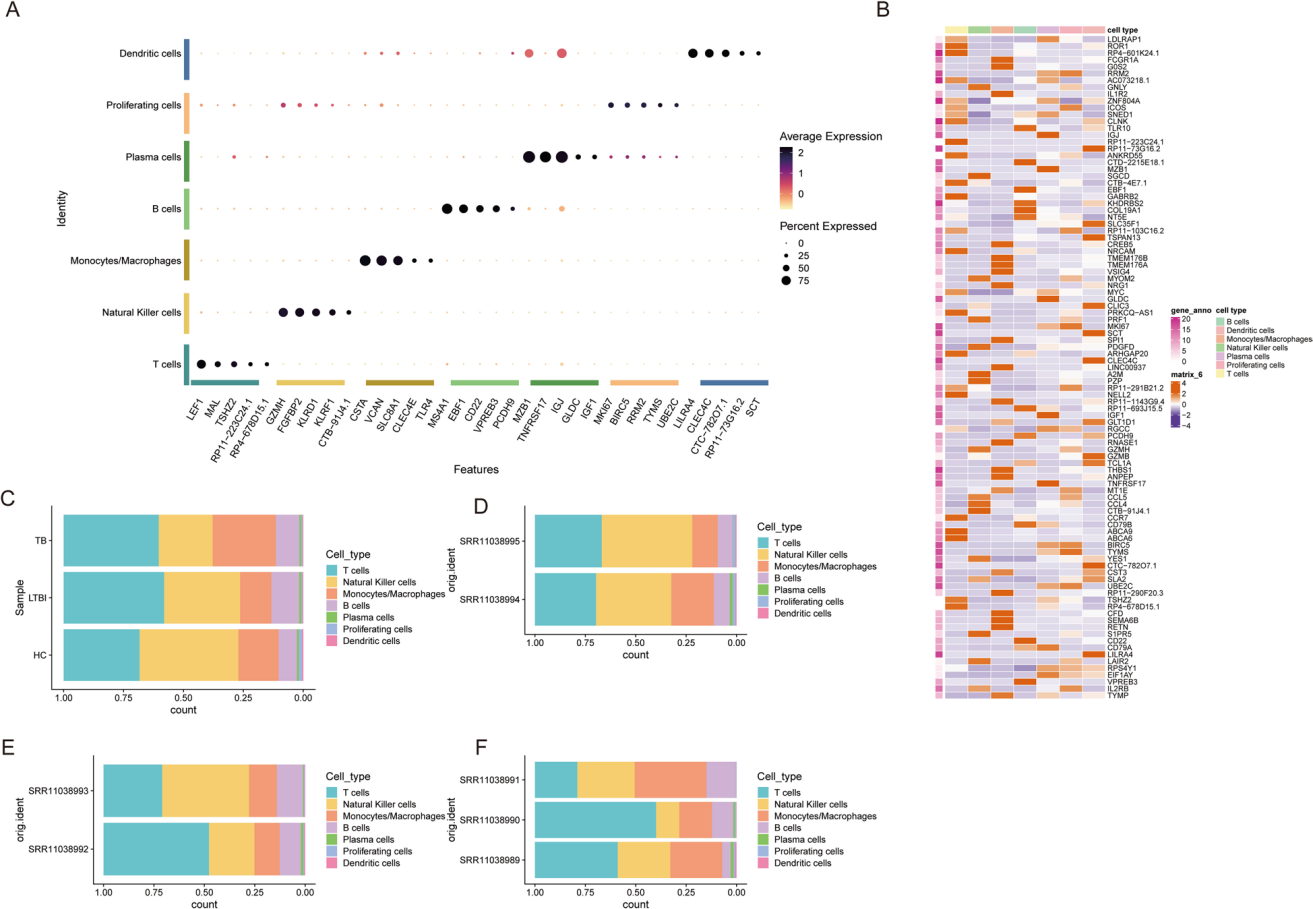
Characterization of cell subpopulations. **A** Dot plot showing key marker gene expression across different cell subpopulations. Cell types are listed on the *y*-axis, and features on the *x*-axis. Dot size indicates the percentage of cells expressing the feature, and color intensity reflects the average expression level. **B** Heatmap showing gene expression levels across cell types for selected genes, with rows representing genes and columns representing cell types. Red indicates high expression, and purple indicates low expression. **C** Bar plot depicting cell-type distribution across sample conditions: TB, LTBI, and HC. Bars represent the proportion of each cell type in each condition. **D** Bar plot of cell-type distribution in samples from SRR11038995 and SRR11038994 data sets, with color-coded bars representing relative cell-type proportions. **E** Bar plot showing cell-type distribution in SRR11038993, with bars representing relative cell-type proportions. **F** Bar plot showing cell-type distribution in SRR11038989, with bars representing relative cell-type proportions

To further characterize the immune landscape, we employed a suite of advanced visualization techniques, including dot plots, heatmaps, and stacked bar charts, to meticulously illustrate the distribution patterns and gene expression signatures of immune subpopulations (monocytes/macrophages, B cells, T cells, and NK cells) across various clusters and samples. The dot plot and heatmap analyses revealed distinct differential expression patterns of pivotal functional genes among cell populations. Specifically, T cells exhibited pronounced expression of LEF1, while monocytes/macrophages demonstrated marked upregulation of FCGR3A (Fig. 3A, B).

Figure 3C delineates the comparative immune cell composition and abundance profiles among TB, LTBI, and HC groups. Notably, TB patients displayed a significant expansion in the monocyte/macrophage compartment concomitant with a reduction in T and NK cell populations, potentially reflecting chronic inflammatory responses and immune suppression mechanisms. Conversely, LTBI patients exhibited an intermediate immune cell composition between healthy individuals and TB patients, suggesting a state of balanced immune regulation. Figure 3D–F provides refined spatial distribution patterns of different cell types across samples, highlighting substantial interindividual variability and offering crucial insights into the compositional dynamics of immune microenvironments under both healthy and diseased states (Fig. 3C–F).

### Functional states and dynamic changes of T-cell subpopulations in TB

To elucidate the functional heterogeneity of T-cell subpopulations in tuberculosis (TB), we isolated T cells and performed comprehensive classification based on key marker gene expression profiles. Specifically, we identified multiple functionally distinct T-cell subsets: CD8 + cytotoxic T cells characterized by elevated expression of CD8A and CTSW; activated/inflammatory CD8 + T cells expressing inflammatory mediators, such as S100A8 and S100A9; and CD4 + naïve T cells marked by KLF2 and KLF3 expression.

**Fig. 4:**
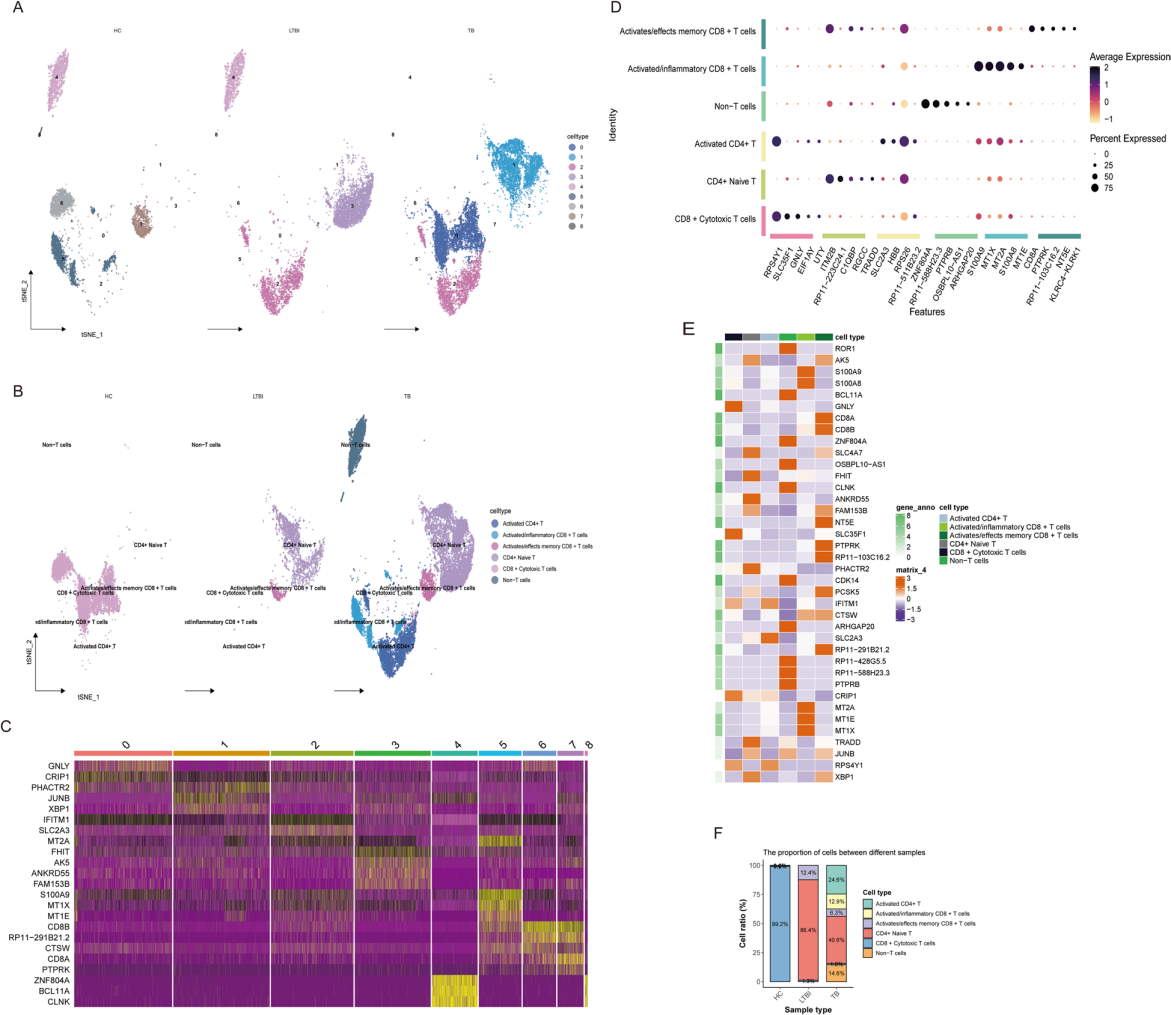
Differentiation and expression profiling of T-cell subtypes in HC, LTBI, and TB samples. **A** t-SNE projection of all samples (HC, LTBI, TB) colored by identified T-cell subtypes. Each dot represents a single cell, colored by its assigned subtype. **B** Annotated t-SNE plot labeling specific T-cell subtypes, such as CD8 + naïve T cells, activated CD8 + T cells, and activated/inflammatory subsets across sample groups. **C** Heatmap showing gene expression profiles across T-cell subtypes. Each column represents a cluster/subtype, each row a gene. Color indicates expression intensity. **D** Dot plot of selected feature genes across major T-cell subtypes. Dot size represents the percentage of cells expressing the gene; color indicates average expression. **E** Heatmap highlighting differentially expressed genes between T-cell subtypes. **F** Bar plot showing the proportion of each T-cell subtype across the three sample groups: HC, LTBI, and TB

Figure 4A, B illustrates the spatial distribution and proportional representation of T-cell subsets through t-SNE dimensionality reduction and quantitative cell proportion analysis. These visualizations reveal a substantial expansion of inflammatory T cells in TB samples, contrasting with the predominant resting/naïve T-cell population in healthy controls (HC). The differential distribution patterns across subgroups further corroborate these findings. Moreover, Fig. 4C–F, employing dot plots and heatmaps, delineates the distinct gene expression signatures of various T-cell subpopulations. TB samples exhibit marked upregulation of inflammation-associated genes and an increased prevalence of inflammatory T cells, while HC samples demonstrate elevated expression of resting/naïve T-cell markers.

These findings uncover dynamic alterations in T-cell subpopulation dynamics during TB infection, characterized by a pronounced expansion of activated/inflammatory T cells concomitant with a reduction in naïve T-cell populations. This suggests potential dysregulation of T-cell-mediated immunity, leading to chronic inflammatory responses and immune imbalance in TB patients. Notably, latent TB infection (LTBI) patients display intermediate T-cell characteristics between HC and TB groups, indicative of a delicate immune regulatory equilibrium during the latent phase.

### Cell communication analysis

Cell communication analysis offers critical insights into intercellular signaling networks mediated through ligand–receptor interactions, elucidating fundamental biological processes including immune regulation, cellular metabolism, and microenvironmental homeostasis. This approach reveals regulatory layers often obscured in conventional gene expression analyses, particularly regarding cytokine, chemokine, and immune checkpoint molecule expression patterns within specific subpopulations.

**Fig. 5:**
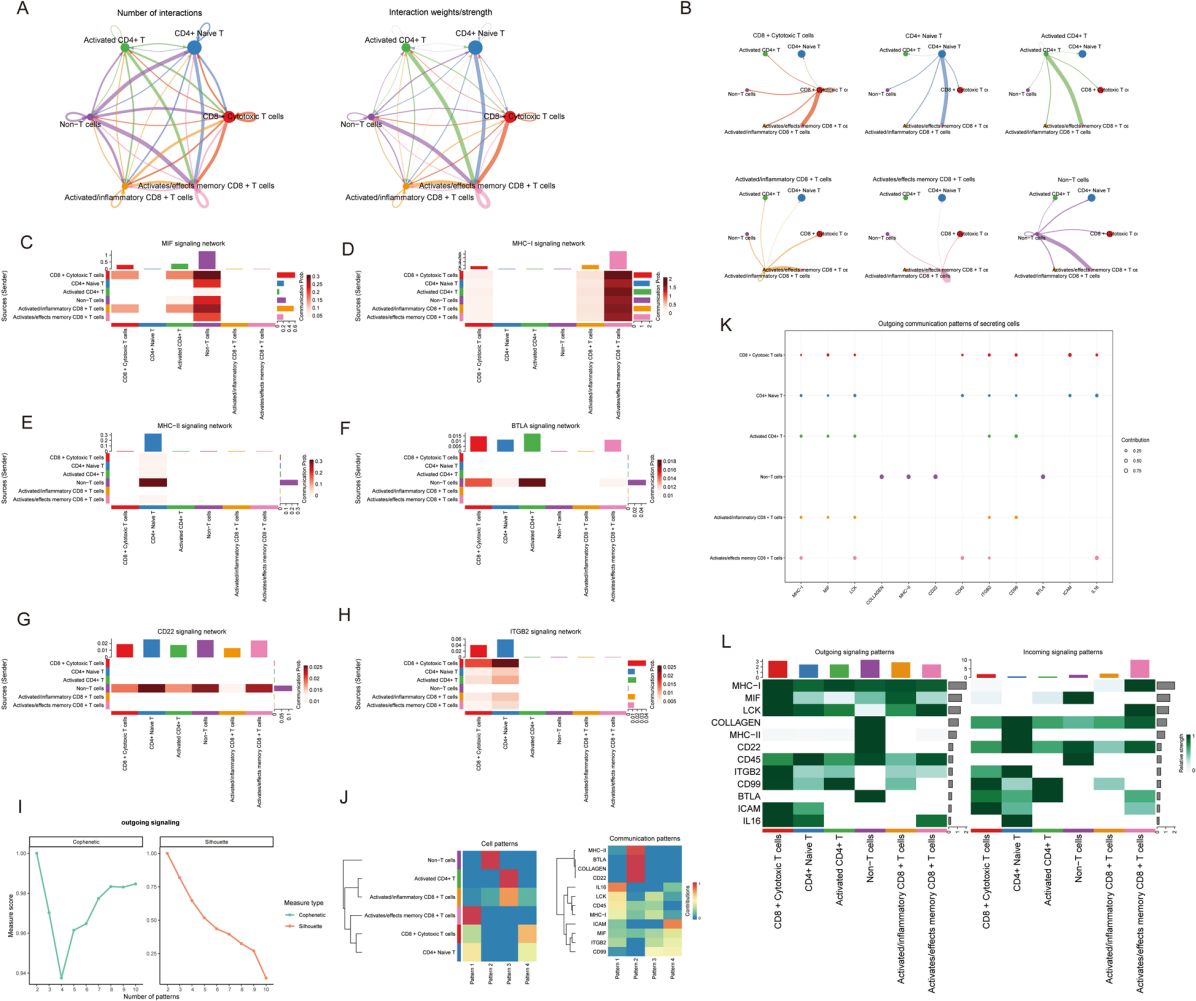
Cell–cell communication network analysis among T-cell subtypes. **A** Circle plots displaying the number (left) and strength (right) of interactions among T-cell subtypes. **B** Chord diagrams showing detailed outgoing communication from each T-cell subtype to others. **C–H** Heatmaps of signaling networks for individual pathways including MIF (**C**), MHC-I (**D**), MHC-II (**E**), BTLA (**F**), CCL22 (**G**), and ITGB2 (**H**). Rows are source cell types and columns are target cell types; color intensity reflects communication probability. **I** Line plots showing the number of outgoing signaling patterns against communication centrality (left: contribution; right: structural centrality). **J** Heatmaps illustrating clustering of outgoing signaling patterns (left) and communication pattern similarity between ligands/receptors and cell types (right). **K** Dot plot showing the outgoing communication patterns of each secreting T-cell subtype. **L** Heatmaps of outgoing (left) and incoming (right) signaling contributions for each T-cell subtype across various signaling pathways

Our investigation identified extensive intercellular communication networks among T-cell subpopulations, with CD8 + cytotoxic T cells demonstrating particularly robust interaction frequencies, underscoring their pivotal role in TB pathogenesis. The extensive and intense interactions of CD8 + cytotoxic T cells suggest their dual role in both immune clearance and modulation of other immune cell functions to maintain immunological balance (Fig. 5A).

Figure 5B provides a detailed subgroup-level analysis of these interactions, revealing that communication between activated memory CD4 + T cells and activated CD8 + T cells is primarily mediated through chemokine and cytokine signaling pathways. Figure 5C–H presents heatmap visualizations of interactions across various signaling families, including MIF, MHC-I, MHC-II, BTLA, CD22, and ITGB2, highlighting the complexity and multilayered nature of T-cell-mediated immune communication networks.

CD8 + cytotoxic T cells emerge as central players in multiple signaling networks, reinforcing their critical position in anti-TB immune responses (SF1G-1L). Activated CD4 + T cells contribute significantly to immune response regulation, supporting both initiation and maintenance of immune reactions. Notably, CD8 + cytotoxic T cells serve as primary signal transducers in key pathways, particularly MHC-I and BTLA signaling, suggesting their involvement in immune surveillance, clearance, and regulation. Meanwhile, CD4 + T cells predominantly regulate humoral immunity through MHC-II signaling pathways (SF1A–1F).

Further analysis of outward signaling patterns, cellular communication modalities, and signaling pathways (Fig. 5I, J) reveals the functional specialization of immune cells across different communication modes and key signaling networks. These cellular patterns not only reflect the distinct functional roles of various cell types in immune responses but also highlight the central regulatory functions of specific molecules (e.g., MHC-II and MIF) in immune modulation.

Figure 5K, L further illustrates the diverse functional roles of immune cells in outward and inward signaling. CD8 + cytotoxic T cells emerge as key effector cells, primarily through MHC-I-mediated cytotoxic functions while also participating in immune regulation via MIF, LCK, and BTLA signaling pathways. Conversely, CD4 + activated T cells and non-T cells are more involved in immune modulation and support through pathways, such as MHC-II and COLLAGEN, playing crucial roles in immune tolerance and regulation.

### Acquisition of metabolic differences

**Fig. 6:**
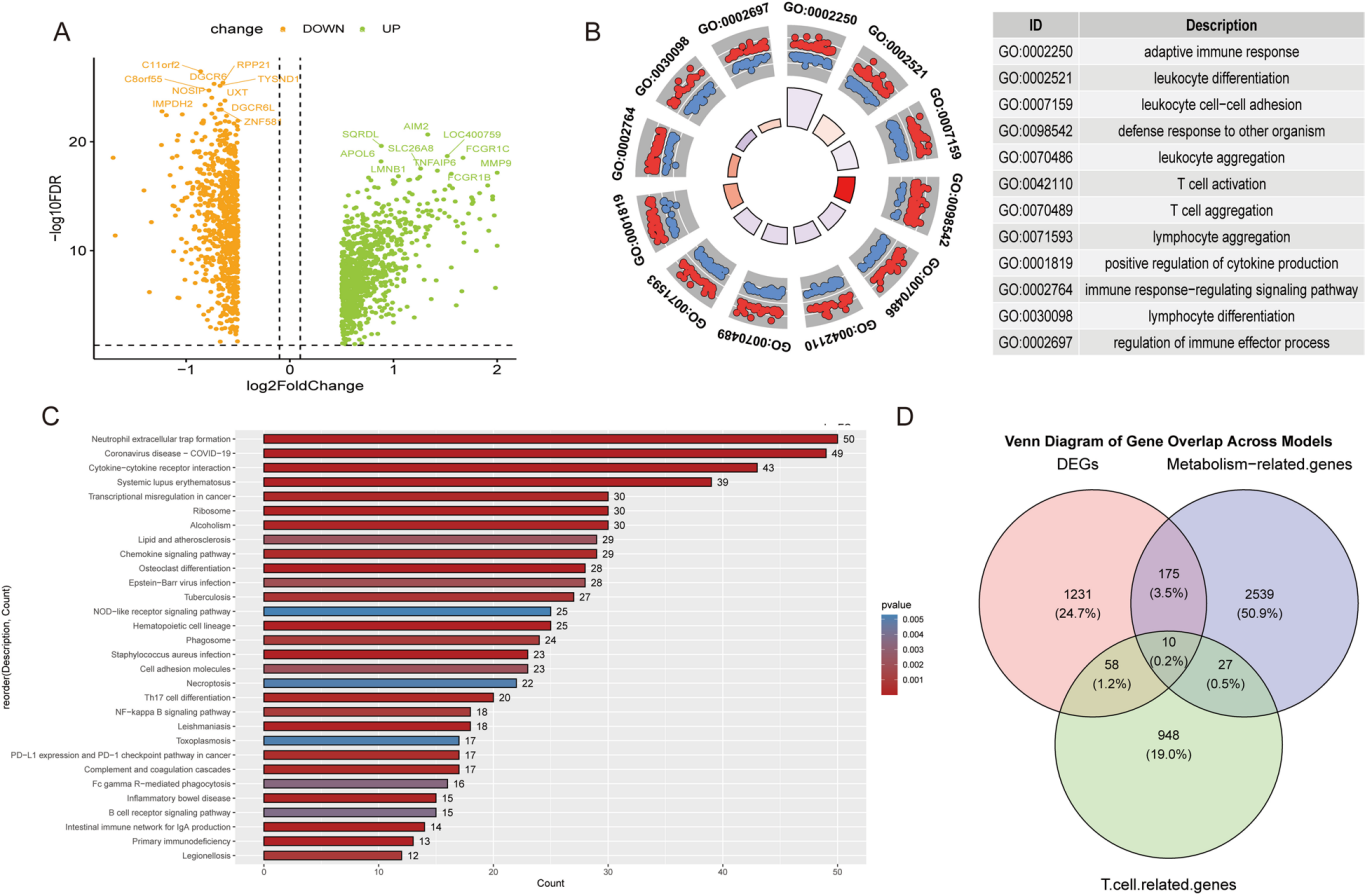
Metabolic differences analysis. **A** Volcano plot showing differentially expressed genes (DEGs) between two conditions. **B** Circular plot of enriched Gene Ontology (GO) biological process terms among DEGs. **C** KEGG pathway enrichment bar chart. **D** Venn diagram showing overlap among different gene sets: DEGs (red), metabolism-related genes (blue), and T-cell-related genes (green). Overlapping areas represent genes shared by two or more sets, with counts and percentages annotated

Building upon the aforementioned findings, we conducted a comprehensive analysis to identify potential biomarkers for distinguishing active tuberculosis (TB) from latent tuberculosis infection (LTBI). We screened TB and LTBI data sets from the GEO database to achieve this objective. The volcano plot (Fig. 6A) illustrates the significantly upregulated and downregulated gene expression profiles in both sample groups. Detailed information on the differential genes can be found in Supplementary Materials 2. Through Gene Ontology (GO) enrichment analysis, we confirmed that these differentially expressed genes are predominantly involved in adaptive immune responses and T-cell activation, underscoring the pivotal role of the immune system in disease pathogenesis (Fig. 6B). Concurrently, KEGG pathway analysis revealed several critical signaling pathways, including NOD-like receptor signaling, PD-1 checkpoints, and NF-κB signaling pathways related to immune regulation, as well as pathways associated with infectious diseases (e.g., COVID-19, tuberculosis, and *Staphylococcus aureus* infection) and inflammatory responses. These findings highlight the significance of these biological processes in immune regulation (Fig. 6C). Furthermore, we employed a Venn diagram to analyze the intersection of differentially expressed genes, T-cell-related genes, and metabolism-related genes, ultimately identifying ten candidate genes (Fig. 6D) as potential biomarkers for TB and LTBI diagnosis.

### Machine learning-based feature gene selection

**Fig. 7:**
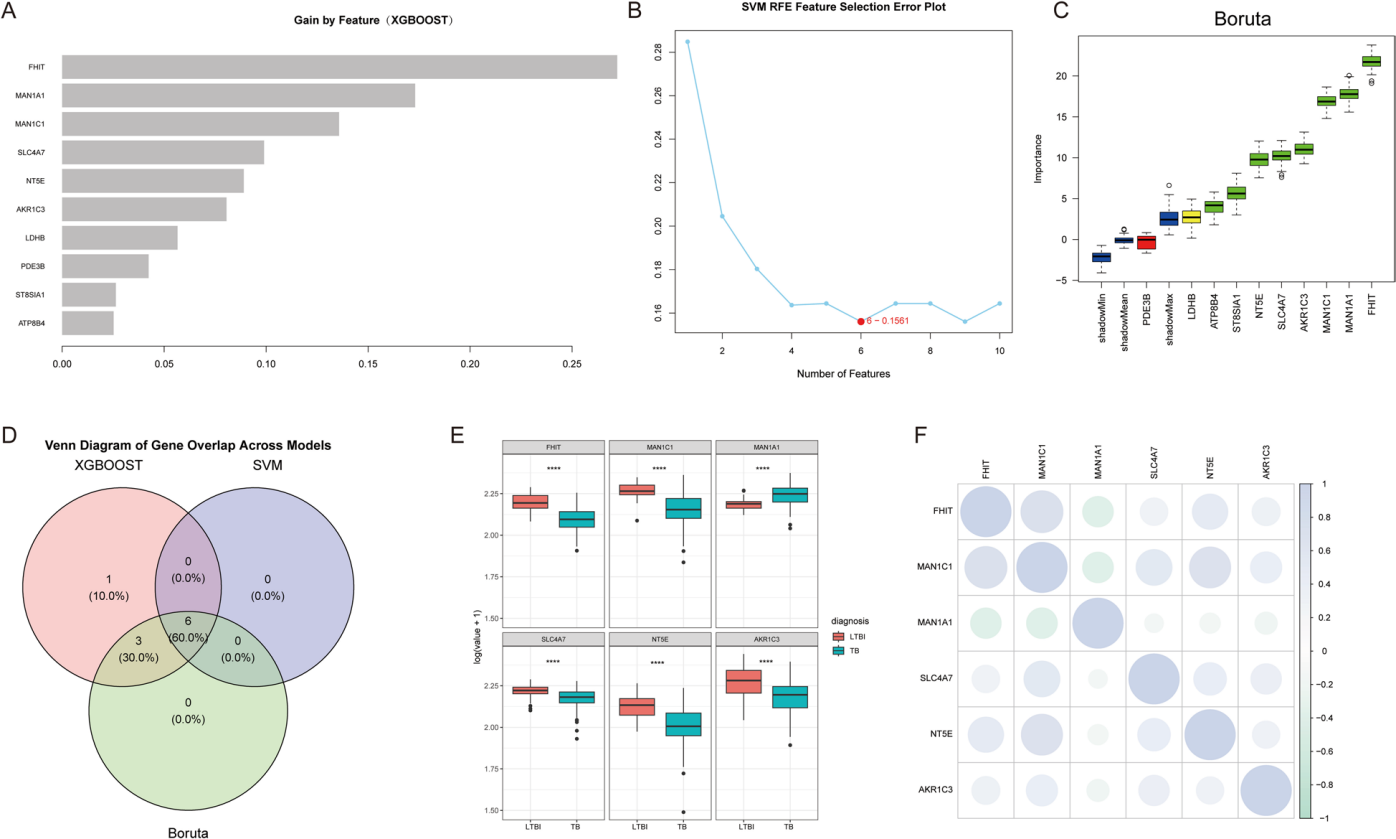
Machine learning-based screening of differentially expressed genes. **A** Bar plot of feature importance from the XGBoost model. The *x*-axis shows gain values, representing the relative importance of each gene. **B** SVM–RFE (support vector machine–recursive feature elimination) error plot. The lowest point indicates the optimal number of features with minimal error (6 features). **C** Boxplot from the Boruta algorithm showing the importance scores of genes. Higher scores indicate greater importance in classification. **D** Venn diagram showing overlap of selected genes among the three models (XGBOOST, SVM, and Boruta). **E** Boxplots comparing expression levels (log-transformed) of top genes between LTBI (latent TB infection) and TB (active tuberculosis) groups. **F** Correlation matrix of selected genes. The size and color of each circle represent the strength and direction of correlation

To refine the selection of TCM–DEGs, we applied three machine learning algorithms for feature selection (Fig. 7A–C). Figure 7D presents the intersection and union of features selected by XGBoost, SVM–RFE, and Boruta through a Venn diagram, with numerical representations indicating the number of features selected by each model either jointly or independently. This approach facilitated the identification of the most consistent and stable potential biomarkers. We identified six TCM–DEGs (FHIT, MAN1C1, MAN1A1, SLC4A7, NT5E, AKR1C3) that demonstrated significant differential expression between the TB and LTBI groups (*p* < 0.05) in the box plot analysis (Fig. 7E). Similar results were observed in the GSE39939 data set (SF2). The correlation heatmap (Fig. 7F) illustrates the positive and negative correlations between features, suggesting potential functional relationships in biological pathways.

**Fig. 8:**
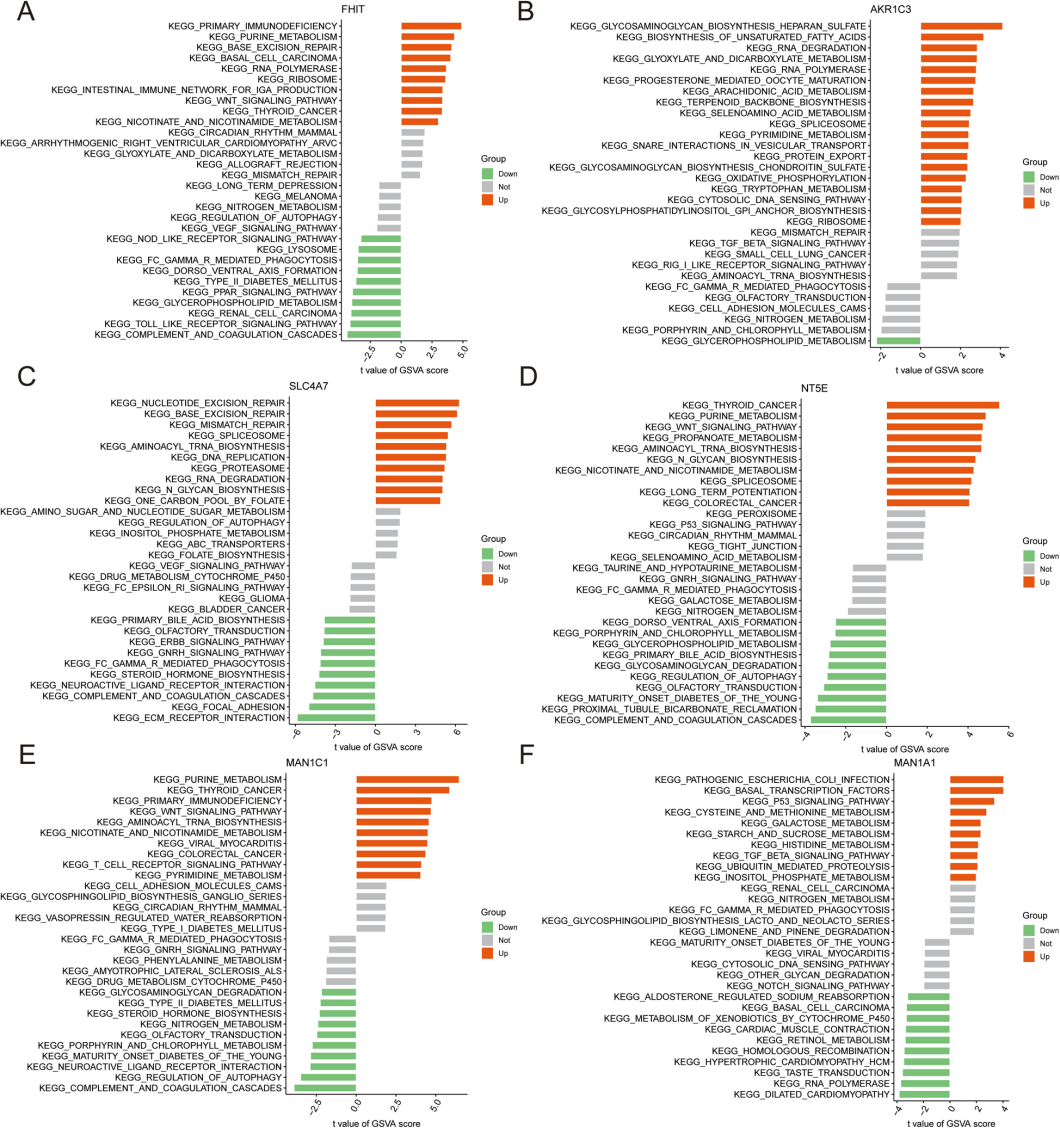
GSVA enrichment analysis of hub genes. **A–F** GSVA-based KEGG pathway enrichment analysis for six key differential genes: FHIT (**A**), AKR1C3 (**B**), SLC4A7 (**C**), NT5E (**D**), MANT1C1 (**E**), and MAN1A1 (**F**). Each panel shows a bar plot of enriched KEGG pathways for high and low expression levels of the indicated gene. Pathways significantly enriched in the high-expression group are shown in orange, those enriched in the low-expression group in green, and non-significant pathways in gray. The *x*-axis represents the *t* value of the GSVA score

Subsequently, we conducted GSVA enrichment analysis to elucidate the biological functions of the six TCM–DEGs. These genes are primarily associated with cytokine signaling, T-cell receptor signaling, glucose metabolism, lipid metabolism, and toll-like receptor signaling pathways. These findings suggest that these genes and their associated pathways provide robust support for potential biomarkers, warranting further investigation for their application in TB diagnosis and treatment (Fig. 8A–F).

### Machine learning to build a diagnostic model

**Fig. 9:**
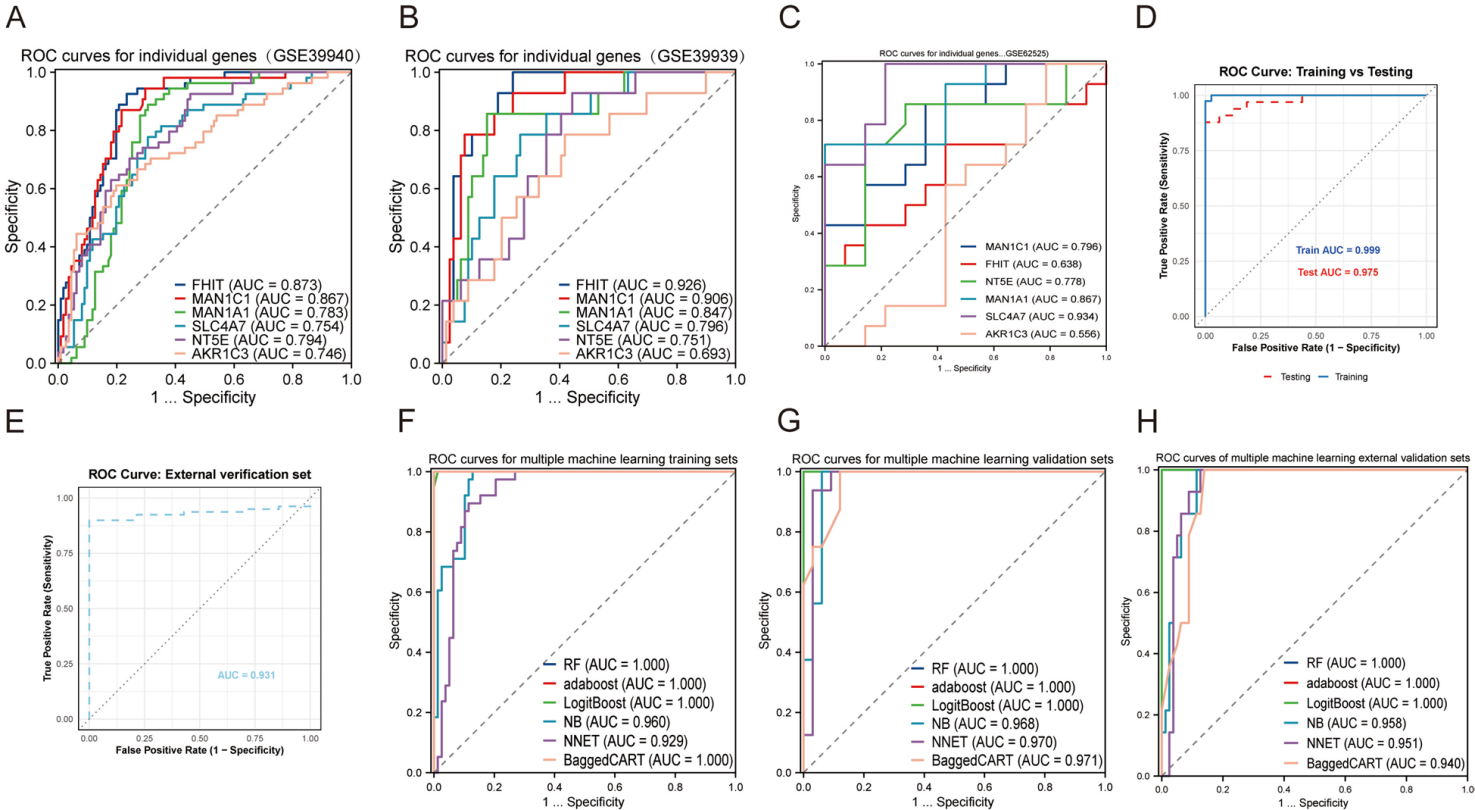
Construction of machine learning diagnostic models. **A–C** ROC curves of six key genes (FHIT, MAN1C1, MAN1A1, SLC4A7, NT5E, and AKR1C3) in three independent data sets: GSE39940 (**A**), GSE39939 (**B**), and GSE52525 (**C**). AUC values are listed for each gene. **D** ROC curve for the XGBoost model, comparing performance on the training set (AUC = 0.999) and testing set (AUC = 0.975). **E** ROC curve of the XGBoost model applied to an external validation set. **F–H** ROC curves for six machine learning algorithms on training sets (**F**), validation sets (**G**), and external validation sets (**H**)

The machine learning-based diagnostic model, incorporating multiple genes including FHIT, MAN1C1, MAN1A1, SLC4A7, NT5E, and AKR1C3, demonstrated exceptional predictive performance. Initial single-gene diagnostic analysis revealed that all six genes achieved AUC values exceeding 0.7 in the GSE39940 data set, with FHIT (AUC = 0.873) and MAN1C1 (AUC = 0.867) exhibiting particularly robust diagnostic capabilities, suggesting their potential as key biomarkers in disease identification (Fig. 9A). Consistent high predictive performance was observed across independent validation data sets (GSE39939 and GSE28623), as illustrated in Fig. 9B, C, confirming both the stability of individual gene markers and the reliability of our findings.

The multi-gene machine learning model developed subsequently demonstrated exceptional diagnostic accuracy and robust generalization ability across the training, internal validation, and external validation data sets (Fig. 9D–H and Supplementary Table 3). These results underscore the substantial potential for clinical implementation in early disease screening and diagnostic applications. Notably, FHIT, MAN1C1, and SLC4A7 consistently demonstrated high predictive performance across all data sets, further validating their status as stable and reliable biomarkers. However, while these preliminary findings are promising, additional validation through large-scale, multi-center clinical trials is critical for assessing the model's applicability across diverse populations and identifying potential limitations.

### Development and validation of clinical prediction models

**Fig. 10:**
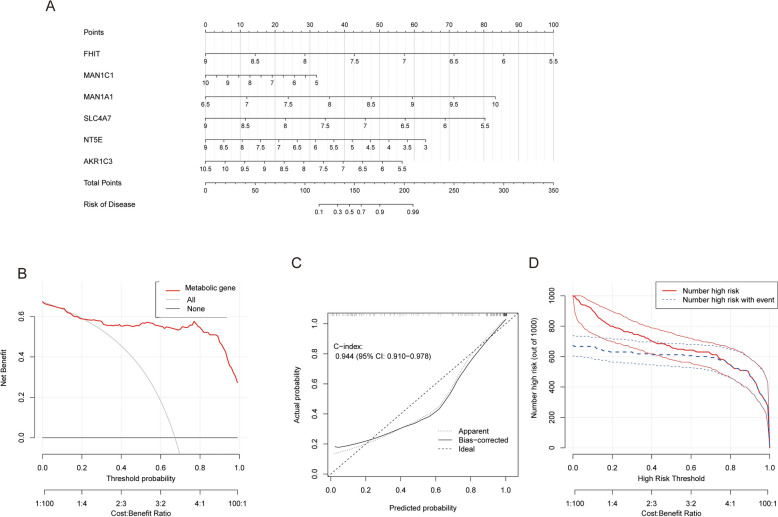
Clinical prediction model based on TCM–DEGs. **A** Nomogram integrating six metabolism-related genes (FHIT, MAN1C1, MAN1A1, SLC4A7, NT5E, and AKR1C3) to predict individual disease risk. Each gene contributes a score, which sums to a total score that translates into a predicted probability of disease. **B** Decision curve analysis (DCA) evaluating the net clinical benefit of the metabolic gene model across different threshold probabilities. The red curve (metabolic gene) shows a higher net benefit than the"All"and"None"strategies. **C** Calibration plot of the predictive model. The dashed line represents apparent accuracy, the solid line is the bias-corrected performance, and the diagonal line represents ideal prediction. C-index = 0.944 (95% CI 0.910–0.978). **D** Clinical impact curve showing the number of high-risk individuals identified (red line) and the number of true positives (blue dashed line) at different high-risk thresholds

To bridge the gap between machine learning predictions and clinical applicability, we constructed a comprehensive nomogram model integrating six TCM–DEGs for tuberculosis (TB) risk prediction (Fig. 10A). The model underwent rigorous validation through calibration curves, decision curve analysis (DCA), and clinical impact curve assessment. The calibration curve exhibited exceptional concordance between predicted and observed outcomes, achieving a C-index of 0.944 (Fig. 10C). DCA results demonstrated substantial net benefit across a broad spectrum of threshold probabilities, affirming the model's clinical utility (Fig. 10B). Furthermore, the clinical impact curve corroborated the model's robust predictive performance (Fig. 10D), underscoring its potential as a valuable decision-support tool in clinical practice.

### Association between TCM–DEGs and immune infiltration

**Fig. 11:**
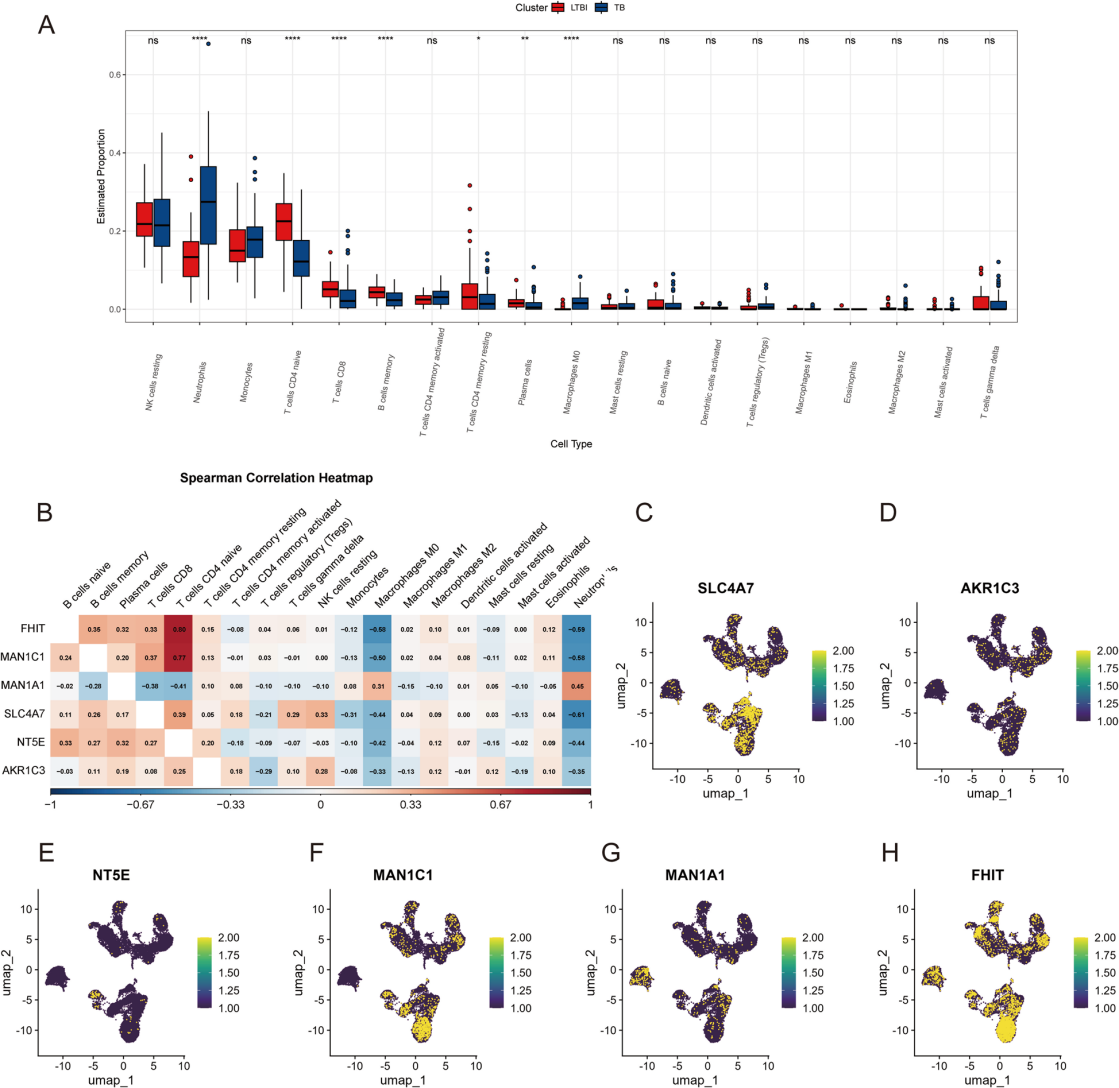
Association of metabolism-related key genes with immune cell subsets. **A** Boxplots comparing the proportions of immune cell subsets between latent tuberculosis infection (LTBI, red) and active tuberculosis (TB, blue) groups using CIBERSORTx deconvolution. Significant differences are indicated with asterisks (*p < 0.05; **p < 0.01; ***p < 0.001). **B** Spearman correlation heatmap between the expression levels of six metabolism-related genes (FHIT, MAN1C1, MAN1A1, SLC4A7, NT5E, and AKR1C3) and proportions of immune cell subsets. Positive correlations are shown in red, and negative correlations in blue. **C–H** UMAP visualization of single-cell transcriptomes showing expression distributions of the six key metabolism-related genes across immune cells, with expression levels indicated by color intensity

We subsequently investigated the relationship between TCM–DEGs and immune cell infiltration patterns. CIBERSORT analysis revealed a significant increase in M0 macrophages and neutrophils within the TB group, suggesting that M0 macrophages had not yet differentiated into M1 (pro-inflammatory) or M2 (anti-inflammatory) phenotypes, indicating an early or dysregulated immune response to Mycobacterium tuberculosis (Mtb) infection. In contrast, the LTBI group exhibited elevated levels of CD4 + and CD8 + T cells, suggesting a more effective immune containment mechanism that potentially restricts Mtb within granulomas and prevents systemic dissemination (Fig. 11A).

Furthermore, we employed Spearman correlation analysis to assess the relationship between metabolism-related genes (FHIT, MAN1C1, MANA1A, SLC4A7, NT5E, and AKR1C3) and immune cell infiltration levels. The results demonstrated significant correlations between gene expression patterns and specific immune cell infiltration profiles, suggesting these genes may play regulatory roles in shaping the immune microenvironment (Fig. 11B–H).

### Identification of TCM–DEGs-related subpopulations in TB

**Fig. 12:**
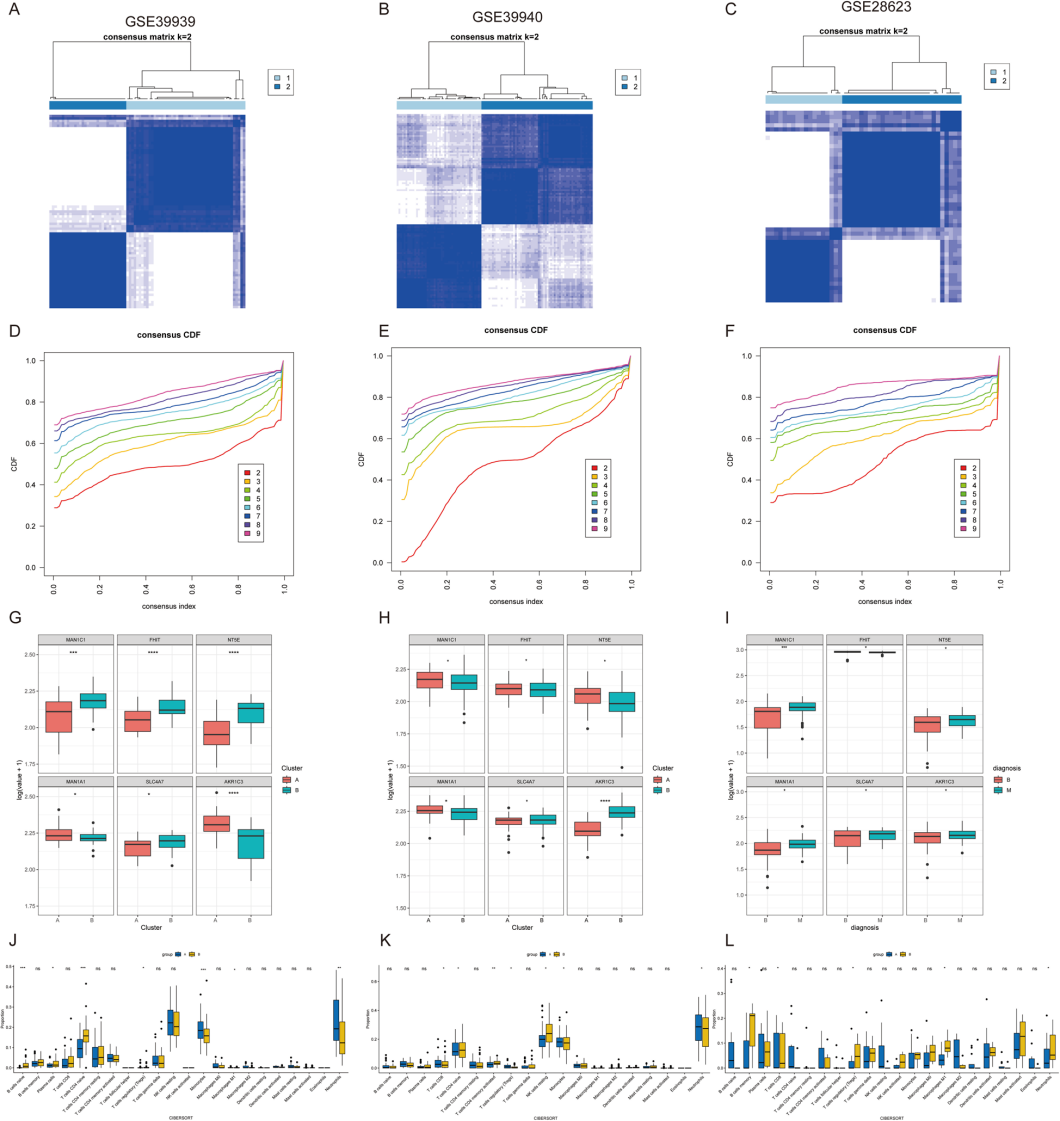
Identification of TCM–DEGs-related subpopulations in TB. **A–C** Consensus clustering heatmaps (*k* = 2) based on the expression profiles of metabolism-related genes across three independent GEO data sets: GSE39939 (**A**), GSE39940 (**B**), and GSE28623 (**C**). Blue blocks represent higher consensus within clusters. **D–F** Cumulative distribution function (CDF) plots showing the consensus index distributions for cluster numbers (*k* = 2–9) in GSE39939 (**D**), GSE39940 (**E**), and GSE28623 (**F**). Flatter CDF curves indicate more stable clustering. **G–I** Boxplots showing expression differences of the six key metabolism-related genes between identified clusters in GSE39939 (**G**), GSE39940 (**H**), and GSE28623 (**I**). **J–L** Immune cell infiltration analysis using CIBERSORTx algorithm for each data set: GSE39939 (**J**), GSE39940 (**K**), and GSE28623 (**L**). Boxplots show proportions of 22 immune cell types across clusters

Building upon these immune infiltration patterns, we explored the molecular classification of TB based on TCM–DEGs expression profiles. Utilizing Consensus Clustering and cumulative distribution function (CDF) analysis of 79 TB samples based on six core gene expressions, we identified k = 2 as the optimal classification, revealing two distinct molecular subtypes of the disease (Fig. 12A). This classification demonstrated consistent reproducibility across multiple independent data sets (Fig. 12B, C), with CDF analysis further supporting the robustness and biological significance of these subtypes (Fig. 12D–F).

Expression analysis of key metabolic genes (MAN1C1, FHIT, SLC4A7, AKR1C3) revealed significant differential expression patterns across subtypes, suggesting their potential influence on disease molecular typing and pathophysiological processes (Fig. 12G–I). Immune cell infiltration analysis further demonstrated substantial heterogeneity in the immune microenvironment across subtypes. One subtype was characterized by M2 macrophage enrichment, indicative of an immunosuppressive state, while another subtype exhibited elevated CD8 + T-cell infiltration, potentially indicating greater sensitivity to immunotherapeutic interventions (Fig. 12J–L). These findings not only elucidate the critical role of metabolic genes in disease subtype classification but also provide novel insights for developing targeted immunotherapy strategies in tuberculosis management.

### Comparative pathological analysis across disease states

**Fig. 13:**
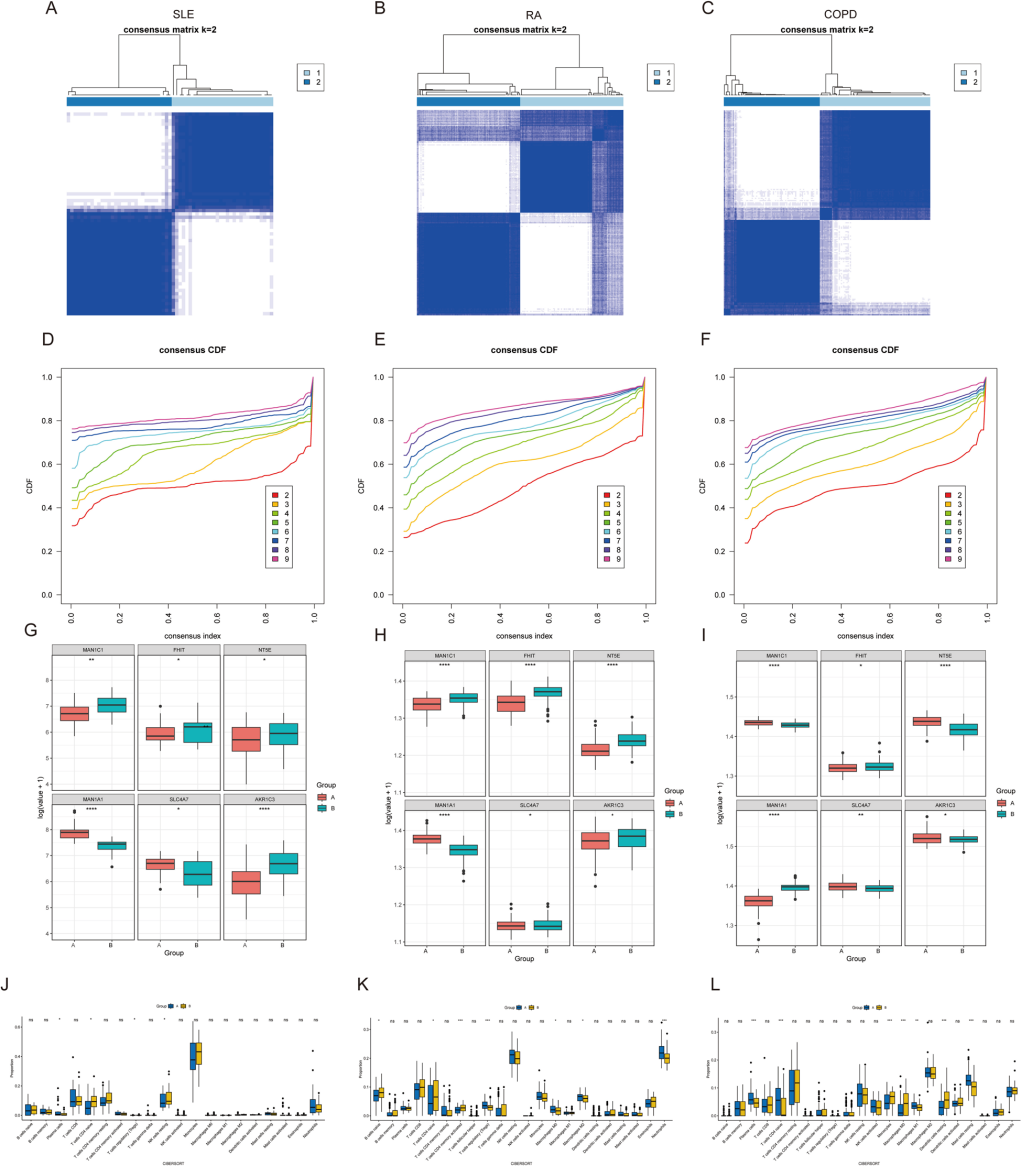
Identification of TCM–DEGs-related subpopulations in other diseases. **A–C** Consensus clustering heatmaps for systemic lupus erythematosus (SLE, **A**), rheumatoid arthritis (RA, **B**), and chronic obstructive pulmonary disease (COPD, **C**) based on immune cell subsets. The consensus matrix (*k* = 2) indicates the degree of consistency within clusters. Dark blue represents high consistency, while white areas represent low consistency. **D–F** Cumulative distribution function (CDF) plots for the consensus index across various consensus numbers (*k* = 2–6) for SLE (**D**), RA (**E**), and COPD (**F**). **G–I** Boxplots showing the expression differences of key immune genes (MAN1C1, MAN1A1, SLC4A7, NT5E, and AKR1C3) between identified clusters in SLE (**G**), RA (**H**), and COPD (**I**). **J–L** Boxplots comparing the proportions of 22 immune cell types between clusters in SLE (**J**), RA (**K**), and COPD (**L**)

The pathogenesis of tuberculosis (TB) exhibits substantial parallels with various other diseases, including rheumatoid arthritis (RA), chronic obstructive pulmonary disease (COPD) (n = 44), and systemic lupus erythematosus (SLE) (n = 51). Consequently, we conducted a subgroup analysis based on the previously established TCM–DEGs correlation features, which revealed that at k = 2, all disease cohorts except RA demonstrated clear bifurcation into two distinct groups (Fig. 13A–F). Notably, comparative analysis of these disease subgroups revealed significant differential expression patterns of TCM–DEGs across most patient subgroups (Fig. 13G–I).

To further elucidate the immune landscape, we employed the CIBERSORT algorithm to quantify the enrichment profiles of 22 immune cell types across these disease states. Comparative analysis between subgroups A and B revealed significant differences in immune cell enrichment patterns. Specifically, among the five disease cohorts, subgroup A exhibited marked variations in the abundance of plasma cells, CD4 + T cells, regulatory T cells, macrophages, and neutrophils relative to subgroup B, while NK cell enrichment remained comparable across groups (Fig. 13J–L). These findings substantiate the broader applicability of the TCM–DEGs-associated genetic signature across diverse pathological contexts.

### Clinical validation of TCM–DEGs expression

**Fig. 14:**
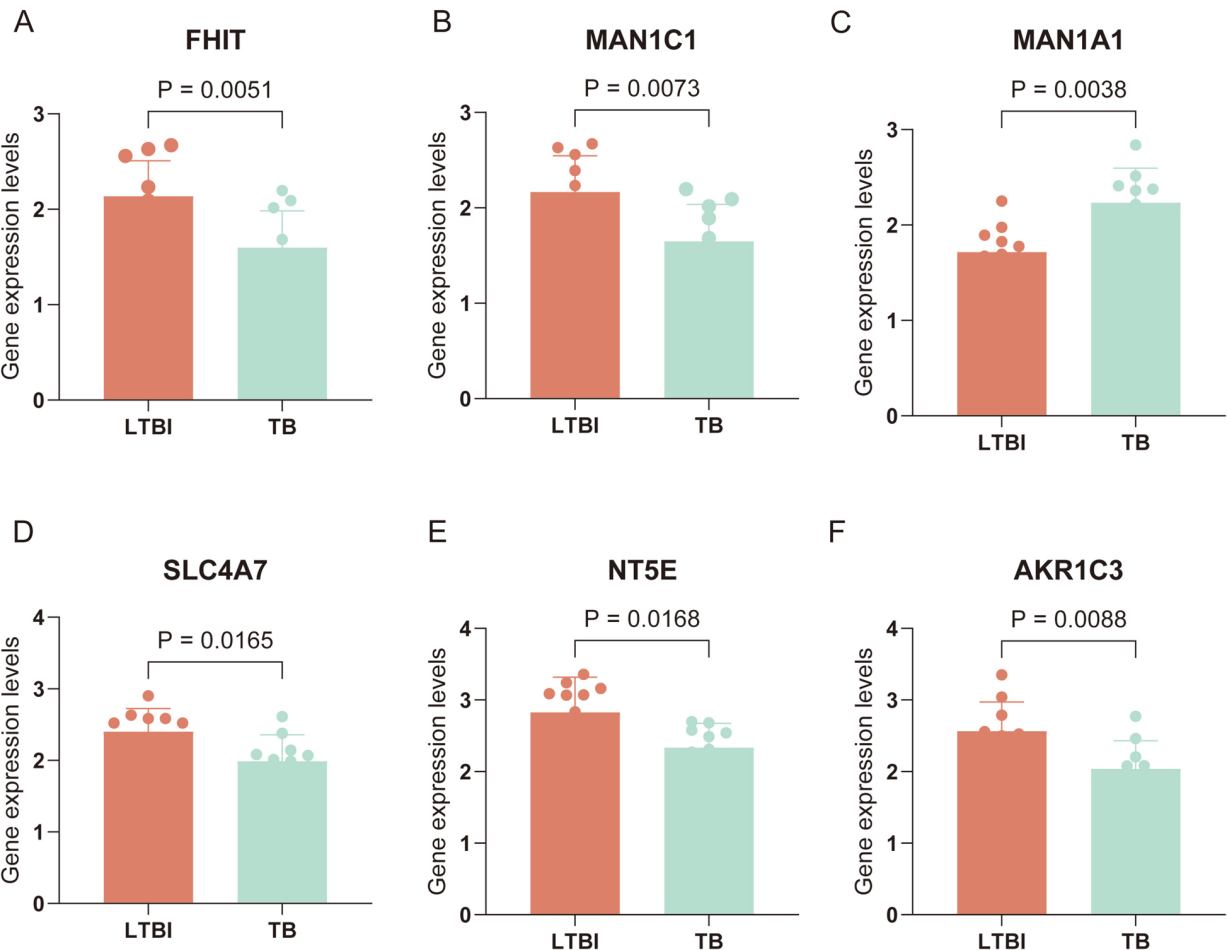
RT-qPCR validation of clinical samples. **A–F** Bar plots showing the expression levels of six key metabolism-related genes in patients with latent tuberculosis infection (LTBI) and active tuberculosis (TB). **A** FHIT expression was significantly decreased in TB compared to LTBI (*p* = 0.0051). **B** MAN1C1 expression was significantly lower in TB (*p* = 0.0073). **C** MAN1A1 expression was significantly increased in TB (*p* = 0.0038). **D** SLC4A7 expression was significantly reduced in TB (*p* = 0.0165). **E** NT5E expression was significantly lower in TB (*p* = 0.0168). **F** AKR1C3 expression was significantly decreased in TB (*p* = 0.0088). Bars represent mean ± SEM; significance was determined using unpaired *t* tests

To validate the expression profiles of the six core genes, we performed RT-qPCR analysis on clinical samples from TB patients (*n* = 10) and LTBI patients (*n* = 10). The results demonstrated significant upregulation of these genes in TB patients, with the exception of MAN1A1, which exhibited elevated expression in LTBI samples. These experimental findings corroborate the results obtained from our bioinformatics analysis, thereby reinforcing the reliability of our identified biomarkers (Fig. 14A–F).

## Discussion

Active tuberculosis (ATB) represents a formidable global health challenge, underscoring the critical need for innovative diagnostic approaches and therapeutic strategies[45]. The intricate immunological mechanisms governing the transition from latent tuberculosis infection (LTBI) to active tuberculosis (ATB) necessitate a comprehensive understanding of transcriptional dynamics. Such an understanding is crucial for elucidating their functional implications in TB pathogenesis. Through single-cell RNA sequencing (scRNA-seq) analysis, we identified distinct immunological profiles, with TB patients demonstrating a reduced proportion of T cells and NK cells, coupled with a significant elevation in the monocyte/macrophage ratio compared to LTBI patients. This immunological dysregulation underscores the systemic impact of Mycobacterium tuberculosis (Mtb) infection. Our findings corroborate those of Rong et al., who similarly observed an increased monocyte/macrophage ratio in TB patients, highlighting their pivotal role in TB pathogenesis [23]. Existing literature demonstrates that Mtb infection induces the secretion of chemokines, including CCL2 and CXCL10. These chemokines facilitate the recruitment of monocytes to infection sites, where they differentiate into macrophages and form granulomas to contain bacterial dissemination [24, 25]. However, excessive macrophage activation may precipitate the release of pro-inflammatory cytokines (e.g., TNF-α and IL-1β), resulting in tissue damage and cavitation [26]. Furthermore, chronic Mtb infection induces the upregulation of inhibitory receptors (e.g., PD-1 and TIM-3) on T cells, leading to impaired proliferation and diminished IFN-γ production [27]. Our results align with these observations, and Mardi et al. similarly documented an increased exhausted T-cell phenotype in TB patients, emphasizing the critical role of inhibitory receptors in Mtb immune evasion [28].

In this study, we systematically analyzed the dynamics of T-cell subpopulations and their intercellular communication patterns during Mycobacterium tuberculosis (Mtb) infection. By applying t-distributed stochastic neighbor embedding (t-SNE) for dimensionality reduction and subsequent cell-proportion analysis, we observed a marked increase in inflammatory T-cell subsets in TB patients, whereas healthy controls (HC) predominantly exhibited resting or naïve T cells. Patients with latent tuberculosis infection (LTBI) displayed an intermediate T-cell phenotype, suggesting that the host immune system maintains a dynamic equilibrium during the latent phase. These observations are consistent with those of Jacob et al., who similarly reported elevated inflammatory T-cell populations in TB patients, thereby reinforcing the pivotal role of these cells in TB pathogenesis. However, whereas Jacob et al. primarily examined alterations in cytokine levels, our analysis further elucidates T-cell contributions to immune responses by characterizing dynamic changes within T-cell subpopulations [29]. Gene expression profiling revealed a significant upregulation of inflammation-associated genes (e.g., IL-6 and TNF-α) in TB samples, whereas HC samples exhibited higher expression of naïve T-cell markers (e.g., CCR7 and LEF1), indicating that TB infection promotes T-cell activation and amplifies immune responses.

Furthermore, intercellular communication analysis among T-cell subpopulations demonstrated that CD8 + cytotoxic T cells assume a dominant role during TB infection. Their frequent cellular interactions and robust signal transduction capabilities underscore their central role in mediating immune clearance and regulation. Further analyses indicated that CD8 + cytotoxic T cells predominantly mediate immune surveillance and cytolytic activities via MHC class I and BTLA signaling pathways, whereas CD4 + T cells predominantly regulate humoral immune responses via the MHC class II pathway. Moreover, interactions between CD4 + and CD8 + T cells are orchestrated by chemokines (e.g., CXCL10 and CCL5) and cytokines (e.g., IL-2 and IFN-γ), further highlighting the complex, multi-layered immune network remodeled during TB infection. Similarly, Qian et al. documented substantial activation of chemokine and cytokine signaling pathways in TB patients, underscoring their pivotal roles in immune regulation [30]. This study not only corroborates these findings but also, through intercellular communication analyses, highlights the dual role of CD8 + T cells in immune surveillance and cytotoxicity, offering novel insights into TB immunopathogenesis. Signaling pathway analysis revealed that T-cell-mediated immune communication during TB infection involves several key molecules, including macrophage migration inhibitory factor (MIF), MHC class I, MHC class II, and BTLA. CD8 + cytotoxic T cells not only execute cytolytic functions via the MHC class I pathway but also engage in immune modulation through MIF, LCK, and BTLA signaling pathways, potentially contributing to immune overactivation and chronic inflammation. Concurrently, activated CD4 + T cells and other immune cells regulate immunity via MHC class II and collagen signaling pathways, playing essential roles in maintaining immune tolerance and homeostasis.

In concordance with previous studies, the present study observed a marked increase in the abundance of inflammatory T cells and monocytes in the peripheral blood of patients with active pulmonary tuberculosis (ATB). We identified six differentially expressed genes related to T-cell metabolism (TCM–DEGs) in individuals with latent tuberculosis infection (LTBI) and active TB. These genes were predominantly enriched in metabolic pathways (e.g., purine metabolism, amino sugar and nucleotide sugar metabolism, and arachidonic acid metabolism), as well as in immune and inflammation-associated pathways (e.g., cytokine–receptor interactions, chemokine signaling, and pathogen recognition receptor pathways), signal transduction cascades (e.g., TCR signaling, JAK–STAT, PI3K–Akt, and NF-κB pathways), and pathways governing cellular structure and interactions. Overall, these findings suggest that T cells in active pulmonary tuberculosis manifest enhanced metabolic activation and inflammatory responses, consistent with the clinical observation of elevated peripheral blood inflammatory T cells and monocytes. The concerted upregulation of metabolic, signal transduction, and inflammatory pathways may constitute a pivotal driver of ATB immunopathology, while these pathway discrepancies offer promising biomarkers and therapeutic targets for the early differentiation between LTBI and active TB, disease progression monitoring, and the development of personalized treatment strategies.

Given the persistent clinical challenge of distinguishing latent tuberculosis infection (LTBI) from active tuberculosis (ATB) using traditional diagnostics, such as the tuberculin skin test (TST) and interferon-gamma release assays (IGRA), this study employed gene expression profiling to identify key metabolic differences and evaluate metabolic genes as potential diagnostic biomarkers. We implemented an integrated bioinformatics and machine learning pipeline to extract stage-specific gene expression signatures. The resulting signature, comprising FHIT, MAN1C1, MAN1A1, SLC4A7, NT5E, and AKR1C3, delineates immune–metabolic alterations characteristic of active disease. Cross-validation with high-throughput data sets and independent clinical specimens confirmed the robust discrimination between LTBI and ATB afforded by this signature and underscored the pivotal involvement of metabolic pathways in disease progression.

The FHIT gene, a canonical tumor suppressor, encodes a critical dinucleotidase that orchestrates purine catabolism, modulates HER2 signaling, and contributes to immune evasion. Song et al. revealed that Mycobacterium tuberculosis (Mtb) infection may potentiate lung cancer progression via FHIT loss of heterozygosity (LOH) [31], a finding subsequently corroborated by Brisebarre et al., who demonstrated that FHIT abrogation hyperactivates HER2 pathways, thereby driving tumor proliferation and metastatic dissemination [32]. Members of the α−1,2-mannosidase family, MAN1C1 and MAN1A1, have been implicated in diverse malignancies owing to their aberrant expression patterns [33]. Notably, diminished MAN1A1 levels correlate with impaired cell–cell adhesion and unfavorable prognosis in breast carcinoma [34], whereas its upregulation portends adverse outcomes in hepatocellular and ovarian carcinomas [35, 36], underscoring context-specific functions. Emerging data further implicate augmented MAN1C1 expression in orchestrating immune and inflammatory gene modules, potentially fostering immune cell infiltration and dysregulation in gliomas [33].

Although our investigation primarily scrutinized the diagnostic utility of these genes in tuberculosis, their expression signatures may also yield prognostic and predictive value. For instance, differential expression of FHIT, MAN1A1, and MAN1C1 could mirror disease severity or therapeutic responsiveness, analogous to their roles in oncology. Future endeavors should appraise their viability as biomarkers for TB progression and treatment efficacy.

Macrophages, as frontline mediators of pathogen defense, depend on phagosomal acidification to effectuate microbial clearance. Sedlyarov et al. identified SLC4A7, a principal bicarbonate transporter, as indispensable for this acidification process [37]. By fine-tuning cytosolic pH, SLC4A7 facilitates phagosome maturation and bactericidal activity [38], whereas its perturbation compromises these critical functions. Given its centrality in macrophage physiology, SLC4A7 may bear prognostic or predictive relevance in TB, meriting in-depth exploration.

The NT5E locus encodes CD73, a 5′-nucleotidase integral to adenosine-mediated immunomodulation. Shi et al. delineated CD73/NT5E as a pivotal downstream effector of the EGFR–MEK axis, driving HPV-negative head and neck squamous cell carcinoma (HNSCC) progression via mechanisms independent of epithelial–mesenchymal transition—namely, tumor invasion and immunosuppression [39]. Similarly, AKR1C3 facilitates hepatocellular carcinoma adaptation to sorafenib by promoting lipid droplet biogenesis, inhibiting autophagy, and reprogramming metabolic circuits [40]. Elucidating whether NT5E and AKR1C3 similarly modulate TB progression or treatment outcomes could unveil novel biomarkers and therapeutic targets.

Moreover, the predictive metabolic–immune nomogram derived from these genes exhibited exceptional prognostic performance (C-index = 0.944) and yielded substantial clinical net benefit, thus furnishing a robust instrument for elucidating TB pathogenesis and guiding therapeutic innovation. Immune infiltration profiling revealed that active TB patients harbored markedly elevated M0 macrophages and neutrophils, whereas LTBI subjects maintained enriched CD4 + and CD8 + T-cell compartments, indicative of divergent immunoregulatory landscapes. The skewed polarization of M0 macrophages likely signifies early stage immune perturbation in TB, whereas sustained CD4 +/CD8 + T-cell prevalence suggests preservation of immune homeostasis in LTBI. Subsequent consensus clustering delineated two discrete molecular subtypes: one characterized by M1 macrophage predominance and the other by amplified CD8 + T-cell infiltration, thereby illuminating avenues for tailored immunotherapeutic regimens. For instance, the M1-dominant subtype may preferentially benefit from checkpoint blockade[41–43], while the CD8 + -enriched subtype could exhibit heightened responsiveness to immunostimulatory interventions[44]. Intriguingly, these TCM-derived DEGs also manifested relevance across other TB-associated disorders—such as COPD and SLE—underscoring their translational potential in multifaceted pathological milieus. Finally, RT-qPCR validation corroborated the differential expression of core genes in clinical specimens, further substantiating their utility as TB diagnostic biomarkers.

Nonetheless, this study offers invaluable insights into the tuberculosis immune microenvironment, several noteworthy limitations warrant acknowledgment. First, the cohort size, particularly within distinct clinical subsets (LTBI vs. ATB), remains relatively constrained, which may undermine the broader applicability of our conclusions. Consequently, large-scale, multicenter trials, incorporating more diverse external cohorts, are imperative to substantiate these observations and enhance the external validity of the model. Second, although our bioinformatics analysis identified significant differential expression patterns of six tuberculosis-related differentially expressed genes (TCM–DEGs), their direct involvement in tuberculosis pathogenesis remains conjectural in the absence of experimental validation. Future investigations will prioritize functional assays, such as gene knockdown and overexpression experiments, to further substantiate the mechanistic roles of these genes in modulating immune responses and T-cell metabolism in tuberculosis.

Moreover, the absence of proteomic and metabolomic validation represents another substantial limitation of this study. While transcriptomic data have provided pivotal insights into tuberculosis immune responses, alterations in proteins and metabolites may offer more immediate biological mechanisms. Future investigations integrating proteomic and metabolomic data will significantly enhance our understanding of the complexity of immune responses in tuberculosis, latent tuberculosis infection, and healthy controls.

Finally, although immune infiltration analysis revealed distinct cellular distributions, the inherent heterogeneity of immune phenotypes and variations in individual immune status may have influenced our findings. In addition, potential confounding factors (such as co-infections, demographic variables, and environmental exposures) may also modulate immune responses and should be meticulously considered when interpreting the findings. We will explicitly address these potential confounders in the revised limitations section and emphasize that this study did not fully account for these influences. Future research should incorporate more comprehensive demographic and clinical data to mitigate these confounding factors.

## Conclusion

By integrating single-cell transcriptomics, machine learning algorithms, and clinical validation, this study comprehensively elucidates the pivotal roles of T-cell-related metabolic genes (TCM–DEGs) in tuberculosis (TB) diagnosis, immune regulation, and molecular classification. Notably, this research represents the first comprehensive identification of significant differential expression patterns of FHIT, MAN1C1, SLC4A7, NT5E, and other genes in patients with tuberculosis (TB). Building on these findings, we developed a high-precision diagnostic model demonstrating exceptional performance (AUC values up to 0.873), thereby validating their potential as novel biomarkers for TB.

Furthermore, the study revealed a dynamic imbalance in the immune microenvironment of TB patients, characterized by an increased monocyte/macrophage ratio coupled with T-cell exhaustion phenotypes. This discovery offers critical insights into the immunopathogenesis of TB. In addition, the identification of two distinct molecular subtypes—M1 macrophage-enriched and CD8⁺ T-cell-infiltrated—provides a robust theoretical framework for advancing personalized immunotherapy strategies. Importantly, the broad relevance of these genes in chronic obstructive pulmonary disease (COPD) and systemic lupus erythematosus (SLE) suggests their potential involvement in cross-pathological regulatory mechanisms. While large-scale external cohort validation remains essential, this study establishes a solid foundation for the development of early diagnostic tools, precise molecular classification systems, and targeted therapeutic strategies for TB, thereby contributing to the advancement of precision medicine in infectious diseases.

## Supplementary Information

Supplementary Material 1

Supplementary Material 2

Supplementary Material 3

Supplementary Material 4

Supplementary Material 5

Supplementary Material 6

Supplementary Material 7

## Data Availability

No datasets were generated or analysed during the current study.
