# Supplementary figures and images for: Exploring T-cell metabolism in tuberculosis: development of a diagnostic model using metabolic genes
Source: Eur J Med Res. 2025 Jun 16;30:483. doi: 10.1186/s40001-025-02768-0 (PMC12168305; doi:10.1186/s40001-025-02768-0)

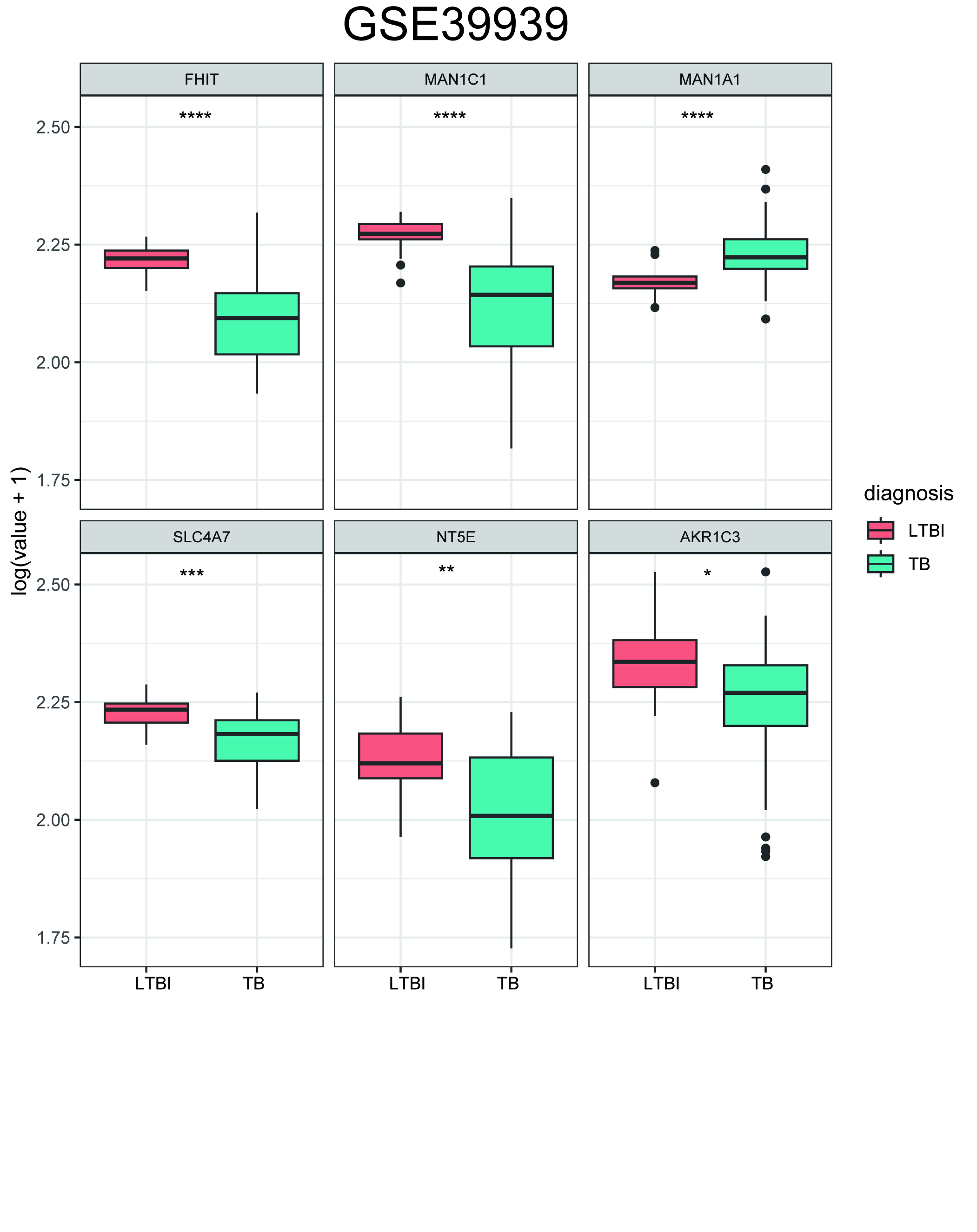

Supplement: Supplementary file 2 — Supplementary Material 2 [file 40001_2025_2768_MOESM2_ESM.tif]
