# Supplementary material for: Exploring T-cell metabolism in tuberculosis: development of a diagnostic model using metabolic genes
Source: Eur J Med Res. 2025 Jun 16;30:483. doi: 10.1186/s40001-025-02768-0 (PMC12168305; doi:10.1186/s40001-025-02768-0)
Supplement: Supplementary file 3 — Supplementary Material 3 [file 40001_2025_2768_MOESM3_ESM.pdf]

Gene Name	Gene Symbol
ATP-binding cassette, sub-family A (ABC1), member 1	ABCA1
ATP-binding cassette, sub-family A (ABC1), member 10	ABCA10
ATP-binding cassette, sub-family A (ABC1), member 12	ABCA12
ATP-binding cassette, sub-family A (ABC1), member 13	ABCA13
ATP-binding cassette, sub-family A (ABC1), member 2	ABCA2
ATP-binding cassette, sub-family A (ABC1), member 3	ABCA3
ATP-binding cassette, sub-family A (ABC1), member 4	ABCA4
ATP-binding cassette, sub-family A (ABC1), member 5	ABCA5
ATP-binding cassette, sub-family A (ABC1), member 6	ABCA6
ATP-binding cassette, sub-family A (ABC1), member 7	ABCA7
ATP-binding cassette, sub-family A (ABC1), member 8	ABCA8
ATP-binding cassette, sub-family A (ABC1), member 9	ABCA9
ATP-binding cassette, sub-family B (MDR/TAP), member 1	ABCB1
ATP-binding cassette, sub-family B (MDR/TAP), member 10	ABCB10
ATP-binding cassette, sub-family B (MDR/TAP), member 11	ABCB11
ATP-binding cassette, sub-family B (MDR/TAP), member 4	ABCB4
ATP-binding cassette, sub-family B (MDR/TAP), member 5	ABCB5
ATP-binding cassette, sub-family B (MDR/TAP), member 6	ABCB6
ATP-binding cassette, sub-family B (MDR/TAP), member 7	ABCB7
ATP-binding cassette, sub-family B (MDR/TAP), member 8	ABCB8
ATP-binding cassette, sub-family B (MDR/TAP), member 9	ABCB9
ATP-binding cassette, sub-family C (CFTR/MRP), member 1	ABCC1
ATP-binding cassette, sub-family C (CFTR/MRP), member 10	ABCC10
ATP-binding cassette, sub-family C (CFTR/MRP), member 11	ABCC11
ATP-binding cassette, sub-family C (CFTR/MRP), member 12	ABCC12
ATP-binding cassette, sub-family C (CFTR/MRP), member 13	ABCC13
ATP-binding cassette, sub-family C (CFTR/MRP), member 2	ABCC2
ATP-binding cassette, sub-family C (CFTR/MRP), member 3	ABCC3
ATP-binding cassette, sub-family C (CFTR/MRP), member 4	ABCC4
ATP-binding cassette, sub-family C (CFTR/MRP), member 5	ABCC5
ATP-binding cassette, sub-family C (CFTR/MRP), member 6	ABCC6
ATP-binding cassette, sub-family C (CFTR/MRP), member 8	ABCC8
ATP-binding cassette, sub-family C (CFTR/MRP), member 9	ABCC9
ATP-binding cassette, sub-family D (ALD), member 1	ABCD1
ATP-binding cassette, sub-family D (ALD), member 2	ABCD2
ATP-binding cassette, sub-family D (ALD), member 3	ABCD3
ATP-binding cassette, sub-family D (ALD), member 4	ABCD4
ATP-binding cassette, sub-family E (OABP), member 1	ABCE1
ATP-binding cassette, sub-family F (GCN20), member 1	ABCF1
ATP-binding cassette, sub-family F (GCN20), member 2	ABCF2
ATP-binding cassette, sub-family F (GCN20), member 3	ABCF3
ATP-binding cassette, sub-family G (WHITE), member 1	ABCG1
ATP-binding cassette, sub-family G (WHITE), member 2	ABCG2
ATP-binding cassette, sub-family G (WHITE), member 4	ABCG4
ATP-binding cassette, sub-family G (WHITE), member 5 (sterolii	ABCG5
ATP-binding cassette, sub-family G (WHITE), member 8 (sterolii	ABCG8
aminoadipate aminotransferase	AADAT

Aminoacylase 1	ACY1
Aminoacylase 1-like	ACY1L
aspartoacylase (aminocyclase) 3	ACY3
arginine decarboxylase	ADC
acireductone dioxygenase 1	ADI1
alkylglycerone phosphate synthase	AGPS
alanine-glyoxylate aminotransferase	AGXT
alanine-glyoxylate aminotransferase 2	AGXT2
alanine-glyoxylate aminotransferase 2-like 1	AGXT2L1
alanine-glyoxylate aminotransferase 2-like 2	AGXT2L2
Asparagine synthetase	ASNS
asparagine synthetase domain containing 1	ASNSD1
aspartoacylase (Canavan disease)	ASPA
asparaginase homolog (S. cerevisiae)	ASPG
asparaginase like 1	ASRGL1
cysteine conjugate-beta lyase; cytoplasmic (glutamine transamir	CCBL1
cysteine conjugate-beta lyase 2	CCBL2
crystallin, mu	CRYM
D-amino-acid oxidase	DAO
D-aspartate oxidase	DDO
glycine C-acetyltransferase (2-amino-3-ketobutyrate coenzyme A	GCAT
glutaryl-Coenzyme A dehydrogenase	GCDH
glutamic-pyruvate transaminase (alanine aminotransferase)	GPT
glutamic pyruvate transaminase (alanine aminotransferase) 2	GPT2
ilvB (bacterial acetolactate synthase)-like	ILVBL
lens protein with glutamine synthetase domain	LGSN
pterin-4 alpha-carbinolamine dehydratase/dimerization cofactor (	PCBD1
pterin-4 alpha-carbinolamine dehydratase/dimerization cofactor (	PCBD2
saccharopine dehydrogenase (putative)	SCCPDH
selenocysteine lyase	SCLY
serine dehydratase	SDS
serine dehydratase-like	SDSL
selenophosphate synthetase 1	SEPHS1
selenophosphate synthetase 2	SEPHS2
threonine synthase-like 1 (S. cerevisiae)	THNSL1
threonine synthase-like 2 (S. cerevisiae)	THNSL2
trimethyllysine hydroxylase, epsilon	TMLHE
chitinase 3-like 1 (cartilage glycoprotein-39)	CHI3L1
chitinase 3-like 2	CHI3L2
chitinase, acidic	CHIA
chitinase domain containing 1	CHID1
chitinase 1	CHIT1
Cytidine monophosphate N-acetylneuraminic acid synthetase	CMAS
glutamine-fructose-6-phosphate transaminase 1	GFPT1
glutamine-fructose-6-phosphate transaminase 2	GFPT2
glucosamine (UDP-N-acetyl)-2-epimerase/N-acetylmannosamin(	GNE
glucosamine-6-phosphate deaminase 1	GNPDA1
glucosamine-6-phosphate deaminase 2	GNPDA2

glucosamine-phosphate N-acetyltransferase 1	GNPNAT1
N-acetylglucosamine-1-phosphate transferase, alpha and beta s	GNPTAB
N-acetylglucosamine-1-phosphate transferase, gamma subunit	GNPTG
N-acetylglucosamine kinase	NAGK
N-acetylneuraminic acid phosphatase	NANP
N-acetylneuraminic acid synthase (sialic acid synthase)	NANS
N-acetylneuraminate pyruvate lyase (dihydrodipicolinate synthas	NPL
phosphoglucomutase 3	PGM3
renin binding protein	RENBP
UDP-N-acteylglucosamine pyrophosphorylase 1	UAP1
UDP-N-acteylglucosamine pyrophosphorylase 1-like 1	UAP1L1
lactate dehydrogenase A	LDHA
lactate dehydrogenase A-like 6A	LDHAL6A
lactate dehydrogenase A-like 6B	LDHAL6B
lactate dehydrogenase B	LDHB
lactate dehydrogenase C	LDHC
lactate dehydrogenase D	LDHD
ATPase type 13A1	ATP13A1
ATPase type 13A2	ATP13A2
ATPase type 13A3	ATP13A3
ATPase type 13A4	ATP13A4
ATPase type 13A5	ATP13A5
methylmalonic aciduria (cobalamin deficiency) cblB type	MMAB
methylmalonic aciduria (cobalamin deficiency) cblC type, with hc	MMACHC
methylmalonic aciduria (cobalamin deficiency) cblD type, with hc	MMADHC
5-methyltetrahydrofolate-homocysteine methyltransferase reduc	MTRR
branched chain aminotransferase 1, cytosolic	BCAT1
branched chain aminotransferase 2, mitochondrial	BCAT2
branched chain keto acid dehydrogenase E1, alpha polypeptide	BCKDHA
branched chain keto acid dehydrogenase E1, beta polypeptide (	BCKDHB
dihydrolipoamide branched chain transacylase E2	DBT
3-hydroxyisobutyrate dehydrogenase	HIBADH
3-hydroxyisobutyryl-Coenzyme A hydrolase	HIBCH
isovaleryl Coenzyme A dehydrogenase	IVD
methylcrotonoyl-Coenzyme A carboxylase 1 (alpha)	MCCC1
methylcrotonoyl-Coenzyme A carboxylase 2 (beta)	MCCC2
GTP cyclohydrolase 1 (dopa-responsive dystonia)	GCH1
6-pyruvoyltetrahydropterin synthase	PTS
quinoid dihydropteridine reductase	QDPR
sepiapterin reductase (7,8-dihydrobiopterin:NADP+ oxidoreducta	SPR
bile acid Coenzyme A: amino acid N-acyltransferase (glycine N-c	BAAT
cytochrome P450, family 27, subfamily A, polypeptide 1	CYP27A1
cytochrome P450, family 7, subfamily A, polypeptide 1	CYP7A1
cytochrome P450, family 7, subfamily B, polypeptide 1	CYP7B1
cytochrome P450, family 8, subfamily B, polypeptide 1	CYP8B1
hydroxy-delta-5-steroid dehydrogenase, 3 beta- and steroid delta	HSD3B7
fructose-1,6-bisphosphatase 1	FBP1
fructose-1,6-bisphosphatase 2	FBP2

glucose-6-phosphatase, catalytic subunit	G6PC
glucose-6-phosphatase, catalytic, 2	G6PC2
glucose 6 phosphatase, catalytic, 3	G6PC3
glucan (1,4-alpha-), branching enzyme 1 (glycogen branching er	GBE1
glycogenin 1	GYG1
glycogenin 2	GYG2
glycogen synthase 1 (muscle)	GYS1
glycogen synthase 2 (liver)	GYS2
phosphoenolpyruvate carboxykinase 1 (soluble)	PCK1
phosphoenolpyruvate carboxykinase 2 (mitochondrial)	PCK2
UDP-glucose pyrophosphorylase 2	UGP2
cytochrome P450, family 51, subfamily A, polypeptide 1	CYP51A1
24-dehydrocholesterol reductase	DHCR24
7-dehydrocholesterol reductase	DHCR7
emopamil binding protein (sterol isomerase)	EBP
emopamil binding protein-like	EBPL
farnesyl-diphosphate farnesyltransferase 1	FDFT1
farnesyl diphosphate synthase (farnesyl pyrophosphate syntheta	FDPS
hydroxysteroid (17-beta) dehydrogenase 7	HSD17B7
Lamin B receptor	LBR
lecithin-cholesterol acyltransferase	LCAT
lanosterol synthase (2,3-oxidosqualene-lanosterol cyclase)	LSS
NAD(P) dependent steroid dehydrogenase-like	NSDHL
sterol-C4-methyl oxidase-like	SC4MOL
sterol-C5-desaturase (ERG3 delta-5-desaturase homolog, S. ce	SC5DL
Squalene epoxidase	SQLE
transmembrane 7 superfamily member 2	TM7SF2
acetoacetyl-CoA synthetase	AACS
acyl-CoA thioesterase 1	ACOT1
acyl-CoA thioesterase 11	ACOT11
acyl-CoA thioesterase 12	ACOT12
acyl-CoA thioesterase 2	ACOT2
acyl-CoA thioesterase 4	ACOT4
acyl-CoA thioesterase 6	ACOT6
acyl-CoA thioesterase 7	ACOT7
Acyl-CoA thioesterase 8	ACOT8
acyl-CoA thioesterase 9	ACOT9
Coenzyme A synthase	COASY
pantothenate kinase 1	PANK1
pantothenate kinase 2 (Hallervorden-Spatz syndrome)	PANK2
pantothenate kinase 3	PANK3
pantothenate kinase 4	PANK4
phosphopantothenoylcysteine decarboxylase	PPCDC
phosphopantothenoylcysteine synthetase	PPCS
S-adenosylhomocysteine hydrolase	AHCY
S-adenosylhomocysteine hydrolase-like 1	AHCYL1
S-adenosylhomocysteine hydrolase-like 2	AHCYL2
butyrobetaine (gamma), 2-oxoglutarate dioxygenase (gamma-bu	BBOX1

biotinidase	BTD
carboxyl ester lipase (bile salt-stimulated lipase)	CEL
carnitine acetyltransferase	CRAT
cubilin (intrinsic factor-cobalamin receptor)	CUBN
cytochrome P450, family 24, subfamily A, polypeptide 1	CYP24A1
cytochrome P450, family 27, subfamily B, polypeptide 1	CYP27B1
dihydroxyacetone kinase 2 homolog (S. cerevisiae)	DAK
FAD1 flavin adenine dinucleotide synthetase homolog (S. cerevi	FLAD1
folate hydrolase (prostate-specific membrane antigen) 1	FOLH1
gephyrin	GPHN
holocarboxylase synthetase (biotin-(proprionyl-Coenzyme A-cark	HLCS
molybdenum cofactor sulfurase	MOCOS
molybdenum cofactor synthesis 1	MOCS1
molybdenum cofactor synthesis 2	MOCS2
molybdenum cofactor synthesis 3	MOCS3
N-acetylglutamate synthase	NAGS
riboflavin kinase	RFK
Thiamine triphosphatase	THTPA
thiamin pyrophosphokinase 1	TPK1
vitamin K epoxide reductase complex, subunit 1	VKORC1
vitamin K epoxide reductase complex, subunit 1-like 1	VKORC1L1
michondrially encoded NADH dehydrogenase 1	MT-ND1
michondrially encoded NADH dehydrogenase 2	MT-ND2
michondrially encoded NADH dehydrogenase 3	MT-ND3
michondrially encoded NADH dehydrogenase 4	MT-ND4
michondrially encoded NADH dehydrogenase 4 like	MT-ND4L
michondrially encoded NADH dehydrogenase 5	MT-ND5
michondrially encoded NADH dehydrogenase 6	MT-ND6
NADH dehydrogenase (ubiquinone) 1 alpha subcomplex, 1, 7.5k	NDUFA1
NADH dehydrogenase (ubiquinone) 1 alpha subcomplex, 10, 42	NDUFA10
NADH dehydrogenase (ubiquinone) 1 alpha subcomplex, 11, 14	NDUFA11
NADH dehydrogenase (ubiquinone) 1 alpha subcomplex, 12	NDUFA12
NADH dehydrogenase (ubiquinone) 1 alpha subcomplex, 13	NDUFA13
NADH dehydrogenase (ubiquinone) 1 alpha subcomplex, 2, 8kD	NDUFA2
NADH dehydrogenase (ubiquinone) 1 alpha subcomplex, 3, 9kD	NDUFA3
NADH dehydrogenase (ubiquinone) 1 alpha subcomplex, 4, 9kD	NDUFA4
NADH dehydrogenase (ubiquinone) 1 alpha subcomplex, 4-like	NDUFA4L
NADH dehydrogenase (ubiquinone) 1 alpha subcomplex, 4-like 2	NDUFA4L2
NADH dehydrogenase (ubiquinone) 1 alpha subcomplex, 5, 13k	NDUFA5
NADH dehydrogenase (ubiquinone) 1 alpha subcomplex, 6, 14k	NDUFA6
NADH dehydrogenase (ubiquinone) 1 alpha subcomplex, 7, 14.5	NDUFA7
NADH dehydrogenase (ubiquinone) 1 alpha subcomplex, 8, 19k	NDUFA8
NADH dehydrogenase (ubiquinone) 1 alpha subcomplex, 9, 39k	NDUFA9
NADH dehydrogenase (ubiquinone) 1, alpha/beta subcomplex, 1	NDUFAB1
NADH dehydrogenase (ubiquinone) 1 alpha subcomplex, assem	NDUFAF1
NADH dehydrogenase (ubiquinone) 1 alpha subcomplex, assem	NDUFAF2
NADH dehydrogenase (ubiquinone) 1 beta subcomplex, 1, 7kDa	NDUFB1
NADH dehydrogenase (ubiquinone) 1 beta subcomplex, 10, 22k	NDUFB10

NADH dehydrogenase (ubiquinone) 1 beta subcomplex, 11, 17.3	NDUFB11
NADH dehydrogenase (ubiquinone) 1 beta subcomplex, 2, 8kDa	NDUFB2
NADH dehydrogenase (ubiquinone) 1 beta subcomplex, 3, 12kD	NDUFB3
NADH dehydrogenase (ubiquinone) 1 beta subcomplex, 4, 12kD	NDUFB4
NADH dehydrogenase (ubiquinone) 1 beta subcomplex, 5, 16kD	NDUFB5
NADH dehydrogenase (ubiquinone) 1 beta subcomplex, 6, 17kD	NDUFB6
NADH dehydrogenase (ubiquinone) 1 beta subcomplex, 7, 18kD	NDUFB7
NADH dehydrogenase (ubiquinone) 1 beta subcomplex, 8, 19kD	NDUFB8
NADH dehydrogenase (ubiquinone) 1 beta subcomplex, 9, 22kD	NDUFB9
NADH dehydrogenase (ubiquinone) 1, subcomplex unknown, 1,	NDUFC1
NADH dehydrogenase (ubiquinone) 1, subcomplex unknown, 2,	NDUFC2
NADH dehydrogenase (ubiquinone) Fe-S protein 1, 75kDa (NAD	NDUFS1
NADH dehydrogenase (ubiquinone) Fe-S protein 2, 49kDa (NAD	NDUFS2
NADH dehydrogenase (ubiquinone) Fe-S protein 3, 30kDa (NAD	NDUFS3
NADH dehydrogenase (ubiquinone) Fe-S protein 4, 18kDa (NAD	NDUFS4
NADH dehydrogenase (ubiquinone) Fe-S protein 5, 15kDa (NAD	NDUFS5
NADH dehydrogenase (ubiquinone) Fe-S protein 6, 13kDa (NAD	NDUFS6
NADH dehydrogenase (ubiquinone) Fe-S protein 7, 20kDa (NAD	NDUFS7
NADH dehydrogenase (ubiquinone) Fe-S protein 8, 23kDa (NAD	NDUFS8
NADH dehydrogenase (ubiquinone) flavoprotein 1, 51kDa	NDUFV1
NADH dehydrogenase (ubiquinone) flavoprotein 2, 24kDa	NDUFV2
NADH dehydrogenase (ubiquinone) flavoprotein 3, 10kDa	NDUFV3
succinate dehydrogenase complex, subunit A, flavoprotein (Fp)	SDHA
succinate dehydrogenase complex, subunit B, iron sulfur (Ip)	SDHB
succinate dehydrogenase complex, subunit C, integral membran	SDHC
succinate dehydrogenase complex, subunit D, integral membran	SDHD
cytochrome c-1	CYC1
cytochrome c, somatic	CYCS
Cytochrome b	MT-CYB
ubiquinol-cytochrome c reductase complex (7.2 kD)	UCRC
ubiquinol-cytochrome c reductase, 6.4kDa subunit	UQCR
ubiquinol-cytochrome c reductase binding protein	UQCRB
ubiquinol-cytochrome c reductase core protein I	UQCRC1
ubiquinol-cytochrome c reductase core protein II	UQCRC2
ubiquinol-cytochrome c reductase, Rieske iron-sulfur polypeptide	UQCRFS1
ubiquinol-cytochrome c reductase hinge protein	UQCRH
ubiquinol-cytochrome c reductase, complex III subunit VII, 9.5kD	UQCRQ
mitochondrially encoded cytochrome c oxidase I	COX1
COX10 homolog, cytochrome c oxidase assembly protein, heme	COX10
COX11 homolog, cytochrome c oxidase assembly protein (yeast	COX11
COX15 homolog, cytochrome c oxidase assembly protein (yeast	COX15
COX16 cytochrome c oxidase assembly homolog (S. cerevisiae)	COX16
COX17 cytochrome c oxidase assembly homolog (S. cerevisiae)	COX17
COX18 cytochrome c oxidase assembly homolog (S. cerevisiae)	COX18
COX19 cytochrome c oxidase assembly homolog (S. cerevisiae)	COX19
mitochondrially encoded cytochrome c oxidase II	COX2
mitochondrially encoded cytochrome c oxidase III	COX3
cytochrome c oxidase subunit IV isoform 1	COX4I1

cytochrome c oxidase subunit IV isoform 2 (lung)	COX4I2
cytochrome c oxidase subunit Va	COX5A
Cytochrome c oxidase subunit Vb	COX5B
cytochrome c oxidase subunit VIa polypeptide 1	COX6A1
cytochrome c oxidase subunit VIa polypeptide 2	COX6A2
cytochrome c oxidase subunit Vib polypeptide 1 (ubiquitous)	COX6B1
cytochrome c oxidase subunit VIb polypeptide 2 (testis)	COX6B2
cytochrome c oxidase subunit VIc	COX6C
cytochrome c oxidase subunit VIIa polypeptide 1 (muscle)	COX7A1
cytochrome c oxidase subunit VIIa polypeptide 2 (liver)	COX7A2
cytochrome c oxidase subunit VIIa polypeptide 2 like	COX7A2L
cytochrome c oxidase subunit VIIb	COX7B
cytochrome c oxidase subunit VIIb2	COX7B2
cytochrome c oxidase subunit VIIc	COX7C
cytochrome c oxidase subunit 8A (ubiquitous)	COX8A
cytochrome c oxidase subunit 8C	COX8C
creatine kinase, brain	CKB
creatine kinase, ectopic expression	CKBE
creatine kinase, muscle	CKM
creatine kinase, mitochondrial 1A	CKMT1A
creatine kinase, mitochondrial 1B	CKMT1B
creatine kinase, mitochondrial 2 (sarcomeric)	CKMT2
guanidinoacetate N-methyltransferase	GAMT
glycine amidinotransferase (L-arginine:glycine amidinotransferas	GATM
2-aminoethanethiol (cysteamine) dioxygenase	ADO
cystathionine-beta-synthase	CBS
cysteine dioxygenase, type I	CDO1
cysteine sulfinic acid decarboxylase	CSAD
cystathionase (cystathionine gamma-lyase)	CTH
NFS1 nitrogen fixation 1 homolog (S. cerevisiae)	NFS1
prenylcysteine oxidase 1	PCYOX1
prenylcysteine oxidase 1 like	PCYOX1L
sulfite oxidase	SUOX
arylacetamide deacetylase (esterase)	AADAC
arylacetamide deacetylase-like 1	AADACL1
arylacetamide deacetylase-like 2	AADACL2
arylacetamide deacetylase-like 3	AADACL3
arylacetamide deacetylase-like 4	AADACL4
aldo-keto reductase family 7-like	AKR7L
butyrylcholinesterase	BCHE
Catalase	CAT
carboxylesterase 1 (monocyte/macrophage serine esterase 1)	CES1
carboxylesterase 2 (intestine, liver)	CES2
carboxylesterase 3 (brain)	CES3
carboxylesterase 7	CES7
dihydrodiol dehydrogenase (dimeric)	DHDH
dipeptidase 1 (renal)	DPEP1
dipeptidase 2	DPEP2

dipeptidase 3	DPEP3
epoxide hydrolase 1, microsomal (xenobiotic)	EPHX1
epoxide hydrolase 2, cytoplasmic	EPHX2
flavin containing monooxygenase 1	FMO1
flavin containing monooxygenase 2	FMO2
flavin containing monooxygenase 3	FMO3
flavin containing monooxygenase 4	FMO4
flavin containing monooxygenase 5	FMO5
superoxide dismutase 1, soluble (amyotrophic lateral sclerosis 1	SOD1
Superoxide dismutase 2, mitochondrial	SOD2
superoxide dismutase 3, extracellular	SOD3
thiopurine S-methyltransferase	TPMT
UDP glucuronosyltransferase 1 family, polypeptide A1	UGT1A1
UDP glucuronosyltransferase 1 family, polypeptide A10	UGT1A10
UDP glucuronosyltransferase 1 family, polypeptide A3	UGT1A3
UDP glucuronosyltransferase 1 family, polypeptide A4	UGT1A4
UDP glucuronosyltransferase 1 family, polypeptide A5	UGT1A5
UDP glucuronosyltransferase 1 family, polypeptide A6	UGT1A6
UDP glucuronosyltransferase 1 family, polypeptide A7	UGT1A7
UDP glucuronosyltransferase 1 family, polypeptide A8	UGT1A8
UDP glucuronosyltransferase 1 family, polypeptide A9	UGT1A9
UDP glucuronosyltransferase 2 family, polypeptide A1	UGT2A1
UDP glucuronosyltransferase 2 family, polypeptide A2	UGT2A2
UDP glucuronosyltransferase 2 family, polypeptide A3	UGT2A3
UDP glucuronosyltransferase 2 family, polypeptide B10	UGT2B10
UDP glucuronosyltransferase 2 family, polypeptide B11	UGT2B11
UDP glucuronosyltransferase 2 family, polypeptide B15	UGT2B15
UDP glucuronosyltransferase 2 family, polypeptide B17	UGT2B17
UDP glucuronosyltransferase 2 family, polypeptide B28	UGT2B28
UDP glucuronosyltransferase 2 family, polypeptide B4	UGT2B4
UDP glucuronosyltransferase 2 family, polypeptide B7	UGT2B7
UDP glycosyltransferase 3 family, polypeptide A1	UGT3A1
UDP glycosyltransferase 3 family, polypeptide A2	UGT3A2
acetyl-Coenzyme A acyltransferase 1 (peroxisomal 3-oxoacyl-Co	ACAA1
acetyl-Coenzyme A acyltransferase 2 (mitochondrial 3-oxoacyl-C	ACAA2
acetyl-Coenzyme A carboxylase alpha	ACACA
acetyl-Coenzyme A carboxylase beta	ACACB
acyl-Coenzyme A dehydrogenase family, member 10	ACAD10
acyl-Coenzyme A dehydrogenase family, member 11	ACAD11
acyl-Coenzyme A dehydrogenase family, member 8	ACAD8
acyl-Coenzyme A dehydrogenase family, member 9	ACAD9
acyl-Coenzyme A dehydrogenase, long chain	ACADL
acyl-Coenzyme A dehydrogenase, C-4 to C-12 straight chain	ACADM
acyl-Coenzyme A dehydrogenase, C-2 to C-3 short chain	ACADS
acyl-Coenzyme A dehydrogenase, short/branched chain	ACADSB
acyl-Coenzyme A dehydrogenase, very long chain	ACADVL
acetyl-Coenzyme A acetyltransferase 1 (acetoacetyl Coenzyme	ACAT1
acetyl-Coenzyme A acetyltransferase 2 (acetoacetyl Coenzyme	ACAT2

acyl-Coenzyme A binding domain containing 3	ACBD3
acyl-Coenzyme A binding domain containing 4	ACBD4
acyl-Coenzyme A binding domain containing 5	ACBD5
acyl-Coenzyme A binding domain containing 6	ACBD6
acyl-Coenzyme A binding domain containing 7	ACBD7
acyl-Coenzyme A oxidase 1, palmitoyl	ACOX1
acyl-Coenzyme A oxidase 2, branched chain	ACOX2
acyl-Coenzyme A oxidase 3, pristanoyl	ACOX3
acyl-Coenzyme A oxidase-like	ACOXL
acyl-CoA synthetase bubblegum family member 1	ACSBG1
acyl-CoA synthetase bubblegum family member 2	ACSBG2
acyl-CoA synthetase family member 2	ACSF2
acyl-CoA synthetase family member 3	ACSF3
acyl-CoA synthetase long-chain family member 1	ACSL1
acyl-CoA synthetase long-chain family member 3	ACSL3
Acyl-CoA synthetase long-chain family member 4	ACSL4
acyl-CoA synthetase long-chain family member 5	ACSL5
acyl-CoA synthetase long-chain family member 6	ACSL6
acyl-CoA synthetase medium-chain family member 1	ACSM1
acyl-CoA synthetase medium-chain family member 2A	ACSM2A
acyl-CoA synthetase medium-chain family member 2B	ACSM2B
acyl-CoA synthetase medium-chain family member 3	ACSM3
acyl-CoA synthetase medium-chain family member 4	ACSM4
acyl-CoA synthetase medium-chain family member 5	ACSM5
Acyl-CoA synthetase short-chain family member 1	ACSS1
acyl-CoA synthetase short-chain family member 2	ACSS2
acyl-CoA synthetase short-chain family member 3	ACSS3
citrate lyase beta like	CLYBL
carnitine palmitoyltransferase 1A (liver)	CPT1A
carnitine palmitoyltransferase 1B (muscle)	CPT1B
carnitine palmitoyltransferase 1C	CPT1C
carnitine palmitoyltransferase II	CPT2
cytochrome P450, family 4, subfamily A, polypeptide 11	CYP4A11
cytochrome P450, family 4, subfamily A, polypeptide 22	CYP4A22
cytochrome P450, family 4, subfamily B, polypeptide 1	CYP4B1
cytochrome P450, family 4, subfamily F, polypeptide 11	CYP4F11
cytochrome P450, family 4, subfamily F, polypeptide 12	CYP4F12
cytochrome P450, family 4, subfamily F, polypeptide 2	CYP4F2
cytochrome P450, family 4, subfamily F, polypeptide 22	CYP4F22
cytochrome P450, family 4, subfamily F, polypeptide 3	CYP4F3
cytochrome P450, family 4, subfamily F, polypeptide 8	CYP4F8
cytochrome P450, family 4, subfamily V, polypeptide 2	CYP4V2
cytochrome P450, family 4, subfamily X, polypeptide 1	CYP4X1
cytochrome P450, family 4, subfamily Z, polypeptide 1	CYP4Z1
dodecenoyl-Coenzyme A delta isomerase (3,2 trans-enoyl-Coen	DCI
2,4-dienoyl CoA reductase 1, mitochondrial	DECR1
2,4-dienoyl CoA reductase 2, peroxisomal	DECR2
diacylglycerol O-acyltransferase homolog 1 (mouse)	DGAT1

diacylglycerol O-acyltransferase homolog 2 (mouse)	DGAT2
diacylglycerol O-acyltransferase 2-like 3	DGAT2L3
diacylglycerol O-acyltransferase 2-like 4	DGAT2L4
diacylglycerol O-acyltransferase 2-like 6	DGAT2L6
diacylglycerol O-acyltransferase 2-like 7	DGAT2L7
enoyl Coenzyme A hydratase 1, peroxisomal	ECH1
enoyl Coenzyme A hydratase domain containing 1	ECHDC1
enoyl Coenzyme A hydratase domain containing 2	ECHDC2
enoyl Coenzyme A hydratase domain containing 3	ECHDC3
enoyl Coenzyme A hydratase, short chain, 1, mitochondrial	ECHS1
enoyl-Coenzyme A, hydratase/3-hydroxyacyl Coenzyme A dehy	EHHADH
elongation of very long chain fatty acids (FEN1/Elo2, SUR4/Elo3	ELOVL1
elongation of very long chain fatty acids (FEN1/Elo2, SUR4/Elo3	ELOVL2
elongation of very long chain fatty acids (FEN1/Elo2, SUR4/Elo3	ELOVL3
elongation of very long chain fatty acids (FEN1/Elo2, SUR4/Elo3	ELOVL4
ELOVL family member 5, elongation of long chain fatty acids (FE	ELOVL5
ELOVL family member 6, elongation of long chain fatty acids (FE	ELOVL6
ELOVL family member 7, elongation of long chain fatty acids (ye	ELOVL7
electron-transfer-flavoprotein, alpha polypeptide (glutaric aciduri	ETFA
electron-transfer-flavoprotein, beta polypeptide	ETFB
electron-transferring-flavoprotein dehydrogenase	ETFDH
fatty acid 2-hydroxylase	FA2H
fatty acid amide hydrolase	FAAH
fatty acid amide hydrolase 2	FAAH2
fatty acid desaturase 1	FADS1
fatty acid desaturase 2	FADS2
fatty acid desaturase 3	FADS3
fatty acyl CoA reductase 1	FAR1
fatty acyl CoA reductase 2	FAR2
fatty acid synthase	FASN
glycoprotein, synaptic 2	GPSN2
2-hydroxyacyl-CoA lyase 1	HACL1
hydroxyacyl-Coenzyme A dehydrogenase	HADH
hydroxyacyl-Coenzyme A dehydrogenase/3-ketoacyl-Coenzyme	HADHA
hydroxyacyl-Coenzyme A dehydrogenase/3-ketoacyl-Coenzyme	HADHB
lipase, hepatic	LIPC
lipase, gastric	LIPF
lipase, endothelial	LIPG
lipase, member H	LIPH
lipase, member I	LIPI
lipase, family member J	LIPJ
lipase, mamber K	LIPK
lipase, member M	LIPM
lipase, member N	LIPN
lipoprotein lipase	LPL
lipid phosphate phosphatase-related protein type 2	LPPR2
malonyl CoA:ACP acyltransferase (mitochondrial)	MCAT
mitochondrial trans-2-enoyl-CoA reductase	MECR

monoglyceride lipase	MGLL
malonyl-CoA decarboxylase	MLYCD
microsomal triglyceride transfer protein	MTTP
oleoyl-ACP hydrolase	OLAH
3-oxoacyl-ACP synthase, mitochondrial	OXSM
peroxisomal D3,D2-enoyl-CoA isomerase	PECI
peroxisomal trans-2-enoyl-CoA reductase	PECR
phytanoyl-CoA 2-hydroxylase	PHYH
phytanoyl-CoA dioxygenase domain containing 1	PHYHD1
pancreatic lipase	PNLIP
pancreatic lipase-related protein 1	PNLIPRP1
pancreatic lipase-related protein 2	PNLIPRP2
pancreatic lipase-related protein 3	PNLIPRP3
patatin-like phospholipase domain containing 1	PNPLA1
patatin-like phospholipase domain containing 2	PNPLA2
patatin-like phospholipase domain containing 3	PNPLA3
patatin-like phospholipase domain containing 4	PNPLA4
patatin-like phospholipase domain containing 5	PNPLA5
patatin-like phospholipase domain containing 6	PNPLA6
patatin-like phospholipase domain containing 7	PNPLA7
patatin-like phospholipase domain containing 8	PNPLA8
stearoyl-CoA desaturase (delta-9-desaturase)	SCD
stearoyl-CoA desaturase 5	SCD5
sterol carrier protein 2	SCP2
short chain dehydrogenase/reductase family 16C, member 6	SDR16C6
short chain dehydrogenase/reductase family 39U, member 1	SDR39U1
short chain dehydrogenase/reductase family 42E, member 1	SDR42E1
short chain dehydrogenase/reductase family 9C, member 7	SDR9C7
dihydrofolate reductase	DHFR
dihydrofolate reductase-like 1	DHFRL1
folylpolyglutamate synthase	FPGS
methylenetetrahydrofolate dehydrogenase (NADP+ dependent)	MTHFD1
Methylenetetrahydrofolate dehydrogenase (NADP+ dependent)	MTHFD1L
methylenetetrahydrofolate dehydrogenase (NADP+ dependent)	MTHFD2
methylenetetrahydrofolate dehydrogenase (NADP+ dependent)	MTHFD2L
5,10-methylenetetrahydrofolate reductase (NADPH)	MTHFR
5,10-methenyltetrahydrofolate synthetase (5-formyltetrahydrofola	MTHFS
methenyltetrahydrofolate synthetase domain containing	MTHFSD
serine hydroxymethyltransferase 1 (soluble)	SHMT1
serine hydroxymethyltransferase 2 (mitochondrial)	SHMT2
aldehyde dehydrogenase 18 family, member A1	ALDH18A1
aldehyde dehydrogenase 5 family, member A1 (succinate-semia	ALDH5A1
glutamate decarboxylase 1 (brain, 67kDa)	GAD1
Glutamate decarboxylase 2 (pancreatic islets and brain, 65kDa)	GAD2
glutamate decarboxylase-like 1	GADL1
glutaminase	GLS
glutaminase 2 (liver, mitochondrial)	GLS2
glutamate dehydrogenase 1	GLUD1

glutamate dehydrogenase 2	GLUD2
glutamate-ammonia ligase (glutamine synthetase)	GLUL
ornithine aminotransferase (gyrate atrophy)	OAT
glutamate-cysteine ligase, catalytic subunit	GCLC
glutamate-cysteine ligase, modifier subunit	GCLM
glutathione peroxidase 1	GPX1
glutathione peroxidase 2 (gastrointestinal)	GPX2
glutathione peroxidase 3 (plasma)	GPX3
glutathione peroxidase 4 (phospholipid hydroperoxidase)	GPX4
glutathione peroxidase 5 (epididymal androgen-related protein)	GPX5
glutathione peroxidase 6 (olfactory)	GPX6
glutathione peroxidase 7	GPX7
glutathione peroxidase 8 (putative)	GPX8
glutathione reductase	GSR
glutathione synthetase	GSS
glutathione S-transferase A1	GSTA1
glutathione S-transferase A2	GSTA2
glutathione S-transferase A3	GSTA3
glutathione S-transferase A4	GSTA4
glutathione S-transferase A5	GSTA5
glutathione S-transferase, C-terminal domain containing	GSTCD
glutathione S-transferase kappa 1	GSTK1
glutathione S-transferase M1	GSTM1
glutathione S-transferase M2 (muscle)	GSTM2
glutathione S-transferase M3 (brain)	GSTM3
glutathione S-transferase M4	GSTM4
glutathione S-transferase M5	GSTM5
glutathione S-transferase omega 1	GSTO1
glutathione S-transferase omega 2	GSTO2
glutathione S-transferase pi	GSTP1
glutathione S-transferase theta 1	GSTT1
glutathione S-transferase theta 2	GSTT2
hydroxyacylglutathione hydrolase	HAGH
hydroxyacylglutathione hydrolase-like	HAGHL
microsomal glutathione S-transferase 1	MGST1
microsomal glutathione S-transferase 2	MGST2
microsomal glutathione S-transferase 3	MGST3
alpha 1,4-galactosyltransferase (globotriaosylceramide synthase	A4GALT
alpha-1,4-N-acetylglucosaminyltransferase	A4GNT
ABO blood group (transferase A, alpha 1-3-N-acetylgalactosami	ABO
asparagine-linked glycosylation 1 homolog (S. cerevisiae, beta-1	ALG1
asparagine-linked glycosylation 10 homolog (yeast, alpha-1,2-gl	ALG10
asparagine-linked glycosylation 10 homolog B (yeast, alpha-1,2-	ALG10B
asparagine-linked glycosylation 11 homolog	ALG11
asparagine-linked glycosylation 12 homolog (S. cerevisiae, alpha	ALG12
asparagine-linked glycosylation 13 homolog (S. cerevisiae)	ALG13
asparagine-linked glycosylation 14 homolog (S. cerevisiae)	ALG14
asparagine-linked glycosylation 2 homolog (S. cerevisiae, alpha-	ALG2

asparagine-linked glycosylation 3 homolog (S. cerevisiae, alpha-	ALG3
Asparagine-linked glycosylation 5 homolog (S. cerevisiae, dolich	ALG5
asparagine-linked glycosylation 6 homolog (S. cerevisiae, alpha-	ALG6
asparagine-linked glycosylation 8 homolog (S. cerevisiae, alpha-	ALG8
asparagine-linked glycosylation 9 homolog (S. cerevisiae, alpha-	ALG9
beta-1,3-N-acetylgalactosaminyltransferase 1 (globoside blood g	B3GALNT1
beta-1,3-N-acetylgalactosaminyltransferase 2	B3GALNT2
UDP-Gal:betaGlcNAc beta 1,3-galactosyltransferase, polypeptid	B3GALT1
UDP-Gal:betaGlcNAc beta 1,3-galactosyltransferase, polypeptid	B3GALT2
UDP-Gal:betaGlcNAc beta 1,3-galactosyltransferase, polypeptid	B3GALT4
UDP-Gal:betaGlcNAc beta 1,3-galactosyltransferase, polypeptid	B3GALT5
UDP-Gal:betaGal beta 1,3-galactosyltransferase polypeptide 6	B3GALT6
beta 1,3-galactosyltransferase-like	B3GALTL
beta-1,3-glucuronyltransferase 1 (glucuronosyltransferase P)	B3GAT1
beta-1,3-glucuronyltransferase 2 (glucuronosyltransferase P)	B3GAT2
beta-1,3-glucuronyltransferase 3 (glucuronosyltransferase I)	B3GAT3
UDP-GlcNAc:betaGal beta-1,3-N-acetylglucosaminyltransferase	B3GNT1
UDP-GlcNAc:betaGal beta-1,3-N-acetylglucosaminyltransferase	B3GNT2
UDP-GlcNAc:betaGal beta-1,3-N-acetylglucosaminyltransferase	B3GNT3
UDP-GlcNAc:betaGal beta-1,3-N-acetylglucosaminyltransferase	B3GNT4
UDP-GlcNAc:betaGal beta-1,3-N-acetylglucosaminyltransferase	B3GNT5
UDP-GlcNAc:betaGal beta-1,3-N-acetylglucosaminyltransferase	B3GNT6
UDP-GlcNAc:betaGal beta-1,3-N-acetylglucosaminyltransferase	B3GNT7
UDP-GlcNAc:betaGal beta-1,3-N-acetylglucosaminyltransferase	B3GNT8
UDP-GlcNAc:betaGal beta-1,3-N-acetylglucosaminyltransferase	B3GNT9
UDP-GlcNAc:betaGal beta-1,3-N-acetylglucosaminyltransferase	B3GNTL1
beta-1,4-N-acetyl-galactosaminyl transferase 1	B4GALNT1
beta-1,4-N-acetyl-galactosaminyl transferase 2	B4GALNT2
beta-1,4-N-acetyl-galactosaminyl transferase 3	B4GALNT3
beta-1,4-N-acetyl-galactosaminyl transferase 4	B4GALNT4
UDP-Gal:betaGlcNAc beta 1,4- galactosyltransferase, polypeptic	B4GALT1
UDP-Gal:betaGlcNAc beta 1,4- galactosyltransferase, polypeptic	B4GALT2
UDP-Gal:betaGlcNAc beta 1,4- galactosyltransferase, polypeptic	B4GALT3
UDP-Gal:betaGlcNAc beta 1,4- galactosyltransferase, polypeptic	B4GALT4
UDP-Gal:betaGlcNAc beta 1,4- galactosyltransferase, polypeptic	B4GALT5
UDP-Gal:betaGlcNAc beta 1,4- galactosyltransferase, polypeptic	B4GALT6
xylosylprotein beta 1,4-galactosyltransferase, polypeptide 7 (gala	B4GALT7
core 1 synthase, glycoprotein-N-acetylgalactosamine 3-beta-gala	C1GALT1
chitobiase, di-N-acetyl-	CTBS
defender against cell death	DAD1
dolichyl-diphosphooligosaccharide-protein glycosyltransferase	DDOST
dehydrodolichyl diphosphate synthase	DHDDS
dolichol kinase	DOLK
dolichyl pyrophosphate phosphatase 1	DOLPP1
dolichyl-phosphate (UDP-N-acetylglucosamine) N-acetylglucosa	DPAGT1
fructosamine 3 kinase	FN3K
fructosamine-3-kinase-related protein	FN3KRP
fucosyltransferase 1 (galactoside 2-alpha-L-fucosyltransferase, I	FUT1

fucosyltransferase 10 (alpha (1,3) fucosyltransferase)	FUT10
fucosyltransferase 11 (alpha (1,3) fucosyltransferase)	FUT11
fucosyltransferase 2 (secretor status included)	FUT2
fucosyltransferase 3 (galactoside 3(4)-L-fucosyltransferase, Lew	FUT3
fucosyltransferase 4 (alpha (1,3) fucosyltransferase, myeloid-spe	FUT4
fucosyltransferase 5 (alpha (1,3) fucosyltransferase)	FUT5
Fucosyltransferase 6 (alpha (1,3) fucosyltransferase)	FUT6
fucosyltransferase 7 (alpha (1,3) fucosyltransferase)	FUT7
fucosyltransferase 8 (alpha (1,6) fucosyltransferase)	FUT8
fucosyltransferase 9 (alpha (1,3) fucosyltransferase)	FUT9
UDP-galactose-4-epimerase	GALE
UDP-N-acetyl-alpha-D-galactosamine:polypeptide N-acetylgalac	GALNT1
UDP-N-acetyl-alpha-D-galactosamine:polypeptide N-acetylgalac	GALNT10
UDP-N-acetyl-alpha-D-galactosamine:polypeptide N-acetylgalac	GALNT11
UDP-N-acetyl-alpha-D-galactosamine:polypeptide N-acetylgalac	GALNT12
UDP-N-acetyl-alpha-D-galactosamine:polypeptide N-acetylgalac	GALNT13
UDP-N-acetyl-alpha-D-galactosamine:polypeptide N-acetylgalac	GALNT14
UDP-N-acetyl-alpha-D-galactosamine:polypeptide N-acetylgalac	GALNT2
UDP-N-acetyl-alpha-D-galactosamine:polypeptide N-acetylgalac	GALNT3
UDP-N-acetyl-alpha-D-galactosamine:polypeptide N-acetylgalac	GALNT4
UDP-N-acetyl-alpha-D-galactosamine:polypeptide N-acetylgalac	GALNT5
UDP-N-acetyl-alpha-D-galactosamine:polypeptide N-acetylgalac	GALNT6
UDP-N-acetyl-alpha-D-galactosamine:polypeptide N-acetylgalac	GALNT7
UDP-N-acetyl-alpha-D-galactosamine:polypeptide N-acetylgalac	GALNT8
UDP-N-acetyl-alpha-D-galactosamine:polypeptide N-acetylgalac	GALNT9
UDP-N-acetyl-alpha-D-galactosamine:polypeptide N-acetylgalac	GALNTL1
UDP-N-acetyl-alpha-D-galactosamine:polypeptide N-acetylgalac	GALNTL2
UDP-N-acetyl-alpha-D-galactosamine:polypeptide N-acetylgalac	GALNTL4
UDP-N-acetyl-alpha-D-galactosamine:polypeptide N-acetylgalac	GALNTL5
UDP-N-acetyl-alpha-D-galactosamine:polypeptide N-acetylgalac	GALNTL6
glucosidase, alpha; neutral AB	GANAB
glucosidase, alpha; neutral C	GANC
glucosidase, beta; acid (includes glucosylceramidase)	GBA
glucosidase, beta (bile acid) 2	GBA2
glucosidase, beta, acid 3 (cytosolic)	GBA3
globoside alpha-1,3-N-acetylgalactosaminyltransferase 1	GBGT1
glucosaminyl (N-acetyl) transferase 1, core 2 (beta-1,6-N-acetylg	GCNT1
glucosaminyl (N-acetyl) transferase 2, I-branching enzyme (I blo	GCNT2
glucosaminyl (N-acetyl) transferase 3, mucin type	GCNT3
glucosaminyl (N-acetyl) transferase 4, core 2 (beta-1,6-N-acetylg	GCNT4
glucosaminyl (N-acetyl) transferase family member 6	GCNT6
glucosaminyl (N-acetyl) transferase family member 7	GCNT7
glucosidase I	GCS1
glycosyltransferase 1 domain containing 1	GLT1D1
glycosyltransferase 25 domain containing 1	GLT25D1
glycosyltransferase 25 domain containing 2	GLT25D2
glycosyltransferase 6 domain containing 1	GLT6D1
glycosyltransferase 8 domain containing 1	GLT8D1

glycosyltransferase 8 domain containing 2	GLT8D2
glycosyltransferase 8 domain containing 3	GLT8D3
glycosyltransferase 8 domain containing 4	GLT8D4
glycosyltransferase-like domain containing 1	GTDC1
glycosyltransferase-like 1B	GYLTL1B
hyaluronan synthase 1	HAS1
hyaluronan synthase 2	HAS2
hyaluronan synthase 3	HAS3
like-glycosyltransferase	LARGE
lysocardiolipin acyltransferase	LYCAT
MFNG O-fucosylpeptide 3-beta-N-acetylglucosaminyltransferase	MFNG
mannosyl (alpha-1,3-)-glycoprotein beta-1,2-N-acetylglucosamin	MGAT1
mannosyl (alpha-1,6-)-glycoprotein beta-1,2-N-acetylglucosamin	MGAT2
mannosyl (beta-1,4-)-glycoprotein beta-1,4-N-acetylglucosaminy	MGAT3
Mannosyl (alpha-1,3-)-glycoprotein beta-1,4-N-acetylglucosamin	MGAT4A
mannosyl (alpha-1,3-)-glycoprotein beta-1,4-N-acetylglucosamin	MGAT4B
mannosyl (alpha-1,3-)-glycoprotein beta-1,4-N-acetylglucosamin	MGAT4C
mannosyl (alpha-1,6-)-glycoprotein beta-1,6-N-acetyl-glucosamir	MGAT5
mannosyl (alpha-1,6-)-glycoprotein beta-1,6-N-acetyl-glucosamir	MGAT5B
N-acetylgalactosaminidase, alpha-	NAGA
N-acetylglucosamine-1-phosphodiester alpha-N-acetylglucosami	NAGPA
protein O-fucosyltransferase 1	POFUT1
protein O-fucosyltransferase 2	POFUT2
protein O-linked mannose beta1,2-N-acetylglucosaminyltransfera	POMGNT1
protein-O-mannosyltransferase 1	POMT1
protein-O-mannosyltransferase 2	POMT2
ST3 beta-galactoside alpha-2,3-sialyltransferase 1	ST3GAL1
ST3 beta-galactoside alpha-2,3-sialyltransferase 2	ST3GAL2
ST3 beta-galactoside alpha-2,3-sialyltransferase 3	ST3GAL3
ST3 beta-galactoside alpha-2,3-sialyltransferase 4	ST3GAL4
ST3 beta-galactoside alpha-2,3-sialyltransferase 5	ST3GAL5
ST3 beta-galactoside alpha-2,3-sialyltransferase 6	ST3GAL6
ST6 beta-galactosamide alpha-2,6-sialyltranferase 1	ST6GAL1
ST6 beta-galactosamide alpha-2,6-sialyltranferase 2	ST6GAL2
ST6 (alpha-N-acetyl-neuraminyl-2,3-beta-galactosyl-1,3)-N-acety	ST6GALNAC1
ST6 (alpha-N-acetyl-neuraminyl-2,3-beta-galactosyl-1,3)-N-acety	ST6GALNAC2
ST6 (alpha-N-acetyl-neuraminyl-2,3-beta-galactosyl-1,3)-N-acety	ST6GALNAC3
ST6 (alpha-N-acetyl-neuraminyl-2,3-beta-galactosyl-1,3)-N-acety	ST6GALNAC4
ST6 (alpha-N-acetyl-neuraminyl-2,3-beta-galactosyl-1,3)-N-acety	ST6GALNAC5
ST6 (alpha-N-acetyl-neuraminyl-2,3-beta-galactosyl-1,3)-N-acety	ST6GALNAC6
ST8 alpha-N-acetyl-neuraminide alpha-2,8-sialyltransferase 1	ST8SIA1
ST8 alpha-N-acetyl-neuraminide alpha-2,8-sialyltransferase 2	ST8SIA2
ST8 alpha-N-acetyl-neuraminide alpha-2,8-sialyltransferase 3	ST8SIA3
ST8 alpha-N-acetyl-neuraminide alpha-2,8-sialyltransferase 4	ST8SIA4
ST8 alpha-N-acetyl-neuraminide alpha-2,8-sialyltransferase 5	ST8SIA5
ST8 alpha-N-acetyl-neuraminide alpha-2,8-sialyltransferase 6	ST8SIA6
Williams-Beuren syndrome chromosome region 17	WBSCR17
xylosyltransferase I	XYLT1

xylosyltransferase II	XYLT2
dolichyl-phosphate mannosyltransferase polypeptide 1, catalytic	DPM1
dolichyl-phosphate mannosyltransferase polypeptide 2, regulato	DPM2
dolichyl-phosphate mannosyltransferase polypeptide 3	DPM3
glycosylphosphatidylinositol specific phospholipase D1	GPLD1
phosphatidylinositol glycan anchor biosynthesis, class A (paroxy	PIGA
phosphatidylinositol glycan anchor biosynthesis, class B	PIGB
phosphatidylinositol glycan anchor biosynthesis, class C	PIGC
phosphatidylinositol glycan anchor biosynthesis, class F	PIGF
phosphatidylinositol glycan anchor biosynthesis, class G	PIGG
phosphatidylinositol glycan anchor biosynthesis, class H	PIGH
phosphatidylinositol glycan anchor biosynthesis, class K	PIGK
phosphatidylinositol glycan anchor biosynthesis, class L	PIGL
phosphatidylinositol glycan anchor biosynthesis, class M	PIGM
phosphatidylinositol glycan anchor biosynthesis, class N	PIGN
phosphatidylinositol glycan anchor biosynthesis, class O	PIGO
phosphatidylinositol glycan anchor biosynthesis, class P	PIGP
phosphatidylinositol glycan anchor biosynthesis, class Q	PIGQ
phosphatidylinositol glycan anchor biosynthesis, class S	PIGS
phosphatidylinositol glycan anchor biosynthesis, class T	PIGT
phosphatidylinositol glycan anchor biosynthesis, class U	PIGU
phosphatidylinositol glycan anchor biosynthesis, class V	PIGV
phosphatidylinositol glycan anchor biosynthesis, class W	PIGW
phosphatidylinositol glycan anchor biosynthesis, class X	PIGX
phosphatidylinositol glycan anchor biosynthesis, class Y	PIGY
phosphatidylinositol glycan anchor biosynthesis, class Z	PIGZ
aspartylglucosaminidase	AGA
ER degradation enhancer, mannosidase alpha-like 1	EDEM1
ER degradation enhancer, mannosidase alpha-like 2	EDEM2
ER degradation enhancer, mannosidase alpha-like 3	EDEM3
endo-beta-N-acetylglucosaminidase	FLJ21865
fucosidase, alpha-L- 1, tissue	FUCA1
fucosidase, alpha-L- 2, plasma	FUCA2
galactosamine (N-acetyl)-6-sulfate sulfatase (Morquio syndrome	GALNS
galactosidase, alpha	GLA
galactosidase, beta 1	GLB1
galactosidase, beta 1-like	GLB1L
galactosidase, beta 2-like	GLB1L2
galactosidase, beta 1 like 3	GLB1L3
glucosamine (N-acetyl)-6-sulfatase (Sanfilippo disease IIID)	GNS
glucuronidase, beta	GUSB
hexosaminidase A (alpha polypeptide)	HEXA
hexosaminidase B (beta polypeptide)	HEXB
hexosaminidase (glycosyl hydrolase family 20, catalytic domain)	HEXDC
hyaluronoglucosaminidase 1	HYAL1
hyaluronoglucosaminidase 2	HYAL2
hyaluronoglucosaminidase 3	HYAL3
hyaluronoglucosaminidase 4	HYAL4

iduronate 2-sulfatase (Hunter syndrome)	IDS
iduronidase, alpha-L-	IDUA
lysozyme G-like 1	LYG1
lysozyme (renal amyloidosis)	LYZ
lysozyme-like 1	LYZL1
lysozyme-like 2	LYZL2
lysozyme-like 4	LYZL4
lysozyme-like 6	LYZL6
mannosidase, alpha, class 1A, member 1	MAN1A1
mannosidase, alpha, class 1A, member 2	MAN1A2
mannosidase, alpha, class 1B, member 1	MAN1B1
mannosidase, alpha, class 1C, member 1	MAN1C1
mannosidase, alpha, class 2A, member 1	MAN2A1
mannosidase, alpha, class 2A, member 2	MAN2A2
mannosidase, alpha, class 2B, member 1	MAN2B1
mannosidase, alpha, class 2B, member 2	MAN2B2
mannosidase, alpha, class 2C, member 1	MAN2C1
mannosidase, beta A, lysosomal	MANBA
mannosidase, beta A, lysosomal-like	MANBAL
mannosidase, endo-alpha	MANEA
mannosidase, endo-alpha-like	MANEAL
N-acetylglucosaminidase, alpha- (Sanfilippo disease IIIB)	NAGLU
sialidase 1 (lysosomal sialidase)	NEU1
sialidase 2 (cytosolic sialidase)	NEU2
sialidase 3 (membrane sialidase)	NEU3
sialidase 4	NEU4
chondroitin polymerizing factor	CHPF
chondroitin sulfate glucuronyltransferase	CHPF2
carbohydrate (keratan sulfate Gal-6) sulfotransferase 1	CHST1
carbohydrate sulfotransferase 10	CHST10
carbohydrate (chondroitin 4) sulfotransferase 11	CHST11
carbohydrate (chondroitin 4) sulfotransferase 12	CHST12
carbohydrate (chondroitin 4) sulfotransferase 13	CHST13
carbohydrate (N-acetylgalactosamine 4-0) sulfotransferase 14	CHST14
carbohydrate (N-acetylglucosamine-6-O) sulfotransferase 2	CHST2
carbohydrate (chondroitin 6) sulfotransferase 3	CHST3
carbohydrate (N-acetylglucosamine 6-O) sulfotransferase 4	CHST4
carbohydrate (N-acetylglucosamine 6-O) sulfotransferase 5	CHST5
carbohydrate (N-acetylglucosamine 6-O) sulfotransferase 6	CHST6
carbohydrate (N-acetylglucosamine 6-O) sulfotransferase 7	CHST7
carbohydrate (N-acetylgalactosamine 4-0) sulfotransferase 8	CHST8
carbohydrate (N-acetylgalactosamine 4-0) sulfotransferase 9	CHST9
carbohydrate (chondroitin) synthase 1	CHSY1
chondroitin synthase-2	CHSY3
chondroitin sulfate N-acetylgalactosaminyltransferase 1	CSGALNACT1
chondroitin sulfate N-acetylgalactosaminyltransferase 2	CSGALNACT2
galactose-3-O-sulfotransferase 1	GAL3ST1
galactose-3-O-sulfotransferase 2	GAL3ST2

galactose-3-O-sulfotransferase 3	GAL3ST3
galactose-3-O-sulfotransferase 4	GAL3ST4
N-sulfoglucosamine sulfohydrolase (sulfamidase)	SGSH
sulfatase 1	SULF1
sulfatase 2	SULF2
uronyl-2-sulfotransferase	UST
aminomethyltransferase	AMT
Betaine-homocysteine methyltransferase	BHMT
betaine-homocysteine methyltransferase 2	BHMT2
dimethylglycine dehydrogenase	DMGDH
glycine cleavage system protein H (aminomethyl carrier)	GCSH
glycine dehydrogenase (decarboxylating)	GLDC
sarcosine dehydrogenase	SARDH
ADP-dependent glucokinase	ADPGK
aldolase A, fructose-bisphosphate	ALDOA
aldolase B, fructose-bisphosphate	ALDOB
aldolase C, fructose-bisphosphate	ALDOC
2,3-bisphosphoglycerate mutase	BPGM
dihydrolipoamide S-acetyltransferase (E2 component of pyruvat	DLAT
enolase 1, (alpha)	ENO1
enolase 2 (gamma, neuronal)	ENO2
enolase 3 (beta, muscle)	ENO3
glyceraldehyde-3-phosphate dehydrogenase	GAPDH
glyceraldehyde-3-phosphate dehydrogenase, spermatogenic	GAPDHS
glucokinase (hexokinase 4, maturity onset diabetes of the young	GCK
glucose phosphate isomerase	GPI
hexokinase 1	HK1
hexokinase 2	HK2
hexokinase 3 (white cell)	HK3
hexokinase domain containing 1	HKDC1
pyruvate dehydrogenase (lipoamide) alpha 1	PDHA1
pyruvate dehydrogenase (lipoamide) alpha 2	PDHA2
pyruvate dehydrogenase (lipoamide) beta	PDHB
pyruvate dehydrogenase complex, component X	PDHX
6-phosphofructo-2-kinase/fructose-2,6-biphosphatase 1	PFKFB1
6-phosphofructo-2-kinase/fructose-2,6-biphosphatase 2	PFKFB2
6-phosphofructo-2-kinase/fructose-2,6-biphosphatase 3	PFKFB3
6-phosphofructo-2-kinase/fructose-2,6-biphosphatase 4	PFKFB4
phosphofructokinase, liver	PFKL
phosphofructokinase, muscle	PFKM
phosphofructokinase, platelet	PFKP
phosphoglycerate mutase 1 (brain)	PGAM1
phosphoglycerate mutase 2 (muscle)	PGAM2
phosphoglycerate mutase family member 4	PGAM4
phosphoglycerate mutase family member 5	PGAM5
phosphoglycerate kinase 1	PGK1
phosphoglycerate kinase 2	PGK2
phosphoglucomutase 1	PGM1

phosphoglucomutase 2	PGM2
Phosphoglucomutase 2-like 1	PGM2L1
phosphoglucomutase 5	PGM5
pyruvate kinase, liver and RBC	PKLR
pyruvate kinase, muscle	PKM2
triosephosphate isomerase 1	TPI1
glyoxalase I	GLO1
glyoxalase domain containing 4	GLOD4
glyoxalase domain containing 5	GLOD5
hydroxyacid oxidase (glycolate oxidase) 1	HAO1
hydroxyacid oxidase 2 (long chain)	HAO2
exostoses (multiple) 1	EXT1
exostoses (multiple) 2	EXT2
exostoses (multiple) 3	EXT3
exostoses (multiple)-like 1	EXTL1
exostoses (multiple)-like 2	EXTL2
exostoses (multiple)-like 3	EXTL3
glucuronic acid epimerase	GLCE
heparan-alpha-glucosaminide N-acetyltransferase	HGSNAT
heparanase	HPSE
heparanase 2	HPSE2
heparan sulfate 2-O-sulfotransferase 1	HS2ST1
heparan sulfate (glucosamine) 3-O-sulfotransferase 1	HS3ST1
heparan sulfate (glucosamine) 3-O-sulfotransferase 2	HS3ST2
heparan sulfate (glucosamine) 3-O-sulfotransferase 3A1	HS3ST3A1
Heparan sulfate (glucosamine) 3-O-sulfotransferase 3B1	HS3ST3B1
heparan sulfate (glucosamine) 3-O-sulfotransferase 4	HS3ST4
heparan sulfate (glucosamine) 3-O-sulfotransferase 5	HS3ST5
heparan sulfate (glucosamine) 3-O-sulfotransferase 6	HS3ST6
Heparan sulfate 6-O-sulfotransferase 1	HS6ST1
heparan sulfate 6-O-sulfotransferase 2	HS6ST2
heparan sulfate 6-O-sulfotransferase 3	HS6ST3
N-deacetylase/N-sulfotransferase (heparan glucosaminyl) 1	NDST1
N-deacetylase/N-sulfotransferase (heparan glucosaminyl) 2	NDST2
N-deacetylase/N-sulfotransferase (heparan glucosaminyl) 3	NDST3
N-deacetylase/N-sulfotransferase (heparan glucosaminyl) 4	NDST4
amidohydrolase domain containing 1	AMDHD1
amidohydrolase domain containing 2	AMDHD2
formiminotransferase cyclodeaminase	FTCD
histidine ammonia-lyase	HAL
Histidine decarboxylase	HDC
histamine N-methyltransferase	HNMT
Urocanase domain containing 1	UROC1
arylalkylamine N-acetyltransferase	AANAT
angiotensin I converting enzyme (peptidyl-dipeptidase A) 1	ACE
angiotensin I converting enzyme (peptidyl-dipeptidase A) 2	ACE2
arachidonate 12-lipoxygenase	ALOX12
arachidonate 12-lipoxygenase, 12R type	ALOX12B

arachidonate 15-lipoxygenase	ALOX15
arachidonate 15-lipoxygenase, type B	ALOX15B
arachidonate 5-lipoxygenase	ALOX5
arachidonate 5-lipoxygenase-activating protein	ALOX5AP
arachidonate lipoxygenase 3	ALOXE3
amine oxidase (flavin containing) domain 1	AOF1
amine oxidase (flavin containing) domain 2	AOF2
acetylserotonin O-methyltransferase	ASMT
acetylserotonin O-methyltransferase-like	ASMTL
cytochrome P450, family 20, subfamily A, polypeptide 1	CYP20A1
deiodinase, iodothyronine, type I	DIO1
deiodinase, iodothyronine, type II	DIO2
deiodinase, iodothyronine, type III	DIO3
hydroxyprostaglandin dehydrogenase 15-(NAD)	HPGD
iodotyrosine deiodinase	IYD
leucyl/cystinyl aminopeptidase	LNPEP
leukotriene A4 hydrolase	LTA4H
leukotriene C4 synthase	LTC4S
membrane metallo-endopeptidase	MME
membrane metallo-endopeptidase-like 1	MMEL1
prostaglandin D2 synthase, hematopoietic	PGDS
prostaglandin D2 synthase 21kDa (brain)	PTGDS
prostaglandin E synthase	PTGES
prostaglandin E synthase 2	PTGES2
prostaglandin E synthase 3 (cytosolic)	PTGES3
prostaglandin I2 (prostacyclin) synthase	PTGIS
prostaglandin reductase 1	PTGR1
prostaglandin reductase 2	PTGR2
prostaglandin-endoperoxide synthase 1 (prostaglandin G/H synt	PTGS1
prostaglandin-endoperoxide synthase 2 (prostaglandin G/H synt	PTGS2
thromboxane A synthase 1 (platelet, cytochrome P450, family 5,	TBXAS1
thyroid peroxidase	TPO
CDP-diacylglycerol--inositol 3-phosphatidyltransferase (phospha	CDIPT
FIG4 homolog (S. cerevisiae)	FIG4
inositol(myo)-1(or 4)-monophosphatase 1	IMPA1
inositol(myo)-1(or 4)-monophosphatase 2	IMPA2
inositol monophosphatase domain containing 1	IMPAD1
inositol polyphosphate-1-phosphatase	INPP1
inositol polyphosphate-4-phosphatase, type I, 107kDa	INPP4A
inositol polyphosphate-4-phosphatase, type II, 105kDa	INPP4B
inositol polyphosphate-5-phosphatase, 40kDa	INPP5A
inositol polyphosphate-5-phosphatase, 75kDa	INPP5B
inositol polyphosphate-5-phosphatase, 145kDa	INPP5D
inositol polyphosphate-5-phosphatase, 72 kDa	INPP5E
inositol polyphosphate-5-phosphatase F	INPP5F
inositol polyphosphate-5-phosphatase J	INPP5J
inositol polyphosphate-5-phosphatase K	INPP5K
inositol polyphosphate phosphatase-like 1	INPPL1

inositol hexaphosphate kinase 1	IP6K1
inositol hexaphosphate kinase 2	IP6K2
inositol hexaphosphate kinase 3	IP6K3
inositol polyphosphate multikinase	IPMK
inositol 1,3,4,5,6-pentakisphosphate 2-kinase	IPPK
myo-inositol 1-phosphate synthase A1	ISYNA1
inositol 1,3,4-triphosphate 5/6 kinase	ITPK1
inositol 1,4,5-trisphosphate 3-kinase A	ITPKA
inositol 1,4,5-trisphosphate 3-kinase B	ITPKB
inositol 1,4,5-trisphosphate 3-kinase C	ITPKC
multiple inositol polyphosphate histidine phosphatase, 1	MINPP1
myo-inositol oxygenase	MIOX
GPI deacylase	PGAP1
phosphatidylinositol 4-kinase type 2 alpha	PI4K2A
Phosphatidylinositol 4-kinase type 2 beta	PI4K2B
phosphatidylinositol 4-kinase, catalytic, alpha	PI4KA
phosphatidylinositol 4-kinase, catalytic, beta	PI4KB
phosphoinositide-3-kinase, class 2, alpha polypeptide	PIK3C2A
phosphoinositide-3-kinase, class 2, beta polypeptide	PIK3C2B
phosphoinositide-3-kinase, class 2, gamma polypeptide	PIK3C2G
phosphoinositide-3-kinase, class 3	PIK3C3
phosphoinositide-3-kinase, catalytic, alpha polypeptide	PIK3CA
phosphoinositide-3-kinase, catalytic, beta polypeptide	PIK3CB
phosphoinositide-3-kinase, catalytic, delta polypeptide	PIK3CD
phosphoinositide-3-kinase, catalytic, gamma polypeptide	PIK3CG
phosphoinositide-3-kinase, regulatory subunit 1 (p85 alpha)	PIK3R1
phosphoinositide-3-kinase, regulatory subunit 2 (p85 beta)	PIK3R2
phosphoinositide-3-kinase, regulatory subunit 3 (p55, gamma)	PIK3R3
phosphoinositide-3-kinase, regulatory subunit 4, p150	PIK3R4
phosphoinositide-3-kinase, regulatory subunit 5, p101	PIK3R5
phosphoinositide-3-kinase, regulatory subunit 6	PIK3R6
phosphatidylinositol-5-phosphate 4-kinase, type II, alpha	PIP4K2A
phosphatidylinositol-5-phosphate 4-kinase, type II, beta	PIP4K2B
phosphatidylinositol-5-phosphate 4-kinase, type II, gamma	PIP4K2C
phosphatidylinositol-4-phosphate 5-kinase, type I, alpha	PIP5K1A
phosphatidylinositol-4-phosphate 5-kinase, type I, beta	PIP5K1B
phosphatidylinositol-4-phosphate 5-kinase, type I, gamma	PIP5K1C
phosphatidylinositol-3-phosphate/phosphatidylinositol 5-kinase, t	PIP5K3
phosphatidylinositol-4-phosphate 5-kinase-like 1	PIP5KL1
phosphatase and tensin homolog	PTEN
synaptojanin 1	SYNJ1
synaptojanin 2	SYNJ2
ATPase, class VI, type 11A	ATP11A
ATPase, class VI, type 11B	ATP11B
ATPase, class VI, type 11C	ATP11C
ATPase, H+/K+ transporting, nongastric, alpha polypeptide	ATP12A
ATPase, Na+/K+ transporting, alpha 1 polypeptide	ATP1A1
ATPase, Na+/K+ transporting, alpha 2 polypeptide	ATP1A2

ATPase, Na+/K+ transporting, alpha 3 polypeptide	ATP1A3
ATPase, Na+/K+ transporting, alpha 4 polypeptide	ATP1A4
ATPase, Na+/K+ transporting, beta 1 polypeptide	ATP1B1
ATPase, Na+/K+ transporting, beta 2 polypeptide	ATP1B2
ATPase, Na+/K+ transporting, beta 3 polypeptide	ATP1B3
ATPase, (Na+)/K+ transporting, beta 4 polypeptide	ATP1B4
ATPase, Ca++ transporting, cardiac muscle, fast twitch 1	ATP2A1
ATPase, Ca++ transporting, cardiac muscle, slow twitch 2	ATP2A2
ATPase, Ca++ transporting, ubiquitous	ATP2A3
ATPase, Ca++ transporting, plasma membrane 1	ATP2B1
ATPase, Ca++ transporting, plasma membrane 2	ATP2B2
ATPase, Ca++ transporting, plasma membrane 3	ATP2B3
ATPase, Ca++ transporting, plasma membrane 4	ATP2B4
ATPase, Ca++-sequestering	ATP2C1
ATPase, Ca++ transporting, type 2C, member 2	ATP2C2
ATPase, Mg++ transporting	ATP3
ATPase, H+/K+ exchanging, gastric, alpha polypeptide	ATP4A
ATPase, H+/K+ exchanging, beta polypeptide	ATP4B
ATPase, Cu++ transporting, alpha polypeptide	ATP7A
ATPase, Cu++ transporting, beta polypeptide	ATP7B
Bestrophin 1	BEST1
Bestrophin 2	BEST2
Bestrophin 3	BEST3
Bestrophin 4	BEST4
Bartter syndrome, infantile, with sensorineural deafness (Barttin)	BSND
Chromosome 14 open reading frame 68	C14orf68
Chromosome 20 open reading frame 59	C20orf59
Chromosome 6 open reading frame 192	C6orf192
calcium channel, voltage-dependent, P/Q type, alpha 1A subunit	CACNA1A
calcium channel, voltage-dependent, N type, alpha 1B subunit	CACNA1B
calcium channel, voltage-dependent, L type, alpha 1C subunit	CACNA1C
calcium channel, voltage-dependent, L type, alpha 1D subunit	CACNA1D
calcium channel, voltage-dependent, R type, alpha 1E subunit	CACNA1E
calcium channel, voltage-dependent, alpha 1F subunit	CACNA1F
calcium channel, voltage-dependent, T type, alpha 1G subunit	CACNA1G
calcium channel, voltage-dependent, T type, alpha 1H subunit	CACNA1H
calcium channel, voltage-dependent, T type, alpha 1I subunit	CACNA1I
calcium channel, voltage-dependent, L type, alpha 1S subunit	CACNA1S
calcium channel, voltage-dependent, alpha2/delta subunit 1	CACNA2D1
calcium channel, voltage-dependent, alpha 2/delta subunit 2	CACNA2D2
calcium channel, voltage-dependent, alpha2/delta subunit 3	CACNA2D3
calcium channel, voltage-dependent, alpha 2/delta subunit 4	CACNA2D4
calcium channel, voltage-dependent, beta 1 subunit	CACNB1
calcium channel, voltage-dependent, beta 2 subunit	CACNB2
calcium channel, voltage-dependent, beta 3 subunit	CACNB3
calcium channel, voltage-dependent, beta 4 subunit	CACNB4
calcium channel, voltage-dependent, gamma subunit 1	CACNG1
calcium channel, voltage-dependent, gamma subunit 2	CACNG2

calcium channel, voltage-dependent, gamma subunit 3	CACNG3
calcium channel, voltage-dependent, gamma subunit 4	CACNG4
calcium channel, voltage-dependent, gamma subunit 5	CACNG5
calcium channel, voltage-dependent, gamma subunit 6	CACNG6
calcium channel, voltage-dependent, gamma subunit 7	CACNG7
calcium channel, voltage-dependent, gamma subunit 8	CACNG8
cation channel, sperm associated 1	CATSPER1
cation channel, sperm associated 2	CATSPER2
cation channel, sperm associated 3	CATSPER3
cation channel, sperm-associated, beta	CATSPERB
Cystic fibrosis transmembrane conductance regulator (ATP-bind	CFTR
Cholinergic receptor, nicotinic, alpha 1 (muscle)	CHRNA1
Cholinergic receptor, nicotinic, alpha 10	CHRNA10
Cholinergic receptor, nicotinic, alpha 2 (neuronal)	CHRNA2
Cholinergic receptor, nicotinic, alpha 3	CHRNA3
Cholinergic receptor, nicotinic, alpha 4	CHRNA4
Cholinergic receptor, nicotinic, alpha 5	CHRNA5
Cholinergic receptor, nicotinic, alpha 6	CHRNA6
Cholinergic receptor, nicotinic, alpha 7	CHRNA7
Cholinergic receptor, nicotinic, alpha 9	CHRNA9
Cholinergic receptor, nicotinic, beta 1 (muscle)	CHRNB1
Cholinergic receptor, nicotinic, beta 2 (neuronal)	CHRNB2
Cholinergic receptor, nicotinic, beta 3	CHRNB3
Cholinergic receptor, nicotinic, beta 4	CHRNB4
Cholinergic receptor, nicotinic, delta	CHRND
Cholinergic receptor, nicotinic, epsilon	CHRNE
Cholinergic receptor, nicotinic, gamma	CHRNG
chloride channel calcium activated 1	CLCA1
chloride channel calcium activated 2	CLCA2
chloride channel calcium activated 3	CLCA3
chloride channel calcium activated 4	CLCA4
chloride channel CLIC-like 1	CLCC1
chloride channel 1	CLCN1
chloride channel 2	CLCN2
chloride channel 3	CLCN3
chloride channel 4-2	CLCN4
chloride channel 5	CLCN5
chloride channel 6	CLCN6
chloride channel 7	CLCN7
Chloride channel Ka	CLCNKA
chloride channel Kb	CLCNKB
chloride intracellular channel 1	CLIC1
chloride intracellular channel 2	CLIC2
chloride intracellular channel 3	CLIC3
chloride intracellular channel 4 (mitochondrial)	CLIC4
chloride intracellular channel 5	CLIC5
chloride intracellular channel 6	CLIC6
cyclic nucleotide gated channel alpha 1	CNGA1

Cyclic nucleotide gated channel alpha 2	CNGA2
cyclic nucleotide gated channel alpha 3	CNGA3
cyclic nucleotide gated channel alpha 4	CNGA4
cyclic nucleotide gated channel beta 1	CNGB1
cyclic nucleotide gated channel beta 3	CNGB3
Gamma-aminobutyric acid (GABA) A receptor, alpha 1	GABRA1
Gamma-aminobutyric acid (GABA) A receptor, alpha 2	GABRA2
Gamma-aminobutyric acid (GABA) A receptor, alpha 3	GABRA3
Gamma-aminobutyric acid (GABA) A receptor, alpha 4	GABRA4
Gamma-aminobutyric acid (GABA) A receptor, alpha 5	GABRA5
Gamma-aminobutyric acid (GABA) A receptor, alpha 6	GABRA6
Gamma-aminobutyric acid (GABA) A receptor, beta 1	GABRB1
Gamma-aminobutyric acid (GABA) A receptor, beta 2	GABRB2
Gamma-aminobutyric acid (GABA) A receptor, beta 3	GABRB3
Gamma-aminobutyric acid (GABA) A receptor, delta	GABRD
Gamma-aminobutyric acid (GABA) A receptor, epsilon	GABRE
Gamma-aminobutyric acid (GABA) A receptor, gamma 1	GABRG1
Gamma-aminobutyric acid (GABA) A receptor, gamma 2	GABRG2
Gamma-aminobutyric acid (GABA) A receptor, gamma 3	GABRG3
Gamma-aminobutyric acid (GABA) A receptor, pi	GABRP
Gamma-aminobutyric acid (GABA) receptor, theta	GABRQ
Gamma-aminobutyric acid (GABA) receptor, rho 1	GABRR1
Gamma-aminobutyric acid (GABA) receptor, rho 2	GABRR2
Gamma-aminobutyric acid (GABA) receptor, rho 3	GABRR3
Glycine receptor, alpha 1 (startle disease/hyperekplexia)	GLRA1
Glycine receptor, alpha 2	GLRA2
Glycine receptor, alpha 3	GLRA3
Glycine receptor, alpha 4	GLRA4
Glycine receptor, beta	GLRB
Glutamate receptor, ionotropic, AMPA 1	GRIA1
Glutamate receptor, ionotropic, AMPA 2	GRIA2
Glutamate receptor, ionotrophic, AMPA 3	GRIA3
Glutamate receptor, ionotrophic, AMPA 4	GRIA4
Glutamate receptor, ionotropic, delta 1	GRID1
Glutamate receptor, ionotropic, delta 2	GRID2
Glutamate receptor, ionotropic, kainate 1	GRIK1
Glutamate receptor, ionotropic, kainate 2	GRIK2
Glutamate receptor, ionotropic, kainate 3	GRIK3
Glutamate receptor, ionotropic, kainate 4	GRIK4
Glutamate receptor, ionotropic, kainate 5	GRIK5
Glutamate receptor, ionotropic, N-methyl D-aspartate 1	GRIN1
Glutamate receptor, ionotropic, N-methyl D-aspartate 2A	GRIN2A
Glutamate receptor, ionotropic, N-methyl D-aspartate 2B	GRIN2B
Glutamate receptor, ionotropic, N-methyl D-aspartate 2C	GRIN2C
Glutamate receptor, ionotropic, N-methyl D-aspartate 2D	GRIN2D
Glutamate receptor, ionotropic, N-methyl-D-aspartate 3A	GRIN3A
Glutamate receptor, ionotropic, N-methyl-D-aspartate 3B	GRIN3B
hyperpolarization activated cyclic nucleotide-gated potassium ch	HCN1

hyperpolarization activated cyclic nucleotide-gated potassium ch	HCN2
hyperpolarization activated cyclic nucleotide-gated potassium ch	HCN3
hyperpolarization activated cyclic nucleotide-gated potassium ch	HCN4
5-hydroxytryptamine (serotonin) receptor 3A	HTR3A
5-hydroxytryptamine (serotonin) receptor 3B	HTR3B
5-hydroxytryptamine (serotonin) receptor 3, family member C	HTR3C
5-hydroxytryptamine (serotonin) receptor 3 family member D	HTR3D
5-hydroxytryptamine (serotonin) receptor 3, family member E	HTR3E
Inositol 1,4,5-triphosphate receptor, type 1	ITPR1
inositol 1,4,5-triphosphate receptor, type 2	ITPR2
inositol 1,4,5-triphosphate receptor, type 3	ITPR3
potassium voltage-gated channel, shaker-related subfamily, mer	KCNA1
potassium voltage-gated channel, shaker-related subfamily, mer	KCNA10
potassium voltage-gated channel, shaker-related subfamily, mer	KCNA2
potassium voltage-gated channel, shaker-related subfamily, mer	KCNA3
potassium voltage-gated channel, shaker-related subfamily, mer	KCNA4
potassium voltage-gated channel, shaker-related subfamily, mer	KCNA5
potassium voltage-gated channel, shaker-related, subfamily, me	KCNA6
potassium voltage-gated channel, shaker-related subfamily, mer	KCNA7
potassium voltage-gated channel, shaker-related subfamily, beta	KCNAB1
potassium voltage-gated channel, shaker-related subfamily, beta	KCNAB2
potassium voltage-gated channel, shaker-related subfamily, beta	KCNAB3
potassium voltage gated channel, Shab-related subfamily, meml	KCNB1
potassium voltage gated channel, Shab-related subfamily, meml	KCNB2
potassium voltage gated channel, Shaw-related subfamily, mem	KCNC1
potassium voltage gated channel, Shaw-related subfamily, mem	KCNC2
potassium voltage gated channel, Shaw-related subfamily, mem	KCNC3
potassium voltage gated channel, Shaw-related subfamily, mem	KCNC4
potassium voltage-gated channel, Shal-related family, member 1	KCND1
potassium voltage-gated channel, Shal-related family, member 2	KCND2
potassium voltage-gated channel, Shal-related family, member 3	KCND3
potassium voltage-gated channel, Isk-related subfamily, membe	KCNE1
potassium voltage-gated channel, Isk-related family, member 1-l	KCNE1L
potassium voltage-gated channel, Isk-related subfamily, gene 2	KCNE2
potassium voltage-gated channel, Isk-related subfamily, gene 3	KCNE3
potassium voltage-gated channel, Isk-related subfamily, gene 4	KCNE4
potassium voltage-gated channel, subfamily F, member 1	KCNF1
potassium voltage-gated channel, subfamily G, member 1	KCNG1
potassium voltage-gated channel, subfamily G, member 2	KCNG2
potassium voltage-gated channel, subfamily G, member 3	KCNG3
potassium voltage-gated channel, subfamily G, member 4	KCNG4
potassium voltage-gated channel, subfamily H (eag-related), me	KCNH1
potassium voltage-gated channel, subfamily H (eag-related), me	KCNH2
potassium voltage-gated channel, subfamily H (eag-related), me	KCNH3
potassium voltage-gated channel, subfamily H (eag-related), me	KCNH4
potassium voltage-gated channel, subfamily H (eag-related), me	KCNH5
potassium voltage-gated channel, subfamily H (eag-related), me	KCNH6
potassium voltage-gated channel, subfamily H (eag-related), me	KCNH7

potassium voltage-gated channel, subfamily H (eag-related), me	KCNH8
potassium inwardly-rectifying channel, subfamily J, member 1	KCNJ1
potassium inwardly-rectifying channel, subfamily J, member 10	KCNJ10
potassium inwardly rectifying channel, subfamily J, member 11	KCNJ11
potassium inwardly-rectifying channel, subfamily J, member 12	KCNJ12
potassium inwardly-rectifying channel, subfamily J, member 13	KCNJ13
potassium inwardly-rectifying channel, subfamily J, member 14	KCNJ14
potassium inwardly-rectifying channel, subfamily J, member 15	KCNJ15
potassium inwardly-rectifying channel, subfamily J, member 16	KCNJ16
potassium inwardly-rectifying channel, subfamily J, member 2	KCNJ2
potassium inwardly-rectifying channel, subfamily J, member 3	KCNJ3
potassium inwardly-rectifying channel, subfamily J, member 4	KCNJ4
potassium inwardly-rectifying channel, subfamily J, member 5	KCNJ5
potassium inwardly-rectifying channel, subfamily J, member 6	KCNJ6
potassium inwardly-rectifying channel, subfamily J, member 8	KCNJ8
potassium inwardly-rectifying channel, subfamily J, member 9	KCNJ9
potassium channel, subfamily K, member 1	KCNK1
potassium channel, subfamily K, member 10	KCNK10
potassium channel, subfamily K, member 12	KCNK12
potassium channel, subfamily K, member 13	KCNK13
potassium channel, subfamily K, member 15	KCNK15
potassium channel, subfamily K, member 16	KCNK16
potassium channel, subfamily K, member 17	KCNK17
Potassium channel, subfamily K, member 18	KCNK18
potassium channel, subfamily K, member 2	KCNK2
potassium channel, subfamily K, member 3	KCNK3
potassium channel, subfamily K, member 4	KCNK4
potassium channel, subfamily K, member 5	KCNK5
potassium inwardly-rectifying channel, subfamily K, member 6	KCNK6
potassium channel, subfamily K, member 7	KCNK7
potassium channel, subfamily K, member 9	KCNK9
potassium large conductance calcium-activated channel, subfam	KCNMA1
potassium large conductance calcium-activated channel, subfam	KCNMB1
potassium large conductance calcium-activated channel, subfam	KCNMB2
potassium large conductance calcium-activated channel, subfam	KCNMB3
potassium large conductance calcium-activated channel, subfam	KCNMB4
potassium intermediate/small conductance calcium-activated cha	KCNN1
potassium intermediate/small conductance calcium-activated cha	KCNN2
potassium intermediate/small conductance calcium-activated cha	KCNN3
potassium intermediate/small conductance calcium-activated cha	KCNN4
potassium voltage-gated channel, subfamily Q, member 1	KCNQ1
potassium voltage-gated channel, subfamily Q, member 2	KCNQ2
potassium voltage-gated channel, subfamily Q, member 3	KCNQ3
potassium voltage-gated channel, subfamily Q, member 4	KCNQ4
potassium voltage-gated channel, subfamily Q, member 5	KCNQ5
K+ voltage-gated channel, subfamily S, 1	KCNS1
K+ voltage-gated channel, subfamily S, 2	KCNS2
potassium voltage-gated channel, delayed-rectifier, subfamily S,	KCNS3

potassium channel, subfamily T, member 1	KCNT1
potassium channel, subfamily T, member 2	KCNT2
potassium channel, subfamily U, member 1	KCNU1
potassium channel, subfamily V, member 1	KCNV1
potassium channel, subfamily V, member 2	KCNV2
potassium channel tetramerisation domain containing 1	KCTD1
potassium channel tetramerisation domain containing 10	KCTD10
potassium channel tetramerisation domain containing 11	KCTD11
potassium channel tetramerisation domain containing 12	KCTD12
potassium channel tetramerisation domain containing 13	KCTD13
potassium channel tetramerisation domain containing 14	KCTD14
potassium channel tetramerisation domain containing 15	KCTD15
potassium channel tetramerisation domain containing 16	KCTD16
potassium channel tetramerisation domain containing 17	KCTD17
potassium channel tetramerisation domain containing 18	KCTD18
potassium channel tetramerisation domain containing 19	KCTD19
potassium channel tetramerisation domain containing 2	KCTD2
potassium channel tetramerisation domain containing 20	KCTD20
potassium channel tetramerisation domain containing 21	KCTD21
potassium channel tetramerisation domain containing 3	KCTD3
potassium channel tetramerisation domain containing 4	KCTD4
potassium channel tetramerisation domain containing 5	KCTD5
potassium channel tetramerisation domain containing 6	KCTD6
potassium channel tetramerisation domain containing 7	KCTD7
potassium channel tetramerisation domain containing 8	KCTD8
potassium channel tetramerisation domain containing 9	KCTD9
purinergic receptor P2X, ligand-gated ion channel, 1	P2RX1
purinergic receptor P2X, ligand-gated ion channel, 2	P2RX2
purinergic receptor P2X, ligand-gated ion channel, 3	P2RX3
purinergic receptor P2X, ligand-gated ion channel, 4	P2RX4
purinergic receptor P2X, ligand-gated ion channel, 5	P2RX5
purinergic receptor P2X, ligand-gated ion channel, 6	P2RX6
purinergic receptor P2X, ligand-gated ion channel, 7	P2RX7
sodium channel, voltage-gated, type X, alpha	SCN10A
sodium channel, voltage-gated, type XI, alpha	SCN11A
sodium channel, voltage-gated, type I, alpha	SCN1A
sodium channel, voltage-gated, type I, beta	SCN1B
sodium channel, voltage-gated, type II, alpha 1	SCN2A
sodium channel, voltage-gated, type II, beta	SCN2B
sodium channel, voltage-gated, type III, alpha	SCN3A
sodium channel, voltage-gated, type III, beta	SCN3B
sodium channel, voltage-gated, type IV, alpha	SCN4A
sodium channel, voltage-gated, type IV, beta	SCN4B
sodium channel, voltage-gated, type V, alpha	SCN5A
sodium channel, voltage-gated, type VII, alpha	SCN7A
sodium channel, voltage-gated, type VIII, alpha	SCN8A
sodium channel, voltage-gated, type IX, alpha	SCN9A
sodium channel, nonvoltage-gated, type I, alpha	SCNN1A

sodium channel, nonvoltage-gated 1 beta	SCNN1B
sodium channel, nonvoltage-gated 1, delta	SCNN1D
sodium channel, nonvoltage-gated 1 gamma	SCNN1G
solute carrier family 11 (proton-coupled divalent metal ion transp	SLC11A1
solute carrier family 11 (proton-coupled divalent metal ion transp	SLC11A2
solute carrier family 12 (sodium/potassium/chloride transporters)	SLC12A1
solute carrier family 12, member 2	SLC12A2
solute carrier family 12, member 3	SLC12A3
solute carrier family 12, member 4	SLC12A4
solute carrier family 12, member 5	SLC12A5
solute carrier family 12, member 6	SLC12A6
solute carrier family 12, member 7	SLC12A7
solute carrier family 12 (potassium/chloride transporters), memb	SLC12A8
solute carrier family 12 (potassium/chloride transporters), memb	SLC12A9
solute carrier family 13 (sodium/sulphate symporters), member 1	SLC13A1
solute carrier family 13 (sodium-dependent dicarboxylate transp	SLC13A2
solute carrier family 13 (sodium-dependent dicarboxylate transp	SLC13A3
solute carrier family 13 (sodium/sulfate symporters), member 4	SLC13A4
solute carrier family 13 (sodium/sulfate symporters), member 5	SLC13A5
solute carrier family 24 (sodium/potassium/calcium exchanger),	SLC24A1
solute carrier family 24 (sodium/potassium/calcium exchanger),	SLC24A2
solute carrier family 24 (sodium/potassium/calcium exchanger),	SLC24A3
solute carrier family 24 (sodium/potassium/calcium exchanger),	SLC24A4
solute carrier family 24, member 5	SLC24A5
solute carrier family 24 (sodium/potassium/calcium exchanger),	SLC24A6
solute carrier family 30 (zinc transporter), member 1	SLC30A1
solute carrier family 30, member 10	SLC30A10
solute carrier family 30 (zinc transporter), member 2	SLC30A2
solute carrier family 30 (zinc transporter), member 3	SLC30A3
solute carrier family 30 (zinc transporter), member 4	SLC30A4
solute carrier family 30 (zinc transporter), member 5	SLC30A5
solute carrier family 30 (zinc transporter), member 6	SLC30A6
solute carrier family 30 (zinc transporter), member 7	SLC30A7
solute carrier family 30 (zinc transporter), member 8	SLC30A8
solute carrier family 30 (zinc transporter), member 9	SLC30A9
solute carrier family 39 (zinc transporter), member 1	SLC39A1
solute carrier family 39 (zinc transporter), member 10	SLC39A10
solute carrier family 39 (metal ion transporter), member 11	SLC39A11
solute carrier family 39 (metal ion transporter), member 12	SLC39A12
solute carrier family 39 (metal ion transporter), member 13	SLC39A13
solute carrier family 39 (zinc transporter), member 14	SLC39A14
solute carrier family 39 (zinc transporter), member 2	SLC39A2
solute carrier family 39 (zinc transporter), member 3	SLC39A3
solute carrier family 39 (zinc transporter), member 4	SLC39A4
solute carrier family 39 (metal ion transporter), member 5	SLC39A5
solute carrier family 39 (metal ion transporter), member 6	SLC39A6
solute carrier family 39, member 7	SLC39A7
solute carrier family 39 (metal ion transporter), member 8	SLC39A8

solute carrier family 39 (zinc transporter), member 9	SLC39A9
solute carrier family 3, member 1	SLC3A1
solute carrier family 3 (activators of dibasic and neutral amino ac	SLC3A2
solute carrier family 40 (iron-regulated transporter), member 1	SLC40A1
solute carrier family 41, member 1	SLC41A1
solute carrier family 41, member 2	SLC41A2
solute carrier family 41, member 3	SLC41A3
voltage-dependent anion channel 1	VDAC1
voltage-dependent anion channel 2	VDAC2
voltage-dependent anion channel 3	VDAC3
zinc activated ligand-gated ion channel	ZACN
hephaestin	HEPH
ligand-gated ion channel, zinc activated 1	LGICZ1
organic anion transporter LST-3b	LST-3TM12
magnesium transporter 1	MAGT1
Mucolipin 1	MCOLN1
Mucolipin 2	MCOLN2
Mucolipin 3	MCOLN3
methylmalonic aciduria (cobalamin deficiency) cblA type	MMAA
sodium leak channel, non-selective	NALCN
non imprinted in Prader-Willi/Angelman syndrome 1	NIPA1
Oculocutaneous albinism II (pink-eye dilution homolog, mouse)	OCA2
ORAI calcium release-activated calcium modulator 1	ORAI1
ORAI calcium release-activated calcium modulator 2	ORAI2
ORAI calcium release-activated calcium modulator 3	ORAI3
Polycystic kidney disease 1 (autosomal dominant)	PKD1
Polycystic kidney disease 2 (autosomal dominant)	PKD2
Polycystic kidney disease 2-like 1	PKD2L1
ryanodine receptor 1 (skeletal)	RYR1
ryanodine receptor 2 (cardiac)	RYR2
ryanodine receptor 3	RYR3
Solute carrier family 22, member 24	SLC22A24
Solute carrier family 22, member 25	SLC25A25
solute carrier family 31, member 1	SLC31A1
solute carrier family 31, member 2	SLC31A2
sarcolipin	SLN
transcobalamin I (vitamin B12 binding protein, R binder family)	TCN1
transcobalamin II; macrocytic anemia	TCN2
transferrin	TF
transient receptor potential cation channel, subfamily A, membe	TRPA1
transient receptor potential cation channel, subfamily C, membe	TRPC1
transient receptor potential cation channel, subfamily C, membe	TRPC3
transient receptor potential cation channel, subfamily C, membe	TRPC4
transient receptor potential cation channel, subfamily C, membe	TRPC5
transient receptor potential cation channel, subfamily C, membe	TRPC6
transient receptor potential cation channel, subfamily C, membe	TRPC7
transient receptor potential cation channel, subfamily M, membe	TRPM1
transient receptor potential cation channel, subfamily M, membe	TRPM2

transient receptor potential cation channel, subfamily M, membe	TRPM3
transient receptor potential cation channel, subfamily M, membe	TRPM4
transient receptor potential cation channel, subfamily M, membe	TRPM5
transient receptor potential cation channel, subfamily M, membe	TRPM6
transient receptor potential cation channel, subfamily M, membe	TRPM7
transient receptor potential cation channel, subfamily M, membe	TRPM8
transient receptor potential cation channel, subfamily V, membe	TRPV1
transient receptor potential cation channel, subfamily V, membe	TRPV2
transient receptor potential cation channel, subfamily V, membe	TRPV3
transient receptor potential cation channel, subfamily V, membe	TRPV4
transient receptor potential cation channel, subfamily V, membe	TRPV5
transient receptor potential cation channel, subfamily V, membe	TRPV6
Tweety homolog 1 (Drosophila)	TTYH1
Tweety homolog 2 (Drosophila)	TTYH2
Tweety homolog 3 (Drosophila)	TTYH3
3-hydroxybutyrate dehydrogenase, type 1	BDH1
3-hydroxybutyrate dehydrogenase, type 2	BDH2
3-oxoacid CoA transferase 1	OXCT1
3-oxoacid CoA transferase 2	OXCT2
ATP citrate lyase	ACLY
aconitase 1, soluble	ACO1
aconitase 2, mitochondrial	ACO2
citrate synthase	CS
D-2-hydroxyglutarate dehydrogenase	D2HGDH
dihydrolipoamide S-succinyltransferase (E2 component of 2-oxo	DLST
fumarate hydratase	FH
holocytochrome c synthase (cytochrome c heme-lyase)	HCCS
isocitrate dehydrogenase 1 (NADP+), soluble	IDH1
isocitrate dehydrogenase 2 (NADP+), mitochondrial	IDH2
isocitrate dehydrogenase 3 (NAD+) alpha	IDH3A
isocitrate dehydrogenase 3 (NAD+) beta	IDH3B
isocitrate dehydrogenase 3 (NAD+) gamma	IDH3G
L-2-hydroxyglutarate dehydrogenase	L2HGDH
malate dehydrogenase 1, NAD (soluble)	MDH1
malate dehydrogenase 1B, NAD (soluble)	MDH1B
malate dehydrogenase 2, NAD (mitochondrial)	MDH2
malic enzyme 1, NADP(+)-dependent, cytosolic	ME1
malic enzyme 2, NAD(+)-dependent, mitochondrial	ME2
malic enzyme 3, NADP(+)-dependent, mitochondrial	ME3
oxoglutarate (alpha-ketoglutarate) dehydrogenase (lipoamide)	OGDH
oxoglutarate dehydrogenase-like	OGDHL
pyruvate carboxylase	PC
succinate-CoA ligase, ADP-forming, beta subunit	SUCLA2
succinate-CoA ligase, alpha subunit	SUCLG1
succinate-CoA ligase, GDP-forming, beta subunit	SUCLG2
aminoadipate-semialdehyde dehydrogenase	AASDH
aminoadipate-semialdehyde dehydrogenase-phosphopantethein	AASDHPPT
aminoadipate-semialdehyde synthase	AASS

pipecolic acid oxidase	PIPOX
1-acylglycerol-3-phosphate O-acyltransferase 1 (lysophosphatidi	AGPAT1
1-acylglycerol-3-phosphate O-acyltransferase 2 (lysophosphatidi	AGPAT2
1-acylglycerol-3-phosphate O-acyltransferase 3	AGPAT3
1-acylglycerol-3-phosphate O-acyltransferase 4 (lysophosphatidi	AGPAT4
1-acylglycerol-3-phosphate O-acyltransferase 5 (lysophosphatidi	AGPAT5
1-acylglycerol-3-phosphate O-acyltransferase 6 (lysophosphatidi	AGPAT6
1-acylglycerol-3-phosphate O-acyltransferase 9	AGPAT9
CDP-diacylglycerol synthase (phosphatidate cytidylyltransferase	CDS1
CDP-diacylglycerol synthase (phosphatidate cytidylyltransferase	CDS2
choline/ethanolamine phosphotransferase 1	CEPT1
choline kinase alpha	CHKA
choline kinase beta	CHKB
choline phosphotransferase 1	CHPT1
Charcot-Leyden crystal protein	CLC
cardiolipin synthase 1	CRLS1
carnitine O-octanoyltransferase	CROT
diacylglycerol lipase, alpha	DAGLA
diacylglycerol lipase, beta	DAGLB
diacylglycerol kinase, alpha 80kDa	DGKA
diacylglycerol kinase, beta 90kDa	DGKB
diacylglycerol kinase, delta 130kDa	DGKD
diacylglycerol kinase, epsilon 64kDa	DGKE
diacylglycerol kinase, gamma 90kDa	DGKG
diacylglycerol kinase, eta	DGKH
diacylglycerol kinase, iota	DGKI
diacylglycerol kinase, kappa	DGKK
diacylglycerol kinase, theta 110kDa	DGKQ
diacylglycerol kinase, zeta 104kDa	DGKZ
ethanolamine kinase 1	ETNK1
ethanolamine kinase 2	ETNK2
glycerophosphodiester phosphodiesterase 1	GDE1
glycerol kinase	GK
glycerol kinase 2	GK2
glycerol kinase 5 (putative)	GK5
glyceronephosphate O-acyltransferase	GNPAT
glycerol-3-phosphate acyltransferase, mitochondrial	GPAM
glycerol-3-phosphate dehydrogenase 1 (soluble)	GPD1
glycerol-3-phosphate dehydrogenase 1-like	GPD1L
glycerol-3-phosphate dehydrogenase 2 (mitochondrial)	GPD2
lysophosphatidylcholine acyltransferase 1	LPCAT1
lysophosphatidylcholine acyltransferase 2	LPCAT2
membrane bound O-acyltransferase domain containing 5	LPCAT3
lysophosphatidylcholine acyltransferase 4	LPCAT4
lysophosphatidylglycerol acyltransferase 1	LPGAT1
lipin 1	LPIN1
lipin 2	LPIN2
lipin 3	LPIN3

lysophospholipase I	LYPLA1
lysophospholipase II	LYPLA2
lysophospholipase-like 1	LYPLAL1
membrane bound O-acyltransferase domain containing 1	MBOAT1
membrane bound O-acyltransferase domain containing 2	MBOAT2
membrane bound O-acyltransferase domain containing 4	MBOAT4
membrane bound O-acyltransferase domain containing 7	MBOAT7
monoacylglycerol O-acyltransferase 1	MOGAT1
monoacylglycerol O-acyltransferase 2	MOGAT2
monoacylglycerol O-acyltransferase 3	MOGAT3
N-acyl-phosphatidylethanolamine-hydrolyzing phospholipase D	NAPE-PLD
phosphatidic acid phosphatase type 2	PAP2D
phosphate cytidylyltransferase 1, choline, alpha	PCYT1A
phosphate cytidylyltransferase 1, choline, beta	PCYT1B
phosphate cytidylyltransferase 2, ethanolamine	PCYT2
phosphatidylethanolamine N-methyltransferase	PEMT
phosphatidylglycerophosphate synthase 1	PGS1
phosphatase orphan 1	PHOSPHO1
phosphatidylserine decarboxylase	PISD
phospholipase A1 member A	PLA1A
phospholipase A2, group X	PLA2G10
phospholipase A2, group XIIA	PLA2G12A
phospholipase A2, group XIIB	PLA2G12B
phospholipase A2, group XV	PLA2G15
phospholipase A2, group XVI	PLA2G16
phospholipase A2, group IB (pancreas)	PLA2G1B
phospholipase A2, group IIA (platelets, synovial fluid)	PLA2G2A
phospholipase A2, group IIC	PLA2G2C
phospholipase A2, group IID	PLA2G2D
phospholipase A2, group IIE	PLA2G2E
phospholipase A2, group IIF	PLA2G2F
phospholipase A2, group III	PLA2G3
phospholipase A2, group IVA (cytosolic, calcium-dependent)	PLA2G4A
phospholipase A2, group IVB (cytosolic)	PLA2G4B
phospholipase A2, group IVC (cytosolic, calcium-independent)	PLA2G4C
phospholipase A2, group IVD (cytosolic)	PLA2G4D
phospholipase A2, group IVE	PLA2G4E
Phospholipase A2, group IVF	PLA2G4F
phospholipase A2, group V	PLA2G5
phospholipase A2, group VI (cytosolic, calcium-independent)	PLA2G6
phospholipase A2, group VII (platelet-activating factor acetylhyd	PLA2G7
phospholipase B1	PLB1
phospholipase C, beta 1 (phosphoinositide-specific)	PLCB1
phospholipase C, beta 2	PLCB2
phospholipase C, beta 3 (phosphatidylinositol-specific)	PLCB3
phospholipase C, beta 4	PLCB4
phospholipase C, delta 1	PLCD1
phospholipase C, delta 3	PLCD3

phospholipase C, delta 4	PLCD4
Phospholipase C, epsilon 1	PLCE1
phospholipase C, gamma 1	PLCG1
Phospholipase C, gamma 2 (phosphatidylinositol-specific)	PLCG2
phospholipase C, eta 1	PLCH1
phospholipase C, eta 2	PLCH2
phospholipase C-like 1	PLCL1
phospholipase C-like 2	PLCL2
phosphatidylinositol-specific phospholipase C, X domain contain	PLCXD1
phosphatidylinositol-specific phospholipase C, X domain contain	PLCXD2
phosphatidylinositol-specific phospholipase C, X domain contain	PLCXD3
phospholipase C, zeta 1	PLCZ1
phospholipase D1, phosphatidylcholine-specific	PLD1
phospholipase D2	PLD2
phospholipase D family, member 3	PLD3
phospholipase D family, member 4	PLD4
phospholipase D family, member 5	PLD5
phospholipase D family, member 6	PLD6
phosphatidic acid phosphatase type 2A	PPAP2A
phosphatidic acid phosphatase type 2B	PPAP2B
phosphatidic acid phosphatase type 2C	PPAP2C
phosphatidic acid phosphatase type 2 domain containing 1A	PPAPDC1A
phosphatidic acid phosphatase type 2 domain containing 1B	PPAPDC1B
phosphatidic acid phosphatase type 2 domain containing 2	PPAPDC2
phosphatidic acid phosphatase type 2 domain containing 3	PPAPDC3
plasticity-related gene 2	PRG2
phosphatidylserine synthase 1	PTDSS1
phosphatidylserine synthase 2	PTDSS2
methionine adenosyltransferase I, alpha	MAT1A
methionine adenosyltransferase II, alpha	MAT2A
methionine adenosyltransferase II, beta	MAT2B
methionine sulfoxide reductase A	MSRA
methionine sulfoxide reductase B2	MSRB2
methionine sulfoxide reductase B3	MSRB3
5-methyltetrahydrofolate-homocysteine methyltransferase	MTR
selenoprotein X, 1	SEPX1
3-hydroxy-3-methylglutaryl-Coenzyme A reductase	HMGCR
3-hydroxy-3-methylglutaryl-Coenzyme A synthase 1 (soluble)	HMGCS1
3-hydroxy-3-methylglutaryl-Coenzyme A synthase 2 (mitochondr	HMGCS2
isopentenyl-diphosphate delta isomerase 1	IDI1
isopentenyl-diphosphate delta isomerase 2	IDI2
mevalonate (diphospho) decarboxylase	MVD
mevalonate kinase (mevalonic aciduria)	MVK
phosphomevalonate kinase	PMVK
abhydrolase domain containing 1	ABHD1
abhydrolase domain containing 10	ABHD10
abhydrolase domain containing 11	ABHD11
abhydrolase domain containing 12	ABHD12

abhydrolase domain containing 12B	ABHD12B
abhydrolase domain containing 13	ABHD13
abhydrolase domain containing 14A	ABHD14A
abhydrolase domain containing 14B	ABHD14B
abhydrolase domain containing 2	ABHD2
abhydrolase domain containing 3	ABHD3
abhydrolase domain containing 4	ABHD4
Abhydrolase domain containing 5	ABHD5
abhydrolase domain containing 6	ABHD6
abhydrolase domain containing 7	ABHD7
abhydrolase domain containing 8	ABHD8
abhydrolase domain containing 9	ABHD9
amiloride binding protein 1 (amine oxidase (copper-containing))	ABP1
acylphosphatase 1, erythrocyte (common) type	ACYP1
acylphosphatase 2, muscle type	ACYP2
alcohol dehydrogenase 1A (class I), alpha polypeptide	ADH1A
Alcohol dehydrogenase 1B (class I), beta polypeptide	ADH1B
alcohol dehydrogenase 1C (class I), gamma polypeptide	ADH1C
alcohol dehydrogenase 4 (class II), pi polypeptide	ADH4
alcohol dehydrogenase 5 (class III), chi polypeptide	ADH5
alcohol dehydrogenase 6 (class V)	ADH6
alcohol dehydrogenase 7 (class IV), mu or sigma polypeptide	ADH7
alcohol dehydrogenase, iron containing, 1	ADHFE1
Aldo-keto reductase family 1, member A1 (aldehyde reductase)	AKR1A1
aldo-keto reductase family 1, member B1 (aldose reductase)	AKR1B1
aldo-keto reductase family 1, member B10 (aldose reductase)	AKR1B10
aldo-keto reductase family 1, member C1 (dihydrodiol dehydroge	AKR1C1
Aldo-keto reductase family 1, member C2 (dihydrodiol dehydrog	AKR1C2
aldo-keto reductase family 1, member C3 (3-alpha hydroxysteroi	AKR1C3
aldo-keto reductase family 1, member C-like 2	AKR1CL2
aldo-keto reductase family 7, member A2 (aflatoxin aldehyde rec	AKR7A2
aldo-keto reductase family 7, member A3 (aflatoxin aldehyde rec	AKR7A3
aldehyde dehydrogenase 16 family, member A1	ALDH16A1
aldehyde dehydrogenase 1 family, member A1	ALDH1A1
aldehyde dehydrogenase 1 family, member A2	ALDH1A2
aldehyde dehydrogenase 1 family, member A3	ALDH1A3
aldehyde dehydrogenase 1 family, member B1	ALDH1B1
aldehyde dehydrogenase 1 family, member L1	ALDH1L1
aldehyde dehydrogenase 1 family, member L2	ALDH1L2
aldehyde dehydrogenase 2 family (mitochondrial)	ALDH2
aldehyde dehydrogenase 3 family, memberA1	ALDH3A1
aldehyde dehydrogenase 3 family, member A2	ALDH3A2
aldehyde dehydrogenase 3 family, member B1	ALDH3B1
aldehyde dehydrogenase 3 family, member B2	ALDH3B2
aldehyde dehydrogenase 6 family, member A1	ALDH6A1
aldehyde dehydrogenase 7 family, member A1	ALDH7A1
aldehyde dehydrogenase 8 family, member A1	ALDH8A1
aldehyde dehydrogenase 9 family, member A1	ALDH9A1

amine oxidase, copper containing 2 (retina-specific)	AOC2
amine oxidase, copper containing 3 (vascular adhesion protein 1	AOC3
Arylsulfatase A	ARSA
arylsulfatase B	ARSB
arylsulfatase D	ARSD
arylsulfatase E (chondrodysplasia punctata 1)	ARSE
arylsulfatase F	ARSF
arylsulfatase G	ARSG
arylsulfatase H	ARSH
arylsulfatase family, member I	ARSI
arylsulfatase family, member J	ARSJ
Arylsulfatase family, member K	ARSK
AU RNA binding protein/enoyl-Coenzyme A hydratase	AUH
carbonic anhydrase I	CA1
carbonic anhydrase X	CA10
carbonic anhydrase XI	CA11
carbonic anhydrase XII	CA12
carbonic anhydrase XIII	CA13
carbonic anhydrase XIV	CA14
carbonic anhydrase II	CA2
carbonic anhydrase III, muscle specific	CA3
carbonic anhydrase IV	CA4
carbonic anhydrase VA, mitochondrial	CA5A
carbonic anhydrase VB, mitochondrial	CA5B
carbonic anhydrase VI	CA6
carbonic anhydrase VII	CA7
carbonic anhydrase VIII	CA8
carbonic anhydrase IX	CA9
carbonyl reductase 1	CBR1
carbonyl reductase 3	CBR3
carbonyl reductase 4	CBR4
dihydrolipoamide dehydrogenase	DLD
gamma-glutamyl hydrolase (conjugase, folylpolygammaglutamyl	GGH
gamma-glutamyltransferase 1	GGT1
gamma-glutamyltransferase 2	GGT2
gamma-glutamyltransferase 5	GGT5
gamma-glutamyltransferase 6 homolog (rat)	GGT6
gamma-glutamyltransferase 7	GGT7
gamma-glutamyltransferase-like activity 4	GGTLC1
gamma-glutamyltransferase light chain 2	GGTLC2
glutamic-oxaloacetic transaminase 1, soluble (aspartate aminotr	GOT1
glutamic-oxaloacetic transaminase 1-like 1	GOT1L1
glutamic-oxaloacetic transaminase 2, mitochondrial (aspartate a	GOT2
glutathione transferase zeta 1 (maleylacetoacetate isomerase)	GSTZ1
3-hydroxymethyl-3-methylglutaryl-Coenzyme A lyase (hydroxym	HMGCL
3-hydroxymethyl-3-methylglutaryl-Coenzyme A lyase-like 1	HMGCLL1
plasticity related gene 1	LPPR4
MAK10 homolog, amino-acid N-acetyltransferase subunit (S. ce	MAK10

monoamine oxidase A	MAOA
Monoamine oxidase B	MAOB
N-acetyltransferase 1 (arylamine N-acetyltransferase)	NAT1
N-acetyltransferase 10	NAT10
N-acetyltransferase 11	NAT11
N-acetyltransferase 12	NAT12
N-acetyltransferase 13	NAT13
N-acetyltransferase 14	NAT14
N-acetyltransferase 15	NAT15
N-acetyltransferase 2 (arylamine N-acetyltransferase)	NAT2
N-acetyltransferase 5	NAT5
N-acetyltransferase 6	NAT6
N-acetyltransferase 8	NAT8
N-acetyltransferase 8B	NAT8B
N-acetyltransferase 8-like	NAT8L
N-acetyltransferase 9	NAT9
P450 (cytochrome) oxidoreductase	POR
zinc binding alcohol dehydrogenase, domain containing 2	ZADH2
bone marrow stromal cell antigen 1	BST1
Chromosome 9 ORF 95	C9ORF95
dual oxidase 1	DUOX1
dual oxidase 2	DUOX2
NAD kinase	NADK
NAD synthetase 1	NADSYN1
nicotinate phosphoribosyltransferase domain containing 1	NAPRT1
nicotinamide nucleotide adenylyltransferase 1	NMNAT1
nicotinamide nucleotide adenylyltransferase 2	NMNAT2
nicotinamide nucleotide adenylyltransferase 3	NMNAT3
nicotinamide N-methyltransferase	NNMT
nicotinamide nucleotide transhydrogenase	NNT
NADPH oxidase 1	NOX1
NADPH oxidase 3	NOX3
NADPH oxidase 4	NOX4
NADPH oxidase, EF-hand calcium binding domain 5	NOX5
NAD(P)H dehydrogenase, quinone 1	NQO1
NAD(P)H dehydrogenase, quinone 2	NQO2
quinolinate phosphoribosyltransferase (nicotinate-nucleotide pyr	QPRT
4-aminobutyrate aminotransferase	ABAT
acetylcholinesterase (Yt blood group)	ACHE
choline acetyltransferase	CHAT
choline dehydrogenase	CHDH
catechol-O-methyltransferase	COMT
catechol-O-methyltransferase domain containing 1	COMTD1
dopamine beta-hydroxylase (dopamine beta-monooxygenase)	DBH
dopa decarboxylase (aromatic L-amino acid decarboxylase)	DDC
N-acetylated alpha-linked acidic dipeptidase 2	NAALAD2
N-acetylated alpha-linked acidic dipeptidase-like 1	NAALADL1
N-acetylated alpha-linked acidic dipeptidase-like 2	NAALADL2

phenylethanolamine N-methyltransferase	PNMT
serine racemase	SRR
tyrosine hydroxylase	TH
tryptophan hydroxylase 1 (tryptophan 5-monooxygenase)	TPH1
tryptophan hydroxylase 2	TPH2
3'(2'), 5'-bisphosphate nucleotidase 1	BPNT1
calcium activated nucleotidase 1	CANT1
deoxycytidine kinase	DCK
ectonucleotide pyrophosphatase/phosphodiesterase 1	ENPP1
ectonucleotide pyrophosphatase/phosphodiesterase 2 (autotaxir	ENPP2
ectonucleotide pyrophosphatase/phosphodiesterase 3	ENPP3
ectonucleotide pyrophosphatase/phosphodiesterase 4 (putative	ENPP4
ectonucleotide pyrophosphatase/phosphodiesterase 5 (putative	ENPP5
ectonucleotide pyrophosphatase/phosphodiesterase 6	ENPP6
ectonucleotide pyrophosphatase/phosphodiesterase 7	ENPP7
ectonucleoside triphosphate diphosphohydrolase 1	ENTPD1
ectonucleoside triphosphate diphosphohydrolase 2	ENTPD2
ectonucleoside triphosphate diphosphohydrolase 3	ENTPD3
ectonucleoside triphosphate diphosphohydrolase 4	ENTPD4
ectonucleoside triphosphate diphosphohydrolase 5	ENTPD5
ectonucleoside triphosphate diphosphohydrolase 6 (putative fun	ENTPD6
ectonucleoside triphosphate diphosphohydrolase 7	ENTPD7
ectonucleoside triphosphate diphosphohydrolase 8	ENTPD8
inosine triphosphatase (nucleoside triphosphate pyrophosphatas	ITPA
non-metastatic cells 1, protein (NM23A) expressed in	NME1
non-metastatic cells 2, protein (NM23A) expressed in	NME2
non-metastatic cells 3, protein expressed in	NME3
non-metastatic cells 4, protein expressed in	NME4
non-metastatic cells 5, protein expressed in (nucleoside-diphosp	NME5
non-metastatic cells 6, protein expressed in (nucleoside-diphosp	NME6
non-metastatic cells 7, protein expressed in (nucleoside-diphosp	NME7
5', 3'-nucleotidase, cytosolic	NT5C
5'-nucleotidase, cytosolic IA	NT5C1A
5'-nucleotidase, cytosolic IB	NT5C1B
5'-nucleotidase, cytosolic II	NT5C2
5'-nucleotidase, cytosolic III	NT5C3
5'-nucleotidase, cytosolic III-like	NT5C3L
5'-nucleotidase domain containing 1	NT5DC1
5'-nucleotidase domain containing 2	NT5DC2
5'-nucleotidase domain containing 3	NT5DC3
5'-nucleotidase domain containing 4	NT5DC4
5'-nucleotidase, ecto (CD73)	NT5E
5',3'-nucleotidase, mitochondrial	NT5M
nudix (nucleoside diphosphate linked moiety X)-type motif 1	NUDT1
nudix (nucleoside diphosphate linked moiety X)-type motif 10	NUDT10
nudix (nucleoside diphosphate linked moiety X)-type motif 11	NUDT11
nudix (nucleoside diphosphate linked moiety X)-type motif 12	NUDT12
nudix (nucleoside diphosphate linked moiety X)-type motif 13	NUDT13

Nudix (nucleoside diphosphate linked moiety X)-type motif 14	NUDT14
nudix (nucleoside diphosphate linked moiety X)-type motif 15	NUDT15
nudix (nucleoside diphosphate linked moiety X)-type motif 16	NUDT16
nudix (nucleoside diphosphate linked moiety X)-type motif 16-lik	NUDT16L1
nudix (nucleoside diphosphate linked moiety X)-type motif 17	NUDT17
nudix (nucleoside diphosphate linked moiety X)-type motif 18	NUDT18
nudix (nucleoside diphosphate linked moiety X)-type motif 19	NUDT19
nudix (nucleoside diphosphate linked moiety X)-type motif 2	NUDT2
nudix (nucleoside diphosphate linked moiety X)-type motif 21	NUDT21
Nudix (nucleoside diphosphate linked moiety X)-type motif 22	NUDT22
nudix (nucleoside diphosphate linked moiety X)-type motif 3	NUDT3
nudix (nucleoside diphosphate linked moiety X)-type motif 4	NUDT4
nudix (nucleoside diphosphate linked moiety X)-type motif 5	NUDT5
nudix (nucleoside diphosphate linked moiety X)-type motif 6	NUDT6
nudix (nucleoside diphosphate linked moiety X)-type motif 7	NUDT7
nudix (nucleoside diphosphate linked moiety X)-type motif 8	NUDT8
nudix (nucleoside diphosphate linked moiety X)-type motif 9	NUDT9
ribonucleotide reductase M1 polypeptide	RRM1
ribonucleotide reductase M2 polypeptide	RRM2
ribonucleotide reductase M2 B (TP53 inducible)	RRM2B
TDP-glucose 4,6-dehydratase	TGDS
UDP-glucose dehydrogenase	UGDH
UDP-glucuronate decarboxylase 1	UXS1
APOBEC1 complementation factor	A1CF
ACN9 homolog (S. cerevisiae)	ACN9
acyloxyacyl hydrolase (neutrophil)	AOAH
N-acylaminoacyl-peptide hydrolase	APEH
arsenic (+3 oxidation state) methyltransferase	AS3MT
biphenyl hydrolase-like (serine hydrolase; breast epithelial mucir	BPHL
carboxymethylenebutenolidase homolog (Pseudomonas)	CMBL
carnosine dipeptidase 1 (metallopeptidase M20 family)	CNDP1
CNDP dipeptidase 2 (metallopeptidase M20 family)	CNDP2
DDHD domain containing 1	DDHD1
DDHD domain containing 2	DDHD2
enolase-phosphatase 1	ENOPH1
enolase superfamily member 1	ENOSF1
eosinophil peroxidase	EPX
esterase D/formylglutathione hydrolase	ESD
frataxin	FXN
glycerophosphodiester phosphodiesterase domain containing 1	GDPD1
glycerophosphodiester phosphodiesterase domain containing 2	GDPD2
glycerophosphodiester phosphodiesterase domain containing 3	GDPD3
glycerophosphodiester phosphodiesterase domain containing 4	GDPD4
glycerophosphodiester phosphodiesterase domain containing 5	GDPD5
glycine-N-acyltransferase	GLYAT
glycine-N-acyltransferase-like 1	GLYATL1
glycine-N-acyltransferase-like 2	GLYATL2
glycine N-methyltransferase	GNMT

histidine acid phosphatase domain containing 1	HISPPD1
histidine acid phosphatase domain containing 2A	HISPPD2A
isoamyl acetate-hydrolyzing esterase 1 homolog (S. cerevisiae)	IAH1
lipoic acid synthetase	LIAS
lipoyltransferase 1	LIPT1
MOCO sulphurase C-terminal domain containing 1	MOSC1
MOCO sulphurase C-terminal domain containing 2	MOSC2
myeloperoxidase	MPO
nitrilase 1	NIT1
nitrilase family, member 2	NIT2
peptidylglycine alpha-amidating monooxygenase	PAM
paraoxonase 1	PON1
paraoxonase 2	PON2
paraoxonase 3	PON3
pyrophosphatase (inorganic) 1	PPA1
pyrophosphatase (inorganic) 2	PPA2
phosphotriesterase related	PTER
thioesterase superfamily member 2	THEM2
thioesterase superfamily member 4	THEM4
thioesterase superfamily member 5	THEM5
UEV and lactate/malate dehyrogenase domains	UEVLD
WW domain containing oxidoreductase	WWOX
glycolipid transfer protein	GLTP
Cystinosis, nephropathic	CTNS
Disrupted in renal carcinoma 2	DIRC2
Feline leukemia virus subgroup C cellular receptor 1	FLVCR1
Feline leukemia virus subgroup C cellular receptor family, memb	FLVCR2
Mitochondrial carrier triple repeat 1	MCART1
Mitochondrial carrier triple repeat 2	MCART2
Mitochondrial carrier triple repeat 6	MCART6
Major Facilitator Superfamily Domain Containing 1	MFSD1
Major Facilitator Superfamily Domain Containing 10	MFSD10
Major Facilitator Superfamily Domain Containing 11	MFSD11
Major Facilitator Superfamily Domain Containing 2	MFSD2
Major Facilitator Superfamily Domain Containing 3	MFSD3
Major Facilitator Superfamily Domain Containing 4	MFSD4
Major Facilitator Superfamily Domain Containing 5	MFSD5
Major Facilitator Superfamily Domain Containing 7	MFSD7
Major Facilitator Superfamily Domain Containing 8	MFSD8
Major Facilitator Superfamily Domain Containing 9	MFSD9
organic solute transporter alpha	OSTalpha
organic solute transporter beta	OSTbeta
stimulated by retinoic acid gene 6 homolog (mouse)	STRA6
transporter 1, ATP-binding cassette, sub-family B (MDR/TAP)	TAP1
transporter 2, ATP-binding cassette, sub-family B (MDR/TAP)	TAP2
Transmembrane and coiled-coil domains 3	TMCO3
Transmembrane protein 104	TMEM104
two pore segment channel 1	TPCN1

Two pore segment channel 2	TPCN2
2-deoxyribose-5-phosphate aldolase homolog (C. elegans)	DERA
glucose-6-phosphate dehydrogenase	G6PD
phosphogluconate dehydrogenase	PGD
6-phosphogluconolactonase	PGLS
ribulose-5-phosphate-3-epimerase	RPE
ribose 5-phosphate isomerase A (ribose 5-phosphate epimerase	RPIA
transaldolase 1	TALDO1
transketolase (Wernicke-Korsakoff syndrome)	TKT
transketolase-like 1	TKTL1
transketolase-like 2	TKTL2
dopachrome tautomerase (dopachrome delta-isomerase, tyrosin	DCT
D-dopachrome tautomerase	DDT
tyrosinase (oculocutaneous albinism IA)	TYR
tyrosinase-related protein 1	TYRP1
alpha-methylacyl-CoA racemase	AMACR
adenosylmethionine decarboxylase 1	AMD1
methylthioadenosine phosphorylase	MTAP
ornithine decarboxylase 1	ODC1
polyamine oxidase (exo-N4-amino)	PAOX
spermidine/spermine N1-acetyltransferase 1	SAT1
spermidine/spermine N1-acetyltransferase family member 2	SAT2
spermidine/spermine N1-acetyl transferase-like 1	SATL1
spermine oxidase	SMOX
spermine synthase	SMS
spermidine synthase	SRM
aminolevulinate, delta-, dehydratase	ALAD
aminolevulinate, delta-, synthase 1	ALAS1
aminolevulinate, delta-, synthase 2 (sideroblastic/hypochromic a	ALAS2
Biliverdin reductase A	BLVRA
biliverdin reductase B (flavin reductase (NADPH))	BLVRB
coproporphyrinogen oxidase	CPOX
ferrochelatase (protoporphyria)	FECH
hydroxymethylbilane synthase	HMBS
heme oxygenase (decycling) 1	HMOX1
heme oxygenase (decycling) 2	HMOX2
protoporphyrinogen oxidase	PPOX
uroporphyrinogen decarboxylase	UROD
uroporphyrinogen III synthase (congenital erythropoietic porphyri	UROS
aldehyde dehydrogenase 4 family, member A1	ALDH4A1
proline dehydrogenase (oxidase) 1	PRODH
proline dehydrogenase (oxidase) 2	PRODH2
pyrroline-5-carboxylate reductase 1	PYCR1
pyrroline-5-carboxylate reductase family, member 2	PYCR2
pyrroline-5-carboxylate reductase-like	PYCRL
methylmalonyl CoA epimerase	MCEE
methylmalonyl Coenzyme A mutase	MUT
propionyl Coenzyme A carboxylase, alpha polypeptide	PCCA

Propionyl Coenzyme A carboxylase, beta polypeptide	PCCB
ATP synthase, H+ transporting, mitochondrial F1 complex, alpha	ATP5A1
ATP synthase, H+ transporting mitochondrial F1 complex, beta s	ATP5B
ATP synthase, H+ transporting, mitochondrial F1 complex, gamr	ATP5C1
ATP synthase, H+ transporting, mitochondrial F1 complex, gamr	ATP5C2
ATP synthase, H+ transporting, mitochondrial F1 complex, delta	ATP5D
ATP synthase, H+ transporting, mitochondrial F1 complex, epsil	ATP5E
ATP synthase, H+ transporting, mitochondrial F0 complex, subu	ATP5F1
ATP synthase, H+ transporting, mitochondrial F0 complex, subu	ATP5G1
ATP synthase, H+ transporting, mitochondrial F0 complex, subu	ATP5G2
ATP synthase, H+ transporting, mitochondrial F0 complex, subu	ATP5G3
ATP synthase, H+ transporting, mitochondrial F0 complex, subu	ATP5H
ATP synthase, H+ transporting, mitochondrial F0 complex, subu	ATP5I
ATP synthase, H+ transporting, mitochondrial F0 complex, subu	ATP5J
ATP synthase, H+ transporting, mitochondrial F0 complex, subu	ATP5J2
ATP synthase, H+ transporting, mitochondrial F0 complex, subu	ATP5L
ATP synthase, H+ transporting, mitochondrial F0 complex, subu	ATP5L2
ATP synthase, H+ transporting, mitochondrial F1 complex, O sul	ATP5O
ATP synthase, H+ transporting, mitochondrial F0 complex, subu	ATP5S
ATP5S-like	ATP5SL
mitochondrially encoded ATP synthase 6	ATP6
ATPase, H+ transporting, lysosomal accessory protein 1	ATP6AP1
ATPase, H+ transporting, lysosomal accessory protein 2	ATP6AP2
ATPase, H+ transporting, lysosomal V0 subunit A1	ATP6V0A1
ATPase, H+ transporting, lysosomal V0 subunit A2	ATP6V0A2
ATPase, H+ transporting, lysosomal V0 subunit A4	ATP6V0A4
ATPase, H+ transporting, lysosomal V0 subunit B	ATP6V0B
ATPase, H+ transporting, lysosomal V0 subunit C	ATP6V0C
ATPase, H+ transporting, lysosomal V0 subunit D1	ATP6V0D1
ATPase, H+ transporting, lysosomal V0 subunit D2	ATP6V0D2
ATPase, H+ transporting, lysosomal V0 subunit E	ATP6V0E1
ATPase, H+ transporting, lysosomal V0 subunit E2	ATP6V0E2
ATPase, H+ transporting, lysosomal V1 subunit A	ATP6V1A
ATPase, H+ transporting, lysosomal V1 subunit B1	ATP6V1B1
ATPase, H+ transporting, lysosomal V1 subunit B2	ATP6V1B2
ATPase, H+ transporting, lysosomal V1 subunit C1	ATP6V1C1
ATPase, H+ transporting, lysosomal V1 subunit C2	ATP6V1C2
ATPase, H+ transporting, lysosomal V1 subunit D	ATP6V1D
ATPase, H+ transporting, lysosomal V1 subunit E1	ATP6V1E1
ATPase, H+ transporting, lysosomal V1 subunit E2	ATP6V1E2
ATPase, H+ transporting, lysosomal V1 subunit F	ATP6V1F
ATPase, H+ transporting, lysosomal V1 subunit G1	ATP6V1G1
ATPase, H+ transporting, lysosomal V1 subunit G2	ATP6V1G2
ATPase, H+ transporting, lysosomal V1 subunit G3	ATP6V1G3
ATPase, H+ transporting, lysosomal V1 subunit H	ATP6V1H
hydrogen voltage-gated channel 1	HVCN1
T-cell, immune regulator 1, ATPase, H+ transporting, lysosomal	TCIRG1
uncoupling protein 1 (mitochondrial, proton carrier)	UCP1

uncoupling protein 2 (mitochondrial, proton carrier)	UCP2
uncoupling protein 3 (mitochondrial, proton carrier)	UCP3
adenosine deaminase	ADA
Adenosine deaminase domain containing 1 (testis-specific)	ADAD1
adenosine deaminase domain containing 2	ADAD2
adenosine deaminase-like	ADAL
adenosine kinase	ADK
adenylosuccinate lyase	ADSL
Adenylosuccinate synthase	ADSS
adenylosuccinate synthase like 1	ADSSL1
adenylate kinase 1	AK1
adenylate kinase 2	AK2
adenylate kinase 3	AK3
adenylate kinase 3-like 1	AK3L1
adenylate kinase 5	AK5
adenylate kinase 7	AK7
adenosine monophosphate deaminase 1 (isoform M)	AMPD1
adenosine monophosphate deaminase 2 (isoform L)	AMPD2
adenosine monophosphate deaminase (isoform E)	AMPD3
adenine phosphoribosyltransferase	APRT
5-aminoimidazole-4-carboxamide ribonucleotide formyltransfera	ATIC
cat eye syndrome chromosome region, candidate 1	CECR1
deoxyguanosine kinase	DGUOK
Fragile histidine triad gene	FHIT
phosphoribosylglycinamide formyltransferase, phosphoribosylgly	GART
guanine deaminase	GDA
guanosine monophosphate reductase	GMPR
guanosine monophosphate reductase 2	GMPR2
guanine monphosphate synthetase	GMPS
guanylate kinase 1	GUK1
Hypoxanthine phosphoribosyltransferase 1 (Lesch-Nyhan syndro	HPRT1
IMP (inosine monophosphate) dehydrogenase 1	IMPDH1
IMP (inosine monophosphate) dehydrogenase 2	IMPDH2
nucleoside phosphorylase	NP
phosphoribosylaminoimidazole carboxylase, phosphoribosylamir	PAICS
phosphoribosylformylglycinamidine synthase (FGAR amidotrans	PFAS
phosphoribosyl pyrophosphate amidotransferase	PPAT
phosphoribosyl pyrophosphate synthetase 1	PRPS1
phosphoribosyl pyrophosphate synthetase 1-like 1	PRPS1L1
phosphoribosyl pyrophosphate synthetase 2	PRPS2
Phosphoribosyl pyrophosphate synthetase-associated protein 1	PRPSAP1
phosphoribosyl pyrophosphate synthetase-associated protein 2	PRPSAP2
xanthine dehydrogenase	XDH
Carbamoyl-phosphate synthetase 2, aspartate transcarbamylase	CAD
cytidine deaminase	CDA
cytidine and dCMP deaminase domain containing 1	CDADC1
cytidylate kinase	CMPK
CTP synthase	CTPS

CTP synthase II	CTPS2
dCMP deaminase	DCTD
dihydroorotate dehydrogenase	DHODH
dihydropyrimidine dehydrogenase	DPYD
dihydropyrimidinase	DPYS
dihydropyrimidinase-like 2	DPYSL2
dihydropyrimidinase-like 3	DPYSL3
dihydropyrimidinase-like 4	DPYSL4
dihydropyrimidinase-like 5	DPYSL5
deoxythymidylate kinase (thymidylate kinase)	DTYMK
deoxyuridine triphosphatase	DUT
thymidine kinase 1, soluble	TK1
thymidine kinase 2, mitochondrial	TK2
Thymidine phosphorylase	TYMP
thymidylate synthetase	TYMS
uridine-cytidine kinase 1	UCK1
uridine-cytidine kinase 2	UCK2
uridine-cytidine kinase 1-like 1	UCKL1
uridine monophosphate synthetase (orotate phosphoribosyl tran	UMPS
ureidopropionase, beta	UPB1
uridine phosphorylase 1	UPP1
uridine phosphorylase 2	UPP2
uracil phosphoribosyltransferase (FUR1) homolog (S. cerevisiae	UPRT
aldehyde oxidase 1	AOX1
dimethylarginine dimethylaminohydrolase 1	DDAH1
dimethylarginine dimethylaminohydrolase 2	DDAH2
nitric oxide synthase 1 (neuronal)	NOS1
Nitric oxide synthase 2 (inducible, hepatocytes)	NOS2
nitric oxide synthase 3 (endothelial cell)	NOS3
ceruloplasmin (ferroxidase)	CP
crystallin, zeta (quinone reductase)	CRYZ
crystallin, zeta (quinone reductase)-like 1	CRYZL1
cytochrome b-561	CYB561
cytochrome b5 type A (microsomal)	CYB5A
cytochrome b5 type B (outer mitochondrial membrane)	CYB5B
cytochrome b5 domain containing 1	CYB5D1
cytochrome b5 domain containing 2	CYB5D2
cytochrome b5 reductase 1	CYB5R1
cytochrome b5 reductase 2	CYB5R2
cytochrome b5 reductase 3	CYB5R3
cytochrome b5 reductase 4	CYB5R4
cytochrome b5 reductase like	CYB5RL
cytochrome b-245, alpha polypeptide	CYBA
cytochrome b, ascorbate dependent 3	CYBASC3
cytochrome b-245, beta polypeptide (chronic granulomatous dis	CYBB
cytochrome b reductase 1	CYBRD1
dehydrogenase/reductase (SDR family) member 1	DHRS1
dehydrogenase/reductase (SDR family) member 11	DHRS11

dehydrogenase/reductase (SDR family) member 12	DHRS12
dehydrogenase/reductase (SDR family) member 13	DHRS13
dehydrogenase/reductase (SDR family) member 2	DHRS2
dehydrogenase/reductase (SDR family) member 3	DHRS3
dehydrogenase/reductase (SDR family) member 4	DHRS4
dehydrogenase/reductase (SDR family) member 4 like 1	DHRS4L1
dehydrogenase/reductase (SDR family) member 4 like 2	DHRS4L2
dehydrogenase/reductase (SDR family) member 7	DHRS7
dehydrogenase/reductase (SDR family) member 7B	DHRS7B
dehydrogenase/reductase (SDR family) member 7C	DHRS7C
dehydrogenase/reductase (SDR family) member 9	DHRS9
dehydrogenase/reductase (SDR family) X-linked	DHRSX
dehydrogenase E1 and transketolase domain containing 1	DHTKD1
ecto-NOX disulfide-thiol exchanger 1	ENOX1
ecto-NOX disulfide-thiol exchanger 2	ENOX2
ferredoxin 1	FDX1
ferredoxin 1-like	FDX1L
ferredoxin reductase	FDXR
FAD-dependent oxidoreductase domain containing 1	FOXRED1
FAD-dependent oxidoreductase domain containing 2	FOXRED2
ferric-chelate reductase 1	FRRS1
glutaredoxin (thioltransferase)	GLRX
glutaredoxin 2	GLRX2
glutaredoxin 3	GLRX3
glutaredoxin 5	GLRX5
glutaredoxin, cysteine rich 1	GRXCR1
glutaredoxin, cysteine rich 2	GRXCR2
iron-sulfur cluster assembly 1 homolog (S. cerevisiae)	ISCA1
iron-sulfur cluster assembly 1 homolog (S. cerevisiae)-l	ISCA1L
iron-sulfur cluster assembly 2 homolog (S. cerevisiae)	ISCA2
lactoperoxidase	LPO
microtubule associated monoxygenase, calponin and LIM domai	MICAL1
microtubule associated monoxygenase, calponin and LIM domai	MICAL2
microtubule associated monoxygenase, calponin and LIM domai	MICAL3
NADPH dependent diflavin oxidoreductase 1	NDOR1
NmrA-like family domain containing 1	NMRAL1
2-oxoglutarate and iron-dependent oxygenase domain containin	OGFOD1
2-oxoglutarate and iron-dependent oxygenase domain containin	OGFOD2
oxidase (cytochrome c) assembly 1-like	OXA1L
peroxiredoxin 1	PRDX1
peroxiredoxin 2	PRDX2
peroxiredoxin 3	PRDX3
peroxiredoxin 4	PRDX4
peroxiredoxin 5	PRDX5
peroxiredoxin 6	PRDX6
pyridine nucleotide-disulphide oxidoreductase domain 1	PYROXD1
Thioredoxin reductase 1	TXNRD1
Thioredoxin reductase 2	TXNRD2

Thioredoxin reductase 3	TXNRD3
phosphoglycerate dehydrogenase	PHGDH
phosphoserine transaminase	PSAT
phosphoserine phosphatase	PSPH
adenylate cyclase 1 (brain)	ADCY1
testicular soluble adenylyl cyclase	ADCY10
adenylate cyclase 2 (brain)	ADCY2
adenylate cyclase 3	ADCY3
adenylate cyclase 4	ADCY4
adenylate cyclase 5	ADCY5
adenylate cyclase 6	ADCY6
adenylate cyclase 7	ADCY7
adenylate cyclase 8 (brain)	ADCY8
Adenylate cyclase 9	ADCY9
Chromosome 9 ORF 98	C9ORF98
2',3'-cyclic nucleotide 3' phosphodiesterase	CNP
guanylate cyclase 1, soluble, alpha 2	GUCY1A2
guanylate cyclase 1, soluble, alpha 3	GUCY1A3
guanylate cyclase 1, soluble, beta 3	GUCY1B3
guanylate cyclase 2C (heat stable enterotoxin receptor)	GUCY2C
guanylate cyclase 2D, membrane (retina-specific)	GUCY2D
guanylate cyclase 2F, retinal	GUCY2F
natriuretic peptide receptor A/guanylate cyclase A (atrionatriureti	NPR1
natriuretic peptide receptor B/guanylate cyclase B (atrionatriureti	NPR2
natriuretic peptide receptor C/guanylate cyclase C (atrionatriuret	NPR3
2',5'-oligoadenylate synthetase 1, 40/46kDa	OAS1
2'-5'-oligoadenylate synthetase 2, 69/71kDa	OAS2
2'-5'-oligoadenylate synthetase 3, 100kDa	OAS3
2'-5'-oligoadenylate synthetase-like	OASL
phosphodiesterase 10A	PDE10A
phosphodiesterase 11A	PDE11A
2'-phosphodiesterase	PDE12
phosphodiesterase 1A, calmodulin-dependent	PDE1A
phosphodiesterase 1B, calmodulin-dependent	PDE1B
phosphodiesterase 1C, calmodulin-dependent 70kDa	PDE1C
phosphodiesterase 2A, cGMP-stimulated	PDE2A
phosphodiesterase 3A, cGMP-inhibited	PDE3A
phosphodiesterase 3B, cGMP-inhibited	PDE3B
phosphodiesterase 4A, cAMP-specific (phosphodiesterase E2 d	PDE4A
phosphodiesterase 4B, cAMP-specific (phosphodiesterase E4 d	PDE4B
phosphodiesterase 4C, cAMP-specific (phosphodiesterase E1 d	PDE4C
phosphodiesterase 4D, cAMP-specific (phosphodiesterase E3 d	PDE4D
phosphodiesterase 5A, cGMP-specific	PDE5A
phosphodiesterase 6A, cGMP-specific, rod, alpha	PDE6A
phosphodiesterase 6B, cGMP-specific, rod, beta (congenital sta	PDE6B
phosphodiesterase 6C, cGMP-specific, cone, alpha prime	PDE6C
phosphodiesterase 6D, cGMP-specific, rod, delta	PDE6D
phosphodiesterase 6G, cGMP-specific, rod, gamma	PDE6G

phosphodiesterase 6H, cGMP-specific, cone, gamma	PDE6H
phosphodiesterase 7A	PDE7A
phosphodiesterase 7B	PDE7B
phosphodiesterase 8A	PDE8A
phosphodiesterase 8B	PDE8B
phosphodiesterase 9A	PDE9A
amiloride-sensitive cation channel 1, neuronal (degenerin)	ACCN1
amiloride-sensitive cation channel 2, neuronal	ACCN2
amiloride-sensitive cation channel 3	ACCN3
amiloride-sensitive cation channel 4, pituitary	ACCN4
amiloride-sensitive cation channel 5, intestinal	ACCN5
Ankylosis, progressive homolog (mouse)	ANKH
aquaporin 1 (Colton blood group)	AQP1
aquaporin 10	AQP10
aquaporin 11	AQP11
aquaporin 12A	AQP12A
aquaporin 12B	AQP12B
aquaporin 2 (collecting duct)	AQP2
aquaporin 3 (Gill blood group)	AQP3
aquaporin 4	AQP4
aquaporin 5	AQP5
aquaporin 6, kidney specific	AQP6
aquaporin 7	AQP7
aquaporin 8	AQP8
aquaporin 9	AQP9
ArsA arsenite transporter, ATP-binding, homolog 1 (bacterial)	ASNA1
ATPase, class V, type 10A	ATP10A
ATPase, class V, type 10B	ATP10B
ATPase, class V, type 10D	ATP10D
mitochondrially encoded ATP synthase 8	ATP8
ATPase, aminophospholipid transporter (APLT), class I, type 8A	ATP8A1
ATPase, aminophospholipid transporter-like, class I, type 8A, me	ATP8A2
ATPase, class I, type 8B, member 1	ATP8B1
ATPase, class I, type 8B, member 2	ATP8B2
ATPase, class I, type 8B, member 3	ATP8B3
ATPase, class I, type 8B, member 4	ATP8B4
ATPase, class II, type 9A	ATP9A
ATPase, class II, type 9B	ATP9B
folate receptor 1 (adult)	FOLR1
folate receptor 2 (fetal)	FOLR2
folate receptor 3 (gamma)	FOLR3
solute carrier family 10 (sodium/bile acid cotransporter family), m	SLC10A1
solute carrier family 10, member 2	SLC10A2
solute carrier family 10 (sodium/bile acid cotransporter family), m	SLC10A3
solute carrier family 10 (sodium/bile acid cotransporter family), m	SLC10A4
solute carrier family 10 (sodium/bile acid cotransporter family), m	SLC10A5
solute carrier family 10 (sodium/bile acid cotransporter family), m	SLC10A6
solute carrier family 10 (sodium/bile acid cotransporter family), m	SLC10A7

solute carrier family 14 (urea transporter), member 1	SLC14A1
solute carrier family 14 (urea transporter), member 2	SLC14A2
solute carrier family 15 (oligopeptide transporter), member 1	SLC15A1
solute carrier family 15 (H+/peptide transporter), member 2	SLC15A2
solute carrier family 15, member 3	SLC15A3
solute carrier family 15, member 4	SLC15A4
solute carrier family 16 (monocarboxylic acid transporters), mem	SLC16A1
solute carrier family 16 (monocarboxylic acid transporters), mem	SLC16A10
solute carrier family 16 (monocarboxylic acid transporters), mem	SLC16A11
solute carrier family 16 (monocarboxylic acid transporters), mem	SLC16A12
solute carrier family 16 (monocarboxylic acid transporters), mem	SLC16A13
solute carrier family 16 (monocarboxylic acid transporters), mem	SLC16A14
solute carrier family 16 (monocarboxylic acid transporters), mem	SLC16A2
solute carrier family 16 (monocarboxylic acid transporters), mem	SLC16A3
solute carrier family 16 (monocarboxylic acid transporters), mem	SLC16A4
solute carrier family 16 (monocarboxylic acid transporters), mem	SLC16A5
solute carrier family 16 (monocarboxylic acid transporters), mem	SLC16A6
solute carrier family 16 (monocarboxylic acid transporters), mem	SLC16A7
solute carrier family 16 (monocarboxylic acid transporters), mem	SLC16A8
solute carrier family 16 (monocarboxylic acid transporters), mem	SLC16A9
solute carrier family 17 (sodium phosphate), member 1	SLC17A1
solute carrier family 17 (sodium phosphate), member 2	SLC17A2
solute carrier family 17 (sodium phosphate), member 3	SLC17A3
solute carrier family 17 (sodium phosphate), member 4	SLC17A4
solute carrier family 17 (anion/sugar transporter), member 5	SLC17A5
solute carrier family 17 (sodium-dependent inorganic phosphate	SLC17A6
solute carrier family 17 (sodium-dependent inorganic phosphate	SLC17A7
solute carrier family 17 (sodium-dependent inorganic phosphate	SLC17A8
solute carrier family 18 (vesicular monoamine), member 1	SLC18A1
solute carrier family 18 (vesicular monoamine), member 2	SLC18A2
solute carrier family 18 (vesicular monoamine), member 3	SLC18A3
solute carrier family 19 (folate transporter), member 1	SLC19A1
solute carrier family 19 (thiamine transporter), member 2	SLC19A2
solute carrier family 19 (sodium/hydrogen exchanger), member 3	SLC19A3
solute carrier family 1 (neuronal/epithelial high affinity glutamate	SLC1A1
solute carrier family 1 (glial high affinity glutamate transporter), m	SLC1A2
solute carrier family 1 (glial high affinity glutamate transporter), m	SLC1A3
solute carrier family 1 (glutamate/neutral amino acid transporter)	SLC1A4
solute carrier family 1 (neutral amino acid transporter), member 5	SLC1A5
solute carrier family 1 (high affinity aspartate/glutamate transport	SLC1A6
solute carrier family 1 (glutamate transporter), member 7	SLC1A7
solute carrier family 20, member 1	SLC20A1
solute carrier family 20, member 2	SLC20A2
solute carrier family 22 (organic cation transporter), member 1	SLC22A1
solute carrier family 22 (organic anion/cation transporter), memb	SLC22A11
solute carrier family 22 (organic anion/cation transporter), memb	SLC22A12
solute carrier family 22 (organic cation transporter), member 13	SLC22A13
solute carrier family 22 (organic cation transporter), member 14	SLC22A14

solute carrier family 22 (organic cation transporter), member 15	SLC22A15
solute carrier family 22 (organic cation transporter), member 16	SLC22A16
solute carrier family 22 (organic cation transporter), member 17	SLC22A17
solute carrier family 22 (organic cation transporter), member 18	SLC22A18
solute carrier family 22 (organic cation transporter), member 18 ε	SLC22A18AS
solute carrier family 22 (organic cation transporter), member 2	SLC22A2
solute carrier family 22 (organic anion transporter), member 20	SLC22A20
solute carrier family 22, member 23	SLC22A23
solute carrier family 22 (organic cation transporter), member 3	SLC22A3
solute carrier family 22 (organic cation transporter), member 4	SLC22A4
solute carrier family 22 (organic cation transporter), member 5	SLC22A5
solute carrier family 22 (organic anion transporter), member 6	SLC22A6
solute carrier family 22 (organic anion transporter), member 7	SLC22A7
solute carrier family 22 (organic anion transporter), member 8	SLC22A8
solute carrier family 22 (organic anion transporter), member 9	SLC22A9
solute carrier family 23 (nucleobase transporters), member 1	SLC23A1
solute carrier family 23 (nucleobase transporters), member 2	SLC23A2
solute carrier family 23 (nucleobase transporters), member 3	SLC23A3
solute carrier family 25 (mitochondrial carrier, citrate transporter)	SLC25A1
solute carrier family 25 (mitochondrial carrier, dicarboxylate trans	SLC25A10
solute carrier family 25 (mitochondrial carrier oxoglutarate carrie	SLC25A11
solute carrier family 25 (mitochondrial carrier, Aralar), member 1	SLC25A12
solute carrier family 25 (mitochondrial carrier, adenine nucleotide	SLC25A13
solute carrier family 25 (mitochondrial carrier, brain), member 14	SLC25A14
solute carrier family 25 (mitochondrial carrier ornithine transporte	SLC25A15
solute carrier family 25 (mitochondrial carrier, Graves disease au	SLC25A16
solute carrier family 25 (mitochondrial carrier, peroxisomal meml	SLC25A17
solute carrier family 25 (mitochondrial carrier), member 18	SLC25A18
solute carrier family 25 (mitochondrial deoxynucleotide carrier), r	SLC25A19
solute carrier family 25 (mitochondrial carrier, ornithine transport	SLC25A2
solute carrier family 25 (mitochondrial carnitine/acylcarnitine tran	SLC25A20
solute carrier family 25 (mitochondrial oxodicarboxylate carrier),	SLC25A21
solute carrier family 25 (mitochondrial carrier, glutamate), memb	SLC25A22
solute carrier family 25 (mitochondrial carrier; phosphate carrier)	SLC25A23
solute carrier family 25 (mitochondrial carrier, phosphate carrier)	SLC25A24
solute carrier family 25 (mitochondrial carrier, phosphate carrier)	SLC25A25
solute carrier family 25 (mitochondrial carrier, phosphate carrier)	SLC25A26
solute carrier family 25, member 27	SLC25A27
solute carrier family 25, member 28	SLC25A28
solute carrier family 25 (mitochondrial carrier, palmitoylcarnitine t	SLC25A29
solute carrier family 25 (mitochondrial carrier, phosphate carrier)	SLC25A3
solute carrier family 25, member 30	SLC25A30
solute carrier family 25 (mitochondrial carrier; adenine nucleotide	SLC25A31
solute carrier family 25, member 32	SLC25A32
solute carrier family 25, member 33	SLC25A33
solute carrier family 25, member 34	SLC25A34
solute carrier family 25, member 35	SLC25A35
solute carrier family 25, member 36	SLC25A36

solute carrier family 25, member 37	SLC25A37
solute carrier family 25, member 38	SLC25A38
solute carrier family 25, member 39	SLC25A39
solute carrier family 25 (mitochondrial carrier, adenine nucleotide	SLC25A4
solute carrier family 25, member 40	SLC25A40
solute carrier family 25, member 41	SLC25A41
solute carrier family 25, member 42	SLC25A42
solute carrier family 25, member 43	SLC25A43
solute carrier family 25, member 44	SLC25A44
solute carrier family 25, member 45	SLC25A45
solute carrier family 25, member 46	SLC25A46
solute carrier family 25 (mitochondrial carrier, adenine nucleotide	SLC25A5
solute carrier family 25 (mitochondrial carrier; adenine nucleotide	SLC25A6
solute carrier family 26 (sulfate transporter), member 1	SLC26A1
solute carrier family 26, member 10	SLC26A10
solute carrier family 26, member 11	SLC26A11
solute carrier family 26 (sulfate transporter), member 2	SLC26A2
solute carrier family 26, member 3	SLC26A3
solute carrier family 26, member 4	SLC26A4
solute carrier family 26, member 5	SLC26A5
solute carrier family 26, member 6	SLC26A6
solute carrier family 26, member 7	SLC26A7
solute carrier family 26, member 8	SLC26A8
solute carrier family 26, member 9	SLC26A9
solute carrier family 27 (fatty acid transporter), member 1	SLC27A1
solute carrier family 27 (fatty acid transporter), member 2	SLC27A2
solute carrier family 27 (fatty acid transporter), member 3	SLC27A3
solute carrier family 27 (fatty acid transporter), member 4	SLC27A4
solute carrier family 27 (fatty acid transporter), member 5	SLC27A5
solute carrier family 27 (fatty acid transporter), member 6	SLC27A6
solute carrier family 28 (sodium-coupled nucleoside transporter),	SLC28A1
solute carrier family 28 (sodium-coupled nucleoside transporter),	SLC28A2
solute carrier family 28 (sodium-coupled nucleoside transporter),	SLC28A3
solute carrier family 29 (nucleoside transporters), member 1	SLC29A1
solute carrier family 29 (nucleoside transporters), member 2	SLC29A2
solute carrier family 29 (nucleoside transporters), member 3	SLC29A3
solute carrier family 29 (nucleoside transporters), member 4	SLC29A4
solute carrier family 2 (facilitated glucose transporter), member 1	SLC2A1
solute carrier family 2 (facilitated glucose transporter), member 1	SLC2A10
solute carrier family 2 (facilitated glucose transporter), member 1	SLC2A11
solute carrier family 2 (facilitated glucose transporter), member 1	SLC2A12
solute carrier family 2 (facilitated glucose transporter), member 1	SLC2A13
Solute carrier family 2 (facilitated glucose transporter), member	SLC2A14
solute carrier family 2 (facilitated glucose transporter), member 2	SLC2A2
solute carrier family 2 (facilitated glucose transporter), member 3	SLC2A3
solute carrier family 2 (facilitated glucose transporter), member 4	SLC2A4
solute carrier family 2 (facilitated glucose transporter), member 5	SLC2A5
solute carrier family 2 (facilitated glucose transporter), member 6	SLC2A6

solute carrier family 2 (facilitated glucose transporter), member 7	SLC2A7
solute carrier family 2, (facilitated glucose transporter), member	SLC2A8
solute carrier family 2 (facilitated glucose transporter), member 9	SLC2A9
solute carrier family 32 (GABA vesicular transporter), member 1	SLC32A1
solute carrier family 33 (acetyl-CoA transporter), member 1	SLC33A1
solute carrier family 34 (sodium phosphate), member 1	SLC34A1
solute carrier family 34 (sodium phosphate), member 2	SLC34A2
solute carrier family 34 (sodium phosphate), member 3	SLC34A3
solute carrier family 35 (CMP-sialic acid transporter), member 1	SLC35A1
solute carrier family 35 (UDP-galactose transporter), member 2	SLC35A2
solute carrier family 35 (UDP-N-acetylglucosamine (UDP-GlcNA	SLC35A3
solute carrier family 35, member A4	SLC35A4
solute carrier family 35, member A5	SLC35A5
solute carrier family 35, member B1	SLC35B1
solute carrier family 35, member B2	SLC35B2
solute carrier family 35, member B3	SLC35B3
solute carrier family 35, member B4	SLC35B4
solute carrier family 35, member C1	SLC35C1
solute carrier family 35, member C2	SLC35C2
solute carrier family 35 (UDP-glucuronic acid/UDP-N-acetylgalac	SLC35D1
solute carrier family 35 (UDP-glucuronic acid/UDP-N-acetylgalac	SLC35D2
solute carrier family 35, member D3	SLC35D3
solute carrier family 35, member E1	SLC35E1
solute carrier family 35, member E2	SLC35E2
solute carrier family 35, member E3	SLC35E3
solute carrier family 35, member E4	SLC35E4
solute carrier family 35, member F1	SLC35F1
solute carrier family 35, member F2	SLC35F2
solute carrier family 35, member F3	SLC35F3
solute carrier family 35, member F4	SLC35F4
solute carrier family 35, member F5	SLC35F5
solute carrier family 36 (proton/amino acid symporter), member	SLC36A1
solute carrier family 36 (proton/amino acid symporter), member	SLC36A2
solute carrier family 36 (proton/amino acid symporter), member	SLC36A3
solute carrier family 36 (proton/amino acid symporter), member	SLC36A4
solute carrier family 37 (glycerol-3-phosphate transporter), meml	SLC37A1
solute carrier family 37 (glycerol-3-phosphate transporter), meml	SLC37A2
solute carrier family 37 (glycerol-3-phosphate transporter), meml	SLC37A3
solute carrier family 37 (glycerol-6-phosphate transporter), meml	SLC37A4
solute carrier family 38, member 1	SLC38A1
solute carrier family 38, member 2	SLC38A2
solute carrier family 38, member 3	SLC38A3
solute carrier family 38, member 4	SLC38A4
solute carrier family 38, member 5	SLC38A5
solute carrier family 38, member 6	SLC38A6
solute carrier family 38, member 7	SLC38A7
solute carrier family 43, member 1	SLC43A1
solute carrier family 43, member 2	SLC43A2

solute carrier family 43, member 3	SLC43A3
solute carrier family 44, member 1	SLC44A1
solute carrier family 44, member 2	SLC44A2
solute carrier family 44, member 3	SLC44A3
solute carrier family 44, member 4	SLC44A4
solute carrier family 44, member 5	SLC44A5
solute carrier family 45, member 1	SLC45A1
solute carrier family 45, member 2	SLC45A2
solute carrier family 45, member 3	SLC45A3
solute carrier family 45, member 4	SLC45A4
Solute carrier family 46 (folate transporter), member 1	SLC46A1
solute carrier family 46, member 2	SLC46A2
solute carrier family 46, member 3	SLC46A3
solute carrier family 47, member 1	SLC47A1
solute carrier family 47, member 2	SLC47A2
solute carrier family 4 (anion exchanger), member 1	SLC4A1
solute carrier family 4, sodium bicarbonate cotransporter-like, me	SLC4A10
solute carrier family 4, member 11	SLC4A11
solute carrier family 4 (anion exchanger), member 1, adaptor prc	SLC4A1AP
solute carrier family 4 (anion exchanger), member 2	SLC4A2
solute carrier family 4 (anion exchanger), member 3	SLC4A3
solute carrier family 4 (anion exchanger), member 4	SLC4A4
solute carrier family 4 (anion exchanger), member 5	SLC4A5
solute carrier family 4 (anion exchanger), member 7	SLC4A7
solute carrier family 4 (anion exchanger), member 8	SLC4A8
solute carrier family 4, sodium bicarbonate cotransporter, memb	SLC4A9
solute carrier family 5 (sodium/glucose cotransporter), member 1	SLC5A1
solute carrier family 5 (sodium/glucose cotransporter), member 1	SLC5A10
solute carrier family 5 (sodium/glucose cotransporter), member 1	SLC5A11
solute carrier family 5 (sodium/glucose cotransporter), member 1	SLC5A12
solute carrier family 5 (sodium/glucose cotransporter), member 2	SLC5A2
solute carrier family 5 (inositol transporters), member 3	SLC5A3
solute carrier family 5, member 4a	SLC5A4
solute carrier family 5 (sodium iodide symporter), member 5	SLC5A5
solute carrier family 5 (sodium-dependent vitamin transporter), m	SLC5A6
solute carrier family 5 (choline transporter), member 7	SLC5A7
solute carrier family 5 (iodide transporter), member 8	SLC5A8
solute carrier family 5 (sodium/glucose cotransporter), member 9	SLC5A9
solute carrier family 6 (neurotransmitter transporter, GABA), mer	SLC6A1
solute carrier family 6 (neurotransmitter transporter, GABA), mer	SLC6A11
solute carrier family 6 (neurotransmitter transporter, betaine/GAE	SLC6A12
solute carrier family 6 (neurotransmitter transporter, GABA), mer	SLC6A13
solute carrier family 6 (neurotransmitter transporter), member 14	SLC6A14
solute carrier family 6 (neurotransmitter transporter), member 15	SLC6A15
solute carrier family 6 (neurotransmitter transporter), member 16	SLC6A16
solute carrier family 6 (neurotransmitter transporter), member 17	SLC6A17
solute carrier family 6 (neurotransmitter transporter), member 18	SLC6A18
solute carrier family 6 (neurotransmitter transporter), member 19	SLC6A19

solute carrier family 6 (neurotransmitter transporter, noradrenalir	SLC6A2
solute carrier family 6 (neurotransmitter transporter), member 20	SLC6A20
solute carrier family 6 (neurotransmitter transporter, dopamine),	SLC6A3
solute carrier family 6 (neurotransmitter transporter, serotonin), r	SLC6A4
solute carrier family 6 (neurotransmitter transporter, glycine), me	SLC6A5
solute carrier family 6 (neurotransmitter transporter, taurine), me	SLC6A6
solute carrier family 6 (neurotransmitter transporter, L-proline), m	SLC6A7
solute carrier family 6 (neurotransmitter transporter, creatine), m	SLC6A8
solute carrier family 6 (neurotransmitter transporter, glycine), me	SLC6A9
solute carrier family 7 (cationic amino acid transporter, y+ systen	SLC7A1
solute carrier family 7 (cationic amino acid transporter, y+ systen	SLC7A10
solute carrier family 7 (cationic amino acid transporter, y+ systen	SLC7A11
solute carrier family 7, (cationic amino acid transporter, y+ syste	SLC7A13
solute carrier family 7 (cationic amino acid transporter, y+ systen	SLC7A14
solute carrier family 7 (cationic amino acid transporter, y+ systen	SLC7A2
solute carrier family 7 (cationic amino acid transporter, y+ systen	SLC7A3
solute carrier family 7 (cationic amino acid transporter, y+ systen	SLC7A4
solute carrier family 7 (cationic amino acid transporter, y+ systen	SLC7A5
solute carrier family 7 (cationic amino acid transporter, y+ systen	SLC7A6
solute carrier family 7 (cationic amino acid transporter, y+ systen	SLC7A7
solute carrier family 7 (cationic amino acid transporter, y+ systen	SLC7A8
solute carrier family 7 (cationic amino acid transporter, y+ systen	SLC7A9
solute carrier family 8 (sodium/calcium exchanger), member 1	SLC8A1
solute carrier family 8 (sodium/calcium exchanger), member 2	SLC8A2
solute carrier family 8 (sodium/calcium exchanger), member 3	SLC8A3
solute carrier family 9 (sodium/hydrogen exchanger), member 1	SLC9A1
solute carrier family 9 (sodium/hydrogen exchanger), isoform 10	SLC9A10
solute carrier family 9, member 11	SLC9A11
solute carrier family 9 (sodium/hydrogen exchanger), member 2	SLC9A2
solute carrier family 9 (sodium/hydrogen exchanger), member 3	SLC9A3
solute carrier family 9 (sodium/hydrogen exchanger), member 4	SLC9A4
solute carrier family 9 (sodium/hydrogen exchanger), member 5	SLC9A5
solute carrier family 9 (sodium/hydrogen exchanger), isoform 6	SLC9A6
solute carrier family 9 (sodium/hydrogen exchanger), isoform 7	SLC9A7
solute carrier family 9 (sodium/hydrogen exchanger), member 8	SLC9A8
solute carrier family 9 (sodium/hydrogen exchanger), isoform 9	SLC9A9
solute carrier organic anion transporter family, member 1a2	SLCO1A2
solute carrier organic anion transporter family, member 1b1	SLCO1B1
solute carrier organic anion transporter family, member 1b3	SLCO1B3
solute carrier organic anion transporter family, member 1c1	SLCO1C1
solute carrier organic anion transporter family, member 2a1	SLCO2A1
solute carrier organic anion transporter family, member 2b1	SLCO2B1
solute carrier organic anion transporter family, member 3a1	SLCO3A1
solute carrier organic anion transporter family, member 4a1	SLCO4A1
solute carrier organic anion transporter family, member 4C1	SLCO4C1
solute carrier organic anion transporter family, member 5A1	SLCO5A1
solute carrier organic anion transporter family, member 6A1	SLCO6A1
Major intrinsic protein of lens fiber	MIP

Niemann-Pick disease, type C1	NPC1
NPC1 (Niemann-Pick disease, type C1, gene)-like 1	NPC1L1
Niemann-Pick disease, type C2	NPC2
Phosphatidylcholine transfer protein	PCTP
Rh-associated glycoprotein	RHAG
Rh family, B glycoprotein	RHBG
Rh family, C glycoprotein	RHCG
Sideroflexin 1	SFXN1
Sideroflexin 2	SFXN2
Sideroflexin 3	SFXN3
Sideroflexin 4	SFXN4
Sideroflexin 5	SFXN5
Spinster homolog 1 (Drosophila)	SPNS1
steroidogenic acute regulatory protein	STAR
StAR-related lipid transfer (START) domain containing 10	STARD10
StAR-related lipid transfer (START) domain containing 13	STARD13
StAR-related lipid transfer (START) domain containing 3	STARD3
STARD3 N-terminal like	STARD3NL
StAR-related lipid transfer (START) domain containing 4	STARD4
StAR-related lipid transfer (START) domain containing 5	STARD5
StAR-related lipid transfer (START) domain containing 6	STARD6
StAR-related lipid transfer (START) domain containing 7	STARD7
StAR-related lipid transfer (START) domain containing 8	STARD8
StAR-related lipid transfer (START) domain containing 9	STARD9
Synaptic vesicle glycoprotein 2A	SV2A
Synaptic vesicle glycoprotein 2B	SV2B
Synaptic vesicle glycoprotein 2C	SV2C
SV2 related protein homolog (rat)	SVOP
ceramide kinase	CERK
ceramide kinase-like	CERKL
degenerative spermatocyte homolog 1, lipid desaturase (Drosop	DEGS1
degenerative spermatocyte homolog 2, lipid desaturase (Drosop	DEGS2
galactosylceramidase	GALC
LAG1 homolog, ceramide synthase 1	LASS1
LAG1 homolog, ceramide synthase 2	LASS2
LAG1 homolog, ceramide synthase 3	LASS3
LAG1 homolog, ceramide synthase 4	LASS4
LAG1 homolog, ceramide synthase 5	LASS5
LAG1 homolog, ceramide synthase 6	LASS6
sphingomyelin synthase 1	SGMS1
sphingomyelin synthase 2	SGMS2
sphingosine-1-phosphate lyase 1	SGPL1
sphingosine-1-phosphate phosphatase 1	SGPP1
sphingosine-1-phosphate phosphotase 2	SGPP2
sphingomyelin phosphodiesterase 1, acid lysosomal (acid sphin	SMPD1
sphingomyelin phosphodiesterase 2, neutral membrane (neutral	SMPD2
sphingomyelin phosphodiesterase 3, neutral membrane (neutral	SMPD3
sphingomyelin phosphodiesterase 4, neutral membrane (neutral	SMPD4

Sphingomyelin phosphodiesterase, acid-like 3A	SMPDL3A
sphingomyelin phosphodiesterase, acid-like 3B	SMPDL3B
sphingosine kinase 1	SPHK1
sphingosine kinase 2	SPHK2
serine palmitoyltransferase, long chain base subunit 1	SPTLC1
serine palmitoyltransferase, long chain base subunit 2	SPTLC2
serine palmitoyltransferase, long chain base subunit 3	SPTLC3
UDP-glucose ceramide glucosyltransferase	UGCG
UDP-glucose ceramide glucosyltransferase-like 1	UGCGL1
UDP-glucose ceramide glucosyltransferase-like 2	UGCGL2
UDP glycosyltransferase 8 (UDP-galactose ceramide galactosylt	UGT8
alkaline ceramidase 1	ACER1
alkaline ceramidase 2	ACER2
phytoceramidase, alkaline	ACER3
N-acylsphingosine amidohydrolase (acid ceramidase) 1	ASAH1
N-acylsphingosine amidohydrolase (non-lysosomal ceramidase)	ASAH2
N-acylsphingosine amidohydrolase (non-lysosomal ceramidase)	ASAH2B
N-acylsphingosine amidohydrolase (non-lysosomal ceramidase)	ASAH2C
N-acylsphingosine amidohydrolase (acid ceramidase)-like	ASAHL
3-ketodihydrosphingosine reductase	KDSR
aldo-keto reductase family 1, member C4 (chlordecone reductas	AKR1C4
aldo-keto reductase family 1, member D1 (delta 4-3-ketosteroid-	AKR1D1
cholesterol 25-hydroxylase	CH25H
cytochrome P450, family 11, subfamily A, polypeptide 1	CYP11A1
cytochrome P450, family 11, subfamily B, polypeptide 1	CYP11B1
cytochrome P450, family 11, subfamily B, polypeptide 2	CYP11B2
cytochrome P450, family 17, subfamily A, polypeptide 1	CYP17A1
cytochrome P450, family 19, subfamily A, polypeptide 1	CYP19A1
cytochrome P450, family 1, subfamily A, polypeptide 1	CYP1A1
cytochrome P450, family 1, subfamily A, polypeptide 2	CYP1A2
cytochrome P450, family 1, subfamily B, polypeptide 1	CYP1B1
cytochrome P450, family 21, subfamily A, polypeptide 2	CYP21A2
cytochrome P450, family 27, subfamily C, polypeptide 1	CYP27C1
cytochrome P450, family 2, subfamily A, polypeptide 13	CYP2A13
cytochrome P450, family 2, subfamily A, polypeptide 6	CYP2A6
cytochrome P450, family 2, subfamily A, polypeptide 7	CYP2A7
cytochrome P450, family 2, subfamily B, polypeptide 6	CYP2B6
cytochrome P450, family 2, subfamily C, polypeptide 18	CYP2C18
cytochrome P450, family 2, subfamily C, polypeptide 19	CYP2C19
cytochrome P450, family 2, subfamily C, polypeptide 8	CYP2C8
Cytochrome P450, family 2, subfamily C, polypeptide 9	CYP2C9
Cytochrome P450, family 2, subfamily D, polypeptide 6	CYP2D6
cytochrome P450, family 2, subfamily E, polypeptide 1	CYP2E1
cytochrome P450, family 2, subfamily F, polypeptide 1	CYP2F1
cytochrome P450, family 2, subfamily J, polypeptide 2	CYP2J2
cytochrome P450, family 2, subfamily R, polypeptide 1	CYP2R1
cytochrome P450, family 2, subfamily S, polypeptide 1	CYP2S1
cytochrome P450, family 2, subfamily U, polypeptide 1	CYP2U1

cytochrome P450, family 2, subfamily W, polypeptide 1	CYP2W1
cytochrome P450, family 39, subfamily A, polypeptide 1	CYP39A1
Cytochrome P450, family 3, subfamily A, polypeptide 4	CYP3A4
cytochrome P450, family 3, subfamily A, polypeptide 43	CYP3A43
cytochrome P450, family 3, subfamily A, polypeptide 5	CYP3A5
cytochrome P450, family 3, subfamily A, polypeptide 7	CYP3A7
cytochrome P450, family 46, subfamily A, polypeptide 1	CYP46A1
geranylgeranyl diphosphate synthase 1	GGPS1
hydroxysteroid (11-beta) dehydrogenase 1	HSD11B1
hydroxysteroid (11-beta) dehydrogenase 1-like	HSD11B1L
hydroxysteroid (11-beta) dehydrogenase 2	HSD11B2
hydroxysteroid (17-beta) dehydrogenase 1	HSD17B1
hydroxysteroid (17-beta) dehydrogenase 10	HSD17B10
hydroxysteroid (17-beta) dehydrogenase 11	HSD17B11
hydroxysteroid (17-beta) dehydrogenase 12	HSD17B12
short-chain dehydrogenase/reductase 9	HSD17B13
hydroxysteroid (17-beta) dehydrogenase 14	HSD17B14
hydroxysteroid (17-beta) dehydrogenase 2	HSD17B2
hydroxysteroid (17-beta) dehydrogenase 3	HSD17B3
Hydroxysteroid (17-beta) dehydrogenase 4	HSD17B4
hydroxysteroid (17-beta) dehydrogenase 6 homolog (mouse)	HSD17B6
hydroxysteroid (17-beta) dehydrogenase 8	HSD17B8
hydroxy-delta-5-steroid dehydrogenase, 3 beta- and steroid delta	HSD3B1
hydroxy-delta-5-steroid dehydrogenase, 3 beta- and steroid delta	HSD3B2
hydroxysteroid dehydrogenase like 1	HSDL1
hydroxysteroid dehydrogenase like 2	HSDL2
klotho	KL
klotho beta	KLB
Lipase A, lysosomal acid, cholesterol esterase (Wolman disease	LIPA
lipase, hormone-sensitive	LIPE
sterol O-acyltransferase (acyl-Coenzyme A: cholesterol acyltrans	SOAT1
sterol O-acyltransferase 2	SOAT2
steroid-5-alpha-reductase, alpha polypeptide 1 (3-oxo-5 alpha-st	SRD5A1
steroid-5-alpha-reductase, alpha polypeptide 2 (3-oxo-5 alpha-st	SRD5A2
steroid 5 alpha-reductase 2-like 2	SRD5A2L2
steroid 5 alpha-reductase 3	SRD5A3
acylglycerol kinase	AGK
amylo-1, 6-glucosidase, 4-alpha-glucanotransferase (glycogen d	AGL
amylase, alpha 1A (salivary)	AMY1A
amylase, alpha 1B (salivary)	AMY1B
amylase, alpha 1C (salivary)	AMY1C
amylase, alpha 2A (pancreatic)	AMY2A
amylase, alpha 2B (pancreatic)	AMY2B
Chromosome 9 ORF 103	C9ORF103
crystallin, lambda 1	CRYL1
fucose-1-phosphate guanylyltransferase	FPGT
fucokinase	FUK
glucosidase, alpha; acid (Pompe disease, glycogen storage dise	GAA

galactokinase 1	GALK1
galactokinase 2	GALK2
Galactose mutarotase (aldose 1-epimerase)	GALM
galactose-1-phosphate uridylyltransferase	GALT
glucose-fructose oxidoreductase domain containing 1	GFOD1
glucose-fructose oxidoreductase domain containing 2	GFOD2
glycerate kinase	GLYCTK
GDP-mannose 4,6-dehydratase	GMDS
GDP-mannose pyrophosphorylase A	GMPPA
GDP-mannose pyrophosphorylase B	GMPPB
glyoxylate reductase/hydroxypyruvate reductase	GRHPR
Hexose-6-phosphate dehydrogenase (glucose 1-dehydrogenase	H6PD
hydroxypyruvate isomerase homolog (E. coli)	HYI
ketohexokinase (fructokinase)	KHK
lactalbumin, alpha-	LALBA
lactase	LCT
lactase-like	LCTL
maltase-glucoamylase (alpha-glucosidase)	MGAM
mannose phosphate isomerase	MPI
phosphomannomutase 1	PMM1
phosphomannomutase 2	PMM2
phosphorylase, glycogen; brain	PYGB
phosphorylase, glycogen; liver (Hers disease, glycogen storage (	PYGL
phosphorylase, glycogen; muscle (McArdle syndrome, glycogen	PYGM
ribokinase	RBKS
sucrase-isomaltase (alpha-glucosidase)	SI
sialic acid acetylesterase	SIAE
sorbitol dehydrogenase	SORD
trehalase (brush-border membrane glycoprotein)	TREH
tissue specific transplantation antigen P35B	TSTA3
dermatan sulfate epimerase	DSE
dermatan sulfate epimerase-like	DSEL
B cell RAG associated protein	GALNAC4S-6ST
mercaptopyruvate sulfurtransferase	MPST
3'-phosphoadenosine 5'-phosphosulfate synthase 1	PAPSS1
3'-phosphoadenosine 5'-phosphosulfate synthase 2	PAPSS2
sulfiredoxin 1 homolog (S. cerevisiae)	SRXN1
steroid sulfatase (microsomal), isozyme S	STS
sulfotransferase family, cytosolic, 1A, phenol-preferring, membe	SULT1A1
sulfotransferase family, cytosolic, 1A, phenol-preferring, membe	SULT1A2
sulfotransferase family, cytosolic, 1A, phenol-preferring, membe	SULT1A3
sulfotransferase family, cytosolic, 1A, phenol-preferring, membe	SULT1A4
sulfotransferase family, cytosolic, 1B, member 1	SULT1B1
sulfotransferase family, cytosolic, 1C, member 2	SULT1C2
sulfotransferase family, cytosolic, 1C, member 3	SULT1C3
sulfotransferase family, cytosolic, 1C, member 4	SULT1C4
sulfotransferase family 1E, estrogen-preferring, member 1	SULT1E1
sulfotransferase family, cytosolic, 2A, dehydroepiandrosterone (I	SULT2A1

sulfotransferase family, cytosolic, 2B, member 1	SULT2B1
sulfotransferase family 4A, member 1	SULT4A1
sulfotransferase family, cytosolic, 6B, member 1	SULT6B1
thiosulfate sulfurtransferase (rhodanese)	TST
aminocarboxymuconate semialdehyde decarboxylase	ACMSD
arylformamidase	AFMID
3-hydroxyanthranilate 3,4-dioxygenase	HAAO
indoleamine 2,3-dioxygenase 1	IDO1
indoleamine-pyrrole 2,3 dioxygenase 2	IDO2
indolethylamine N-methyltransferase	INMT
kynurenine 3-monooxygenase (kynurenine 3-hydroxylase)	KMO
kynureninase (L-kynurenine hydrolase)	KYNU
tryptophan 2,3-dioxygenase	TDO2
fumarylacetoacetate hydrolase (fumarylacetoacetase)	FAH
fumarylacetoacetate hydrolase domain containing 1	FAHD1
fumarylacetoacetate hydrolase domain containing 2A	FAHD2A
fumarylacetoacetate hydrolase domain containing 2B	FAHD2B
homogentisate 1,2-dioxygenase (homogentisate oxidase)	HGD
4-hydroxyphenylpyruvate dioxygenase	HPD
4-hydroxyphenylpyruvate dioxygenase-like	HPDL
phenylalanine hydroxylase	PAH
tyrosine aminotransferase	TAT
chaperone, ABC1 activity of bc1 complex homolog (S. pombe)	CABC1
coenzyme Q10 homolog A (S. cerevisiae)	COQ10A
Coenzyme Q10 homolog B (S. cerevisiae)	COQ10B
coenzyme Q2 homolog, prenyltransferase (yeast)	COQ2
coenzyme Q3 homolog, methyltransferase (S. cerevisiae)	COQ3
coenzyme Q4 homolog (S. cerevisiae)	COQ4
coenzyme Q5 homolog, methyltransferase (S. cerevisiae)	COQ5
coenzyme Q6 homolog, monooxygenase (S. cerevisiae)	COQ6
coenzyme Q7 homolog, ubiquinone (yeast)	COQ7
coenzyme Q9 homolog (S. cerevisiae)	COQ9
prenyl (decaprenyl) diphosphate synthase, subunit 1	PDSS1
prenyl (decaprenyl) diphosphate synthase, subunit 2	PDSS2
sulfide quinone reductase-like (yeast)	SQRDL
agmatine ureohydrolase (agmatinase)	AGMAT
Arginase, liver	ARG1
arginase, type II	ARG2
argininosuccinate lyase	ASL
argininosuccinate synthetase 1	ASS1
carbamoyl-phosphate synthetase 1, mitochondrial	CPS1
ornithine carbamoyltransferase	OTC
beta carotene 15, 15 monooxygenase 1	BCMO1
beta-carotene dioxygenase 2	BCO2
cytochrome P450, family 26, subfamily A, polypeptide 1	CYP26A1
cytochrome P450, family 26, subfamily B, polypeptide 1	CYP26B1
cytochrome P450, family 26, subfamily C, polypeptide 1	CYP26C1
lecithin retinol acyltransferase (phosphatidylcholine--retinol O-ac	LRAT

retinol dehydrogenase 10 (all-trans)	RDH10
retinol dehydrogenase 11 (all-trans/9-cis/11-cis)	RDH11
retinol dehydrogenase 12 (all-trans/9-cis/11-cis)	RDH12
retinol dehydrogenase 13 (all-trans/9-cis)	RDH13
retinol dehydrogenase 14 (all-trans/9-cis/11-cis)	RDH14
retinol dehydrogenase 16 (all-trans)	RDH16
retinol dehydrogenase 5 (11-cis/9-cis)	RDH5
retinol dehydrogenase 8 (all-trans)	RDH8
retinol saturase (all-trans-retinol 13,14-reductase)	RETSAT
retinal pigment epithelium-specific protein 65kDa	RPE65
short chain dehydrogenase/reductase family 16C, member 5	SDR16C5
pyridoxal (pyridoxine, vitamin B6) kinase	PDXK
pyridoxal (pyridoxine, vitamin B6) phosphatase	PDXP
phosphatase orphan 2	PHOSPHO2
pyridoxamine 5'-phosphate oxidase	PNPO
dicarbonyl/L-xylulose reductase	DCXR
xylulokinase homolog (H. influenzae)	XYLB
