# Supplementary material for: Exploring T-cell metabolism in tuberculosis: development of a diagnostic model using metabolic genes
Source: Eur J Med Res. 2025 Jun 16;30:483. doi: 10.1186/s40001-025-02768-0 (PMC12168305; doi:10.1186/s40001-025-02768-0)
Supplement: Supplementary file 4 — Supplementary Material 4 [file 40001_2025_2768_MOESM4_ESM.pdf]

	logFC	AveExpr	t	P.Value	adj.P.Val	B	change
CACNG6	-1.45361	6.9286055	-7.64969	1.5243983	4.7907266	15.711293	down
ITPKB	-0.7526	9.4232391	-7.00798	3.2915230	5.1721347	12.851823	down
OLIG2	-1.96084	6.9652346	-6.90728	5.2952247	5.5471009	12.409122	down
RNF144	-0.92064	9.4315565	-6.76308	1.0423469	5.5981942	11.778500	down
PRSS33	-2.35523	6.2163833	-6.76297	1.0428729	5.5981942	11.778030	down
SIGLEC8	-1.21645	6.0075678	-6.73901	1.1666292	5.5981942	11.673612	down
GPR44	-2.24835	6.9115984	-6.72476	1.2469328	5.5981942	11.611627	down
SPNS3	-1.11026	7.5101439	-6.60896	2.1388180	7.6389952	11.109224	down
RBL2	-0.65626	11.857536	-6.6041	2.1876398	7.6389952	11.088209	down
ALOX15	-1.44164	6.8336290	-6.56128	2.6684900	8.3862635	10.903213	down
RPS6KA2	-1.08797	7.1083681	-6.50584	3.4489788	9.8537326	10.664339	down
EPHA1	-0.94457	7.3436196	-6.32598	7.8862549	1.9064717	9.8944267	down
KLHL22	-0.65858	10.025642	-6.26929	1.0217067	2.2935127	9.6534179	down
MARCKSL1	-0.97765	10.760507	-6.11403	2.0668377	4.3303007	8.9977958	down
SOX8	-0.77362	7.4308625	-5.99536	3.5246662	6.9231054	8.5012617	down
RNF216	-0.63473	9.4752303	-5.97496	3.8618492	6.9662796	8.4162911	down
ZCWPW1	-0.51648	8.3538378	-5.96766	3.9899778	6.9662796	8.3859357	down
INPP5B	-0.53896	7.8093766	-5.91654	5.0126411	8.2911723	8.1737464	down
FHIT	-1.1124	7.2557796	-5.87933	5.9153643	8.8524836	8.0197819	down
MOAP1	-0.58326	10.212348	-5.82381	7.5669782	0.0001033	7.7908674	down
U2AF1L4	-0.60799	7.6582010	-5.80558	8.2026185	0.0001055	7.7158937	down
LDLRAP1	-1.04103	8.8927247	-5.80034	8.3946465	0.0001055	7.6943852	down
EEF2K	-0.71229	8.3721160	-5.74974	1.0493193	0.0001203	7.4870149	down
CHMP7	-0.73913	7.8717530	-5.74491	1.0718768	0.0001203	7.4672502	down
TLE2	-0.84305	6.4209493	-5.71549	1.2198612	0.0001205	7.3470826	down
LOC391157	-0.88425	6.7852902	-5.7045	1.2801294	0.0001205	7.3022776	down
FOXO1	-0.73461	10.011778	-5.70274	1.2900394	0.0001205	7.2951129	down
SUSD3	-0.77328	10.064000	-5.69962	1.3078007	0.0001205	7.2824089	down
NELL2	-1.34688	9.3803194	-5.69551	1.3315993	0.0001205	7.2656545	down
ARHGEF18	-0.6794	12.783865	-5.69246	1.3495330	0.0001205	7.2532260	down
DBP	-0.80705	7.3460108	-5.68928	1.3684447	0.0001205	7.2402975	down
RNF144A	-0.70216	8.6979603	-5.68728	1.3804860	0.0001205	7.2321586	down
HRK	-1.79216	8.0658954	-5.64451	1.6644640	0.0001393	7.0583889	down
STMN3	-1.16493	11.473008	-5.64174	1.6846679	0.0001393	7.0471825	down
EMR4P	-1.30444	7.2429317	-5.63078	1.7672321	0.0001407	7.0027439	down
TOMM20	-0.60051	10.063413	-5.62508	1.8116573	0.0001407	6.9796854	down
CACNA1H	-0.54231	6.6650258	-5.62199	1.8362239	0.0001407	6.9671762	down
RALA	-0.50734	10.957360	-5.58924	2.1174966	0.0001511	6.8348230	down
CABC1	-0.60562	9.4645571	-5.58752	2.1334181	0.0001511	6.8278672	down
FAM102A	-1.3273	8.0234758	-5.58525	2.1545385	0.0001511	6.8187199	down
LFNG	-0.74896	12.709859	-5.58424	2.1640189	0.0001511	6.8146431	down
SLC16A10	-1.51223	6.9158403	-5.57212	2.2810010	0.0001558	6.7657593	down
EDAR	-1.04538	7.1718440	-5.54935	2.5176493	0.0001621	6.6741129	down
C1QB	2.4183803	9.5363862	5.5461963	2.5523321	0.0001621	6.6614112	up
SLC7A6	-0.93646	9.2560962	-5.54235	2.5952350	0.0001621	6.6459360	down
RSL1D1	-0.58487	11.052827	-5.53678	2.6584761	0.0001621	6.6235857	down
C16orf30	-1.37655	9.1093476	-5.52737	2.7690384	0.0001621	6.5857607	down
FLNB	-0.99141	8.5376584	-5.5264	2.7806054	0.0001621	6.5818912	down
FAM171A1	-0.62314	8.6124733	-5.52203	2.8336207	0.0001621	6.5643598	down
C10orf33	-1.23681	7.9368182	-5.51531	2.9170882	0.0001621	6.5374130	down

ADORA3	-1.16055	6.9184999	-5.51503	2.9206418	0.0001621	6.5362829		down
PLEKHB1	-0.63272	7.6799137	-5.51347	2.9404390	0.0001621	6.5300125		down
ELL2	0.8505437	8.8052632	5.5015468	3.0957437	0.0001677	6.4822407		up
TMEM204	-1.242	8.9283206	-5.49244	3.2197927	0.0001696	6.4457765		down
CYTH1	-0.51698	11.261524	-5.48947	3.2612987	0.0001696	6.4338892		down
ATPGD1	-0.67286	7.3324763	-5.48726	3.2924850	0.0001696	6.4250568		down
LEPROTL1	-0.75491	11.407090	-5.47538	3.4653240	0.0001756	6.3775757		down
NMT2	-1.04928	8.7265487	-5.45881	3.7213356	0.0001856	6.3114349		down
SLC29A1	-1.35534	7.2391542	-5.43531	4.1166047	0.0001996	6.2177732		down
LPHN1	-0.75212	7.0169521	-5.43445	4.1319024	0.0001996	6.2143319		down
RNU6-1	0.8875481	11.531811	5.4302013	4.2078030	0.0001996	6.1974442		up
H2AFJ	1.1693000	11.564481	5.4239963	4.3212469	0.0001996	6.1727636		up
SERTAD2	-0.58123	11.133081	-5.42091	4.3787210	0.0001996	6.1605061		down
EIF4B	-0.73879	12.755994	-5.42063	4.3840313	0.0001996	6.1593818		down
PITPNC1	-0.68298	9.6389658	-5.41288	4.5320311	0.0002034	6.1285817		down
GAL3ST4	-0.58081	7.8355545	-5.36958	5.4534127	0.0002346	5.9569240		down
GIMAP5	-0.9184	11.699106	-5.36647	5.5260808	0.0002346	5.9446480		down
FAM190B	-0.56889	9.6705024	-5.35387	5.8310899	0.0002443	5.8948267		down
LOC4007591	1.3721842	8.5201795	5.3421072	6.1304550	0.0002472	5.8484032		up
MFNG	-0.66171	11.650836	-5.33891	6.2144046	0.0002472	5.8357921		down
CD96	-1.01635	10.193365	-5.32875	6.4888848	0.0002549	5.7957188		down
IL23A	-0.97498	6.9275427	-5.32274	6.6565841	0.0002582	5.7720624		down
PSCD1	-0.53376	12.910488	-5.31161	6.9787908	0.0002674	5.7282404		down
PPP1R2	-0.61492	10.684485	-5.30637	7.1355797	0.0002677	5.7076440		down
VPREB1	-1.45712	5.7790453	-5.30571	7.1554456	0.0002677	5.7050667		down
C12orf41	-0.54008	10.612617	-5.30103	7.2989629	0.0002698	5.6866581		down
TRAF3IP3	-0.65827	10.573748	-5.28228	7.9021370	0.0002795	5.6130604		down
NOSIP	-0.73465	12.698982	-5.28192	7.9142094	0.0002795	5.6116455		down
DDX18	-0.57377	11.045615	-5.27196	8.2546643	0.0002795	5.5726093		down
FAM117B	-0.90314	11.327324	-5.27133	8.2768738	0.0002795	5.5701191		down
SSBP2	-0.61356	9.0687336	-5.26968	8.3347440	0.0002795	5.5636618		down
ACACB	-0.94408	7.2311113	-5.26664	8.4425769	0.0002795	5.5517485		down
HPCAL4	-0.72418	7.3674020	-5.26473	8.5107751	0.0002795	5.5442925		down
RPL15	-0.76464	12.785850	-5.26463	8.5146560	0.0002795	5.5438700		down
UNC84A	-0.64161	8.9801517	-5.2601	8.6790600	0.0002795	5.5261471		down
DIMT1L	-0.53311	10.574634	-5.25871	8.7302847	0.0002795	5.5206937		down
RPL22	-0.89384	11.878120	-5.25848	8.7385050	0.0002795	5.5198215		down
ZNF540	-0.87254	6.8068690	-5.25844	8.7400970	0.0002795	5.5196527		down
TBC1D10C	-0.69047	12.201076	-5.25662	8.8073031	0.0002795	5.5125543		down
COG2	-0.50414	9.1368201	-5.25002	9.0560995	0.0002814	5.4867401		down
)C1001316	-0.99281	6.7417141	-5.24795	9.1355792	0.0002814	5.4786431		down
CRTC3	-0.6451	10.593280	-5.24794	9.1359770	0.0002814	5.4786028		down
TYSND1	-0.62672	9.9477068	-5.2381	9.5231217	0.0002855	5.4401468		down
VEGFB	-0.62925	8.1906444	-5.23768	9.5397710	0.0002855	5.4385284		down
CLCF1	-0.7193	7.3783662	-5.22714	9.9729922	0.0002925	5.3973805		down
ID3	-1.43857	9.1481637	-5.22669	9.9919084	0.0002925	5.3956249		down
TCF7	-1.28517	8.4611727	-5.22435	1.0090979	0.0002925	5.3864840		down
FAM62B	-0.69598	12.163725	-5.20516	1.0938666	0.0003069	5.311759		down
)C1001331	-0.55134	8.6740252	-5.19375	1.1475521	0.0003191	5.2673781		down
CCRL2	0.8521359	7.4765107	5.1837861	1.1965454	0.0003298	5.2286553		up
TOP2B	-0.56795	11.463480	-5.14134	1.4290739	0.0003806	5.0642037		down

ZNF792	-0.71044	7.9939796	-5.13057	1.4947334	0.0003914	5.0226141	down
PRAGMIN	-0.99506	10.558147	-5.12697	1.5173985	0.0003916	5.0086817	down
LRRN3	-2.00184	9.4931461	-5.1265	1.5203388	0.0003916	5.0068895	down
CD40LG	-1.3307	7.7750961	-5.10926	1.6335624	0.0004145	4.9403967	down
C2orf44	-0.53152	9.2117352	-5.10311	1.6759037	0.0004182	4.9167109	down
NOG	-0.88233	6.5620477	-5.10297	1.6768295	0.0004182	4.9162	down
C11orf2	-0.73772	11.439663	-5.0997	1.6998028	0.0004206	4.9036051	down
SMPD3	-0.65617	6.6157533	-5.09738	1.7162919	0.0004213	4.8946700	down
SFRS6	-0.5534	12.581041	-5.09302	1.7476445	0.0004257	4.8779154	down
CACNA2D3	-1.29688	6.5287591	-5.08431	1.8120481	0.0004336	4.8444236	down
SNHG6	-0.67862	11.716647	-5.08306	1.8215143	0.0004336	4.8396016	down
LEF1	-1.31632	11.572135	-5.07813	1.8591383	0.0004388	4.8206817	down
CRIP2	-0.98054	6.0630040	-5.0766	1.8709849	0.0004388	4.8148038	down
QARS	-0.66404	12.596202	-5.07172	1.9091996	0.0004422	4.7960938	down
GLTSCR2	-0.68032	14.254872	-5.06938	1.9278244	0.0004422	4.7871107	down
CD28	-0.99229	7.9421944	-5.06728	1.9446896	0.0004428	4.7790509	down
TTC9	-0.73889	6.8525316	-5.06166	1.9905246	0.0004458	4.7574957	down
MEGF6	-0.82969	6.9990988	-5.05762	2.0240882	0.0004458	4.7420246	down
FZp761P04	-0.83355	9.1707838	-5.05472	2.0485322	0.0004458	4.7309180	down
C1QC	1.9821902	8.1335424	5.0532665	2.0608845	0.0004458	4.7253560	up
EFHD1	-0.61813	5.7930842	-5.05259	2.0666567	0.0004458	4.7227683	down
PAFAH2	-0.65608	9.3728994	-5.05207	2.0711257	0.0004458	4.7207698	down
RASSF7	-0.68516	10.210764	-5.04042	2.1733573	0.0004618	4.6761959	down
APRT	-0.65034	10.167958	-5.04025	2.1748775	0.0004618	4.6755490	down
ZNF395	-0.8434	10.181172	-5.03752	2.1995281	0.0004639	4.6651229	down
KIAA1618	0.7438217	8.6263624	5.0350622	2.2219889	0.0004655	4.6557245	up
MAN1C1	-1.29158	7.5587547	-5.03192	2.2509718	0.0004684	4.6437366	down
PAQR8	-0.79029	9.6453191	-5.02005	2.3639910	0.0004855	4.5984229	down
LOC642741	-0.68435	14.070172	-5.01622	2.4016955	0.0004873	4.5837875	down
BCAS4	-0.77726	9.5157462	-5.016	2.4038766	0.0004873	4.5829479	down
IL7R	-1.19485	12.778091	-5.01217	2.4421133	0.0004892	4.5683524	down
EHBP1	-0.5186	8.4474036	-4.99474	2.6237677	0.0005153	4.5020012	down
OLIG1	-1.25013	8.3075933	-4.98848	2.6922211	0.0005231	4.4781857	down
C18orf1	-0.63726	6.0997934	-4.98584	2.7215975	0.0005247	4.4681510	down
LOC648980	-0.51876	8.9933469	-4.97932	2.7955056	0.0005324	4.4433770	down
CLC	-1.98998	11.741713	-4.97542	2.8405901	0.0005332	4.4285850	down
PLEKHG4	-0.62559	7.0205564	-4.97455	2.8507573	0.0005332	4.4252818	down
HIST2H2AC	1.2787715	11.357403	4.9693401	2.9123452	0.0005383	4.4055211	up
MFGE8	-0.88606	8.6665708	-4.96181	3.0037223	0.0005520	4.3769611	down
ACOT11	-0.76136	5.6749002	-4.96027	3.0226445	0.0005522	4.3711559	down
AP2A2	-0.50873	7.7002945	-4.94945	3.1596311	0.0005698	4.3301848	down
ESYT1	-0.83061	11.910964	-4.94899	3.1656195	0.0005698	4.3284346	down
FBL	-0.77501	11.039231	-4.9484	3.1732738	0.0005698	4.3262024	down
SPOCK2	-1.05356	12.042633	-4.93939	3.2924368	0.0005806	4.2921298	down
HSPA8	-0.65034	13.106334	-4.9378	3.3139321	0.0005806	4.2861154	down
BCL11B	-1.11415	11.368408	-4.93692	3.3258900	0.0005806	4.2827865	down
RNU6-15	0.8438632	11.551548	4.9350030	3.3520475	0.0005820	4.2755462	up
C10orf35	-0.68275	6.7062056	-4.93059	3.4130256	0.0005878	4.2588853	down
ZNF837	-0.52075	7.0724039	-4.92859	3.4410285	0.0005878	4.2513339	down
PIK3IP1	-1.0701	11.738049	-4.92853	3.4418695	0.0005878	4.2511081	down
C9orf123	-0.93442	8.4758807	-4.92088	3.5511100	0.0006000	4.2222340	down

HIST2H2AA3	1.3448749	11.633511	4.9125893	3.6732557	0.0006140	4.1909850	up
FLT3LG	-0.92172	9.4399323	-4.90803	3.7421008	0.0006222	4.1738279	down
C21orf2	-0.7655	8.3643488	-4.90608	3.7719710	0.0006236	4.1664821	down
EIF3D	-0.50975	12.248771	-4.90489	3.7902898	0.0006236	4.1620058	down
HIST2H2AB	0.9586934	6.6058316	4.8851790	4.1069638	0.0006585	4.0878759	up
LOC729742	-0.6781	12.659864	-4.88236	4.1542237	0.0006627	4.0773070	down
TDP1	-0.71306	7.6340395	-4.86655	4.4298358	0.0006932	4.0179750	down
LOC645173	-0.94391	12.949719	-4.86619	4.4361728	0.0006932	4.0166548	down
CCR9	-0.68058	8.0122166	-4.86511	4.4557155	0.0006932	4.0125951	down
TBC1D4	-0.9583	8.4092246	-4.86113	4.5281481	0.0006975	3.9977027	down
SNHG7	-0.7418	10.005336	-4.84967	4.7434450	0.0007271	3.9548073	down
RPL12	-0.6126	13.477101	-4.84423	4.8490906	0.0007361	3.9344674	down
EPHB6	-0.81956	7.4513183	-4.83741	4.9848100	0.0007495	3.9089799	down
LTB	-0.89335	11.674341	-4.83473	5.0391223	0.0007541	3.8989747	down
LOC286444	-0.73336	13.868316	-4.82148	5.3161931	0.0007880	3.8495599	down
LOC729679	-0.88343	12.801087	-4.8174	5.4045264	0.0007972	3.8343475	down
DHRS3	-0.93839	8.2435660	-4.81629	5.4286422	0.0007972	3.8302377	down
ZBTB42	-0.65072	8.1641411	-4.81311	5.4987455	0.0008037	3.8183939	down
PPP1R13B	-0.79929	7.4335245	-4.8111	5.5434337	0.0008046	3.8109226	down
DNAJC8	-0.50354	12.265020	-4.81053	5.5562222	0.0008046	3.8087957	down
POLE3	-0.54996	11.745532	-4.80569	5.6657589	0.0008093	3.7907764	down
SIRT4	-0.81213	6.6860698	-4.80148	5.7627025	0.0008194	3.7751177	down
ZCCHC14	-0.77545	7.5875225	-4.79727	5.8612154	0.0008277	3.7594739	down
KLHL3	-1.13034	8.5530655	-4.79675	5.8734446	0.0008277	3.7575503	down
LOC6525780	0.9069959	8.2228719	4.7951189	5.9121737	0.0008294	3.7514848	up
C2orf89	-1.29714	9.2280588	-4.78891	6.0617943	0.0008449	3.7284208	down
B3GALTL	-0.71786	6.6742294	-4.78831	6.0764970	0.0008449	3.7261853	down
C1orf93	-0.6845	7.313343	-4.7804	6.2726017	0.0008657	3.6968756	down
BAT2L	-0.55684	7.5609669	-4.77917	6.3037106	0.0008657	3.6923107	down
MRFAP1L1	-0.57402	11.443878	-4.779	6.3081758	0.0008657	3.6916573	down
LOC6440860	0.6848258	6.6562732	4.7766290	6.3684638	0.0008701	3.6828808	up
RCAN3	-0.81571	7.0623754	-4.77426	6.4292532	0.0008709	3.6741153	down
TCN2	1.0477307	7.9345655	4.7720111	6.4876587	0.0008713	3.6657715	up
MORC2	-0.66181	7.9258213	-4.76856	6.5782043	0.0008713	3.6529838	down
RNU4-2	0.9235492	8.8822962	4.7649014	6.6754168	0.0008713	3.6394496	up
C22orf32	-0.72364	7.9324589	-4.76224	6.7470426	0.0008713	3.6296035	down
C20orf100	-0.79168	9.3302355	-4.76138	6.7704062	0.0008713	3.6264145	down
DDX46	-0.50576	9.1275013	-4.75958	6.8193653	0.0008713	3.6197674	down
RPL13A	-0.84956	13.462604	-4.75931	6.8268223	0.0008713	3.6187592	down
TMC6	-0.51082	10.314544	-4.75784	6.8671291	0.0008713	3.6133286	down
GCET2	-0.78825	6.9486316	-4.75751	6.8760994	0.0008713	3.6121243	down
LOC93622	-0.66833	8.6183517	-4.75482	6.9506428	0.0008737	3.6021776	down
LOC650369	-0.55414	11.560903	-4.75112	7.0546563	0.0008782	3.5884758	down
GOLGA7B	-0.69358	6.1281124	-4.75058	7.0698826	0.0008782	3.5864871	down
)C1001327	-0.67338	12.690981	-4.74911	7.1116395	0.0008799	3.5810551	down
IFITM3	1.1765864	14.616020	4.7473143	7.1628364	0.0008827	3.5744385	up
LILRA5	1.2271926	9.3897037	4.7444426	7.2456379	0.0008860	3.5638371	up
S100A12	1.9888208	10.749905	4.7427396	7.2951814	0.0008886	3.5575519	up
FLJ43093	-1.06093	7.7765513	-4.7413	7.3373390	0.0008903	3.5522372	down
SQRDL	0.7986504	9.5858041	4.7373604	7.4538386	0.0009009	3.5377083	up
TTC3	-0.65887	11.195282	-4.73449	7.5398540	0.0009037	3.5271264	down

LOC284023	-0.63793	8.2560341	-4.73233	7.6051016	0.0009037	3.5191800	down
RNU1F1	0.7138602	10.043383	4.7298452	7.6811230	0.0009037	3.5100072	up
FAM44B	-0.599	8.8249100	-4.72968	7.6861983	0.0009037	3.5093980	down
RPL4	-0.70632	13.442867	-4.72901	7.7067056	0.0009037	3.5069408	down
GPR84	1.5626394	8.0244505	4.7277013	7.7471835	0.0009050	3.5021099	up
LOC644511	-0.77594	12.801385	-4.72082	7.9628567	0.0009268	3.4767892	down
PDE7B	-0.94717	6.6542403	-4.71667	8.0958890	0.0009348	3.4615116	down
CBX7	-0.52283	10.747028	-4.71534	8.1389919	0.0009348	3.4566155	down
EEPD1	-0.62925	6.8837421	-4.71461	8.1627742	0.0009348	3.4539252	down
LYRM4	-0.70201	7.6298905	-4.71331	8.2049792	0.0009348	3.4491702	down
FBLN5	-0.76296	7.0675217	-4.71216	8.2429952	0.0009352	3.4449081	down
RICS	-0.70634	8.0544946	-4.70689	8.4178145	0.0009448	3.4255588	down
EPHX2	-0.88892	8.8967644	-4.69863	8.6991593	0.0009716	3.3952498	down
HIST2H2AA	1.2467910	12.005676	4.6968044	8.7627438	0.0009730	3.3885362	up
TSHZ1	-0.52308	8.7382419	-4.69125	8.9584204	0.0009809	3.3681780	down
ATG9B	-0.59703	5.9638993	-4.68489	9.1878840	0.0010025	3.3448652	down
ZBTB9	-0.61766	8.7949050	-4.68015	9.3626657	0.0010181	3.3274964	down
LOC731096	-0.61036	14.073564	-4.67695	9.4823920	0.0010269	3.3157854	down
SLC41A3	-0.63869	9.1674896	-4.67623	9.5094708	0.0010269	3.3131573	down
MUM1	-0.63448	8.6869953	-4.67302	9.6312047	0.0010365	3.3014344	down
CASK	-0.5232	7.4775023	-4.66896	9.7877278	0.0010458	3.2865778	down
KIAA0355	-0.62317	9.3401475	-4.66509	9.9391797	0.0010518	3.2724277	down
LOC645688	-0.63879	12.115922	-4.66505	9.9407941	0.0010518	3.2722780	down
Septin 6	-0.77196	10.717294	-4.66378	9.9908336	0.0010536	3.2676511	down
LRIG1	-0.6703	8.3049451	-4.66046	1.0123312	0.0010577	3.2555127	down
RPA2	-0.50208	11.753697	-4.66027	1.0131047	0.0010577	3.2548089	down
HIST1H1C	1.1863585	11.333446	4.6470764	1.0674121	0.0011004	3.2066970	up
C2orf15	-0.52992	6.8454902	-4.64568	1.0733071	0.0011008	3.2016230	down
LOC643433	-0.59365	12.897462	-4.64521	1.0753432	0.0011008	3.1998770	down
HSF2	-0.59947	7.9710154	-4.6371	1.1103541	0.0011292	3.1703616	down
ZZZ3	-0.62252	8.9677762	-4.63185	1.1336322	0.0011492	3.1512496	down
APEX1	-0.60015	11.545567	-4.63037	1.1402761	0.0011522	3.1458669	down
EXD2	-0.52267	7.7609970	-4.62945	1.1444062	0.0011527	3.1425367	down
FBLN7	-0.57534	7.3694675	-4.62691	1.1559550	0.0011606	3.1332881	down
GSTM3	-0.70467	6.8600830	-4.62533	1.1631937	0.0011641	3.1275384	down
MMP8	2.3567660	6.2424724	4.6225359	1.1760694	0.0011733	3.1173992	up
LOC441013	-0.64947	14.345394	-4.6194	1.1906938	0.0011841	3.1060173	down
IFI27	2.4911239	13.254545	4.6162794	1.2054424	0.0011950	3.0946797	up
C14orf64	-0.76115	6.3382515	-4.61478	1.2125656	0.0011969	3.0892537	down
RLTPR	-0.78095	6.8486151	-4.61063	1.2325630	0.0012066	3.0741902	down
FAM43A	-0.63268	9.0887266	-4.60985	1.2363558	0.0012066	3.0713609	down
HNRNPU	-0.51871	8.3508684	-4.60618	1.2543756	0.0012174	3.058037	down
DHDDS	0.5768181	9.0903455	4.6060310	1.2550985	0.0012174	3.0575060	up
TNFRSF25	-1.20708	9.7583223	-4.60271	1.2715928	0.0012248	3.0454837	down
EBI2	-1.13305	10.609158	-4.60219	1.2742381	0.0012248	3.0435702	down
VIPR1	-0.6319	8.7250136	-4.60215	1.2744375	0.0012248	3.0434261	down
OC1001279	-0.55389	13.313171	-4.59324	1.3198796	0.0012503	3.0111678	down
KLF9	-0.56663	10.281940	-4.59304	1.3208973	0.0012503	3.0104582	down
SNORD13	-1.14704	10.467671	-4.59083	1.3324425	0.0012552	3.0024462	down
IMMP2L	-0.62538	6.9000294	-4.59051	1.3340871	0.0012552	3.0013105	down
POLR1E	-0.61856	7.8240570	-4.5821	1.3788728	0.0012782	2.9709133	down

FAM134B	-0.81688	7.7426775	-4.58031	1.3885874	0.0012835	2.9644506	down
LDLR	0.6277082	10.181246	4.5784376	1.3988502	0.0012891	2.9576723	up
TCEA2	-0.59355	8.1432654	-4.57221	1.4334371	0.0013095	2.9351905	down
LOC648249	-0.74536	12.794158	-4.56944	1.4490806	0.0013146	2.9252002	down
C1orf71	-0.50037	11.928123	-4.56922	1.4503575	0.0013146	2.9243895	down
LMNB1	0.9945194	10.143324	4.5652324	1.4731664	0.0013216	2.9100281	up
FAIM3	-1.06695	12.675279	-4.56375	1.4817714	0.0013216	2.9046680	down
APBA2	-0.8379	6.6320108	-4.56308	1.4856482	0.0013216	2.9022632	down
ZFP90	-0.58805	9.7223674	-4.56182	1.4929682	0.0013216	2.8977399	down
DBH	-0.53707	5.3644451	-4.5587	1.5113448	0.0013305	2.8864815	down
ATP5G2	-0.5614	12.659775	-4.55867	1.5115065	0.0013305	2.8863830	down
ZNF329	-0.69879	8.3146413	-4.54451	1.5975391	0.0013792	2.8354444	down
HOOK1	-0.70146	7.8497073	-4.54212	1.6125473	0.0013833	2.8268411	down
CD7	-0.93267	11.539079	-4.54164	1.6155587	0.0013833	2.8251245	down
KLHDC8B	1.0149848	9.0625109	4.5342441	1.6628821	0.0014028	2.7985625	up
LOC283412	-0.56097	13.160832	-4.53391	1.6650227	0.0014028	2.7973791	down
PCSK5	-0.5574	8.8216090	-4.53322	1.6695652	0.0014029	2.7948726	down
RPS4X	-0.75425	13.737795	-4.53175	1.6791333	0.0014040	2.7896156	down
TFAP4	-0.53736	7.1262760	-4.53164	1.6798691	0.0014040	2.7892126	down
CCR7	-1.37384	12.058061	-4.53051	1.6872873	0.0014055	2.7851592	down
LOC730316	-0.50935	11.231967	-4.52709	1.7099098	0.0014178	2.772908	down
PASK	-0.69533	9.3765244	-4.52001	1.7577346	0.0014445	2.7475339	down
CD5	-1.09601	9.5710749	-4.51828	1.7696532	0.0014445	2.7413183	down
ICOS	-1.00097	9.2776060	-4.512	1.8133954	0.0014725	2.7188610	down
HIST1H3D	0.9831306	7.8646471	4.5094109	1.8317830	0.0014808	2.7095827	up
C22orf29	-0.59168	7.7240906	-4.50924	1.8330094	0.0014808	2.7089672	down
CCL23	-1.12962	6.5187323	-4.50756	1.8450441	0.0014867	2.7029490	down
TUBGCP6	-0.64565	8.8554316	-4.5055	1.8598341	0.0014948	2.6956067	down
CEACAM6	2.0063350	7.9159253	4.5029122	1.8786534	0.0015061	2.6863483	up
OSBPL6	0.5733996	5.1929243	4.5004731	1.8965421	0.0015164	2.6776337	up
CD47	-0.51126	11.290995	-4.49984	1.9011969	0.0015164	2.6753796	down
NOV	-0.95232	7.1681572	-4.49861	1.9103130	0.0015166	2.6709811	down
LY9	-0.73174	8.7566728	-4.49851	1.9110716	0.0015166	2.6706160	down
SNORD89	0.6497533	10.132549	4.4891803	1.9815372	0.0015607	2.6373238	up
CAMK4	-0.80174	7.3035664	-4.48854	1.9864912	0.0015607	2.6350281	down
ZNF581	-0.55307	10.986168	-4.48272	2.0317855	0.0015907	2.6143013	down
LILRB2	0.7537794	7.1668735	4.4823398	2.0348003	0.0015907	2.6129383	up
HSF5	-0.67581	6.7983163	-4.47683	2.0787474	0.0016130	2.5932955	down
LOC347544	-0.68861	13.253912	-4.47342	2.1063394	0.0016264	2.5811747	down
AOAH	0.5669059	9.9622046	4.4708650	2.1272727	0.0016275	2.5720848	up
DDX51	-0.59631	7.4117386	-4.47025	2.1323298	0.0016275	2.5699023	down
LOC649447	-0.53807	13.334557	-4.4701	2.1336136	0.0016275	2.5693491	down
EMR4	-0.63347	6.8082523	-4.46851	2.1467818	0.0016325	2.5636939	down
MOBKL1B	0.6789950	9.7463418	4.4680503	2.1505688	0.0016325	2.5620739	up
BEX4	-0.66119	8.9271554	-4.46405	2.1841004	0.0016535	2.5478540	down
C16orf74	-0.78552	6.5862766	-4.46349	2.1888732	0.0016535	2.5458478	down
SLC22A4	1.2196240	9.8741397	4.4571401	2.2432205	0.0016825	2.5233083	up
ZNF266	-0.5645	9.6224374	-4.45634	2.2501650	0.0016837	2.5204678	down
RBM14	-0.52916	10.955817	-4.45394	2.2711367	0.0016913	2.5119428	down
HIST1H4H	1.1647671	8.9696679	4.4464087	2.3381103	0.0017289	2.4852376	up
LOC646294	-0.65466	13.944453	-4.44541	2.3471371	0.0017315	2.4816971	down

MRPS6	-0.56869	12.234272	-4.44134	2.3843131	0.0017548	2.4672585	down
RBM15B	-0.54424	7.3037891	-4.43922	2.4038569	0.0017568	2.4597584	down
ACTA2	0.9215732	9.5245618	4.4382557	2.4127832	0.0017593	2.4563531	up
ADAM23	-0.92244	5.6807836	-4.43293	2.4627715	0.0017884	2.4375141	down
SNPH	-0.51949	7.0214580	-4.43092	2.4819505	0.0017972	2.4303878	down
)C1001317	-0.52619	14.329106	-4.427	2.5196747	0.0018120	2.4165307	down
FAM168B	-0.53911	8.9153555	-4.42531	2.5360951	0.0018177	2.4105640	down
TIGA1	-0.6015	10.686625	-4.42441	2.5449086	0.0018177	2.4073773	down
CD63	0.6737414	11.199813	4.4222491	2.5661435	0.0018287	2.3997449	up
SF3A3	-0.61723	11.049354	-4.42066	2.5818250	0.0018357	2.3941491	down
HPSE	0.9727722	9.6418811	4.4138352	2.6504901	0.0018682	2.3700418	up
C5orf13	-0.6839	8.8976029	-4.41343	2.6545821	0.0018682	2.3686250	down
ITK	-0.85573	11.658224	-4.4113	2.6763787	0.0018722	2.3611151	down
LOC730029	-0.66243	12.847476	-4.40932	2.6968539	0.0018722	2.3541162	down
HSZFP36	-0.87687	7.5453577	-4.40799	2.7106205	0.0018722	2.3494404	down
LOC653881	-0.59595	14.060478	-4.40643	2.7268922	0.0018793	2.3439443	down
SEMA4F	-0.56072	7.7348609	-4.40057	2.7889428	0.0019051	2.3232836	down
AGMAT	-0.93821	7.5296173	-4.4002	2.7928871	0.0019051	2.3219859	down
LOC4407311	.0458359	8.8463992	4.3996344	2.7989574	0.0019051	2.3199924	up
KLF12	-0.93284	8.9435962	-4.39947	2.8006946	0.0019051	2.3194227	down
LOC728014	-0.82083	8.5040502	-4.3982	2.8143403	0.0019075	2.3149599	down
TFF3	-1.35065	7.4645051	-4.3955	2.8436764	0.0019177	2.3054387	down
FLJ38969	-0.52041	7.4676527	-4.3937	2.8633825	0.0019269	2.2990982	down
AXIN2	-1.16309	8.7161759	-4.39231	2.8785895	0.0019330	2.2942351	down
LOC644590	-0.52597	8.8532740	-4.39159	2.8866029	0.0019342	2.2916829	down
ZNF30	-0.7106	7.5569523	-4.3906	2.8975485	0.0019374	2.2882082	down
SPRY1	-0.76669	7.0814238	-4.38971	2.9074040	0.0019399	2.2850909	down
PBXIP1	-0.62214	7.6326772	-4.38712	2.9364339	0.0019551	2.2759698	down
MT1G	1.1012300	7.5738155	4.3821965	2.9922730	0.0019797	2.2586772	up
LOC648771	-0.6142	13.021184	-4.38146	3.0006963	0.0019811	2.2560968	down
IMPDH2	-0.73564	10.599895	-4.37904	3.0286402	0.0019843	2.2475882	down
FAM84B	-0.91743	7.6193873	-4.37886	3.0306666	0.0019843	2.2469742	down
HNRPDL	-0.59135	9.8147913	-4.37885	3.0308636	0.0019843	2.2469145	down
)C1001329	-0.64811	7.1436020	-4.37322	3.0966990	0.0020095	2.2271903	down
ZNF211	-0.51103	8.6324228	-4.37285	3.1011291	0.0020095	2.2258782	down
ZNF671	-0.5066	9.1408902	-4.37285	3.1011876	0.0020095	2.2258609	down
NPAL3	-0.54373	9.6441879	-4.3718	3.1135954	0.0020095	2.2221960	down
GBP1	1.0955554	11.74903	4.3712317	3.1203596	0.0020095	2.2202043	up
DEM1	0.6160679	10.290200	4.3712219	3.1204771	0.0020095	2.2201697	up
DPH5	-0.63578	9.0049560	-4.36845	3.1536357	0.0020267	2.2104687	down
TRAPPC6A	-0.60589	9.6002059	-4.36674	3.1743815	0.0020315	2.2044511	down
KIAA0748	-1.03003	9.8976487	-4.36641	3.1784041	0.0020315	2.2032889	down
EEF1G	-0.62974	13.708421	-4.36578	3.1860446	0.0020315	2.2010854	down
FCGR1C	1.4682240	10.198938	4.3633798	3.2153177	0.0020455	2.1926921	up
IMP3	-0.56053	12.000545	-4.36087	3.2462546	0.0020568	2.1839047	down
ADCK2	-0.61438	9.3536345	-4.35991	3.2581395	0.0020602	2.1805512	down
RPS2	-0.61518	14.121136	-4.35421	3.3297939	0.0020962	2.1605896	down
LOC6487100	.7587801	7.1115463	4.3537888	3.3350924	0.0020962	2.1591307	up
ANKRD22	1.8156971	9.8201085	4.352931	3.3460101	0.0020989	2.1561319	up
FBN2	0.6490419	8.1395008	4.3438337	3.4639632	0.0021685	2.1243462	up
CTGF	-0.51947	6.7370598	-4.34008	3.5137737	0.0021910	2.1112484	down

FAM159A	-0.93407	8.3241903	-4.33884	3.5304575	0.0021970	2.1069031	down
EPHA4	-0.80219	7.583071	-4.33772	3.5454356	0.0021982	2.1030195	down
NXT1	-0.54873	10.219704	-4.33765	3.5464142	0.0021982	2.1027663	down
GEMIN4	-0.60285	9.8205645	-4.32456	3.7272143	0.0022967	2.0571580	down
LOC151162	-0.58495	11.226716	-4.32404	3.7345709	0.0022967	2.0553495	down
OCIAD2	-0.71915	10.567523	-4.32342	3.7434092	0.0022977	2.0531816	down
ARG1	1.9392728	8.2458258	4.3222577	3.7599575	0.0023033	2.0491364	up
USE1	-0.60179	7.8461625	-4.31832	3.8165166	0.0023334	2.0354441	down
C16orf57	0.6366368	10.497909	4.3166698	3.8405038	0.0023436	2.0296985	up
C12orf57	-0.84841	11.811679	-4.31116	3.9215175	0.0023884	2.0105565	down
BIN1	-0.78978	11.336217	-4.30899	3.9539471	0.0023974	2.0030051	down
CEACAM8	2.0189019	7.9149993	4.308414	3.9625607	0.0023974	2.0010098	up
FXYD2	-0.66374	5.1196531	-4.30766	3.9739645	0.0023974	1.9983749	down
ZXDB	-0.59099	7.0173094	-4.30688	3.9856975	0.0023974	1.9956718	down
ETS1	-0.80333	13.133323	-4.30569	4.0036377	0.0023974	1.9915541	down
LOC285900	-0.61651	12.817422	-4.30553	4.0060026	0.0023974	1.9910127	down
LOC649095	-0.65676	7.2821423	-4.30443	4.0228316	0.0023989	1.9871692	down
DUSP14	-0.55277	8.3449704	-4.30192	4.0612378	0.0024172	1.9784578	down
CYBASC3	-0.71735	10.706392	-4.30109	4.0739209	0.0024196	1.9755992	down
ZNF134	-0.62443	7.8967392	-4.29798	4.1222033	0.0024297	1.9647979	down
ZIK1	-0.53984	7.2439842	-4.29538	4.1628726	0.0024407	1.9557980	down
ZNF248	-0.57971	7.8199408	-4.29279	4.2038929	0.0024564	1.9468094	down
FCGR1B	1.5393293	11.604121	4.2927012	4.2052663	0.0024564	1.9465099	up
HP	1.8973625	8.2211273	4.2893700	4.2585313	0.0024757	1.9349725	up
CD248	-0.96026	7.9129795	-4.28904	4.2638080	0.0024757	1.9338375	down
SLC16A6	0.5603822	8.0255382	4.2881885	4.2775793	0.0024757	1.9308818	up
GTPBP6	-0.54133	11.501330	-4.28677	4.3004910	0.0024844	1.9259855	down
VCAN	0.8686758	12.179153	4.2850875	4.3279627	0.0024956	1.9201491	up
ATXN7L2	-0.54588	7.4158183	-4.28391	4.3472115	0.0025021	1.9160818	down
RAB11FIP3	-0.50585	9.7379432	-4.28334	4.3565352	0.0025029	1.9141182	down
TAF6L	-0.56147	8.6300412	-4.2813	4.3902094	0.0025177	1.9070612	down
C9orf109	0.5873303	6.6364631	4.2801189	4.4098846	0.0025194	1.9029631	up
YIPF4	0.5366957	10.109761	4.2796713	4.4173373	0.0025194	1.9014156	up
LOC642161	-0.87363	8.6117581	-4.27898	4.4288274	0.0025214	1.8990348	down
RFXDC2	-0.53199	7.0897202	-4.27591	4.4804630	0.0025416	1.8884120	down
MGC33556	-0.60852	9.6251320	-4.26857	4.6060411	0.0025954	1.8630819	down
BUB3	-0.50161	12.471702	-4.2683	4.6107381	0.0025954	1.8621480	down
CAMK1	-0.73243	8.3257508	-4.26749	4.6249316	0.0025954	1.8593317	down
PAQR7	-0.59501	7.2738846	-4.25631	4.8235403	0.0026877	1.8208090	down
)C1001709	1.3281292	9.7860490	4.2551262	4.8451334	0.0026950	1.8167171	up
C17orf45	-0.55756	12.790734	-4.25445	4.8575586	0.0026971	1.8143708	down
ELOVL4	-0.56028	6.7033792	-4.25371	4.8710425	0.0026998	1.8118315	down
LOC399988	-0.60474	12.811219	-4.25049	4.9303046	0.0027171	1.8007540	down
C5orf32	1.3137873	10.342922	4.2499217	4.9408353	0.0027171	1.7987996	up
EIF3L	-0.69581	12.776574	-4.24943	4.9499490	0.0027171	1.7971115	down
MYC	-0.7099	11.149856	-4.24588	5.0163722	0.0027465	1.7849018	down
GYG1	1.0338815	12.133447	4.2452908	5.0275176	0.0027478	1.7828691	up
CD27	-1.12686	10.535992	-4.24432	5.0459052	0.0027530	1.7795252	down
)C1001294	-0.51211	13.950622	-4.24367	5.0581131	0.0027549	1.7773120	down
CCR4	-0.68135	6.7677328	-4.24256	5.0793263	0.0027617	1.7734788	down
CMTM8	-1.06622	9.0190966	-4.24107	5.1078199	0.0027724	1.7683554	down

CRISPLD2	1.1623408	10.736085	4.2405633	5.1175179	0.0027729	1.7666181	up
ABHD14B	-0.56112	8.2903208	-4.2383	5.1612387	0.0027869	1.7588269	down
STRBP	-0.92583	7.8125924	-4.23547	5.2162734	0.0028118	1.7491134	down
ATP8B2	-0.76032	10.187910	-4.23382	5.2485674	0.0028244	1.7434613	down
CHCHD6	-0.63187	6.2239709	-4.23284	5.2678691	0.0028299	1.7400998	down
ARSK	-0.51023	7.1936499	-4.23233	5.2780097	0.0028305	1.7383387	down
FLJ23834	-0.96361	5.8937354	-4.22849	5.3543874	0.0028496	1.7251826	down
DENND2D	-0.65842	11.37392	-4.22826	5.3589777	0.0028496	1.7243980	down
LOC728139	-0.65904	12.692675	-4.21891	5.5498620	0.0029322	1.6923523	down
FAM71E1	-0.51285	5.7176968	-4.20891	5.7612726	0.0030264	1.6581283	down
CYFIP2	-0.53421	12.758212	-4.20563	5.8323427	0.0030507	1.6469060	down
LOC728453	-0.7116	11.658919	-4.2051	5.8437954	0.0030507	1.6451104	down
LOC652616	0.8032958	8.6583185	4.2024069	5.9028873	0.0030713	1.6359017	up
Septin 4	1.6874821	8.7318889	4.1949896	6.0684636	0.0031213	1.6105839	up
HNRPA1P4	-0.80881	10.683995	-4.1915	6.1478939	0.0031518	1.5986837	down
LOC91561	-0.93629	9.3858073	-4.18593	6.2768360	0.0032075	1.5796909	down
DEAF1	-0.51453	7.6579656	-4.18212	6.3663433	0.0032374	1.5667358	down
C6orf64	-0.50067	7.8652628	-4.17975	6.4227553	0.0032556	1.5586645	down
C19orf2	-0.54814	11.433220	-4.17852	6.4522217	0.0032603	1.5544768	down
EEF2	-0.53584	13.866266	-4.17557	6.5234200	0.0032801	1.5444371	down
RFTN1	-0.6198	11.188790	-4.17446	6.5503734	0.0032861	1.5406651	down
LHFPL2	0.9454726	9.1452939	4.1742163	6.5562929	0.0032861	1.5398388	up
FTH1	0.7363999	10.401691	4.1732185	6.5806488	0.0032931	1.5364468	up
LOC387867	-0.6622	13.398465	-4.17132	6.6273324	0.0033059	1.5299803	down
LOC642357	-0.55822	14.275454	-4.1708	6.6401623	0.0033071	1.5282111	down
WWP1	-0.50318	9.9275816	-4.16962	6.6692751	0.0033108	1.524209	down
LOC440311	-0.70669	8.7567870	-4.16444	6.7987986	0.0033489	1.5066158	down
RPS3	-0.51665	14.405982	-4.16286	6.8386840	0.0033616	1.5012658	down
TXNDC12	-0.71988	11.272086	-4.16215	6.8566165	0.0033616	1.4988707	down
STAT1	0.7233352	11.796589	4.1608356	6.8902449	0.0033728	1.4943961	up
ZNF589	-0.71287	7.9893704	-4.15417	7.0626366	0.0034305	1.4717967	down
LOC646483	-0.59318	12.312845	-4.15144	7.1343876	0.0034483	1.4625535	down
OXNAD1	-0.53903	6.1695954	-4.15059	7.1570225	0.0034483	1.4596570	down
S100A8	0.9041762	14.844018	4.1497940	7.1780683	0.0034483	1.4569721	up
AEBP1	-0.68345	7.0199518	-4.14891	7.2016553	0.0034483	1.4539724	down
MMP9	2.1194096	10.783605	4.1480188	7.2254070	0.0034483	1.4509617	up
GIMAP7	-0.59697	12.815453	-4.14786	7.2297776	0.0034483	1.4504088	down
MAL	-0.95733	9.8650747	-4.14565	7.2889874	0.0034704	1.4429511	down
LOC643531	-0.52987	14.039719	-4.14527	7.2992875	0.0034704	1.4416600	down
PDE3B	-0.62449	8.8848400	-4.14432	7.3249796	0.0034739	1.4384474	down
BATF2	1.3420027	8.0567481	4.1407909	7.4212689	0.0034966	1.4265074	up
BRI3P1	0.8898915	10.601969	4.1401922	7.4377191	0.0034991	1.4244831	up
PHB2	-0.5363	10.649622	-4.13243	7.6542168	0.0035849	1.3982536	down
GPR18	-0.84162	10.107056	-4.12677	7.8159370	0.0036450	1.3791425	down
DPH3	0.5197417	8.9699522	4.1229517	7.9267346	0.0036782	1.3662772	up
SWAP70	-0.55612	9.9307175	-4.12225	7.9471618	0.0036782	1.3639251	down
CXCR7	-0.9136	7.3704553	-4.12053	7.9979031	0.0036928	1.3581085	down
RPL3	-0.6431	13.684475	-4.12038	8.0021474	0.0036928	1.3576236	down
ACTR1B	-0.5319	9.2788731	-4.11987	8.0172869	0.0036944	1.355896	down
DGCR6	-0.50004	9.9576881	-4.11642	8.1200195	0.0037308	1.3442606	down
METTL7B	1.2831486	7.0723677	4.1116229	8.2647187	0.0037752	1.3281204	up

KIAA1324L	-0.77873	6.1941132	-4.10783	8.3809730	0.0038061	1.3153575	down
KIAA1430	-0.63887	7.0154811	-4.10687	8.4107080	0.0038141	1.3121216	down
ST8SIA1	-0.70399	6.7656456	-4.10594	8.4393166	0.0038144	1.3090192	down
ZNF529	-0.63166	8.9849478	-4.10556	8.4513424	0.0038144	1.3077182	down
ANKRD46	-0.73196	8.6837927	-4.10489	8.4719573	0.0038144	1.3054923	down
LOC728537	-0.54024	7.075795	-4.09898	8.6581479	0.0038508	1.2856320	down
LOC441506	-0.72059	12.154510	-4.09681	8.7274331	0.0038685	1.2783510	down
CA5B	-0.56714	8.7579095	-4.09626	8.7452776	0.0038709	1.2764851	down
CORO1C	0.6883129	9.2006338	4.0948711	8.7899485	0.0038852	1.2718311	up
BAG3	-0.71629	8.5235069	-4.09394	8.8201463	0.0038931	1.2686983	down
FBXO31	-0.60618	9.4495880	-4.09235	8.8717502	0.0039104	1.2633698	down
AIM2	1.2326390	11.027628	4.0901033	8.9451995	0.0039354	1.2558390	up
GATA3	-0.53861	7.1713000	-4.08631	9.0706990	0.0039735	1.2431142	down
ZNF77	-0.53641	6.8766245	-4.08624	9.0729523	0.0039735	1.2428874	down
BEX2	-0.8046	8.2611529	-4.08573	9.0897886	0.0039735	1.2411942	down
CDC42	0.5370821	9.9915364	4.0857025	9.0908307	0.0039735	1.2410895	up
PAFAH1B3	-0.75345	9.3427629	-4.08388	9.1517695	0.0039835	1.2349880	down
SERPING1	1.4915020	9.4239270	4.0827409	9.1901129	0.0039843	1.2311697	up
RNU1-3	0.8136131	10.149184	4.0824506	9.1998998	0.0039843	1.2301977	up
MEFV	0.7495187	8.7192607	4.0810181	9.2483427	0.0039843	1.2254017	up
TRIB2	-0.81438	9.5251104	-4.08053	9.2649155	0.0039843	1.2237667	down
LOC729816	0.6748080	9.3072339	4.0801852	9.2766237	0.0039843	1.2226134	up
ZCCHC3	-0.60493	7.9073182	-4.07789	9.3548209	0.0040053	1.2149482	down
ELL3	-0.59902	7.0518137	-4.07676	9.3937665	0.0040165	1.2111545	down
LOC641814	-0.5207	13.845187	-4.07529	9.4446032	0.0040278	1.2062263	down
C1QA	0.7171456	7.5928203	4.0752551	9.4457188	0.0040278	1.2061185	up
EIF3F	-0.60735	12.766631	-4.07407	9.4866382	0.0040358	1.2021714	down
EPB41L3	0.7277707	9.2775908	4.0735823	9.5037614	0.0040358	1.2005248	up
MSRB2	0.7065987	11.244797	4.0726607	9.5358861	0.0040358	1.1974437	up
ADAM9	0.5284925	7.3547112	4.0717417	9.5680229	0.0040358	1.1943718	up
ANKRD33	0.7543230	9.0227040	4.0691243	9.6601225	0.0040358	1.1856254	up
DUSP3	0.7169584	12.076425	4.0667926	9.7428841	0.0040608	1.1778370	up
LOC401431	-0.51013	6.4369930	-4.0635	9.8610368	0.0040948	1.1668325	down
NR2C2AP	-0.50245	8.3036772	-4.06341	9.8641682	0.0040948	1.1665427	down
PGAP3	-0.52674	8.7936038	-4.06239	9.9010705	0.0040948	1.1631339	down
EDG1	-0.613	10.287262	-4.0618	9.9222180	0.0040948	1.1611862	down
FCGR1A	1.5380197	11.151781	4.0609980	9.9515116	0.0040948	1.1584952	up
LCN2	1.5808083	10.780791	4.0587199	0.0001003	0.0041135	1.1508965	up
KLHL34	-0.64727	6.6390190	-4.05859	0.0001003	0.0041135	1.1504753	down
LOC644132	0.5041315	8.8804100	4.0561805	0.0001012	0.0041398	1.1424293	up
LOC286208	-0.56773	9.4091259	-4.05514	0.0001016	0.0041440	1.1389737	down
MARCKS	0.6248340	12.662257	4.0501898	0.0001035	0.0041978	1.1224695	up
LOC649049	-0.69839	13.097613	-4.04511	0.0001054	0.0042403	1.1055532	down
GAPT	-0.65946	11.176801	-4.04505	0.0001054	0.0042403	1.1053742	down
CTSD	0.6877292	10.625780	4.0447684	0.0001055	0.0042403	1.1044240	up
RPS5	-0.56304	14.450529	-4.04077	0.0001071	0.0042864	1.0911248	down
BRSK1	0.8215467	9.9188817	4.0401565	0.0001073	0.0042864	1.0890864	up
NET1	-0.50993	8.8969918	-4.03979	0.0001075	0.0042864	1.0878695	down
LAP3	0.8324131	11.997885	4.0395520	0.0001076	0.0042864	1.0870770	up
TMEM194A	-0.59168	8.6041981	-4.03255	0.0001103	0.0043725	1.0638104	down
ZNF256	-0.75059	7.9781856	-4.03225	0.0001105	0.0043725	1.0628115	down

FAM113B	-0.70382	11.560169	-4.01967	0.0001156	0.0045389	1.0210880	down
ZNF831	-0.86508	7.3525809	-4.01801	0.0001163	0.0045492	1.0156088	down
IRAK3	1.0359905	9.8320361	4.0161653	0.0001171	0.0045612	1.0094964	up
TMEM51	0.6691425	7.6476439	4.0100114	0.0001198	0.0046314	0.9891348	up
CTSG	1.6156626	7.9999448	4.0091440	0.0001201	0.0046403	0.9862664	up
RPL36	-0.56809	13.457916	-4.00735	0.0001209	0.0046449	0.9803192	down
TYMP	0.7770848	9.6652040	4.0069819	0.0001211	0.0046449	0.9791186	up
B4GALT5	0.8305433	11.825164	4.0069267	0.0001211	0.0046449	0.9789361	up
LRFN3	-0.63885	8.5763782	-4.00684	0.0001211	0.0046449	0.9786611	down
LOC650815	-0.6617	7.9026036	-4.00616	0.0001214	0.0046508	0.9763950	down
TRIM32	-0.53583	7.7303781	-4.00311	0.0001228	0.0046911	0.9663140	down
C14orf167	-0.61552	8.3063845	-4.00271	0.0001230	0.0046922	0.9649917	down
HAPLN3	-0.76784	7.4493904	-3.99756	0.0001253	0.0047688	0.9480160	down
LOC339290	-0.72227	7.9540278	-3.9959	0.0001260	0.0047918	0.9425299	down
MYST4	-0.63191	7.3978211	-3.99327	0.0001273	0.0048260	0.9338386	down
KCTD12	0.6726954	11.401641	3.9902265	0.0001287	0.0048735	0.9238191	up
FLJ22662	0.9411317	12.836499	3.9849912	0.0001311	0.0049487	0.9065736	up
LOC649821	-0.60401	12.461077	-3.98189	0.0001326	0.0049917	0.8963611	down
S100A6	0.5128027	13.545872	3.9812940	0.0001329	0.0049917	0.8944046	up
PSG3	0.9816220	7.9748906	3.9799969	0.0001335	0.0050041	0.8901374	up
CAMP	1.3599294	10.676825	3.9794279	0.0001338	0.0050041	0.8882657	up
DRAM1	0.7792431	9.2527160	3.9782174	0.0001344	0.0050110	0.8842843	up
SATB1	-0.64703	8.3635004	-3.97591	0.0001355	0.0050309	0.8766888	down
DBNDD1	-0.55293	6.1005788	-3.97581	0.0001355	0.0050309	0.8763659	down
LOC388275	-0.778	9.2723478	-3.974	0.0001364	0.0050519	0.8704255	down
MAN1A1	0.5543966	8.2422946	3.9733296	0.0001368	0.0050574	0.8682173	up
PLSCR1	1.0650507	9.5518542	3.9728703	0.0001370	0.0050574	0.8667082	up
ATP6V0E2	-0.84942	10.611249	-3.97223	0.0001373	0.0050574	0.8646081	down
NEXN	0.6378438	8.8413108	3.9714098	0.0001377	0.0050583	0.8619107	up
LOC390345	-0.7275	13.684265	-3.97104	0.0001379	0.0050583	0.8607118	down
C20orf127	0.6653572	7.9121275	3.9696539	0.0001386	0.0050755	0.8561439	up
STOM	0.7837008	10.324091	3.9686593	0.0001391	0.0050797	0.8528786	up
PHF15	-0.50326	9.9003688	-3.96153	0.0001427	0.0051803	0.8294907	down
C7orf23	-0.50299	10.795674	-3.96102	0.0001430	0.0051838	0.8278206	down
CD6	-0.95605	11.257891	-3.95501	0.0001461	0.0052546	0.8081385	down
CNR2	-0.55507	5.9011050	-3.95452	0.0001463	0.0052546	0.8065187	down
ZFP82	-0.77078	7.7298633	-3.95438	0.0001464	0.0052546	0.8060600	down
POLRMT	-0.59032	7.9693730	-3.95034	0.0001486	0.0053072	0.7928307	down
MXD4	-0.67214	11.289891	-3.94689	0.0001504	0.0053580	0.7815492	down
RHAG	1.4517594	7.1718330	3.9431403	0.0001525	0.0054032	0.7692903	up
SFT2D3	-0.54615	6.946605	-3.94165	0.0001533	0.0054139	0.7644112	down
HVCN1	-0.63527	9.8236376	-3.93337	0.0001579	0.0055397	0.7373927	down
MARS2	-0.51758	8.1465585	-3.9328	0.0001582	0.0055447	0.7355420	down
NCR3	-0.81088	8.1808380	-3.93088	0.0001593	0.0055768	0.7292725	down
ZNF439	-0.6243	7.9389112	-3.92971	0.0001600	0.0055879	0.7254469	down
SCML1	-0.90678	8.1371932	-3.92932	0.0001602	0.0055895	0.7241727	down
ASAP1IT1	0.7651536	8.0666237	3.9286574	0.0001606	0.0055965	0.7220211	up
NCRNA0021	-0.53695	10.464274	-3.92473	0.0001629	0.0056631	0.7092311	down
ELANE	1.8923544	9.2472759	3.921331	0.0001648	0.0057072	0.6981549	up
HIST3H2BB	0.6536255	6.1437858	3.9185356	0.0001665	0.0057582	0.6890591	up
CEBPE	-0.8837	7.4803792	-3.91723	0.0001673	0.0057742	0.6848127	down

DNAJC30	-0.61625	9.7435501	-3.91715	0.0001673	0.0057742	0.6845391	down
LOC648294	-0.62204	13.532833	-3.91498	0.0001686	0.0058062	0.6775035	down
)C1001323	-0.67054	6.9435119	-3.91375	0.0001694	0.0058190	0.6735068	down
COL17A1	1.2381627	7.7542033	3.9123133	0.0001702	0.0058312	0.6688260	up
TIAM1	-0.70279	7.8634739	-3.91165	0.0001707	0.0058312	0.6666557	down
ZNF548	-0.61712	8.6848678	-3.91038	0.0001714	0.0058434	0.6625518	down
C9orf127	-0.67116	7.3493267	-3.91014	0.0001716	0.0058434	0.6617802	down
ZNF614	-0.548	8.2808505	-3.90971	0.0001718	0.0058445	0.6603543	down
MTE	0.7397034	8.5556761	3.9094871	0.0001720	0.0058445	0.6596436	up
SCML4	-0.66624	6.7524104	-3.90401	0.0001754	0.0059407	0.6418478	down
C19orf59	1.7946113	11.632413	3.9012325	0.0001771	0.0059932	0.6328515	up
LOC730246	-0.52385	12.901781	-3.8994	0.0001783	0.0060195	0.6269176	down
LOC388707	-0.67301	11.750487	-3.89475	0.0001813	0.0060983	0.6118257	down
MTUS1	-0.54007	6.2954108	-3.88841	0.0001854	0.0061737	0.5913014	down
GNPDA2	-0.56717	8.1331738	-3.88485	0.0001878	0.0062324	0.5798046	down
TASP1	-0.5745	6.7754377	-3.88288	0.0001891	0.0062399	0.5734408	down
C10orf2	-0.53872	7.9013968	-3.88267	0.0001892	0.0062399	0.5727405	down
LOC400455	-0.50631	10.419411	-3.8823	0.0001895	0.0062399	0.5715499	down
FBXO6	0.8412896	10.558749	3.8821956	0.0001895	0.0062399	0.5712169	up
ZNF337	-0.55656	9.0489283	-3.87998	0.0001910	0.0062623	0.5640530	down
LOC728428	-0.54118	13.588802	-3.87859	0.0001920	0.0062798	0.5595619	down
PLCG1	-0.71883	8.8758010	-3.87695	0.0001931	0.0062969	0.5542659	down
LOC728744	1.3864030	8.8178479	3.8764286	0.0001935	0.0063020	0.5525885	up
RNU1G2	0.7752350	10.028706	3.8722419	0.0001964	0.0063897	0.5390767	up
LOC642393	0.5832228	8.2369940	3.8703912	0.0001977	0.0064251	0.5331073	up
LOC648984	1.2794282	9.9717331	3.8690202	0.0001986	0.0064431	0.5286866	up
LANCL1	-0.52852	9.7672768	-3.8664	0.0002005	0.0064898	0.5202516	down
SAP30	0.8231205	7.8907122	3.8646072	0.0002017	0.0065245	0.5144645	up
LOC729423	-0.80614	8.4402083	-3.85975	0.0002052	0.0066105	0.4988338	down
RNU1A3	0.5701938	11.264807	3.8583629	0.0002063	0.0066205	0.4943608	up
LOC441775	-0.71629	13.508132	-3.85756	0.0002068	0.0066205	0.4917708	down
CREG1	0.9663332	11.463133	3.8570265	0.0002072	0.0066205	0.4900611	up
SKAP1	-0.78913	11.058698	-3.85699	0.0002073	0.0066205	0.4899339	down
LOC283953	0.6570264	8.8895254	3.8569671	0.0002073	0.0066205	0.4898700	up
LOC646200	-0.53349	13.233515	-3.85673	0.0002075	0.0066205	0.4891210	down
TRAPPC5	0.5267503	11.713768	3.8523491	0.0002107	0.0066968	0.4750209	up
KRT73	-0.64233	6.8545178	-3.85168	0.0002112	0.0067006	0.4728556	down
DEFA3	2.0481993	12.276179	3.8513506	0.0002114	0.0067006	0.4718119	up
CD79B	-0.99506	10.144505	-3.84991	0.0002125	0.0067006	0.4671773	down
TXN	0.7274387	11.900875	3.8452848	0.0002160	0.0067703	0.4523308	up
ENTPD1	0.5043450	9.2707136	3.8428239	0.0002179	0.0068092	0.4444336	up
ACCS	-0.58033	8.4269557	-3.84107	0.0002193	0.0068378	0.4387955	down
CLYBL	-0.84965	8.3032806	-3.84062	0.0002196	0.0068418	0.4373661	down
GNG7	-0.80921	9.1466248	-3.83825	0.0002215	0.0068788	0.4297637	down
UBE2G2	-0.52272	9.6348273	-3.82967	0.0002283	0.0070255	0.4022900	down
MTX3	-0.61189	8.8181090	-3.82933	0.0002285	0.0070255	0.4011971	down
C21orf57	-0.54851	8.7374035	-3.8292	0.0002286	0.0070255	0.4007716	down
C8orf55	-0.59294	9.9257855	-3.8268	0.0002306	0.0070499	0.3931078	down
IFFO2	-0.528	7.4561432	-3.82655	0.0002308	0.0070499	0.3923152	down
DPPA4	-0.62917	6.3963431	-3.82357	0.0002332	0.0071041	0.3827670	down
CLIP3	-0.83124	6.3515101	-3.82355	0.0002332	0.0071041	0.3827148	down

IL15	0.6913505	8.8773380	3.8223512	0.0002342	0.0071088	0.3788764	up
NLRC3	-0.54678	8.5183862	-3.82229	0.0002343	0.0071088	0.3786875	down
CD58	0.6545918	9.7308789	3.8219971	0.0002345	0.0071088	0.3777449	up
TMEM194	-0.50912	7.9556531	-3.82169	0.0002348	0.0071096	0.3767547	down
DOCK9	-0.60521	6.4146475	-3.81896	0.0002370	0.0071583	0.3680454	down
THEM4	-0.56914	5.9309029	-3.8188	0.0002372	0.0071583	0.3675405	down
LOC645904	-0.50743	7.3977220	-3.81867	0.0002373	0.0071583	0.3671297	down
GRN	0.7620238	11.143458	3.8176783	0.0002381	0.0071760	0.3639486	up
SLC25A6	-0.55846	12.349767	-3.81663	0.0002390	0.0071955	0.3606125	down
COL9A2	-0.77529	6.7553581	-3.81551	0.0002399	0.0072171	0.3570234	down
B3GNT6	-0.58722	6.5421516	-3.81307	0.0002420	0.0072721	0.3492529	down
LTB4R	0.8031547	8.6396112	3.8124026	0.0002426	0.0072823	0.3471111	up
PDLIM7	0.8919662	9.2889611	3.8119173	0.0002430	0.0072878	0.3455630	up
EDC3	-0.52653	7.7047315	-3.80997	0.0002446	0.0073169	0.3393479	down
FAM20A	0.8118984	6.5084251	3.8082074	0.0002462	0.0073348	0.3337338	up
ADCY3	1.0294842	10.656872	3.8032902	0.0002505	0.0074142	0.3180681	up
RPL14L	-0.59411	12.498801	-3.80132	0.0002522	0.0074493	0.3118042	down
TLR5	1.0050113	10.682620	3.7997246	0.0002536	0.0074712	0.3067176	up
ZNF84	-0.53843	7.3014182	-3.79729	0.0002558	0.0075086	0.2989687	down
PTPRCAP	-0.7841	12.085551	-3.79602	0.0002569	0.0075263	0.2949402	down
DEFA4	1.8069699	9.0431492	3.7945597	0.0002582	0.0075579	0.2902901	up
LOC729102	-0.59553	11.846362	-3.79402	0.0002587	0.0075651	0.2885830	down
SVOPL	-0.62821	5.4313664	-3.79289	0.0002598	0.0075881	0.2849789	down
CD1C	-0.56923	6.7625119	-3.78432	0.0002677	0.0077828	0.2577817	down
RNY4	0.7252882	7.6856452	3.7834438	0.0002685	0.0077924	0.2549891	up
LOC158345	-0.62882	12.926406	-3.78276	0.0002691	0.0077958	0.2528348	down
C11orf75	0.5803213	10.458031	3.7818707	0.0002700	0.0077958	0.2499995	up
LTC4S	-0.61918	5.5768773	-3.77903	0.0002727	0.0078483	0.2410002	down
MPO	1.3478940	7.8132916	3.7781963	0.0002735	0.0078640	0.2383511	up
PLXDC2	0.6976528	9.7493133	3.7760417	0.0002755	0.0078945	0.2315243	up
LTBR	0.6741560	9.9486546	3.7724680	0.0002790	0.0079646	0.2202074	up
DTX3	-0.64078	6.8641502	-3.76855	0.0002828	0.0080591	0.2078039	down
LDOC1	-0.64128	6.5796415	-3.76536	0.0002860	0.0081271	0.1977290	down
DYNLT1	0.7149851	11.429297	3.7650159	0.0002863	0.0081271	0.1966345	up
CD3E	-0.72933	10.224143	-3.76428	0.0002871	0.0081361	0.1943200	down
)C1001282	-0.6721	7.7273980	-3.76251	0.0002888	0.0081719	0.1887025	down
ECGF1	0.7568072	9.2146880	3.7602430	0.0002911	0.0082218	0.1815543	up
LOC728031	-0.61219	12.078850	-3.75899	0.0002924	0.0082357	0.1775824	down
ZNF285A	-0.56493	7.0073538	-3.75805	0.0002934	0.0082479	0.1746164	down
HIST1H2BC	0.9334292	8.5822258	3.7569164	0.0002945	0.0082668	0.1710518	up
)C1001338	1.0045878	9.6532122	3.7471315	0.0003047	0.0084955	0.1402001	up
LCK	-0.55695	8.7138590	-3.74673	0.0003051	0.0084955	0.1389224	down
RNU1-5	0.6816193	10.610963	3.7443877	0.0003076	0.0085572	0.1315595	up
DEFA1	1.7816406	13.11956	3.7425134	0.0003096	0.0085988	0.1256595	up
P2RX7	0.5068108	9.3785435	3.7424832	0.0003097	0.0085988	0.1255645	up
VNN1	1.4522689	9.0321103	3.7403399	0.0003120	0.0086554	0.1188207	up
PTPDC1	-0.66166	6.2215108	-3.74001	0.0003123	0.0086576	0.1177912	down
GBA	0.6614144	9.3669048	3.7396062	0.0003128	0.0086622	0.1165127	up
RNU4ATAC	0.8343265	8.0904126	3.7343760	0.0003185	0.0087821	0.1000703	up
NLRC4	0.9297755	8.5278047	3.7340108	0.0003189	0.0087855	0.0989229	up
DEFA1B	2.0163582	11.812444	3.7317164	0.0003215	0.0088170	0.0917160	up

LOC647856	-0.61851	13.393117	-3.73102	0.0003222	0.0088305	0.0895379	down
ASAP1	0.5266302	11.611794	3.7299882	0.0003234	0.0088468	0.0862894	up
ANXA2	0.5408313	9.1822249	3.7281979	0.0003254	0.0088864	0.0806698	up
MEGF9	0.7720402	8.7558898	3.7273890	0.0003263	0.0088959	0.0781316	up
ZDHHC23	-0.54957	7.8350746	-3.72598	0.0003279	0.0089316	0.0737155	down
TCN1	1.2295165	9.6580475	3.7232597	0.0003310	0.0089928	0.0651801	up
SCO2	0.7355647	11.248000	3.7226487	0.0003317	0.0089963	0.0632644	up
PHB	-0.51898	9.3464095	-3.71969	0.0003351	0.0090811	0.0539764	down
ANXA3	1.6011984	9.5415028	3.7164131	0.0003390	0.0091450	0.0437288	up
IL2RA	-0.60414	7.2277332	-3.71567	0.0003398	0.0091530	0.0413919	down
PARP9	0.7566311	12.156188	3.7143377	0.0003414	0.0091872	0.0372323	up
HBD	1.2202118	14.543652	3.7132797	0.0003426	0.0091921	0.0339212	up
ZNF296	-0.54976	10.010333	-3.70581	0.0003516	0.0093893	0.0105801	down
RAP1GAP	2.2951231	10.463347	3.7050939	0.0003525	0.0093923	0.0083284	up
IRF8	-0.52584	12.035296	-3.70498	0.0003526	0.0093923	0.0079801	down
LOC653610	0.9467956	8.1624579	3.7005150	0.0003581	0.0095220	-0.00597	up
LOC25845	-0.64467	8.1611345	-3.69968	0.0003591	0.0095335	-0.00859	down
MYOF	1.0608450	8.4390348	3.6985600	0.0003605	0.0095621	-0.01207	up
CLEC5A	1.1426152	7.3848706	3.6973192	0.0003621	0.0095789	-0.01594	up
REG4	-0.6218	5.7980519	-3.69636	0.0003633	0.0096026	-0.01894	down
FAM124B	-0.5569	6.0436666	-3.69218	0.0003685	0.0097011	-0.03198	down
C10orf10	0.5252808	6.4488667	3.6886247	0.0003731	0.0097714	-0.04304	up
CTSF	-0.54167	7.1586680	-3.68862	0.0003731	0.0097714	-0.04306	down
CBS	0.7689530	6.9284373	3.6876987	0.0003742	0.0097861	-0.04592	up
BPI	1.3927838	8.0012972	3.6874482	0.0003746	0.0097864	-0.0467	up
RAB6B	0.6398775	6.6803347	3.6837254	0.0003794	0.0098823	-0.05828	up
MT2A	0.8639118	12.315020	3.6836474	0.0003795	0.0098823	-0.05853	up
LACTB	0.6012349	7.9187613	3.6827399	0.0003807	0.0099049	-0.06135	up
LOC650799	-0.51474	7.4656842	-3.68134	0.0003825	0.0099225	-0.0657	down
LAMP2	0.6055279	10.283755	3.6799771	0.0003843	0.0099500	-0.06994	up
SERPINA10	-0.61163	8.8593949	-3.67924	0.0003853	0.0099507	-0.07223	down
SLC4A7	-0.53003	7.8354448	-3.67874	0.0003860	0.0099596	-0.07379	down
PPP3CC	-0.59073	10.113036	-3.67617	0.0003894	0.0099790	-0.08177	down
LOC401537	-0.66541	12.646103	-3.67604	0.0003895	0.0099790	-0.08217	down
NT5C3	0.6829653	10.301165	3.6760135	0.0003896	0.0099790	-0.08225	up
CXorf45	-0.52626	8.2181179	-3.67579	0.0003899	0.0099790	-0.08295	down
ANKRD9	0.8705003	7.1518689	3.6745580	0.0003915	0.0100131	-0.08677	up
SESN1	-0.60219	8.4121303	-3.67332	0.0003932	0.0100476	-0.09062	down
GPX7	-0.66782	9.6785880	-3.67285	0.0003938	0.0100557	-0.09208	down
IDO1	-1.28104	8.3901609	-3.67105	0.0003963	0.0101014	-0.09766	down
TTC27	-0.54122	9.2690980	-3.67044	0.0003971	0.0101143	-0.09954	down
SLC26A8	1.0429679	6.4032959	3.6688185	0.0003993	0.0101443	-0.10458	up
PSTPIP2	0.8823543	10.198035	3.6648950	0.0004047	0.0102339	-0.11675	up
UST	-0.6185	6.9748707	-3.66416	0.0004057	0.0102433	-0.11903	down
TNFAIP6	1.2106691	11.618626	3.6629965	0.0004074	0.0102652	-0.12263	up
PTPRK	-0.63223	6.6768023	-3.6626	0.0004079	0.0102652	-0.12386	down
IFI27L2	0.6346359	8.9217388	3.6604803	0.0004109	0.0103151	-0.13042	up
LOC729617	-0.56661	13.784779	-3.65913	0.0004128	0.0103404	-0.1346	down
C2orf88	0.6436631	6.3841409	3.6562221	0.0004169	0.0104203	-0.1436	up
SH3GLB1	0.6593844	11.907001	3.6561170	0.0004171	0.0104203	-0.14392	up
C9orf45	-0.98507	8.0147356	-3.6551	0.0004185	0.0104221	-0.14707	down

DYSF	1.1343650	11.243346	3.6546744	0.0004191	0.0104221	-0.14838	up
LRRC8C	-0.54964	8.1146365	-3.65334	0.0004210	0.0104531	-0.1525	down
NAT6	-0.55812	7.5367090	-3.64977	0.0004262	0.0105427	-0.16356	down
SDK2	-0.58201	7.0202076	-3.64947	0.0004267	0.0105427	-0.16446	down
NPL	0.8346105	10.978857	3.6482794	0.0004284	0.0105605	-0.16815	up
OR2A9P	-0.66603	6.8533835	-3.64751	0.0004295	0.0105739	-0.17052	down
FLVCR2	0.7139256	9.9216496	3.6446348	0.0004338	0.0106466	-0.1794	up
LOC127295	-0.58794	12.328694	-3.64432	0.0004342	0.0106466	-0.18036	down
HIST1H2BE	0.8711689	7.5425782	3.6442980	0.0004343	0.0106466	-0.18044	up
LAT	-0.52842	9.5572341	-3.63923	0.0004418	0.0107692	-0.19606	down
CCDC109B	-0.50798	11.956721	-3.63726	0.0004448	0.0108126	-0.20214	down
FCRLA	-1.05203	10.933847	-3.63421	0.0004495	0.0108920	-0.21154	down
SIGLEC9	0.7534872	8.1872793	3.6322078	0.0004525	0.0109411	-0.2177	up
APOL6	0.6685000	8.6137203	3.6299538	0.0004560	0.0109916	-0.22463	up
LOC728519	1.0752952	9.3043797	3.6263494	0.0004617	0.0110732	-0.23572	up
LOC90925	-1.08749	9.4985016	-3.62621	0.0004619	0.0110732	-0.23616	down
FLJ12334	-0.57308	7.2666105	-3.62569	0.0004627	0.0110843	-0.23775	down
FCER2	-1.13634	8.1897023	-3.62376	0.0004657	0.0111402	-0.24367	down
ASB13	-0.55403	6.7533915	-3.62139	0.0004695	0.0112188	-0.25096	down
RPUSD2	-0.57479	9.6992099	-3.62121	0.0004698	0.0112188	-0.25152	down
ETV7	0.6976549	6.8525910	3.6164085	0.0004775	0.0113018	-0.26625	up
LOC644907	-0.60061	12.656887	-3.60994	0.0004881	0.0114836	-0.28609	down
RRAGD	0.7167384	10.600485	3.6093371	0.0004891	0.0114900	-0.28793	up
CLEC4E	0.7905487	8.4593826	3.6093319	0.0004891	0.0114900	-0.28795	up
TNFRSF21	-0.71394	7.1218027	-3.60811	0.0004912	0.0115290	-0.29168	down
RETN	1.5738535	8.5642938	3.6064828	0.0004939	0.0115756	-0.29667	up
C1orf19	-0.50095	9.4700971	-3.60622	0.0004943	0.0115771	-0.29746	down
LOC644353	-0.60574	6.4552772	-3.60568	0.0004952	0.0115899	-0.29913	down
MOBKL2B	-0.8582	6.7532244	-3.60481	0.0004967	0.0116153	-0.30178	down
LOC391045	0.6745319	7.7965911	3.6028283	0.0005000	0.0116850	-0.30786	up
CR2	-0.77997	7.6209670	-3.60144	0.0005024	0.0117141	-0.31211	down
RSAD1	-0.57021	7.5848325	-3.60086	0.0005034	0.0117285	-0.31389	down
LOC730525	-0.60124	7.9601300	-3.59881	0.0005069	0.0117835	-0.32015	down
C10orf125	-0.51658	6.7957746	-3.59825	0.0005079	0.0117835	-0.32186	down
TMEM167A	0.6379406	8.2459026	3.5980997	0.0005081	0.0117835	-0.32232	up
SERPINE2	-0.67909	8.7486442	-3.5961	0.0005116	0.0118399	-0.32843	down
ITGAM	0.7894319	10.789752	3.5898774	0.0005224	0.0120209	-0.34743	up
FTHL8	1.1214510	12.528323	3.5894462	0.0005232	0.0120232	-0.34874	up
BAZ1A	0.5182561	10.423953	3.5893871	0.0005233	0.0120232	-0.34892	up
IGDCC3	0.6270497	4.6408660	3.5890605	0.0005239	0.0120277	-0.34992	up
ENDOG	-0.56578	7.4529963	-3.58215	0.0005363	0.0122458	-0.37098	down
C16orf68	0.5702297	9.6595839	3.5810424	0.0005383	0.0122683	-0.37436	up
CUTA	-0.52665	12.381354	-3.57801	0.0005438	0.0123585	-0.38358	down
ZNF416	-0.53732	7.3552104	-3.57732	0.0005451	0.0123784	-0.38569	down
OC1001335	0.8958637	13.265993	3.5712378	0.0005564	0.0125894	-0.40419	up
LOC653600	2.1849038	9.9789538	3.5700332	0.0005586	0.0126214	-0.40785	up
SHPRH	-0.6015	7.3972067	-3.56525	0.0005677	0.0127723	-0.42239	down
GGA2	-0.53659	9.3630545	-3.56232	0.0005733	0.0128551	-0.43125	down
CKAP4	0.8227617	11.120387	3.5622646	0.0005734	0.0128551	-0.43143	up
AP3M2	-0.59758	8.4933667	-3.56154	0.0005748	0.0128772	-0.43362	down
TMED3	-0.5278	11.410806	-3.56001	0.0005778	0.0129345	-0.43827	down

CEACAM1	1.2318637	8.9418526	3.5586860	0.0005804	0.0129829	-0.44228	up
MTF1	0.5690779	10.832915	3.5563234	0.0005850	0.0130493	-0.44944	up
QPCT	0.9092645	12.026101	3.5554514	0.0005867	0.0130690	-0.45209	up
RAP1A	0.5027241	9.3004091	3.5537796	0.0005900	0.0131333	-0.45715	up
LOC653061	0.6317447	6.3029248	3.5521483	0.0005933	0.0131517	-0.46209	up
CXXC5	-0.63113	11.204367	-3.55176	0.0005940	0.0131517	-0.46326	down
BTAF1	-0.5081	10.618145	-3.55175	0.0005941	0.0131517	-0.46329	down
ICRNA0015	0.6497054	8.0371181	3.5491907	0.0005992	0.0132248	-0.47104	up
INDO	-1.23086	7.5527881	-3.5478	0.0006020	0.0132679	-0.47524	down
SLC41A1	-0.58175	7.4749321	-3.54427	0.0006092	0.0133534	-0.4859	down
RPS23	-1.33908	9.1269639	-3.54304	0.0006117	0.0133876	-0.48963	down
STAB1	0.6098355	7.1733494	3.5396262	0.0006187	0.0134904	-0.49994	up
TSHZ3	0.7989472	8.9032509	3.5367007	0.0006248	0.0135851	-0.50877	up
ABCB4	-0.66825	6.5036227	-3.53498	0.0006284	0.0136479	-0.51395	down
FRMD3	0.6592620	9.2639204	3.5323817	0.0006339	0.0137305	-0.52179	up
FER1L3	1.0617878	9.2000927	3.5313043	0.0006362	0.0137422	-0.52504	up
Septin 1	-0.56553	8.1611046	-3.52469	0.0006504	0.0139294	-0.54495	down
LOC650795	-0.57168	7.3709111	-3.52459	0.0006506	0.0139294	-0.54524	down
TMEM99	-0.50062	8.7727081	-3.5207	0.0006591	0.0140829	-0.55695	down
BEND5	-0.8614	8.5246868	-3.51845	0.0006641	0.0141795	-0.56372	down
CKB	-0.60933	6.6515741	-3.51772	0.0006657	0.0141947	-0.56591	down
TC2N	-0.85826	7.9971242	-3.51366	0.0006748	0.0143114	-0.57812	down
CNN3	-0.5954	6.7053398	-3.50835	0.0006868	0.0145001	-0.59404	down
LOC283340	-0.61244	6.7679958	-3.50313	0.0006989	0.0146827	-0.60969	down
ZNF783	-0.53607	7.8431917	-3.50292	0.0006994	0.0146832	-0.61032	down
CD177	1.6830216	7.2759834	3.5018597	0.0007018	0.0147153	-0.61349	up
NT5E	-0.90136	6.6983498	-3.49929	0.0007079	0.0148020	-0.62117	down
C1orf165	-0.6314	6.7506454	-3.49842	0.0007099	0.0148351	-0.62378	down
LOC388564	-0.57988	9.2576428	-3.4978	0.0007114	0.0148481	-0.62563	down
SIGLEC16	0.6518627	6.6210968	3.4971397	0.0007129	0.0148687	-0.62761	up
AIF1	0.6622425	11.741352	3.4961633	0.0007153	0.0148972	-0.63053	up
DPRXP4	0.6265871	9.1613394	3.4924832	0.0007241	0.0150135	-0.64153	up
FAM160B1	0.6204496	7.5210203	3.4922184	0.0007247	0.0150135	-0.64232	up
SLC7A5	1.0969618	10.464859	3.4921554	0.0007248	0.0150135	-0.64251	up
CYBB	0.5143397	10.997098	3.4920305	0.0007251	0.0150135	-0.64289	up
AGTRAP	0.6259677	10.135248	3.4889328	0.0007326	0.0151015	-0.65214	up
UBFD1	0.5733905	8.3920558	3.4886861	0.0007332	0.0151015	-0.65287	up
HIST2H2BE	0.8597160	10.846292	3.4870645	0.0007372	0.0151384	-0.65772	up
EVL	-0.65181	12.378338	-3.48687	0.0007377	0.0151384	-0.65831	down
LOC647285	-0.59037	11.472807	-3.48664	0.0007382	0.0151384	-0.65899	down
ZNF260	-0.58774	8.0003394	-3.48657	0.0007384	0.0151384	-0.65919	down
MAOA	1.3159761	5.9714554	3.4836307	0.0007456	0.0152469	-0.66796	up
ZNF404	-0.5032	6.1292755	-3.48143	0.0007511	0.0153088	-0.67452	down
LOC728093	1.0771543	7.2940596	3.4788894	0.0007574	0.0154281	-0.68209	up
KIF1B	0.6486351	7.8301573	3.4773403	0.0007613	0.0154974	-0.68671	up
SGK	-0.50633	10.996173	-3.47688	0.0007625	0.0155109	-0.68807	down
LOC653907	0.7845275	8.9288872	3.4746597	0.0007681	0.0155661	-0.69469	up
MOSC1	0.9099988	10.060296	3.4732711	0.0007717	0.0156165	-0.69882	up
LOC729985	-0.53989	7.3930936	-3.47206	0.0007748	0.0156489	-0.70242	down
C1001292	0.5842878	9.0394276	3.4681164	0.0007849	0.0158441	-0.71415	up
SLC6A12	0.7371803	7.5040710	3.4661234	0.0007901	0.0159080	-0.72007	up

STXBP2	0.6455665	11.665808	3.4565447	0.0008155	0.0163034	-0.7485	up
LOC440607	0.6015556	6.3640028	3.4517028	0.0008286	0.0164721	-0.76284	up
CRIP1	-0.65571	13.051009	-3.45061	0.0008316	0.0165093	-0.76608	down
C5orf53	-0.5772	7.8042614	-3.44846	0.0008375	0.0165859	-0.77244	down
TFE3	0.5097310	8.6017893	3.4468850	0.0008419	0.0166502	-0.7771	up
CCNDBP1	0.7462796	8.8043142	3.4466441	0.0008425	0.0166502	-0.77782	up
HNMT	0.7889972	7.4746351	3.4457574	0.0008450	0.0166712	-0.78044	up
SIRPG	-0.65358	6.8489129	-3.44499	0.0008471	0.0167028	-0.7827	down
TXK	-0.68761	9.0673813	-3.44371	0.0008507	0.0167420	-0.7865	down
TSPYL5	-0.55347	6.1872184	-3.44166	0.0008565	0.0168197	-0.79255	down
UBE2H	0.8408525	8.3314266	3.4390871	0.0008637	0.0169346	-0.80015	up
LOC389293	1.1636581	8.5491511	3.4360151	0.0008725	0.0170958	-0.80922	up
C14orf124	-0.53937	6.8747087	-3.43508	0.0008752	0.0171271	-0.81198	down
SIPA1L2	1.0727203	9.0542976	3.4339757	0.0008784	0.0171678	-0.81523	up
TDRD9	1.0848963	7.2601810	3.4304818	0.0008885	0.0173202	-0.82554	up
GPR141	0.7483709	7.0458607	3.4303193	0.0008890	0.0173202	-0.82601	up
RAB32	0.5949953	11.508970	3.4273239	0.0008978	0.0174543	-0.83484	up
PRKCH	-0.6653	11.900754	-3.42339	0.0009094	0.0176213	-0.84643	down
KLRB1	-0.83766	11.829807	-3.42087	0.0009169	0.0177178	-0.85384	down
TIMM10	0.8724559	10.538981	3.4170253	0.0009286	0.0178534	-0.86514	up
ZNF286A	-0.52947	6.3880191	-3.41666	0.0009297	0.0178534	-0.86622	down
FADS1	0.6787490	7.4225311	3.4162323	0.0009310	0.0178534	-0.86747	up
DMXL2	0.5577438	8.0644977	3.4160182	0.0009316	0.0178534	-0.86809	up
ILVBL	-0.52284	8.0148550	-3.4155	0.0009332	0.0178731	-0.86963	down
SMYD2	-0.5412	7.0467106	-3.41445	0.0009364	0.0178921	-0.87269	down
LOC730051	-0.60568	7.7153480	-3.41443	0.0009365	0.0178921	-0.87277	down
DISC1	0.7029249	7.3812624	3.4140736	0.0009376	0.0179019	-0.87381	up
MTMR3	0.7353076	7.4963932	3.4132618	0.0009401	0.0179386	-0.87619	up
MYBPC3	0.7263909	8.2377296	3.4122488	0.0009432	0.0179654	-0.87916	up
SIDT1	-0.56456	8.5582601	-3.41155	0.0009453	0.0179702	-0.8812	down
PRKCQ	-0.58639	10.536494	-3.41154	0.0009454	0.0179702	-0.88124	down
CCR6	-0.8132	10.034541	-3.4044	0.0009677	0.0182224	-0.90219	down
LOC731985	-0.57829	12.424502	-3.40027	0.0009808	0.0184030	-0.91426	down
NFIL3	0.7413181	10.361237	3.3983678	0.0009869	0.0184692	-0.91984	up
IFI30	0.5993146	12.036523	3.3973157	0.0009903	0.0185085	-0.92292	up
WDR54	-0.56517	10.144173	-3.39172	0.0010085	0.0187446	-0.93929	down
LOC729009	1.1769507	11.225347	3.3914421	0.0010094	0.0187502	-0.94009	up
CD4	-0.58672	8.5945568	-3.38925	0.0010167	0.0188511	-0.94649	down
MTHFD2	0.6046687	9.1074309	3.3849897	0.0010309	0.0190671	-0.95892	up
C2	0.7636067	7.1605186	3.3822579	0.0010401	0.0191583	-0.96689	up
OSCAR	0.7471662	10.594712	3.3793117	0.0010501	0.0192848	-0.97548	up
KIAA1958	0.5118977	7.4479979	3.3788274	0.0010517	0.0192848	-0.97689	up
NTN3	0.8353812	7.1860502	3.3770563	0.0010578	0.0193509	-0.98204	up
UBE2L6	0.5425952	10.913389	3.3754629	0.0010633	0.0194061	-0.98668	up
C17orf60	0.6933804	9.4198037	3.3736652	0.0010695	0.0194568	-0.99191	up
RNF19B	0.5363293	10.143710	3.3729327	0.0010721	0.0194644	-0.99404	up
TYW3	-0.59443	8.7850933	-3.37156	0.0010768	0.0195062	-0.99804	down
LDHB	-0.67869	11.317797	-3.37005	0.0010821	0.0195721	-1.00243	down
CYP4F22	-0.71867	6.7449187	-3.36981	0.0010830	0.0195721	-1.00312	down
ISCA1L	0.9859719	9.9776788	3.3693357	0.0010846	0.0195882	-1.0045	up
OSBPL10	-0.99899	8.6710996	-3.36613	0.0010960	0.0197199	-1.01383	down

SMPDL3A	0.9200548	9.4993180	3.3658967	0.0010968	0.0197199	-1.01449	up
CLIC5	-0.65909	6.0567608	-3.36268	0.0011083	0.0198451	-1.02383	down
IFT57	-0.54237	7.3098713	-3.36178	0.0011115	0.0198825	-1.02645	down
FAM8A1	0.5455173	9.3715856	3.3607931	0.0011151	0.0199092	-1.02931	up
SGK1	-0.51534	10.483338	-3.3588	0.0011223	0.0200065	-1.03508	down
IL18R1	1.1435792	10.269470	3.3568911	0.0011292	0.0200717	-1.04062	up
BLR1	-0.61915	6.0586628	-3.35617	0.0011319	0.0200943	-1.04272	down
FAM72D	0.5205031	5.4113168	3.3557202	0.0011335	0.0201043	-1.04401	up
CEP68	-0.65586	7.4261354	-3.35265	0.0011448	0.0202761	-1.0529	down
LOC645385	-0.53916	11.944610	-3.35113	0.0011505	0.0203471	-1.05729	down
ASGR2	0.8154155	7.7397349	3.3500996	0.0011543	0.0203716	-1.06029	up
TCTN1	-0.52159	7.9633191	-3.35006	0.0011544	0.0203716	-1.06039	down
RPS18	-0.55345	13.375665	-3.34993	0.0011549	0.0203716	-1.06078	down
BCAT1	0.6063653	8.0792452	3.3462569	0.0011687	0.0205200	-1.0714	up
SLPI	1.2933579	8.0237149	3.3452547	0.0011725	0.0205750	-1.0743	up
CARD17	1.0486289	7.6775610	3.3431239	0.0011806	0.0206072	-1.08045	up
LOC642684	0.9178292	7.5521837	3.3425988	0.0011826	0.0206090	-1.08197	up
ZSCAN18	-0.50445	9.5743326	-3.3389	0.0011968	0.0207917	-1.09264	down
RNU4-1	0.7572381	7.1784715	3.3372334	0.0012032	0.0208694	-1.09746	up
C16orf7	0.7447304	9.6318288	3.3358657	0.0012085	0.0209304	-1.1014	up
LILRA3	0.8382840	12.133351	3.3358136	0.0012087	0.0209304	-1.10155	up
GRAMD1B	0.6367719	7.3075891	3.3342969	0.0012147	0.0209636	-1.10593	up
CICE	0.5183932	10.077836	3.3310629	0.0012274	0.0210788	-1.11524	up
PDCD2L	-0.6414	8.0094558	-3.33067	0.0012289	0.0210822	-1.11637	down
DNASE1L3	-0.6651	6.0143188	-3.32792	0.0012398	0.0211885	-1.12428	down
HLA-DOB	-0.79705	9.5153963	-3.32474	0.0012526	0.0213267	-1.13345	down
SLC2A14	0.9124645	7.8451159	3.3209908	0.0012678	0.0215255	-1.14422	up
CYP4F12	-0.65047	6.0143065	-3.3193	0.0012747	0.0215971	-1.14907	down
CCL3	0.5458139	7.4377763	3.3185664	0.0012777	0.0216003	-1.15119	up
BST1	0.7894120	9.2625772	3.3163083	0.0012870	0.0216874	-1.15767	up
DSE	0.5844382	9.9070234	3.3158046	0.0012890	0.0217109	-1.15912	up
TARBP1	-0.58717	8.4582175	-3.31482	0.0012931	0.0217559	-1.16193	down
AKR1B1	-0.52684	11.253073	-3.31271	0.0013019	0.0218802	-1.16801	down
CCDC66	-0.5135	7.9165511	-3.31221	0.0013040	0.0218802	-1.16942	down
GSTM4	-0.56854	6.9757527	-3.31009	0.0013129	0.0219518	-1.17552	down
CD79A	-0.96548	8.9027331	-3.30995	0.0013135	0.0219518	-1.17592	down
INADL	-0.70696	8.5280466	-3.30915	0.0013168	0.0219814	-1.1782	down
PNOC	-0.74247	8.1524425	-3.30631	0.0013289	0.0221325	-1.18633	down
ABHD14A	-0.54234	8.4503008	-3.30507	0.0013342	0.0221855	-1.18989	down
MAN2A2	0.5015837	8.7128166	3.3032059	0.0013422	0.0222945	-1.19522	up
DTX3L	0.6031580	9.0589388	3.3029074	0.0013434	0.0223041	-1.19608	up
OC1001304	-0.52667	7.8205190	-3.30142	0.0013498	0.0223867	-1.20033	down
JAK2	0.6076305	9.1448443	3.2984728	0.0013626	0.0225393	-1.20876	up
FCER1G	0.6619659	13.805122	3.2962195	0.0013725	0.0226545	-1.2152	up
ENO2	-0.5156	7.4304879	-3.29412	0.0013817	0.0227590	-1.22119	down
LOC646836	-0.70655	6.9955817	-3.29329	0.0013854	0.0228079	-1.22358	down
CD24	-0.53161	6.6233240	-3.29305	0.0013864	0.0228129	-1.22424	down
HLA-DOA	-0.63224	9.7237492	-3.29281	0.0013875	0.0228186	-1.22493	down
CNIH4	0.7001127	10.794704	3.2913287	0.0013941	0.0228790	-1.22916	up
CXCR5	-1.08789	9.9189781	-3.29042	0.0013981	0.0228975	-1.23177	down
LOC645822	0.5980591	7.4961425	3.2903578	0.0013984	0.0228975	-1.23193	up

LOC645436	-0.52363	11.511422	-3.29026	0.0013989	0.0228975	-1.23222	down
GLUL	0.7015158	7.2368767	3.2899448	0.0014003	0.0229085	-1.23311	up
FTHL2	0.8824599	11.982705	3.2857796	0.0014190	0.0231534	-1.24499	up
LTF	0.7824527	7.1991548	3.2854210	0.0014206	0.0231571	-1.24601	up
LOC285359	-0.54278	7.7224231	-3.28052	0.0014430	0.0234365	-1.25997	down
GRINA	0.7268212	10.880924	3.2788145	0.0014508	0.0235277	-1.26482	up
XK	1.2927105	9.6171792	3.2778881	0.0014551	0.0235851	-1.26746	up
SELM	-0.60612	7.4981703	-3.27736	0.0014575	0.0236122	-1.26894	down
SNX10	0.7357266	9.6893244	3.2748325	0.0014693	0.0237788	-1.27614	up
LOC642120	0.5562421	6.9077780	3.2736954	0.0014747	0.0238527	-1.27937	up
FTO	-0.53514	7.0446805	-3.27112	0.0014868	0.0240237	-1.28668	down
ZNF17	-0.50125	8.0775503	-3.27053	0.0014896	0.0240446	-1.28837	down
LOC728126	-0.60633	13.159412	-3.26938	0.0014950	0.0241200	-1.29162	down
LARGE	-0.7009	6.6046655	-3.26869	0.0014983	0.0241487	-1.2936	down
SDSL	0.5534996	7.5564568	3.2668017	0.0015073	0.0242687	-1.29895	up
EMILIN2	0.5867121	10.704995	3.2649441	0.0015163	0.0243678	-1.30421	up
CCDC106	-0.7039	8.1263619	-3.26378	0.0015219	0.0244276	-1.30752	down
GPR114	-0.74522	9.2783881	-3.26127	0.0015340	0.0245659	-1.31463	down
SNRPD3	-0.53192	8.9522232	-3.26113	0.0015347	0.0245659	-1.31502	down
H1F0	0.9410332	10.289251	3.2586780	0.0015467	0.0246905	-1.32197	up
PLOD2	0.8142332	7.7429335	3.2572576	0.0015537	0.0247167	-1.32599	up
LOC441019	0.7176833	8.2957915	3.2568672	0.0015556	0.0247167	-1.32709	up
ZMYND19	-0.50901	7.8756427	-3.25357	0.0015719	0.0249026	-1.33642	down
LOC651524	0.5154946	6.8881179	3.2494179	0.0015928	0.0251164	-1.34815	up
PSG9	0.8158390	6.7049078	3.2460748	0.0016097	0.0253073	-1.35759	up
SCGB3A1	-1.17451	6.7499131	-3.24522	0.0016141	0.0253481	-1.36001	down
SIGLEC10	-0.61504	10.009664	-3.24461	0.0016172	0.0253481	-1.36173	down
MAD1L1	-0.60629	9.1315507	-3.24457	0.0016174	0.0253481	-1.36183	down
)C1001325	0.6508906	7.8355592	3.2419991	0.0016306	0.0255079	-1.36909	up
PRAM1	0.5651049	9.1352188	3.2415571	0.0016328	0.0255125	-1.37034	up
SAMD9L	0.6791640	11.628697	3.2400124	0.0016408	0.0256048	-1.37469	up
HEPACAM2	0.9971197	7.0542934	3.2395221	0.0016434	0.0256190	-1.37607	up
NOD2	0.6740466	10.395687	3.2378410	0.0016521	0.0257146	-1.38081	up
TCL1A	-1.00778	7.8571949	-3.23602	0.0016617	0.0258272	-1.38595	down
VAMP5	0.5679183	11.875860	3.2357778	0.0016629	0.0258340	-1.38662	up
LOC646688	-0.64433	12.818726	-3.23337	0.0016756	0.0259544	-1.39341	down
DSC1	-0.55567	5.8061489	-3.23233	0.0016811	0.0260007	-1.39631	down
LOC390530	0.5565608	9.4550163	3.2310106	0.0016881	0.0260818	-1.40003	up
TWISTNB	-0.52183	7.4735829	-3.22405	0.0017256	0.0264541	-1.4196	down
HLA-DRB6	1.6514850	10.906058	3.2234257	0.0017289	0.0264698	-1.42134	up
)C1001343	0.5959566	6.0704122	3.2217426	0.0017381	0.0265638	-1.42606	up
CD2	-0.70015	12.129219	-3.22056	0.0017446	0.0266160	-1.42937	down
METTL9	0.7622367	8.8380559	3.2181809	0.0017577	0.0267772	-1.43605	up
)C1001296	-0.58861	7.7394860	-3.21617	0.0017688	0.0269141	-1.44168	down
EPSTI1	0.6405024	13.081379	3.2148961	0.0017760	0.0269489	-1.44526	up
TMCC2	1.3040494	8.5820221	3.2145943	0.0017776	0.0269501	-1.4461	up
MGC13057	1.0115285	9.9517277	3.2141412	0.0017802	0.0269741	-1.44737	up
PTPLA	0.7502599	7.4966073	3.2112800	0.0017963	0.0271135	-1.45538	up
MGC15763	-0.71246	8.4900962	-3.21097	0.0017980	0.0271135	-1.45625	down
CTRC	0.5050830	7.0495875	3.2107151	0.0017994	0.0271135	-1.45696	up
PNPLA7	-0.51583	8.4975033	-3.21053	0.0018005	0.0271135	-1.45749	down

SORT1	0.7764348	9.3232235	3.2098670	0.0018042	0.0271455	-1.45933	up
BCL3	0.6281006	10.382565	3.2092412	0.0018078	0.0271711	-1.46108	up
SCARNA9	0.5188960	9.3863019	3.2075440	0.0018174	0.0272397	-1.46583	up
OLR1	1.0255972	7.0691457	3.2067264	0.0018221	0.0272811	-1.46812	up
CPEB4	0.6992082	9.2799120	3.2065850	0.0018229	0.0272811	-1.46851	up
WSB1	0.6775735	9.5272276	3.2052962	0.0018303	0.0273136	-1.47211	up
FLJ36644	0.5412075	6.9888956	3.2033332	0.0018416	0.0274431	-1.4776	up
IGFBP4	-0.62467	7.2123977	-3.19972	0.0018626	0.0276508	-1.48768	down
BRI3	0.5686323	11.995297	3.1968376	0.0018795	0.0278883	-1.49572	up
HSPA7	0.6401649	9.7757810	3.1955638	0.0018870	0.0279601	-1.49927	up
CEBPD	0.6279399	13.040759	3.1945986	0.0018927	0.0280183	-1.50196	up
LOC642934	-0.77448	8.5032851	-3.19353	0.0018990	0.0280929	-1.50495	down
TMEM144	0.7364214	6.7699127	3.1903070	0.0019183	0.0282886	-1.51392	up
CACNA1E	1.0597393	7.3187053	3.1901753	0.0019190	0.0282886	-1.51428	up
HIST1H2BD	0.7457594	10.993926	3.1887955	0.0019273	0.0283489	-1.51812	up
DPYD	0.5377479	9.0386621	3.1865809	0.0019407	0.0284580	-1.52428	up
LOC642178	-0.6683	7.4107405	-3.18655	0.0019409	0.0284580	-1.52436	down
TNFSF10	0.6761257	12.407763	3.1864698	0.0019414	0.0284580	-1.52459	up
ZNF438	0.7581436	8.9429107	3.1838907	0.0019571	0.0286478	-1.53176	up
C3AR1	0.7150367	8.8178344	3.1780538	0.0019931	0.0290124	-1.54797	up
[illegible]C1001309	0.9610333	7.5261988	3.1766665	0.0020017	0.0290440	-1.55182	up
HNRPA1L-2	-0.691	8.3804513	-3.1759	0.0020065	0.0290773	-1.55393	down
ST3GAL4	0.6859325	7.9749354	3.1746025	0.0020146	0.0291775	-1.55755	up
TRAT1	-0.6624	9.5059938	-3.17424	0.0020169	0.0291936	-1.55855	down
CROT	-0.53855	7.3812849	-3.17309	0.0020242	0.0292121	-1.56175	down
GALM	0.6147081	9.7081622	3.1719737	0.0020312	0.0292790	-1.56483	up
ZNF789	-0.54712	8.1301359	-3.17079	0.0020387	0.0293365	-1.5681	down
GAMT	-0.58608	8.3969317	-3.167	0.0020629	0.0295763	-1.5786	down
ADK	-0.54265	6.2235573	-3.16344	0.0020858	0.0298381	-1.58845	down
PGLYRP1	1.1586603	10.455144	3.1613647	0.0020993	0.0299762	-1.59419	up
SELP	0.7047699	6.9842929	3.1569892	0.0021281	0.0302626	-1.60627	up
RHOBTB3	-0.62477	7.5713995	-3.15378	0.0021494	0.0304193	-1.61512	down
LOC730631	0.6202108	6.2428350	3.1537246	0.0021497	0.0304193	-1.61528	up
GBP6	1.0737023	7.331131	3.1520921	0.0021607	0.0304640	-1.61978	up
LOC651149	-0.58134	9.0558715	-3.14943	0.0021786	0.0305873	-1.62712	down
C11orf80	-0.70821	7.5001623	-3.14935	0.0021791	0.0305873	-1.62735	down
IFIH1	0.5807677	10.56605	3.1487438	0.0021832	0.0306134	-1.62901	up
FTHL3	0.9839579	10.443935	3.1453210	0.0022065	0.0307909	-1.63843	up
FCER1A	-1.14387	9.6264810	-3.14512	0.0022078	0.0307909	-1.63898	down
IL1RN	0.9703414	9.9920475	3.1445325	0.0022119	0.0308147	-1.6406	up
CD72	-0.68144	8.8886450	-3.14451	0.0022120	0.0308147	-1.64066	down
LOC643313	0.8053586	10.627837	3.1440681	0.0022150	0.0308361	-1.64188	up
FLJ20273	0.9045782	8.6025443	3.1422263	0.0022277	0.0309786	-1.64695	up
LIME1	-0.66919	12.172374	-3.14179	0.0022307	0.0310071	-1.64816	down
GADD45B	0.5014632	10.301599	3.1406147	0.0022388	0.0311059	-1.65138	up
LOC730278	0.8200280	12.678634	3.1386269	0.0022527	0.0312287	-1.65684	up
RAB15	-0.53495	6.1862950	-3.13733	0.0022617	0.0312990	-1.6604	down
AGPAT9	0.7046480	10.381499	3.1361546	0.0022699	0.0313991	-1.66363	up
TRIM25	0.5298795	10.280991	3.1348036	0.0022794	0.0314971	-1.66734	up
[illegible]C1001321	0.6874949	8.1190058	3.1347189	0.0022800	0.0314971	-1.66757	up
LRRK2	0.7904022	9.6814695	3.1328730	0.0022931	0.0316077	-1.67264	up

TNFAIP8L1	-0.58656	9.2052664	-3.13207	0.0022987	0.0316303	-1.67483	down
)C1001333	-0.57111	11.447310	-3.1316	0.0023021	0.0316439	-1.67613	down
MT1A	0.6128515	12.432493	3.1307592	0.0023081	0.0317034	-1.67843	up
HNRPLL	0.5599400	7.8348726	3.1301058	0.0023127	0.0317536	-1.68023	up
RHD	1.2701892	7.7446222	3.1296090	0.0023163	0.0317830	-1.68159	up
LOC253039	-0.50943	9.9258055	-3.12952	0.0023169	0.0317830	-1.68183	down
ADM	0.9116550	12.453175	3.1287747	0.0023223	0.0317909	-1.68387	up
AGPAT5	-0.50652	8.5448266	-3.12768	0.0023302	0.0318673	-1.68689	down
RAB20	0.6434708	7.5048991	3.1257547	0.0023440	0.0320288	-1.69215	up
HIST1H1T	0.6149673	6.0453993	3.1226858	0.0023663	0.0322279	-1.70055	up
LOC641693	0.6214988	7.4629104	3.1226175	0.0023668	0.0322279	-1.70073	up
MOV10	0.5348841	9.4273418	3.1204004	0.0023830	0.0323786	-1.7068	up
FAR2	0.5156129	7.6731508	3.1184294	0.0023975	0.0324915	-1.71219	up
FHDC1	0.8058509	6.7230726	3.1157232	0.0024176	0.0326787	-1.71958	up
CHPT1	0.7732169	12.160887	3.1142526	0.0024285	0.0327986	-1.7236	up
AHI1	-0.53314	6.3791397	-3.11235	0.0024428	0.0329487	-1.72879	down
E2F5	-0.65277	9.4916211	-3.11166	0.0024480	0.0329774	-1.73068	down
TMEM118	-0.54515	7.8004889	-3.11076	0.0024548	0.0329972	-1.73314	down
LIMS2	-0.58794	6.9751736	-3.10872	0.0024702	0.0331196	-1.7387	down
)C1001294	-0.6141	6.3292953	-3.10821	0.0024741	0.0331430	-1.74008	down
TOR2A	0.7841867	7.6596693	3.1077355	0.0024777	0.0331771	-1.74137	up
)C1001322	0.6872889	7.8938395	3.1065817	0.0024865	0.0332666	-1.74452	up
AHSP	1.1141436	13.759974	3.1047719	0.0025003	0.0334092	-1.74945	up
SLC38A1	-0.54508	12.055646	-3.10414	0.0025052	0.0334470	-1.75117	down
AZU1	0.9780976	7.3097780	3.1036956	0.0025086	0.0334520	-1.75238	up
OTOF	1.6982397	8.3450895	3.0995257	0.0025409	0.0338219	-1.76373	up
LOC650898	0.8858596	8.7346798	3.0964090	0.0025653	0.0340312	-1.7722	up
YPEL4	0.8025324	6.6486734	3.0961950	0.0025669	0.0340391	-1.77278	up
VPREB3	-1.28261	10.416552	-3.09483	0.0025777	0.0341243	-1.77649	down
CD163	0.7344862	9.2580840	3.0923489	0.0025974	0.0342546	-1.78323	up
ZNF550	-0.53121	7.5953036	-3.08929	0.0026218	0.0344323	-1.79153	down
SEMA4A	0.6681142	9.6302646	3.0880195	0.0026320	0.0345086	-1.79497	up
NFE2	0.6418396	13.560268	3.0861847	0.0026468	0.0346450	-1.79995	up
)5-1022P6.	0.5720990	8.6906369	3.0850628	0.0026559	0.0347062	-1.80299	up
PIK3C2B	-0.50635	8.0541222	-3.08339	0.0026695	0.0348388	-1.80751	down
SNRNP48	-0.54219	6.6366693	-3.08269	0.0026752	0.0348570	-1.80941	down
RRP12	0.5436657	8.7773312	3.0785177	0.0027095	0.0352311	-1.8207	up
GYPB	1.5783691	10.488830	3.0705103	0.0027765	0.0358498	-1.84234	up
NFIB	0.5922560	7.0074931	3.0622553	0.0028471	0.0364815	-1.8646	up
MCTP2	0.7107072	8.8081349	3.0622165	0.0028475	0.0364815	-1.8647	up
FAM129C	-0.80362	8.3770555	-3.06087	0.0028592	0.0366016	-1.86833	down
CYP27A1	0.7020823	9.4437555	3.0599221	0.0028674	0.0366920	-1.87088	up
SLC1A5	0.8566151	9.3089928	3.0574505	0.0028890	0.0369386	-1.87753	up
TSPAN13	-0.85727	9.2654264	-3.0553	0.0029080	0.0371168	-1.88332	down
RAB30	-0.65681	7.9031408	-3.05372	0.0029219	0.0372481	-1.88755	down
GABARAPL2	0.5557637	13.567495	3.0516406	0.0029404	0.0373803	-1.89314	up
PDZD4	-0.52661	7.4189084	-3.05158	0.0029410	0.0373803	-1.89331	down
)C1001348	0.5337411	7.8265499	3.0514014	0.0029426	0.0373803	-1.89378	up
ACSL1	0.9724632	11.553297	3.0436236	0.0030128	0.0380893	-1.91465	up
PIK3AP1	0.8020894	9.6632502	3.0427823	0.0030205	0.0381226	-1.9169	up
HSPA6	0.6480660	9.0736697	3.0423457	0.0030245	0.0381577	-1.91807	up

TLR2	0.8431239	8.1914207	3.0371489	0.0030724	0.0386342	-1.93198	up
CA4	1.0300053	9.7551726	3.0350643	0.0030918	0.0388361	-1.93756	up
C7orf53	0.6458508	7.6373604	3.0335696	0.0031058	0.0389349	-1.94155	up
ERMAP	0.6245053	8.3525084	3.0333177	0.0031082	0.0389349	-1.94223	up
CTSA	0.6349740	9.6601348	3.0333056	0.0031083	0.0389349	-1.94226	up
MBOAT2	0.6789744	6.8544359	3.0327480	0.0031135	0.0389687	-1.94375	up
IL15RA	0.5097804	6.6004751	3.0255243	0.0031822	0.0395600	-1.96303	up
LOC2558090	0.6202693	9.3577806	3.0237722	0.0031990	0.0396910	-1.9677	up
FAM3C	-0.57663	7.5423694	-3.02358	0.0032009	0.0396987	-1.96822	down
CD14	0.6271289	12.462178	3.0233491	0.0032031	0.0397103	-1.96883	up
TOP1P2	0.7004117	7.7420235	3.0226813	0.0032096	0.0397747	-1.97061	up
ZNF365	-0.51414	5.5812344	-3.02188	0.0032173	0.0398548	-1.97274	down
IFI35	0.6078948	11.673675	3.0192025	0.0032434	0.0400590	-1.97987	up
CYP1B1	1.1020097	9.4579937	3.0155707	0.0032790	0.0403380	-1.98954	up
ATP8B4	0.5452143	9.7622536	3.0143476	0.0032911	0.0404347	-1.99279	up
HSPC159	0.5794776	7.208897	3.0133739	0.0033008	0.0404923	-1.99538	up
BPGM	0.8642223	6.8244068	3.0091814	0.0033427	0.0408760	-2.00652	up
LOC3893860	0.5574347	8.8398017	3.0029387	0.0034059	0.0415254	-2.02308	up
SLC22A16	0.6956069	7.3869372	3.0017852	0.0034177	0.0415907	-2.02613	up
DDX60L	0.7274620	9.5816807	3.0017662	0.0034179	0.0415907	-2.02618	up
CD247	-0.65295	13.168597	-3.00128	0.0034229	0.0416303	-2.02746	down
CTLA4	-0.543	8.3809452	-3.00049	0.0034310	0.0416647	-2.02956	down
WDR26	0.6276664	10.162243	3.0000459	0.0034356	0.0417043	-2.03074	up
ZNF91	-0.57644	8.6523240	-2.9989	0.0034474	0.0417828	-2.03376	down
RGL1	0.6810511	8.1331240	2.9972364	0.0034647	0.0418952	-2.03818	up
LOC728820	-0.60014	11.416735	-2.99553	0.0034825	0.0420619	-2.0427	down
2-Mar	0.5281972	9.4499894	2.9930082	0.0035088	0.0422990	-2.04936	up
HNRNPA3P1	0.5282573	7.7825738	2.9879436	0.0035624	0.0427645	-2.06274	up
MAFB	0.5555751	10.680287	2.9832278	0.0036130	0.0432230	-2.07517	up
PILRA	0.5517884	9.5366100	2.9823718	0.0036222	0.0433007	-2.07743	up
BMP2K	0.7551250	7.4472940	2.9815958	0.0036306	0.0433682	-2.07948	up
JAZF1	0.6343847	10.511226	2.9792533	0.0036561	0.0435897	-2.08565	up
SLC6A9	1.0489377	7.5778484	2.9779766	0.0036701	0.0437064	-2.08901	up
CLEC1B	0.9604404	9.4062404	2.9774707	0.0036756	0.0437392	-2.09034	up
TXNDC3	0.6500303	8.2439651	2.9761151	0.0036905	0.0438673	-2.09391	up
FTHL11	0.7411464	12.315550	2.9758822	0.0036931	0.0438673	-2.09452	up
MYL9	0.9979853	8.6716120	2.9742390	0.0037112	0.0439963	-2.09884	up
ZNF827	-0.6099	8.9073955	-2.97224	0.0037334	0.0441754	-2.10409	down
FNDC3B	0.5601890	10.445253	2.9708325	0.0037491	0.0443282	-2.10779	up
SOX6	0.6280749	6.3163915	2.9672921	0.0037888	0.0446298	-2.11709	up
NACC2	0.5558752	8.4069082	2.9669943	0.0037922	0.0446298	-2.11787	up
IL18RAP	0.7043039	12.459990	2.9629267	0.0038384	0.0450292	-2.12854	up
LOC6537780	0.7574271	12.337975	2.9609567	0.0038609	0.0452543	-2.1337	up
PLEKHA1	-0.61295	9.7970773	-2.95681	0.0039088	0.0457178	-2.14457	down
ITM2A	-0.62413	13.012097	-2.9566	0.0039111	0.0457281	-2.1451	down
GAPDHL6	0.5457818	7.3864714	2.9533503	0.0039491	0.0460179	-2.15361	up
JUNB	0.5613309	9.5807965	2.9511136	0.0039754	0.0462728	-2.15945	up
P2RX1	0.5318857	8.6946292	2.9457740	0.0040388	0.0468028	-2.17339	up
)C1001336'	-0.70657	12.340856	-2.94547	0.0040424	0.0468162	-2.17418	down
S1PR1	-0.64649	9.7544854	-2.94543	0.0040429	0.0468162	-2.17429	down
BMX	0.6977643	7.2961973	2.9415548	0.0040896	0.0471477	-2.18439	up

ANPEP	0.7256114	9.1183685	2.9407533	0.0040993	0.0472070	-2.18648	up
HK3	0.7553541	10.467468	2.9394378	0.0041153	0.0473397	-2.18991	up
CHMP5	0.7044122	9.8677430	2.9380438	0.0041323	0.0475179	-2.19354	up
)C1001296	-0.67286	12.086057	-2.93684	0.0041471	0.0475475	-2.19668	down
GPR109A	0.7647504	8.1543538	2.9302855	0.0042281	0.0482137	-2.21371	up
PPP1R3B	0.5673647	6.9454952	2.9288364	0.0042462	0.0483676	-2.21747	up
GPA33	-0.51212	8.5062930	-2.92783	0.0042588	0.0484937	-2.22008	down
NCALD	-0.64293	9.3266694	-2.92699	0.0042694	0.0485616	-2.22227	down
C6orf173	0.6365982	7.6621076	2.9237660	0.0043101	0.0488483	-2.23063	up
TMEM156	-0.59708	7.6227094	-2.9223	0.0043288	0.0489718	-2.23443	down
SPIB	-0.79148	8.6298140	-2.92185	0.0043345	0.0490010	-2.23559	down
SIGLEC5	0.5121449	7.3634464	2.9212206	0.0043426	0.0490703	-2.23722	up
SERINC5	-0.58738	6.6222218	-2.91848	0.0043777	0.0492261	-2.24432	down
)C1001308	0.7143578	7.5191711	2.9146627	0.0044272	0.0495849	-2.2542	up
RPL5	-0.51552	12.671436	-2.91258	0.0044545	0.0497659	-2.25959	down
BCL6	0.7119778	12.926782	2.9099995	0.0044883	0.0500774	-2.26625	up
CDA	0.7106325	10.320364	2.9071358	0.0045262	0.0503349	-2.27364	up
KIAA1539	0.5397977	10.417971	2.9063344	0.0045369	0.0504356	-2.27571	up
ZNF135	-0.59888	6.4642316	-2.90368	0.0045723	0.0507043	-2.28255	down
C4orf18	0.5941118	9.2573671	2.9000931	0.0046207	0.0511141	-2.29179	up
LOC390354	-0.62161	13.633312	-2.89892	0.0046365	0.0512103	-2.2948	down
CD274	0.8582926	6.0767061	2.8941034	0.0047024	0.0517091	-2.3072	up
TPM2	-0.56994	7.4988171	-2.89296	0.0047181	0.0518094	-2.31013	down
GALNT14	0.6232205	6.5615511	2.8894954	0.0047662	0.0521865	-2.31904	up
MS4A6A	0.5070725	12.267255	2.8891964	0.0047704	0.0521865	-2.31981	up
HES6	0.8726967	8.5173044	2.8890527	0.0047724	0.0521865	-2.32018	up
GP6	0.7148500	8.0762232	2.8878068	0.0047898	0.0522859	-2.32338	up
STEAP4	0.7908077	8.4303226	2.8801991	0.0048974	0.0530364	-2.34288	up
RNASE3	-1.20206	8.8428541	-2.87805	0.0049282	0.0532372	-2.34838	down
RIOK3	0.7584107	12.788355	2.8760735	0.0049566	0.0534571	-2.35343	up
PADI4	0.7320090	11.790999	2.8747622	0.0049756	0.0536249	-2.35678	up
LOC646753	-0.67286	11.091474	-2.87351	0.0049938	0.0537473	-2.35999	down
IFIT3	1.0100627	10.474711	2.8728843	0.0050029	0.0537679	-2.36158	up
KIFAP3	-0.53213	8.7617096	-2.87283	0.0050037	0.0537679	-2.36173	down
OSTalpha	0.7121156	6.3076985	2.8724703	0.0050089	0.0537995	-2.36264	up
KCNJ15	0.6006595	6.7221537	2.8691392	0.0050577	0.0541570	-2.37115	up
FAM26F	0.7030574	8.4815886	2.8635097	0.0051412	0.0548451	-2.3855	up
CDC25A	0.5472517	6.4992671	2.8617011	0.0051683	0.0550966	-2.39011	up
LOC644642	0.5657772	9.5632369	2.8595496	0.0052007	0.0553697	-2.39559	up
LMO2	0.5097125	11.017218	2.8576358	0.0052296	0.0555809	-2.40046	up
NCF2	0.5554941	11.565683	2.8554739	0.0052625	0.0557797	-2.40595	up
KCTD14	0.7034685	6.4262026	2.8553750	0.0052640	0.0557797	-2.4062	up
KIAA0367	0.8404803	6.7808154	2.8547066	0.0052742	0.0558473	-2.4079	up
ALPL	1.1089078	12.670330	2.8541301	0.0052831	0.0559219	-2.40937	up
RTP4	0.6488949	8.8057866	2.8499484	0.0053474	0.0564954	-2.41999	up
CST7	0.6402401	12.258733	2.8491333	0.0053601	0.0565005	-2.42206	up
GYPE	1.3898578	11.245316	2.8490388	0.0053616	0.0565005	-2.4223	up
TFDP1	0.6118975	11.545410	2.8488650	0.0053643	0.0565005	-2.42274	up
SBNO2	0.5604491	9.1726341	2.8487999	0.0053653	0.0565005	-2.4229	up
SERPINA1	0.5622215	7.1895653	2.8487210	0.0053665	0.0565005	-2.4231	up
POM121L4P	0.5201334	6.7007734	2.8470407	0.0053927	0.0566355	-2.42736	up

FLJ41603	0.5045428	6.4725069	2.8468927	0.0053950	0.0566355	-2.42774	up
GADD45A	0.6661578	8.4915196	2.8440096	0.0054401	0.0568758	-2.43505	up
ECHDC3	0.6811930	7.4939600	2.8420355	0.0054713	0.0571064	-2.44005	up
USP12	0.5945272	7.2248119	2.8385771	0.0055262	0.0574436	-2.4488	up
TREML3	0.7556298	8.7090651	2.8380326	0.0055349	0.0574474	-2.45017	up
PHTF1	0.5219786	9.3315304	2.8368908	0.0055532	0.0575409	-2.45306	up
GZMM	-0.64262	9.9269121	-2.83167	0.0056375	0.0581842	-2.46626	down
SAMD4A	0.5147285	7.4857189	2.8276782	0.0057027	0.0586836	-2.47632	up
KCNE1	0.8749176	6.3020966	2.8213068	0.0058082	0.0593421	-2.49237	up
LIMA1	-0.55448	7.3041059	-2.82084	0.0058160	0.0594025	-2.49354	down
)C1001328	0.5301442	7.1732363	2.8202065	0.0058266	0.0594725	-2.49514	up
PPAP2C	0.5285848	7.9612894	2.8184016	0.0058569	0.0597341	-2.49968	up
NCF1	0.5217756	11.690852	2.8182897	0.0058588	0.0597341	-2.49996	up
C21orf7	-0.60985	10.970094	-2.81823	0.0058599	0.0597341	-2.50012	down
LILRB3	0.6550576	13.358277	2.8179461	0.0058646	0.0597478	-2.50082	up
LOC439949	-0.70788	9.5329662	-2.81702	0.0058802	0.0598445	-2.50315	down
SOCS3	0.7516068	7.8233583	2.8167468	0.0058848	0.0598720	-2.50384	up
C5AR1	0.5912984	11.125953	2.8142206	0.0059277	0.0601133	-2.51018	up
NQO2	0.5469086	9.4672899	2.8127706	0.0059524	0.0602474	-2.51382	up
CMTM5	0.6887810	8.5910943	2.8102394	0.0059958	0.0606084	-2.52017	up
DOK3	0.5669856	7.8710003	2.8081263	0.0060322	0.0608593	-2.52547	up
CD19	-0.79125	10.606594	-2.80658	0.0060591	0.0610323	-2.52936	down
C9orf106	0.5983269	7.7174122	2.8053464	0.0060805	0.0611379	-2.53244	up
NCF1B	0.5944007	8.7855006	2.8024321	0.0061314	0.0614851	-2.53973	up
MAZ	0.6655946	7.9846749	2.8019697	0.0061396	0.0615029	-2.54089	up
CYP4F3	0.6950206	10.839859	2.8015782	0.0061465	0.0615374	-2.54187	up
BASP1	0.6310479	13.937955	2.7989029	0.0061937	0.0618526	-2.54856	up
SLAMF8	0.5450514	7.1439420	2.7961441	0.0062427	0.0622238	-2.55545	up
KREMEN1	1.0107015	8.3836365	2.7952377	0.0062589	0.0623061	-2.55771	up
CD52	-0.57879	13.552215	-2.78418	0.0064595	0.0635978	-2.58527	down
CCR1	0.6942633	10.928239	2.7838015	0.0064665	0.0636349	-2.58622	up
SLC22A15	0.6579294	8.3321058	2.7783924	0.0065669	0.0641729	-2.59967	up
EBF1	-0.71876	8.5469038	-2.77626	0.0066069	0.0644431	-2.60496	down
FTHL12	0.8414334	12.611833	2.7688312	0.0067478	0.0653717	-2.62338	up
GBP5	0.7600268	12.048850	2.7668592	0.0067857	0.0656257	-2.62827	up
OSM	0.6666795	8.3167169	2.7657031	0.0068080	0.0657317	-2.63113	up
LOC642035	-0.6113	8.6990844	-2.76411	0.0068388	0.0659678	-2.63506	down
SAMSN1	0.6103149	9.6815357	2.7628242	0.0068638	0.0661284	-2.63825	up
C8orf13	-0.5255	6.6312310	-2.76121	0.0068953	0.0662894	-2.64224	down
UQCRB	0.6020385	8.1470674	2.7590363	0.0069379	0.0666377	-2.64761	up
SMOX	0.9467630	11.482407	2.7583875	0.0069506	0.0666991	-2.64921	up
SIAH2	0.8128739	11.587244	2.7554480	0.0070087	0.0671211	-2.65646	up
)C1001347	0.5636493	7.5207804	2.7549232	0.0070191	0.0671717	-2.65776	up
PCGF5	0.5797623	8.0258234	2.7541336	0.0070348	0.0672605	-2.65971	up
LOC730234	0.5480386	8.4188456	2.7498853	0.0071198	0.0677283	-2.67017	up
GCLC	0.6976597	8.8854058	2.7476917	0.0071641	0.0679901	-2.67557	up
ANK1	1.0431310	8.1873670	2.7465689	0.0071868	0.0681383	-2.67834	up
MGC3020	-0.55407	8.8422732	-2.74651	0.0071881	0.0681383	-2.67849	down
TSEN54	-0.53757	8.1284798	-2.74597	0.0071991	0.0681909	-2.67982	down
LOC441268	0.6407631	9.4126711	2.7439358	0.0072405	0.0684353	-2.68481	up
GLDN	0.6786547	5.0757434	2.7419995	0.0072801	0.0686862	-2.68957	up

ZNF287	-0.52507	6.1186681	-2.74095	0.0073017	0.0687782	-2.69215	down
PVRIG	-0.61451	10.199343	-2.73616	0.0074010	0.0693060	-2.70391	down
GLT1D1	0.5538324	7.8802653	2.7337166	0.0074520	0.0695706	-2.70989	up
LOC6464630	0.6591808	9.7055339	2.7336421	0.0074536	0.0695706	-2.71007	up
KLF1	0.8672051	8.6857290	2.7272885	0.0075879	0.0704898	-2.72562	up
SH2D1A	-0.57765	10.912196	-2.7226	0.0076884	0.0710983	-2.73707	down
LOC7284170	0.5853523	8.7626690	2.7206246	0.0077312	0.0713872	-2.7419	up
HIST1H2BF	0.6047145	6.8480183	2.7183678	0.0077803	0.0716416	-2.74741	up
PRTN3	0.7482825	7.0571455	2.7178550	0.0077915	0.0716817	-2.74866	up
ACSL4	0.5773469	9.8513765	2.7168846	0.0078127	0.0717254	-2.75102	up
CLEC4D	0.8371308	8.2961526	2.7161589	0.0078286	0.0717502	-2.75279	up
HMGB2	0.5732194	9.5630673	2.7136489	0.0078839	0.0720882	-2.75891	up
RTN1	-0.65554	8.4447240	-2.71117	0.0079388	0.0723460	-2.76495	down
LOC6417100	0.5903368	8.9602242	2.7074005	0.0080229	0.0728719	-2.77411	up
LOC6421030	0.9370712	9.3760812	2.7064874	0.0080434	0.0729949	-2.77632	up
PVRL2	0.5908046	7.8372501	2.7055289	0.0080650	0.0730641	-2.77865	up
ABCA1	0.5735490	11.375340	2.7035982	0.0081086	0.0732692	-2.78334	up
LOC388339	-0.54476	11.989971	-2.70222	0.0081398	0.0734863	-2.78668	down
LOC388654	-0.57267	13.354404	-2.70097	0.0081682	0.0735791	-2.78971	down
ZDHHC19	1.4086728	8.5131905	2.6996888	0.0081976	0.0737689	-2.79282	up
MIR21	0.6815640	9.0338127	2.6989029	0.0082156	0.0738113	-2.79473	up
11-529I10	0.8561762	7.9870411	2.6969333	0.0082608	0.0740802	-2.7995	up
TSTA3	0.8706439	9.6648453	2.6968441	0.0082629	0.0740802	-2.79972	up
LOC6517380	0.7842650	11.175241	2.6966776	0.0082667	0.0740802	-2.80012	up
SPTA1	0.7694701	7.7277843	2.6947386	0.0083115	0.0743121	-2.80481	up
TNFSF13B	0.6288655	12.780173	2.6929750	0.0083524	0.0745086	-2.80908	up
CECR6	0.5563129	8.1177478	2.6918921	0.0083777	0.0746068	-2.8117	up
IL27	0.6779064	6.0451507	2.6902867	0.0084152	0.0748348	-2.81558	up
BLK	-0.70101	9.8122830	-2.69012	0.0084191	0.0748486	-2.81599	down
SLAMF1	-0.54019	9.1645589	-2.68874	0.0084515	0.0750512	-2.81932	down
TNFRSF13C	-0.71584	7.8461454	-2.68765	0.0084772	0.0751942	-2.82195	down
C11orf82	0.5167703	8.2885133	2.6854305	0.0085297	0.0754476	-2.82731	up
LY96	0.9597910	11.353316	2.6828226	0.0085917	0.0758464	-2.8336	up
IFRD1	0.5237983	9.7531879	2.6825251	0.0085988	0.0758878	-2.83432	up
RBM47	0.5922802	11.022689	2.6746127	0.0087897	0.0771539	-2.85337	up
CLEC4C	-0.57644	7.2132870	-2.67037	0.0088936	0.0777766	-2.86356	down
TERF2IP	0.5517603	11.540755	2.6680348	0.0089513	0.0780348	-2.86918	up
USP10	0.5670393	11.265616	2.6675830	0.0089625	0.0780675	-2.87026	up
COX7B	0.6340919	9.4669132	2.6673507	0.0089683	0.0780957	-2.87082	up
SPI1	0.5219978	12.080334	2.6672435	0.0089710	0.0780957	-2.87107	up
CSTA	0.5610146	7.9862302	2.6627801	0.0090824	0.0788280	-2.88178	up
GPR109B	0.7394911	8.3167578	2.6627769	0.0090825	0.0788280	-2.88178	up
FAH	0.5457939	7.7727567	2.6607671	0.0091331	0.0791299	-2.8866	up
IER3	0.5848822	9.5694922	2.6602072	0.0091472	0.0791711	-2.88794	up
BEST1	0.5837594	8.3125407	2.6596080	0.0091623	0.0792804	-2.88937	up
SH3KBP1	-0.66216	10.191219	-2.65667	0.0092369	0.0797058	-2.89639	down
SAMD14	0.6922594	7.2875105	2.6557029	0.0092616	0.0798538	-2.89871	up
C13orf15	-0.57849	12.158378	-2.65429	0.0092978	0.0800777	-2.90209	down
TMEM56	0.8981234	6.9157327	2.6507084	0.0093901	0.0805412	-2.91064	up
MATK	-0.5478	6.7985617	-2.65051	0.0093952	0.0805633	-2.91112	down
MMRN1	0.7793920	7.5460945	2.6486973	0.0094422	0.0808782	-2.91544	up

FHL2	0.6256744	8.9401065	2.6450440	0.0095377	0.0814978	-2.92415	up
ABCA13	0.6753960	5.1064399	2.6433504	0.0095822	0.0816543	-2.92818	up
RASGRP1	-0.62312	9.8012486	-2.63887	0.0097009	0.0822730	-2.93884	down
C1001284	0.5430060	7.6295731	2.6375362	0.0097365	0.0824743	-2.94201	up
PROS1	0.8427727	8.3473283	2.6330819	0.0098563	0.0832843	-2.95259	up
HLA-DQA1	-0.62868	12.002321	-2.63231	0.0098772	0.0833996	-2.95443	down
IFI44	0.8977072	11.750152	2.6319184	0.0098878	0.0834563	-2.95535	up
OSBP2	1.2140987	10.709954	2.6315797	0.0098970	0.0834765	-2.95615	up
PLXNC1	0.5585143	9.7133947	2.6308680	0.0099163	0.0836170	-2.95784	up
ITGB3	0.7255126	7.8571801	2.6289040	0.0099698	0.0838357	-2.9625	up
FURIN	0.6307891	9.1325993	2.6263470	0.0100399	0.0841175	-2.96855	up
TIFA	0.5518826	7.7834650	2.6250369	0.0100759	0.0843298	-2.97165	up
SLC2A11	0.6700477	8.4250070	2.6205145	0.0102014	0.0851083	-2.98235	up
CA2	0.9032032	10.322538	2.6184675	0.0102586	0.0854627	-2.98718	up
TCL6	-0.53646	6.2863828	-2.617	0.0102999	0.0856569	-2.99066	down
AQP9	0.6209064	13.802831	2.6164858	0.0103143	0.0857082	-2.99186	up
TMEM140	0.5883958	12.232761	2.6107532	0.0104769	0.0865387	-3.00538	up
OASL	0.8280183	10.951294	2.6102931	0.0104901	0.0865801	-3.00646	up
TMEM63B	0.5336266	7.9600050	2.6102657	0.0104908	0.0865801	-3.00653	up
TFDP2	0.5912709	7.9210033	2.6079053	0.0105585	0.0869106	-3.01208	up
LOC6464340	0.5181524	7.9418815	2.6072962	0.0105761	0.0870322	-3.01352	up
REPS2	0.5710837	9.5718070	2.6058877	0.0106167	0.0872713	-3.01683	up
CMBL	0.8930672	7.9781630	2.6051425	0.0106383	0.0873155	-3.01858	up
PINK1	0.6654539	9.8667015	2.6044673	0.0106579	0.0874305	-3.02017	up
CCPG1	0.5218232	10.381597	2.6029046	0.0107033	0.0876202	-3.02384	up
GK	0.7589980	10.00868	2.6024124	0.0107176	0.0876235	-3.025	up
COBLL1	-0.60419	9.3786539	-2.59917	0.0108127	0.0881481	-3.03262	down
NP	0.6494911	10.857649	2.5987444	0.0108251	0.0882096	-3.03361	up
GBP3	0.5619030	6.4434115	2.5959656	0.0109071	0.0887341	-3.04013	up
LOC7290100	0.5318145	7.6614134	2.5950062	0.0109356	0.0888966	-3.04238	up
ALPK1	0.6117743	9.6552205	2.5938308	0.0109705	0.0890563	-3.04513	up
ZMYND15	0.5839661	7.3160091	2.5937142	0.0109740	0.0890563	-3.0454	up
LBH	-0.55466	9.2167791	-2.5922	0.0110193	0.0892304	-3.04895	down
PCOLCE2	0.6533264	6.0784724	2.5912625	0.0110473	0.0892739	-3.05114	up
C6orf150	0.5541150	7.8984662	2.5912599	0.0110473	0.0892739	-3.05115	up
ZBP1	0.5410980	11.892629	2.5904265	0.0110723	0.0894530	-3.0531	up
HK1	1.2413663	6.9664546	2.5849763	0.0112371	0.0903197	-3.06584	up
RAB3IL1	0.8420335	8.6557632	2.5838337	0.0112719	0.0904839	-3.06851	up
ARHGEF12	0.7892439	7.4898804	2.5816453	0.0113389	0.0909286	-3.07361	up
FAM49B	0.5184792	9.2094618	2.5802124	0.0113829	0.0911222	-3.07696	up
ATF5	0.5702350	9.4531486	2.5784949	0.0114359	0.0912373	-3.08096	up
TLR4	0.5562977	9.2738766	2.5766665	0.0114926	0.0915072	-3.08522	up
HIST1H4D	0.5286617	6.6278211	2.5755689	0.0115267	0.0916629	-3.08778	up
ITGA2B	0.9178019	9.1847710	2.5734710	0.0115922	0.0919340	-3.09266	up
SIGLEC11	0.5216000	6.0396818	2.5720536	0.0116367	0.0920943	-3.09595	up
ANKRD55	-0.72232	8.8090364	-2.56893	0.0117351	0.0926171	-3.10321	down
CCDC99	-0.51108	7.0240590	-2.56864	0.0117444	0.0926202	-3.10389	down
SLC16A3	0.5303836	12.069770	2.5680143	0.0117641	0.0926831	-3.10534	up
AQP10	0.9094294	8.3258920	2.5675639	0.0117784	0.0926859	-3.10639	up
TSHZ2	-0.52527	7.3029721	-2.56706	0.0117943	0.0927448	-3.10755	down
CA1	1.4674834	12.069438	2.5613075	0.0119786	0.0938551	-3.1209	up

C1001299	0.5916845	7.0395450	2.5556686	0.0121616	0.0946754	-3.13395	up
FPR1	0.6278770	13.548748	2.5503542	0.0123364	0.0955392	-3.14623	up
PGS1	0.5840285	11.281817	2.5493659	0.0123692	0.0957456	-3.14851	up
CD300C	0.5045302	8.5348973	2.5492515	0.0123730	0.0957464	-3.14877	up
HDAC4	0.6155609	8.0356918	2.5476517	0.0124262	0.0960922	-3.15246	up
CISD2	0.7839594	9.4278792	2.5433576	0.0125700	0.0967571	-3.16236	up
TMEM88	0.5929838	6.8171619	2.5419506	0.0126174	0.0970459	-3.1656	up
WARS	0.5978443	11.951977	2.5381139	0.0127476	0.0977363	-3.17442	up
FPR2	0.6848205	11.303188	2.5363147	0.0128091	0.0980843	-3.17856	up
SLC14A1	0.9061764	9.3411259	2.5348623	0.0128589	0.0983736	-3.1819	up
LOC728790	0.5945653	7.6984462	2.5340191	0.0128879	0.0984756	-3.18383	up
LOC729915	0.6666069	6.6647548	2.5331928	0.0129164	0.0985979	-3.18573	up
CD3G	-0.66908	11.248652	-2.53319	0.0129165	0.0985979	-3.18573	down
MS4A3	0.7622724	8.3884137	2.5329076	0.0129262	0.0986485	-3.18638	up
C1001296	0.6339907	10.465585	2.5300194	0.0130263	0.0992193	-3.19301	up
RFESD	0.5386778	8.0585392	2.5256916	0.0131775	0.1001042	-3.20292	up
C1001295	-0.52399	8.2694401	-2.52486	0.0132069	0.1001821	-3.20483	down
STK3	0.5133005	8.4213760	2.5243662	0.0132241	0.1002646	-3.20595	up
IL1B	0.6309519	10.655641	2.5173516	0.0134734	0.1017126	-3.22198	up
TFPI	0.5783137	7.1504157	2.5171987	0.0134789	0.1017162	-3.22233	up
TLR1	0.8380812	8.7559549	2.5170652	0.0134837	0.1017168	-3.22264	up
CDKN1C	-0.71843	8.3118096	-2.51667	0.0134979	0.1017995	-3.22354	down
LOC643031	-0.58065	11.781653	-2.51516	0.0135521	0.1020371	-3.22698	down
ANKDD1A	0.5149615	8.7437370	2.5139666	0.0135952	0.1022390	-3.2297	up
VNN2	0.5448210	13.331257	2.5107198	0.0137129	0.1028537	-3.2371	up
TMEM86B	0.6778664	9.3092532	2.5052529	0.0139132	0.1037863	-3.24954	up
P2RX5	-0.57202	7.5412897	-2.50122	0.0140627	0.1046009	-3.2587	down
FLJ11795	-0.65335	8.5825961	-2.50114	0.0140657	0.1046009	-3.25888	down
SEPX1	0.5351669	14.004925	2.4976073	0.0141977	0.1053586	-3.26689	up
LOC644928	-0.59567	12.00221	-2.49577	0.0142670	0.1057231	-3.27106	down
NTNG2	0.6554767	7.2448724	2.4932594	0.0143619	0.1062254	-3.27674	up
LOC644934	-0.50995	12.501986	-2.48347	0.0147378	0.1080648	-3.29887	down
TLR8	0.56177	11.695621	2.4813483	0.0148202	0.1085426	-3.30364	up
NFIA	0.5348521	6.7797725	2.4805650	0.0148508	0.1086907	-3.30541	up
GZMK	-0.69749	11.306854	-2.47747	0.0149724	0.1092498	-3.31238	down
LOC283663	-0.66672	8.5301777	-2.47462	0.0150850	0.1096892	-3.31879	down
LIMK2	0.6723841	7.7218371	2.4728018	0.0151571	0.1099786	-3.32287	up
HSPA1A	0.7012517	10.786269	2.4727342	0.0151598	0.1099786	-3.32303	up
FOS	0.5244119	10.259391	2.4699921	0.0152694	0.1106460	-3.32918	up
LOC653867	0.5294124	7.0006362	2.4695621	0.0152866	0.1107199	-3.33015	up
IGSF6	0.5801502	10.265142	2.4653773	0.0154553	0.1116076	-3.33954	up
RNF123	0.5509147	9.2156169	2.4621200	0.0155878	0.1123577	-3.34683	up
IGF2BP3	0.5777468	8.3931779	2.4595833	0.0156917	0.1128257	-3.35251	up
LOC644695	0.5629956	6.8253867	2.4591960	0.0157076	0.1128588	-3.35337	up
AJC25-GNG	0.6071661	7.1675274	2.4550707	0.0158780	0.1137892	-3.36259	up
FCGR3B	0.5248297	13.577953	2.4544763	0.0159027	0.1139056	-3.36392	up
ESAM	0.6249576	8.8785845	2.4514590	0.0160285	0.1145102	-3.37065	up
CEACAM3	0.6538995	8.4726595	2.4496242	0.0161055	0.1149555	-3.37474	up
C1orf24	0.5524660	12.927837	2.4408660	0.0164775	0.1171315	-3.39423	up
C18orf32	0.5546528	7.0723705	2.4397079	0.0165272	0.1173259	-3.3968	up
C1001338	-0.55143	9.0379919	-2.42932	0.0169795	0.1194307	-3.41983	down

IL32	-0.52654	9.9850717	-2.42662	0.0170988	0.1200991	-3.4258	down
SPATS2L	0.6779179	9.2724436	2.4265648	0.0171013	0.1200991	-3.42592	up
C6orf190	-0.68368	9.2992541	-2.42532	0.0171564	0.1203516	-3.42866	down
ROPN1L	0.5909121	9.2489652	2.4237493	0.0172265	0.1206180	-3.43214	up
SIRPB1	0.5590293	7.8179377	2.4232878	0.0172472	0.1206963	-3.43316	up
NFIX	0.7264877	9.0887197	2.4202418	0.0173837	0.1212697	-3.43988	up
LOC401640	-0.57177	8.3504868	-2.41783	0.0174927	0.1217598	-3.4452	down
LIN7A	0.6350746	7.2588397	2.4104799	0.0178280	0.1233557	-3.46136	up
SOD2	0.5861809	6.7293916	2.4078084	0.0179513	0.1240451	-3.46723	up
FAM89A	0.5289535	8.4053938	2.4073865	0.0179709	0.1241252	-3.46816	up
OAS3	0.7571776	10.977740	2.4061617	0.0180277	0.1243542	-3.47084	up
F5	0.6458365	8.8155513	2.4050410	0.0180799	0.1245773	-3.4733	up
GNG10	0.6403027	7.6811207	2.4033612	0.0181583	0.1249259	-3.47698	up
LOC642567	0.6021053	7.7830086	2.4025870	0.0181945	0.1250932	-3.47868	up
CCNA1	0.8735870	5.9936230	2.4022962	0.0182082	0.1251595	-3.47932	up
EIF2AK2	0.5478922	11.996865	2.3998662	0.0183224	0.1257250	-3.48464	up
ORM2	0.6819256	6.5066150	2.3949392	0.0185561	0.1267195	-3.49541	up
ANKRD34B	0.8152393	5.5533797	2.3942114	0.0185908	0.1267639	-3.497	up
MIR1974	0.6222533	11.516113	2.3936310	0.0186186	0.1268705	-3.49827	up
CSF3R	0.6168928	10.105445	2.3905781	0.0187651	0.1275648	-3.50493	up
SULT1B1	0.5375609	7.5453944	2.3894838	0.0188179	0.1278405	-3.50732	up
LOC441763	0.5187215	13.310253	2.3844570	0.0190620	0.1288143	-3.51827	up
LOC728650	0.6615009	7.8099161	2.3842835	0.0190705	0.1288325	-3.51865	up
HIST1H2BG	0.5925847	7.9139410	2.3815703	0.0192034	0.1293670	-3.52455	up
LOC650298	-0.5587	9.2385928	-2.38152	0.0192059	0.1293670	-3.52466	down
)C1001289:	-0.50069	12.149524	-2.38123	0.0192201	0.1293982	-3.52528	down
LRG1	0.7432079	11.078114	2.3655488	0.0200056	0.1326568	-3.55927	up
LOC654103	0.5082264	13.672555	2.3591107	0.0203362	0.1342098	-3.57317	up
PROK2	0.6543706	13.310971	2.3546048	0.0205705	0.1352921	-3.58288	up
)C1001341:	-0.5937	7.4151875	-2.34528	0.0210631	0.1375057	-3.60292	down
C6orf105	-0.60756	6.7547470	-2.34353	0.0211564	0.1378852	-3.60666	down
C19orf35	0.5007590	6.9821780	2.3367540	0.0215222	0.1396580	-3.62116	up
AKR1C3	-0.71733	8.7143217	-2.33004	0.0218903	0.1413201	-3.6355	down
MANSC1	0.5602822	7.7893550	2.3288551	0.0219556	0.1415094	-3.63802	up
GSTA5	0.8948518	6.1139153	2.3195453	0.0224763	0.1436868	-3.65783	up
MAP1A	0.5151284	6.4749404	2.3189947	0.0225074	0.1438566	-3.659	up
GYPA	0.8824343	6.4321785	2.3167388	0.0226354	0.1444862	-3.66379	up
)C1001319(	0.6305827	7.4196745	2.3138948	0.0227977	0.1451337	-3.66982	up
OPLAH	0.7942355	7.6113218	2.3073946	0.0231723	0.1463500	-3.68357	up
KLRG1	-0.60099	10.391861	-2.30694	0.0231987	0.1464581	-3.68453	down
NAMPT	0.6746525	12.435922	2.3062369	0.0232396	0.1466277	-3.68602	up
TBC1D8	0.5004655	8.6966232	2.2990691	0.0236601	0.1485947	-3.70114	up
XAF1	0.5360736	12.160636	2.2981647	0.0237136	0.1488416	-3.70305	up
BEND7	0.6467013	7.2139158	2.2967665	0.0237966	0.1490306	-3.70599	up
KCNJ2	0.5495556	10.877064	2.2967102	0.0238000	0.1490306	-3.70611	up
MCTP1	0.5658194	9.5136069	2.2920839	0.0240763	0.1500061	-3.71584	up
CMPK2	0.6704886	8.0390807	2.2902424	0.0241871	0.1504911	-3.71971	up
OLAH	0.5279125	6.6717174	2.2900810	0.0241969	0.1504957	-3.72005	up
YOD1	0.7720095	11.519353	2.2886040	0.0242861	0.1508443	-3.72315	up
CTNNAL1	0.6476185	8.8425698	2.2798199	0.0248227	0.1530518	-3.74155	up
SRXN1	0.5586069	10.143803	2.2751257	0.0251137	0.1543043	-3.75135	up

LOC644937	-0.5896	8.7116088	-2.27355	0.0252123	0.1547251	-3.75465	down
KLHL14	-0.56782	7.4707497	-2.27122	0.0253585	0.1553584	-3.75951	down
FAM83A	0.8232023	6.6332418	2.2694412	0.0254702	0.1556721	-3.76321	up
CXCL10	0.7970854	8.8840240	2.2642959	0.0257966	0.1571757	-3.77391	up
IFIT1L	1.2353060	11.250652	2.2636442	0.0258383	0.1572463	-3.77527	up
IDI1	0.5429535	8.4275272	2.2627596	0.0258948	0.1575601	-3.7771	up
CAPN13	0.5173923	5.1273455	2.2603642	0.0260486	0.1579452	-3.78208	up
TBCEL	0.6743477	7.7641551	2.2500038	0.0267228	0.1609156	-3.80353	up
MICAL2	0.6790852	7.8034516	2.2494763	0.0267575	0.1610254	-3.80462	up
WSB2	0.5253411	8.3177538	2.2494156	0.0267615	0.1610254	-3.80475	up
LGALS3	0.6854062	8.9636222	2.2469802	0.0269224	0.1615605	-3.80978	up
FLCN	0.5574259	7.6509346	2.2467374	0.0269385	0.1615644	-3.81028	up
TMEM119	0.8656928	6.1263499	2.2367681	0.0276064	0.1644713	-3.83082	up
XKR3	0.5096168	6.0037450	2.2340813	0.0277888	0.1653307	-3.83634	up
KEL	0.6421852	7.2373104	2.2318886	0.0279385	0.1657901	-3.84084	up
IFI44L	0.8736910	12.739117	2.2284477	0.0281748	0.1667770	-3.84789	up
RPL10A	-0.52025	12.342477	-2.22191	0.0286287	0.1688336	-3.86127	down
APOBEC3A	0.5736809	6.7516074	2.216662	0.0289975	0.1704337	-3.87198	up
CASP5	0.8537908	8.4835228	2.2122230	0.0293128	0.1715804	-3.88103	up
KIFC3	0.5067908	7.6876366	2.2118216	0.0293415	0.1716522	-3.88184	up
TBC1D22B	0.5191378	8.2395495	2.1867310	0.0311820	0.1779798	-3.93265	up
HSD17B11	0.5033854	9.0112628	2.1851478	0.0313014	0.1783847	-3.93584	up
PRRG4	0.5204207	7.4778733	2.1803440	0.0316663	0.1796996	-3.9455	up
RMND5A	0.5271952	7.7408677	2.1559165	0.0335793	0.1869715	-3.99433	up
IL8RBP	0.5044848	8.5140554	2.1508625	0.0339875	0.1884484	-4.00437	up
CXCR1	0.5754682	10.737485	2.1458451	0.0343969	0.1900144	-4.01431	up
PYGL	0.5173798	11.820087	2.1300437	0.0357144	0.1944891	-4.0455	up
LOC347376	0.5756494	11.051876	2.1274313	0.0359363	0.1952918	-4.05064	up
ERAP2	-0.64573	11.274965	-2.1251	0.0361357	0.1960362	-4.05522	down
LOC654053	0.6677584	7.6471628	2.1196014	0.0366088	0.1978514	-4.066	up
)C1000085	0.6251631	10.240958	2.1180053	0.0367472	0.1981929	-4.06912	up
SIGLEC1	0.5481697	6.0181288	2.1082149	0.0376061	0.2012687	-4.08824	up
SNORD8	0.6628922	9.4197436	2.1009545	0.0382543	0.2029006	-4.10237	up
CHI3L2	-0.50616	6.9312046	-2.08981	0.0392682	0.2063686	-4.12398	down
TSPAN9	0.5573928	9.6078974	2.0805471	0.0401279	0.2092850	-4.14184	up
PTGES3	0.5523685	9.3224370	2.0738435	0.0407604	0.2115922	-4.15473	up
FAM46C	0.6737858	13.622843	2.0645162	0.0416547	0.2148503	-4.1726	up
VSTM1	-0.59397	7.9802073	-2.06118	0.0419789	0.2158497	-4.17898	down
MPP1	0.6256135	12.884864	2.0584747	0.0422429	0.2166044	-4.18414	up
HPS1	0.6427366	8.8734423	2.0546788	0.0426161	0.2178073	-4.19137	up
RGL4	0.5376872	10.613626	2.0540595	0.0426773	0.2179781	-4.19255	up
C14orf45	0.6821220	8.8437945	2.0446736	0.0436134	0.2211423	-4.21037	up
RSAD2	0.8788998	10.738975	2.0342908	0.0446695	0.2245048	-4.23001	up
TCEA3	-0.52852	8.6062743	-2.01556	0.0466299	0.2301887	-4.26519	down
HBBP1	0.6872860	7.7543547	2.0072590	0.0475222	0.2327019	-4.28069	up
TMEM158	0.8053234	10.007281	2.0058813	0.0476716	0.2331069	-4.28326	up
KLC3	0.6727324	8.0845173	2.0002970	0.0482816	0.2346311	-4.29365	up
