# Supplementary material for: Exploring T-cell metabolism in tuberculosis: development of a diagnostic model using metabolic genes
Source: Eur J Med Res. 2025 Jun 16;30:483. doi: 10.1186/s40001-025-02768-0 (PMC12168305; doi:10.1186/s40001-025-02768-0)
Supplement: Supplementary file 5 — Supplementary Material 5 [file 40001_2025_2768_MOESM5_ESM.docx]

**Supplementary Table 1. Hyperparameters Used in Each Machine Learning Model**

| Model | Hyperparameters | Package R version |
| --- | --- | --- |
| XGBoost | learning rate = 0.1, max_depth = 6, n_estimators = 100 | xgboost v1.7.8.1 |
| Support Vector Machine (SVM) | kernel = RBF, C = 1.0, gamma = 'scale' | e1071 v1.7-16 |
| Random Forest | n_estimators = 100, max_depth = None, max_features = 'sqrt' | randomForest v4.7-1.2 |
| AdaBoost | n_estimators = 50, learning_rate = 1.0 | fastAdaboost v1.0.0 |
| LogitBoost | n_estimators = 100, learning_rate = 0.1 | caTools v1.18.3 |
| Partitioning Around Medoid (PAM) | number of clusters = 3, distance = Euclidean | pamr v1.57 |
| Naive Bayes | no tunable hyperparameters | caret v6.0-94 |
| Neural Network | hidden_layer_sizes = (100,), activation = 'relu', solver = 'adam', max_iter = 200 | caret v6.0-94 |
| Bagged CART | base_estimator = DecisionTreeClassifier(), n_estimators = 100, bootstrap = True | caret v6.0-94 |
