# Supplementary material for: Exploring T-cell metabolism in tuberculosis: development of a diagnostic model using metabolic genes
Source: Eur J Med Res. 2025 Jun 16;30:483. doi: 10.1186/s40001-025-02768-0 (PMC12168305; doi:10.1186/s40001-025-02768-0)
Supplement: Supplementary file 6 — Supplementary Material 6 [file 40001_2025_2768_MOESM6_ESM.docx]

| Characteristics | TB (n=10) | LTBI (n=10) | P-value |
| --- | --- | --- | --- |
| Age (years) | 67.9±10.65 | 52.4±20.01 | 0.048 |
| Gender (Male/Female) | 5/5 | 6/4 | 0.72 |
| Smoking history (Yes/No) | 4/6 | 2/8 | 0.29 |
| Types of tuberculosis infection | ATB | LTBI | — |
| Average blood pressure (mmHg) | 117/80 | 118/79 | 0.42 |

**Supplementary Table 2. Demographic characteristics**
