# Supplementary material for: Exploring T-cell metabolism in tuberculosis: development of a diagnostic model using metabolic genes
Source: Eur J Med Res. 2025 Jun 16;30:483. doi: 10.1186/s40001-025-02768-0 (PMC12168305; doi:10.1186/s40001-025-02768-0)
Supplement: Supplementary file 7 — Supplementary Material 7 [file 40001_2025_2768_MOESM7_ESM.docx]

| **Training set performance metrics** | | | | |
| --- | --- | --- | --- | --- |
| Model | Accuracy | Precision | Recall | F1-score |
| XGBoost | 0.9655 | 1 | 0.9512 | 0.9753 |
| Random Forest | 1.0000 | 1.0000 | 1.0000 | 1.0000 |
| LogitBoost | 1.0000 | 1.0000 | 1.0000 | 1.0000 |
| adaBoost | 1.0000 | 1.0000 | 1.0000 | 1.0000 |
| Naive Bayes | 0.9052 | 0.8000 | 0.9474 | 0.8675 |
| NNET | 0.8793 | 0.7857 | 0.8684 | 0.8250 |
| Bagged CART | 1.0000 | 1.0000 | 1.0000 | 1.0000 |
| **Validation set performance metrics,** | | | | |
| Model | Accuracy | Precision | Recall | F1-score |
| XGBoost | 0.8776 | 0.9697 | 0.8649 | 0.9159 |
| Random Forest | 1.0000 | 1.0000 | 1.0000 | 1.0000 |
| LogitBoost | 1.0000 | 1.0000 | 1.0000 | 1.0000 |
| adaBoost | 1.0000 | 1.0000 | 1.0000 | 1.0000 |
| Naive Bayes | 0.9388 | 0.8824 | 0.9375 | 0.9091 |
| NNET | 0.8776 | 0.7500 | 0.9375 | 0.8333 |
| Bagged CART | 0.898 | 0.7619 | 1.0000 | 0.8649 |
| **External validation set performance metrics** | | | | |

| Model | Accuracy | Precision | Recall | F1-score |
| --- | --- | --- | --- | --- |
| XGBoost | 0.8925 | 0.9139 | 0.96 | 0.9343 |
| Random Forest | 1.0000 | 1.0000 | 1.0000 | 1.0000 |
| LogitBoost | 1.0000 | 1.0000 | 1.0000 | 1.0000 |
| adaBoost | 1.0000 | 1.0000 | 1.0000 | 1.0000 |
| Naive Bayes | 0.8172 | 0.4516 | 1.0000 | 0.6222 |
| NNET | 0.7742 | 0.7000 | 1.0000 | 0.5714 |
| Bagged CART | 0.7097 | 0.3333 | 0.9286 | 0.4906 |

**Supplementary Table 3. Machine Learning Performance Metrics**
